# Comparative proteomic analysis of serum from nonhuman primates administered BIO 300: a promising radiation countermeasure

**DOI:** 10.1038/s41598-020-76494-4

**Published:** 2020-11-09

**Authors:** Michael Girgis, Yaoxiang Li, Junfeng Ma, Miloslav Sanda, Stephen Y. Wise, Oluseyi O. Fatanmi, Michael D. Kaytor, Amrita K. Cheema, Vijay K. Singh

**Affiliations:** 1 Department of Oncology, Lombardi Comprehensive Cancer Center, Georgetown University Medical Center, Washington, DC USA; 2 Division of Radioprotectants, Department of Pharmacology and Molecular Therapeutics, F. Edward Hébert School of Medicine, Uniformed Services University of the Health Sciences, 4301 Jones Bridge Road, Bethesda, MD 20814 USA; 3 Armed Forces Radiobiology Research Institute, Uniformed Services University of the Health Sciences, Bethesda, MD USA; 4 Humanetics Corporation, Edina, MN 55435 USA; 5 Department of Biochemistry, Molecular and Cellular Biology, Georgetown University Medical Center, Washington, DC USA

**Keywords:** Predictive markers, Diagnostic markers

## Abstract

Hematopoietic acute radiation syndrome (H-ARS) and delayed effects of acute radiation exposure (DEARE) are detrimental health effects that occur after exposure to high doses of ionizing radiation. BIO 300, a synthetic genistein nanosuspension, was previously proven safe and effective against H-ARS when administered (via the oral (*po*) or intramuscular (*im*) route) prior to exposure to lethal doses of total-body radiation. In this study, we evaluated the proteomic changes in serum of nonhuman primates (NHP) after administering BIO 300 by different routes *(po* and *im*)*.* We utilized nanoflow-ultra-performance liquid chromatography quadrupole time-of-flight mass spectrometry (NanoUPLC-MS/MS) methods for comprehensive global profiling and quantification of serum proteins. The results corroborate previous findings that suggest a very similar metabolic profile following both routes of drug administration. Furthermore, we observed minor alterations in protein levels, 2 hours after drug administration, which relates to the C_max_ of BIO 300 for both routes of administration. Taken together, this assessment may provide an insight into the mechanism of radioprotection of BIO 300 and a reasonable illustration of the pharmacodynamics of this radiation countermeasure.

## Introduction

Nuclear threats and accidents have continued to be a major concern for the United States government. Unsupervised possession of nuclear weapons can lead to a massive loss of life and property^1^. Furthermore, the constant dependence on nuclear power as an alternative source of energy has led to the spread of nuclear plants worldwide^2^. Due to their catastrophic potential, nuclear threats and accidents remain a priority for major federal entities and public health agencies^3^. Accidental exposure to high doses of radiation can lead to a variety of potentially lethal syndromes^4^. Hematopoietic acute radiation syndrome (H-ARS) is the primary cause of death in humans and occurs within 30–60 days following > 2 Gy total-body irradiation due to bone marrow failure^5^. Developing new medical countermeasures to combat radiation injury, especially H-ARS, continues to be a primary focus for numerous research institutions^6^. There are multiple classes of medical countermeasures for radiation exposure. They are defined based on the time of countermeasure administration in relation to radiation exposure. Radioprotectants are agents which are administered prior to radiation exposure to protect individuals. Radiomitigators are administered shortly after radiation exposure, but before the appearance of symptoms of radiation injury. Finally, radiotherapeutics are administered once clinical symptoms of radiation exposure are present. Radioprotectants and radiomitigators can also be used in clinical oncology to minimize the side effects of radiotherapy. Moreover, personnel at risk of unexpected radiation exposure (e.g., military personnel responding to a radiological/nuclear attack, first responders to radiation incidents, or astronauts exposed to space radiation during periods of high solar activity) can benefit greatly from the prophylactic administration of these agents.

The US Food and Drug Administration (US FDA) has approved a small number of radiation countermeasures for H-ARS. Leukocyte growth factors (Neupogen, Leukine, and Neulasta) are radiomitigators for the recovery of radiation-induced bone marrow failure. However, these agents may cause significant adverse effects and thus require close patient monitoring upon administration^7, 8^. Amifostine and palifermin are two other FDA approved radioprotectants that act as cytoprotective agents and are also associated with serious side effects^9, 10^. Researchers have demonstrated the potential toxicity and lethality of using high doses of amifostine^10–13^. Clearly there is a need for new and effective medical countermeasures against radiation exposure.

One such molecule is genistein (5,7-dihydroxy-3-(4-hydroxyphenyl)chromen-4-one), a naturally occurring isoflavone found at low levels in soybeans, which has shown efficacy as a radioprotectant against H-ARS in murine models^14, 15^. Unfortunately, genistein has high hydrophobicity and low water solubility which leads to poor bioavailability and hinders its medical utility^16^. This problem was recently resolved by the development of a pharmaceutically acceptable nanosuspension of genistein (BIO 300) which was manufactured by Humanetics Corporation. This nanoparticle formulation utilizes a wet-nanomilling technique, which enhances water solubility through reducing the average particle size by two orders of magnitude, and hence, enhances its bioavailability^17–19^.

Although extensively studied, the mechanism of radioprotection by genistein remains only partly understood^20^. These studies revealed a broad scope of pharmacological actions of genistein. For example, some studies highlighted its antioxidant and free radical scavenging ability^21–23^, its anti-inflammatory properties^24, 25^, and its ability to arrest hematopoietic stem cells at the G2/M phase of the cell cycle^26^ which induces a senescent state and thus reduces the detrimental effects of radiation exposure. In addition, genistein possesses a benzopyran structural scaffold that gives it estrogen-like biological activity. There are two estrogen-binding ligand-dependent intracellular transcription factors: estrogen receptor-α (ERα) and estrogen receptor-β (ERβ)^27, 28^. Upon ligand activation, these receptors dimerize and translocate to the nucleus and bind to specific DNA transcripts to facilitate site-specific gene transcription. The two transcriptional ERs (α and β) appear to have opposite functions in normal cells. The activation of ERα primarily promotes cellular growth, whereas ERβ negatively regulates ERα and quenches cell proliferation^20^. Genistein (and BIO 300) demonstrated a selective agonistic activity towards ERβ with a half maximal effective concentration (EC_50_) of 0.9 nM, which was a 2000-fold greater potency than ERα^29^. In addition to ERα and ERβ, a third receptor has been identified and termed the G-protein coupled Estrogen Receptor 1 (GPER1). ERα and ERβ are nuclear receptors that are structurally similar, while GPER1 is a less characterized G-protein coupled receptor capable of mediating rapid-translational signal transduction cascades in response to an estrogenic signal^28, 30, 31^.

In previously published work, a global metabolomic approach with an ultra-performance liquid chromatography (UPLC) quadrupole time-of-flight mass spectrometry (QTOF-MS) platform was used to assess the metabolomic and lipidomic changes induced by BIO 300 following *po* or *im* administration as part of the drug’s safety and efficacy evaluation in an NHP model. Transient alterations in phenylalanine, tyrosine, glycerophosphocholine, and glycerophosphoserine reached maximum levels 3–7 days after drug administration and reverted to near-baseline levels 14 days after drug administration. Furthermore, a significant similarity was reported in the metabolic profiles between the serum samples obtained after *po* and *im* administration of BIO 300^32^.

In the present study, we analyzed serum samples obtained from NHPs at 14 different time points (collected before and after drug administration through *po* and *im* routes). The serum samples were immunodepleted, rebuffered, and analyzed for protein concentration. The protein samples were reduced, alkylated, and digested. Tryptic peptides were desalted and analyzed using NanoUPLC-MS/MS methods for comprehensive profiling and quantification of serum proteins. The methods used in this study involve the depletion of most abundant plasma proteins including albumins and globulins, utilizing Data Dependent Acquisition (DDA) in order to develop a library of identified proteins, and subsequently utilizing a label-free Data Independent Acquisition (DIA) SWATH technique for protein quantification.

## Results

### Route of administration of BIO 300 did not significantly impact NHP serum proteomics profile

**Figure 1:**
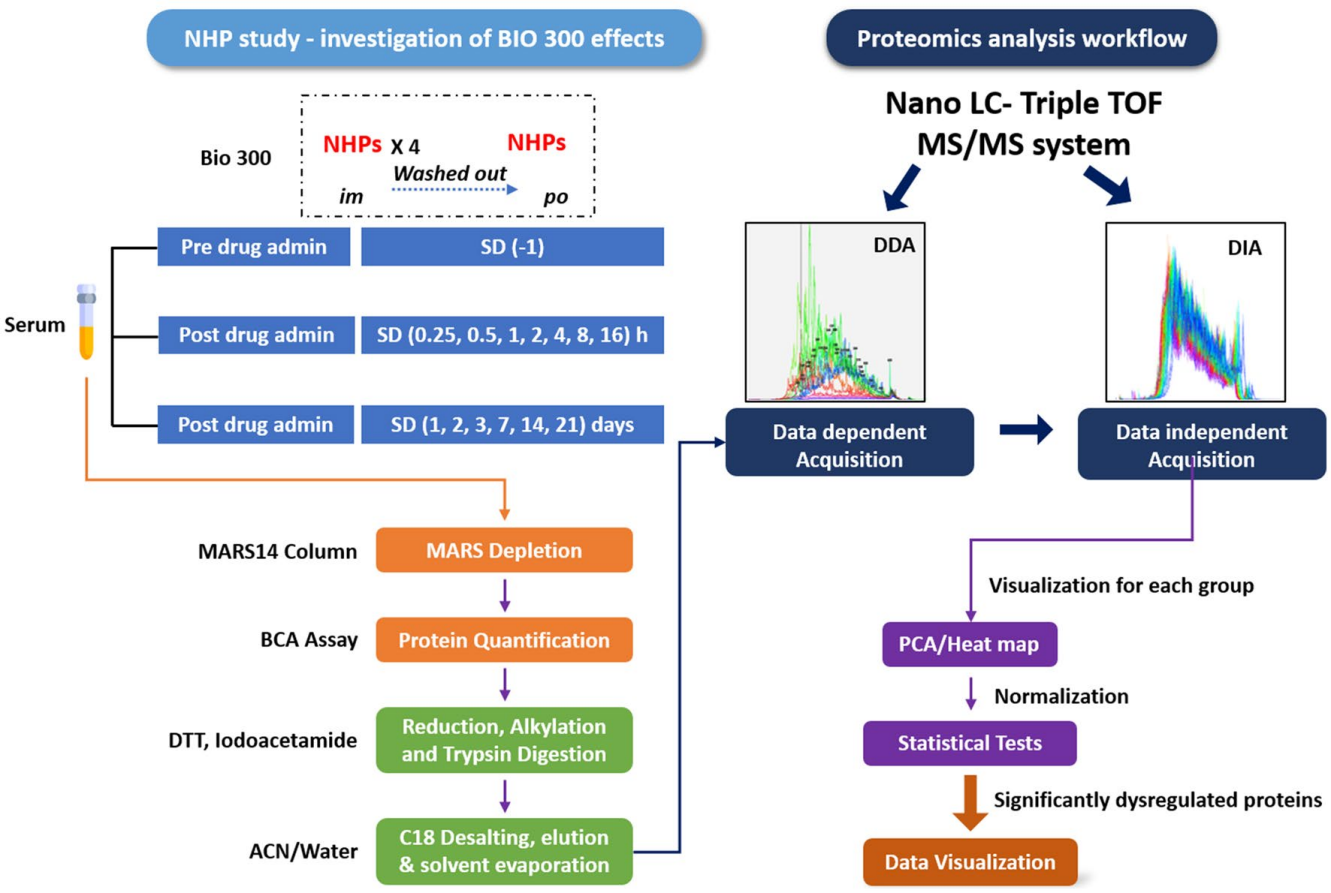
Study design schema.

To understand the impact of *im* versus *po* administration of BIO 300, we analyzed serum samples collected at 14 different time points distributed before and after drug administration (Fig. 1). PCA plot demonstrating the overall perturbation in protein expression across all time points for both routes of administrations is presented in Supplementary Fig. 1. Human database search yielded a total of 468 proteins of which 158 proteins were successfully quantified using Peakview SWATH Microapp. Meanwhile, searching rhesus macaque database, a total of 347 proteins were identified at a level 1% Global FDR, however, only 157 proteins were successfully quantified using Peakview SWATH Microapp.

Because of the high percent homology between humans and NHPs^33^ and the small number of reviewed proteins in the rhesus macaque database compared to the well supported human database, we decided to search both the human and rhesus macaque databases. Therefore, the figures included here were based on the search results of the human database. Supplementary Tables 1 and 2 include a list of identified proteins, their entry identifiers, the corresponding gene names and the length of their canonical sequence as obtained from the human database and rhesus macaque databases, respectively.

**Figure 2:**
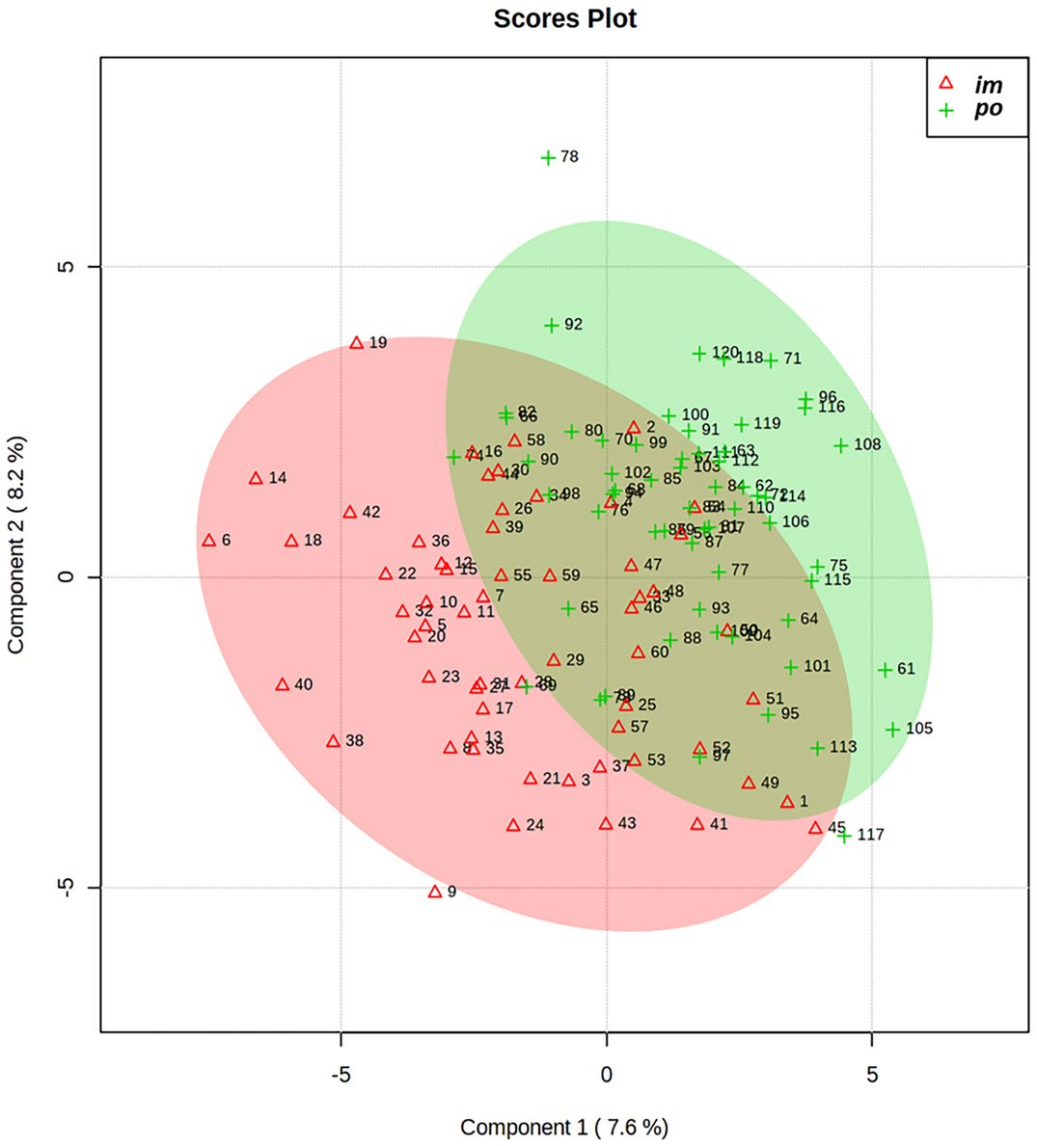
PCA plot demonstrating the overall perturbation in protein expression across all time points for both routes of drug administration.

Principal Component Analysis (PCA), an unsupervised feature reduction algorithm, was used to measure the degree of difference between sample groups; results indicated mostly overlapping proteomic profiles between the two routes of administration throughout all time points (for human database: Fig. 2 and Supplementary Table 1, for rhesus macaque database: Supplementary Fig. 2 and Supplementary Table 2).

### A transient difference in the proteomic profiles of *po* compared to *im* occurred after drug administration which returned to near normal over time.

While comparing the two routes of administration at each individual time point, we observed that there was minimal separation at individual time points, for example 30 min post-drug administration. This mild transient alteration in the proteomic profiles was diminished with time (for human database search: Supplementary Table 3).

**Figure 3:**
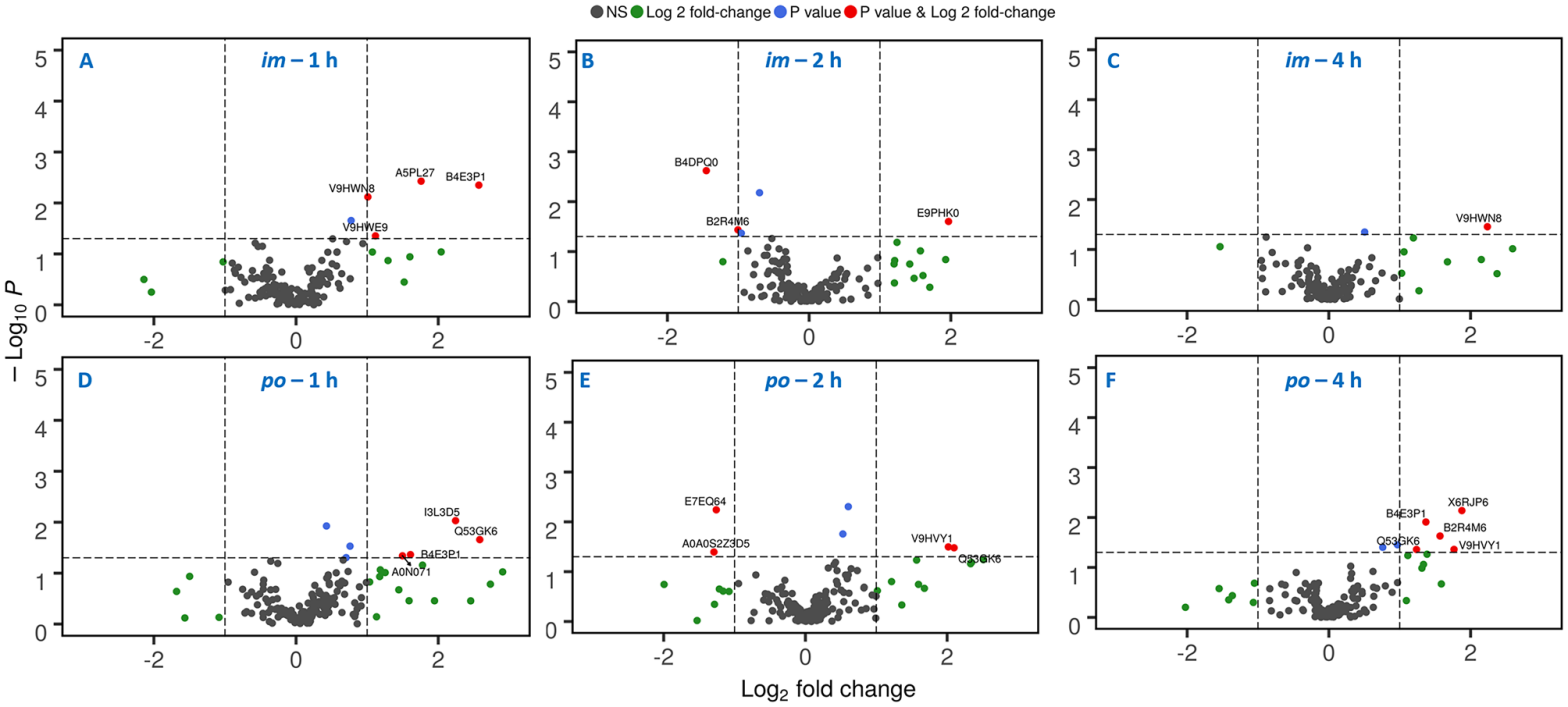
Volcano plots illustrating significantly dysregulated proteins that were selected on the basis of fold change (X-axis) and p-value (Y-axis) at 1 h (**a**, **d**), 2 h (**b**, **e**), and 4 h post-drug administration (**c**, **f**) for both routes compared to pre-administration of the drug (t_-1d_).

Volcano plots further confirmed that there were few significantly dysregulated proteins at 1 h post-administration (Fig. 3). Interestingly, the results showed no downregulated proteins for either route at the 1 h time point. However, a few proteins were upregulated like Glutathione peroxidase (V9HWN8), Glutathione S-transferase pi 1 (V9HWE9), CP protein (A5PL27), and Ceruloplasmin (B4E3P1) in the *im* route. On the other hand, only B4E3P1, Delta globin (A0N071), Beta actin variant (Q53GK6), and I3L3D6 were significantly upregulated following *po* administration (Fig. 3a,d).

Minimal dysregulation was observed at the 2 h time point which is the C_max_ of BIO 300 in both routes. For the *im* route, Complement C1r subcomponent (B4DPQ0) was downregulated, Protein S100 (B2R4M6) was marginally downregulated, but Putative 26S proteasome regulatory subunit 6b (B9PHK0) was upregulated. The *po* route demonstrated a similar pattern. Trypsin-1 (E7EQ64) and Apolipoprotein E isoform 1 (A0A0S2Z3D5) were slightly down regulated, while Q53GK6 and Epididymis secretory sperm binding protein Li 78p (V9HVY1) were slightly upregulated (Fig. 3b,e).

Furthermore, analysis of serum proteomics 2 h after C_max_ did not show any downregulated proteins. Only V9HWN8 was found upregulated in the *im* route. However, several proteins were upregulated in the *po* route like B4E3P1, B2R4M6 and X6RJR6. Meanwhile, V9HVY1 and Q53GK6 were marginally elevated.

Similar patterns of protein expression were observed for both routes of administration at the 30-min and 60-min time points in the results obtained from the rhesus macaque database search (Supplementary Fig. 3).

### Longitudinal analysis of BIO 300 administration revealed minimal changes over time

**Figure 4:**
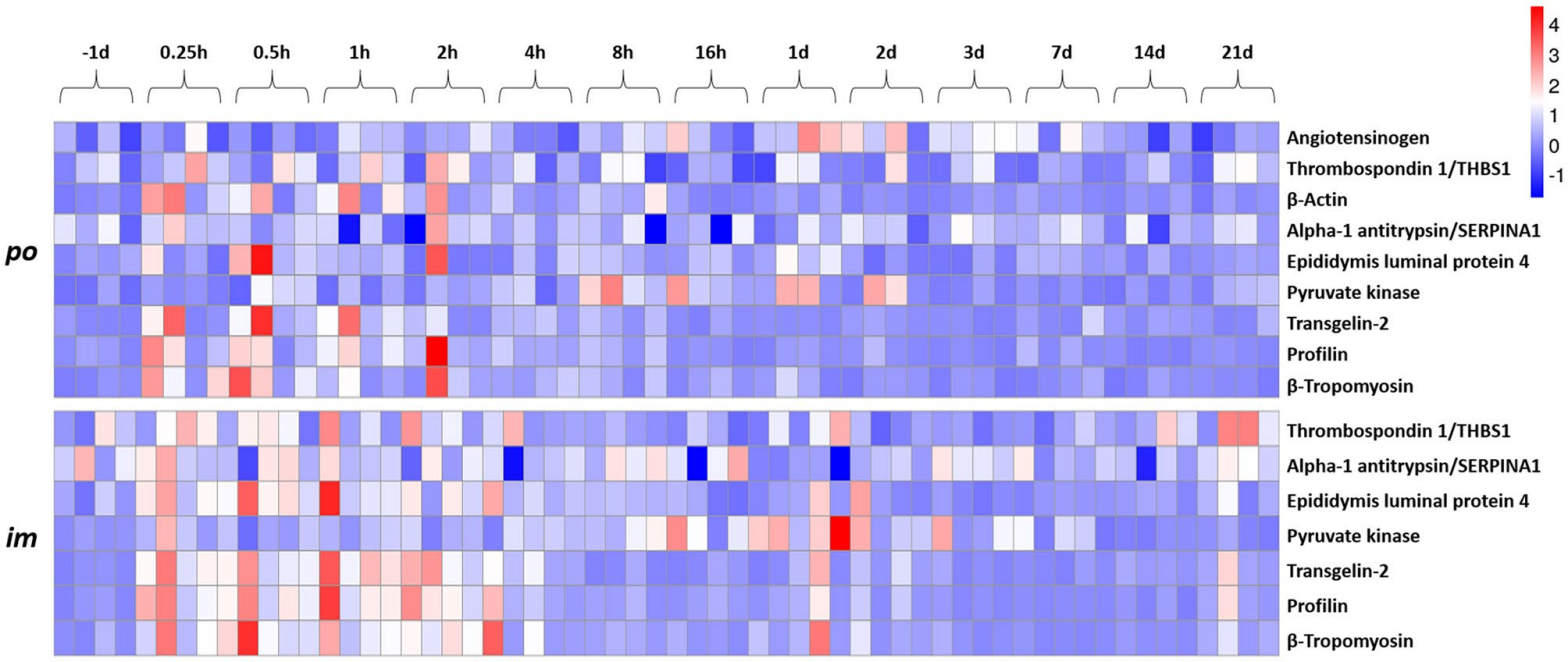
Heatmap showing circulating levels of a subset of proteins on a longitudinal time scale in both routes of administration for BIO 300. At each timepoint (SD; study day) post drug treatment (*po,* top; *im*, bottom). The relative concentration of protein expressed in peak intensity is shown for the 4 individual NHPs. Blue is lower expression and red is higher expression.

To further examine the temporal effects of *im* compared to *po* of BIO 300 administration, we used a machine learning algorithm, and generated a heatmap to identify proteins that were most associated with relative change over time post-drug administration. These proteins, including Transgelin-2 (X6RJP6), Profilin (I3L3D5) and Beta actin variant (Q53GK6) showed transient changes in serum abundance at 30 min and 1 h in both *im* and *po* groups (for human database search: Fig. 4, for rhesus macaque database: Supplementary Fig. 4). However, their levels stabilized between 2 and 16 h post-BIO 300 administration, where they remained close to their normal levels until day 21 (Supplementary Tables 4 and 5).

**Figure 5:**
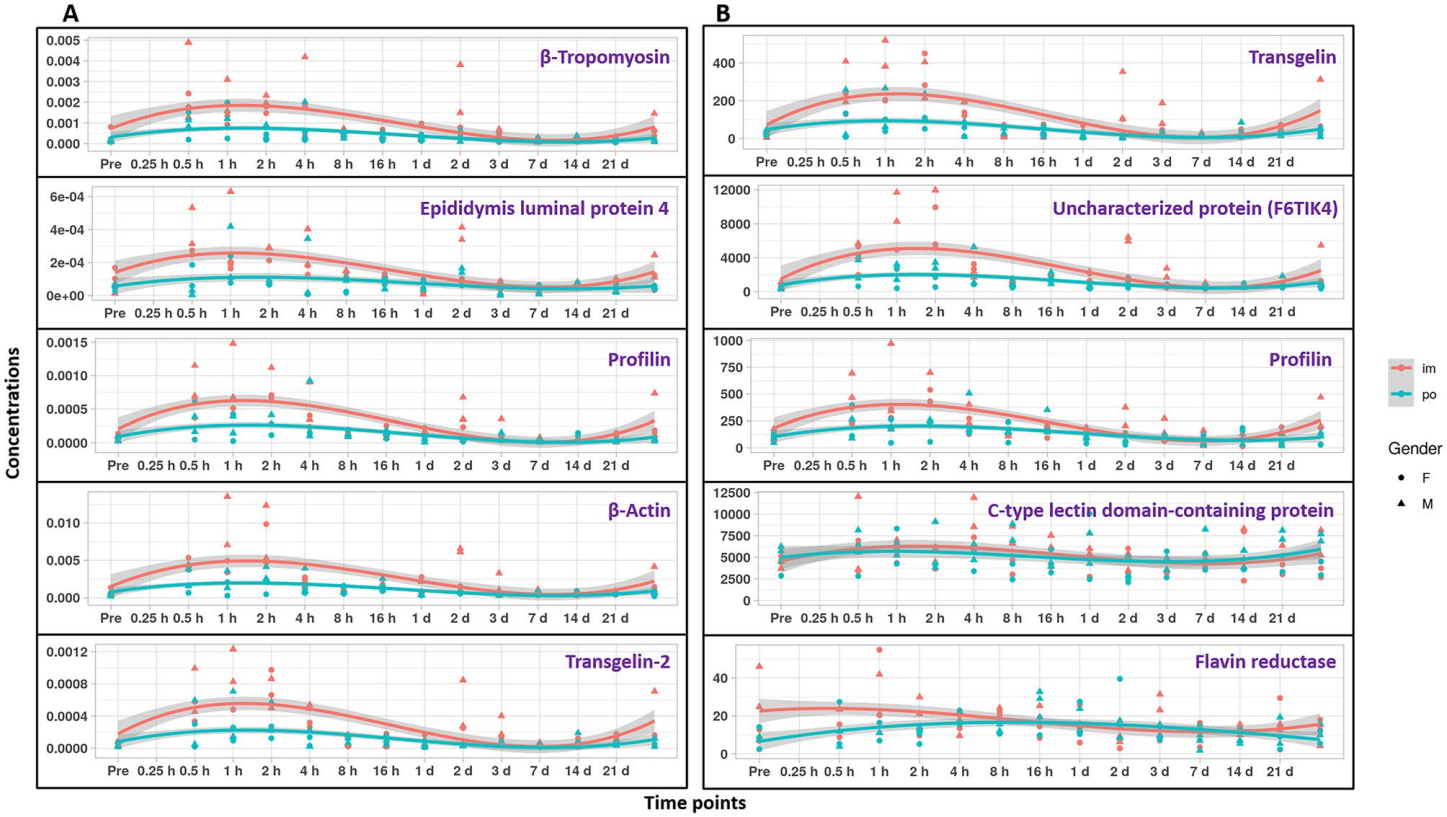
Trend lines of selected proteins that rapidly increase in expression following *po* or *im* administration of BIO 300. Top hits are shown from the search results against the human database (**a**) and the rhesus macaque database (**b**). In both panels, protein levels are represented as relative abundance. Protein levels for individual NHPs are shown as symbols (circle, male; triangle, female). Gray shading is the confidence interval for the trend lines.

**Figure 6:**
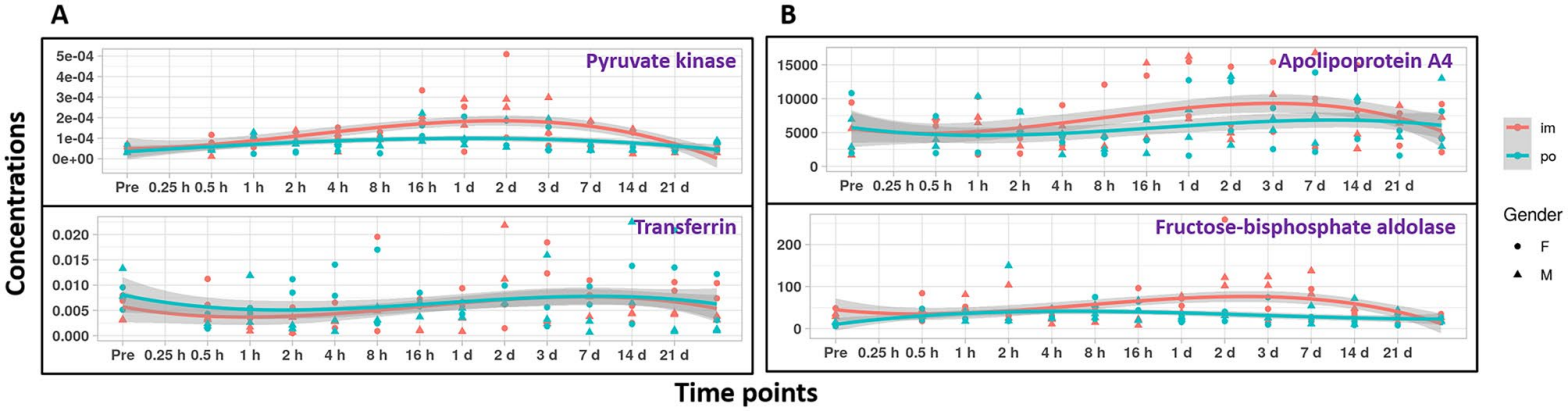
Expression pattern for a subset of proteins after oral as well as intramuscular administration of BIO 300. Panel A represents the search results against the human database. Panel B represents the search results against Rhesus Macaque database. In both panels, the protein level was a slightly elevated.

In addition, we studied the common time trend for each route of administration via trend line analyses. The results showed that certain proteins were slightly upregulated 30 min to 2 h after drug administration by both routes. However, this pattern returned to near normal 2–4 h after administration (Fig. 5 and Supplementary Table 6). On the contrary, some proteins progressively increased in expression starting at 2–4 h following the drug administration by both routes (Fig. 6). These proteins started to return down to baseline 7–21 days post drug administration.

## Discussion

Developing safe and effective radioprotectants has always been the focus of researchers in the past few decades and still a subject of discovery. This is considered very crucial for the safety of our first responders and armed forces who can be under radiological/nuclear threats or involved in responding to accidents. To date, the FDA has approved three countermeasures (Neupogen, Leukine, and Neulasta) for H-ARS which are effective after individuals are already exposed to radiation. Nevertheless, there is no FDA approved agent that can be used prophylactically to prevent either ARS or DEARE. Genistein, as well as other radioprotective agents, have been the subject of multiple reviews^34–37^.

Genistein is a radioprotectant that is believed to have anti-inflammatory activity, cell cycle effects, and estrogen-like pharmacological action with a 2,000-fold binding preference for ERβ over ERα^29^. Either individually or in combination with other agents, genistein has been demonstrated to protect various organs^38–41^. Despite the poor water solubility, genistein was developed as a nanosuspension formulation (BIO 300) utilizing a wet-nanomilling manufacturing process. This genistein nanosuspension (BIO 300) is currently being evaluated for prevention of H-ARS and has an open Investigational New Drug application. In addition, BIO 300 is currently being assessed for safety and efficacy in a phase 1b/2a clinical trial in non-small cell lung cancer (NSCLC) patients (NCT02567799). This new nanosuspension technology has enabled the administration of genistein through different routes such as *po*, subcutaneous (*sc*) or *im*^17, 19, 29^. Furthermore, BIO 300 was shown to be effective in mitigating DEARE to the lung (DEARE-lung) in mice when administered daily via oral gavage for up to 6 weeks, starting 24 h after whole-thorax lung irradiation^19^.

In an earlier study, NHPs were used to compare longitudinal changes in molecular metabolomic and lipidomic profiles following a single dose either *im* or *po* administration of BIO 300^32^. Results of this study revealed that the administration of the drug resulted in modest changes in the metabolic/lipidomic profiles over time. More importantly, the observed changes were most apparent between days 3–7 post BIO 300 administration and this temporal effect was quenched with time. Also, there were no significant differences between the metabolic profiles upon comparing the *po* to the *im* routes as indicated by the AUC calculations of the PK studies performed in the same report.

In this current study, global proteomic profiling of the NHP serum samples was done utilizing a NanoUPLC MS/MS technique. We used a shotgun proteomics approach for global interrogation of alterations in plasma proteomic profiles upon drug administration.

Comparing the two routes of administration at each time point independently, there were few differences. However, the greatest difference was observed at 0.5–1 h post drug administration. This difference might be related to the fact that *im* drug administration may reach the bloodstream faster than the drug administered *po route*. Genistein aglycone levels are ~ 1000 × higher with *im* administration compared to *po* administration 30 min after drug delivery^32^. To further understand the changes happening 0.5 h and 1 h post drug administration, we compared the levels of proteins detected at these time points to the level of the same proteins measured 1 day prior to drug administration.

While a few proteins were found downregulated 0.5 h to 1 h post drug administration, several proteins were upregulated at 0.5 h including Tubulin α chain (B3KT06), cDNA FLJ57036 (B4E3P1), CP protein (A5PL27), Glutathione peroxidase (V9HWN8), Carbonic anhydrase I (V9HWE3), Glutathione S-transferase pi 1 (V9HWE9) and Apolipoprotein E isoform 1 fragment (A0A0S2Z3D5). The levels of these elevated proteins ultimately returned to near normal except for B4E3P1, A5PL27 and V9HWN8. B3KT06, α Tubulin, is a major constituent of microtubules. It binds two molecules of guanosine triphosphate (GTP), one at an exchangeable site on the β chain and one at a non-exchangeable site on the α chain. It has GTPase activity and participates in mitochondrial based processes. B4E3P1 is highly homologous to tropomyosin which is a member of a large family of integral components of actin filaments that play a critical role in regulating the function of actin filaments. A5PL27 is a ceruloplasmin (CP) protein that participates in copper ion binding and iron transport. V9HWN8 is Glutathione peroxidase which is highly secreted in response to oxidative stress. V9HWE9 is Epididymis secretory protein Li 22 that plays an important role in glutathione binding and transfer. Additionally, at 1 h, there were a few upregulated proteins including I3L3D5, Q53GK6, and marginal B4E3P1 and δ-globin (A0N071). Q59EZ3 is an insulin-like growth factor-2 receptor variant that participates in signaling receptor activity and lysosomal transport.

These changes around the 0.5 to 1 h time points demonstrated an increase in proteins that are involved in the biosynthesis and binding of actin proteins which may play an important role in supporting the infrastructure of cellular membranes against radiation exposure. Several studies reported that radiation promotes changes in the distribution of actin^42, 43^. Nitration of the tyrosine residue is one of the post translational modifications that is usually enhanced upon exposure to ionizing radiation due to the production of reactive nitrogen species like nitrogen dioxide. Nitrotyrosine is an indicator or marker of cell damage, inflammation as well as NO (nitric oxide) production. Inhibiting tyrosine nitration of actin has been a proposed mechanism of action of several radioprotectors like amifostine^44^. Our results endorse the hypothesis that BIO 300 exerts its radioprotecting effect through the inhibition of Nitrotyrosine of actin. Furthermore, there was evidence of an elevation in the levels of glutathione peroxidase which is a crucial defensive mechanism against oxidative stress produced by irradiation.

At the C_max_ of BIO 300 in both routes, minor changes were observed. For the *im* route, Complement C1r subcomponent (B4DPQ0), which is a major component in the innate immunity system, was downregulated indicating a potential anti-inflammatory activity of the drug. While protein S100 (B2R4M6), which is a low molecular weight damage associated protein, was marginally downregulated, Putative 26S proteasome regulatory subunit 6b (B9PHK0) was upregulated. Also, the *po* route demonstrated a similar pattern. Trypsin-1 (E7EQ64) and Apolipoprotein E isoform 1 (A0A0S2Z3D5) were slightly downregulated, while Q53GK6 and V9HVY1 were slightly elevated. D0PNI1 is Epididymis luminal protein 4 that has monooxygenase activity and participates in protein domain specific binding. The temporary increase of this protein was associated with a decrease in the levels of phenylalanine and tyrosine at the C_max_ of the drug^32^. These changes returned to near normal levels within the following few hours. This transient elevation in D0PNI1 might be related to the pharmacokinetic effect produced in response to drug absorption into the blood stream.

Furthermore, we used heatmap-based visualization to examine changes in selected proteins along all the time points for the *po* and *im* routes of administration. Interestingly, we noticed similar patterns for both routes. Most of the protein perturbation occurred earlier in time between 30 min to about 1-h post drug administration but these changes gradually disappeared within a few minutes after C_max_. This pattern was clearly observed in a few proteins e.g., I3L3D5, Q53GK6, A0N071, Pyruvate kinase (B4DRT3), Angiotensinogen variant (Q59EP2) and A0A024R9Q1.

Other proteins that showed transient changes in serum abundance included I3L3D5, which is profilin that modulates actin binding and actin cytoskeletal organization, Q53GK6 is β actin variant that is involved in the cytoskeleton structure and A0N071 is a δ-globin that facilitates heme, oxygen and metal binding. B4DRT3 is pyruvate kinase which is involved in kinase activities, magnesium ion binding, and potassium ion binding. Q59EP2 an angiotensinogen variant that regulates systemic arterial blood pressure and X6RJP6 (transgelin-2) which has several biological functions including protein binding were also found to show transient changes in expression post drug administration. Thrombospondin1 (A0A024R9Q1), a cell adhesion molecule was found to decrease in expression 1–2 h post drug administration. Thrombospondin1 plays a role in inducing expression of pro-inflammatory cytokines/genes such as Il-1, IL-6, TNFα and NF-kb^45^. Furthermore, blocking thrombospondin1 signaling is radioprotective of bone marrow in mice^46^. Thus, BIO 300′s radioprotective mechanism could be functioning by inhibiting Thrombospondin1 expression. Meanwhile, Alpha-1 antitrypsin (G3V2W1) did not seem to be significantly changed in both routes of administration throughout all time points. This suggests that the anti-inflammatory capability of BIO 300 is not related to the inhibition of protease enzyme activity.

In addition, we examined the longitudinal alterations along all 14 points for proteins that showed clear perturbation at early time points. We used trend line analyses to examine temporal expression of select proteins after *po* as well as *im* administration of BIO 300. The search results against the human and rhesus macaque databases both revealed that protein level was slightly elevated in serum, especially in the *im* route 30 min to 1 h, meanwhile, this trend was demolished after 1–2 h post-drug administration. Interestingly, gender did not impact the expression pattern of proteins. Proteins D0PNI1, I3L3D5, X6RJP6, B4E3P1, and Q53GK6 showed the same pattern emphasizing the same finding from previous statistical analysis. Q53H26 is transferrin variant that plays an important role in ferric binding and ferric ion transmembrane transport. On the other hand, performing similar analysis on the search results of rhesus macaque database yielded a similar pattern for Transgelin (F6RKI4), F6TIK4 (uncharacterized), Profilin (F7H2A5), Biliverdin reductase (G7NLH4), and C-type lectin domain-containing protein (F7H263). F6RKI4 is a transgelin that facilitates epithelial cell differentiation. F6TIK4 is an uncharacterized protein that is thought to play a role in ATP binding in the synaptic vesicles. F7H2A5 is profilin which regulates actin binding and formation. G7NLH4 is biliverdin reductase B has biliverdin and riboflavin reductase activity and participate in heme catabolism. F7H263 is a C-type lectin domain-containing protein in mannose binding and complement activation.

Moreover, we noticed a temporal late elevation of a few proteins as a result of drug administration between 8 h and 7 days. The search results against the human database yielded proteins B4DRT3 and Q53H26. Meanwhile, the search results against the rhesus macaque database resulted in two proteins: Apolipoprotein A4 (F7FQN6) and Fructose-bisphosphate aldolase (F6SYK0). F7FQN6 has antioxidant activity and participates in cholesterol biosynthesis, transfer and binding. F6SYK0 is Fructose-bisphosphate aldolase in glycolytic process and fructose-bisphosphate aldolase activity.

Taken together, the findings from this study suggest that there were no significant differences in overall serum proteomic profiles resulting from administration of BIO 300 through *im* and *po* routes. Our results demonstrate minor changes in proteomic profiles in sera at/around the C_max_ of the drug. However, these changes subsided to near normal levels over a short period of time. Furthermore, our longitudinal studies suggested that most of the pharmacokinetic alteration in proteins occurred 30 min to 1 h before C_max_. These alterations suggested that BIO 300 may exert its radioprotective activity through an anti-inflammatory function either by hindering the innate complement system or Thrombospondin 1. Furthermore, these temporal changes suggest that BIO 300 contributes to the elevation of actin by inhibiting its nitration post translation modification. The results of the present study support the advanced development of BIO 300 as an effective medical radiation countermeasure.

## Material and methods

### Animals and animal care

A total of four naïve rhesus macaques (*Macaca mulatta*, Chinese sub-strain, two males and two females) 3–7 years of age, weighing 5 to 7 kg, were obtained from National Institutes of Health Animal Center (NIHAC, Poolesville, MD, USA) and maintained in a facility accredited by the Association for Assessment and Accreditation of Laboratory Animal Care (AAALAC)-International. Animals were quarantined for six weeks prior to initiation of the experiment. Animal housing, health monitoring, care, and enrichment during the experimental period have been described earlier^47^. Animals were fed a primate diet (Teklad T.2050 diet; Harlan Laboratories Inc., Madison, WI, USA) twice daily with at least 6 h between feedings (animals were fed four biscuits each at 7:00 AM and 2:00 PM) and received drinking water ad libitum. All procedures involving animals were approved (Protocol # P2017-02-005 approved on 23 February 2017) by the Institutional Animal Care and Use Committee (IACUC, Armed Forces Radiobiology Research Institute) and the Department of Defense Animal Care and Use Review Office (ACURO). This study was carried out in strict accordance with the recommendations in the *Guide for the Care and Use of Laboratory Animals* of the National Institutes of Health.

### Drug preparation and administration

In this study, two formulations of BIO 300 were used, an oral formulation and an injectable formulation. BIO 300 injectable formulation (323 mg/mL genistein, 5% povidone K17 (w/w), 0.2% polysorbate 80 (w/w) in 50 mM phosphate buffered saline (61 mM sodium chloride)) was used for *im* dosing and BIO 300 oral formulation (325 mg/mL genistein, 5% povidone K25 (w/w), 0.2% polysorbate 80 (w/w), 0.18% methylparaben (w/w), and 0.02% propylparaben (w/w)) was used for *po* dosing^32^. These two formulations contain the same active pharmaceutical ingredient (synthetic genistein nanoparticles) but differ in their excipients, which have been optimized for the route of administration. A single dose of BIO 300 injectable formulation (50 mg/kg) using a 23 G needle length of 5/8″ attached to a 1 mL syringe was administered *im* to the four NHPs. The site for injection (thigh quadriceps) was prepared as a surgical site before the injection: hair was clipped using #40 surgical blade and the site was scrubbed at least three times using 4% chlorhexidine and 70% alcohol. The same NHPs used for the *im* study were used for the *po* study after a 31-day wash-out period. BIO 300 oral formulation (100 mg/kg) was administered *po* as a single dose via a nasogastric (NG) tube. NG tube placement was confirmed via digital X-ray prior to drug administration.

### Serum sample collection

Blood samples were collected by venipuncture from the saphenous vein of the lower leg after the site was cleaned using a 70% isopropyl alcohol wipe and dried with sterile gauze. All animals were restrained using the pole-and-collar method and placed in a chair for blood collection. On the day of drug administration, animals were bled repeatedly at 0.25, 0.5, 1, 2, 4, 8, and 16 h post-drug administration^32^. On days when animals were only bled once, the blood draw was conducted between 08:00 AM and 10:00 AM, 1–3 h after animals were fed. A 3 mL disposable luer-lock syringe with a 25-gauge needle was used to collect the desired volume of blood. For serum collection, blood samples were transferred to Capiject serum separator tubes (3T-MG; Terumo Medical Corp, Elkton, MD, USA), allowed to clot for 30 min, and then centrifuged (10 min, 400 × g). Serum samples were stored at − 70 °C until shipped on dry ice to the Georgetown University Medical Center (Washington, DC, USA) for the global proteomic analysis.

### Abundant protein depletion

Serum samples were diluted tenfold using the load/wash buffer supplied by the Agilent Corporation and the remaining particulates in the diluted serum were removed by centrifugation through a 0.22-μm spin filter (Agilent Cat.#: 5185-5990) for 2 min at 16,000 × g. Multiple Affinity Removal System (MARS14, Agilent Cat.#: 5188-6557) columns (4.6 × 50 mm) designed to deplete 14 abundant proteins in serum (albumin, IgG, antitrypsin, IgA, transferrin, haptoglobin, fibrinogen, alpha2-macroglobulin, alpha1-acid glycoprotein, IgM, apolipoprotein AI, apolipoprotein AII, complement C3, and transthyretin) were used according to the manufacturer protocol on a Waters Acquity HPLC system. Protein peaks were collected and transferred to Spin Concentrators, 5 K MWCO, 4 mL capacity (Agilent Cat. #: 5185-5991) and centrifuged at 17,172 × g for 45 min at 4 °C. A volume of 2 × 1 mL of water was added to the concentrated sample and centrifuged at 17,172 × g for 45 min at 4 °C. A volume of 1 mL of 50 mM ammonium bicarbonate buffer pH of approximately 8, was used to rebuffer each sample of concentrated proteins. The final protein concentration was determined using a BCA protein assay kit (Thermo Fisher Scientific Cat. #: 23227).

### Sample preparation

Briefly, protein concentration was adjusted to 10 μg/μL in 50 mM ammonium bicarbonate buffer, reduced with 5 mM Dithiothreitol (DTT) for 60 min at 56 °C and alkylated with 15 mM iodoacetamide for 30 min at 37 °C in the dark. The reaction was quenched by addition of another aliquot of 5 mM DTT followed by digestion with (2.5 ng/μL) Trypsin Gold (Promega, Cat # V5280) at 37 °C overnight, to complete the reaction. Trypsin was deactivated by heating at 90 °C for 10 min and samples were allowed to cool down to room temperature then acidified to a pH = 3 using 0.1% trifluoroacetic acid in water. The digested peptides were purified on microspin C_18_ columns (The Nest Group Inc., HEM S18V) and eluted using 2 × 500 µL of 50% ACN/H_2_O acidified by 0.1% formic acid. The collected fractions were evaporated under vacuum and reconstituted for LC–MS for SWATH analysis.

### Serum proteomics using NanoUPLC-MS/MS

First, we established a protein library of all serum proteins detectable with tandem mass-spectrometry (MS/MS) by the analysis of a sample pool from all the subjects (with equal amount of proteins combined). To get a comprehensive ion library, the pooled peptide sample was fractionated with a 2.1 mm × 100 mm column XTerra MS C_18_ column (Waters) on an Acquity UPLC instrument with a TUV detector in high pH reversed-phase chromatography mode. Solvent A (2% acetonitrile, 4.5 mM ammonium formate, pH 10) and a nonlinear increasing concentration of solvent B (90% acetonitrile, 4.5 mM ammonium formate, pH 10) were used to separate peptides. The 60-min separation LC gradient followed this profile: (min: %B) 0:2; 5:2; 10:10; 40:40; 42:90; 45:90; 50:2, and 60: 2. The flow rate was 0.2 mL/min. A total of twelve fractions were obtained in a step-wise concatenation strategy, followed by acidification to a final concentration of 0.1% formic acid and dried down with a SpeedVac. Peptides in each fraction were then analyzed with a nanoAcquity UPLC system (Waters) coupled with a TripleTOF 6600 mass spectrometer (Sciex), with similar settings as shown previously^48^. Specifically, dried peptides were dissolved into 2% ACN in water plus 0.1% formic acid and loaded onto a C_18_ Trap column (Waters Acquity UPLC Symmetry C_18_ NanoAcquity 10 K 2G V/M, 100 A, 5 μm, 180 μm × 20 mm) at 15 µL/min for 4 min. Peptides were then separated with an analytical column (Waters Acquity UPLC M-Class, peptide BEH C18 column, 300 A, 1.7 μm, 75 μm × 150 mm) which was temperature controlled at 40 °C. The flow rate was set as 400 nL/min. A 60-min gradient of buffer A (2% ACN, 0.1% formic acid) and buffer B (0.1% formic acid in ACN) was used for separation: 1% buffer B at 0 min, 5% buffer B at 1 min, 35% buffer B at 35 min, 99% buffer B at 37 min, 99% buffer B at 40 min, 1% buffer B at 40.1 min, with the column equilibrated with 1% buffer B for 20 min. Data were acquired with the TripleTOF 6600 mass spectrometer using an ion spray voltage of 2.3 kV, GS15 psi, GS2 0, CUR 30 psi and an interface heater temperature of 150 °C. For spectra library generation, the pooled sample (equally combined from 120 samples) was analyzed, and mass spectra were recorded with Analyst TF 1.7 software in DDA mode. Each cycle consisted of a full scan (m/z 400–1800) and fifty (DIAs) (m/z 100–2000) in the high sensitivity mode for precursors with a 2 + to 5 + charge state. Rolling collision energy was used.

Each sample from the cohort was acquired individually via label-free SWATH DIA to quantify proteins in the samples. For SWATH acquisition, each of the samples were injected into the same NanoUPLC-MS/MS system but acquired by repeatedly cycling through 32 consecutive 25-Da precursor isolation windows, generating time-resolved fragment ion spectra for all the analytes detectable within the 400–1250 m/z precursor ion scope.

### Data analysis

For spectral library generation, data files for DDA of the pooled sample fractions were submitted for simultaneous searches using ProteinPilot version 5.0 software (Sciex) utilizing the Paragon, ProGroup algorithms^49^ and the integrated false discovery rate (FDR) analysis function^32, 50^. The MS/MS data was searched against the NCBI Uniprot database of *Homo sapiens* downloaded on Oct. 10th, 2019, and NCBI Uniprot database of rhesus macaque downloaded on Oct. 11, 2019. Trypsin was selected as the digesting enzyme. Carbamidomethylation was set as a fixed modification on cysteine. Variable peptide modifications included methionine (M) oxidation. Other search parameters including instrument (TripleTOF 6600), ID Focus (Biological modifications), search effort (Thorough), FDR analysis (Yes), and user modified parameter files (No) were optimized accordingly. Proteins were inferred based on the ProGroupTM algorithm associated with the ProteinPilot software. The detected protein threshold in the software was set to the value which corresponded to 1% FDR. Peptides were defined as redundant if they had identical cleavage site(s), amino acid sequence, and modification.

For the label-free SWATH quantification, data from each sample was analyzed by PeakView 2.2 (Sciex), with the following settings: (1) Peptide filter: # of peptides per protein: 6; # of transitions per peptide: 6; peptide confidence threshold: 99%; FDR threshold: 1%; (2) XIC Options: XIC extraction window (min): 5; XIC width (ppm): 75. The peak area of each protein was used for protein level quantification. The retention time was calibrated before processing based on a selected set of reference peptides. Quantifiable peptides were carefully chosen, and the peak intensities were normalized based on total ion current (TIC) for further statistical analysis.

### Statistical tests and comparisons

Spectra were searched against Uniprot human database. Peak intensities of identified proteins were then extracted, filtered, TIC normalized and summarized for protein quantification. The p values were calculated either by Mann–Whitney U test or two-tailed unpaired Student *t*-test, whereas, p values of less than 0.05 exclusively were considered significant. Furthermore, p values were corrected for multiple testing using Benjamini–Hochberg procedure which limits the FDRs. Principal-Component Analysis (PCA), Hierarchical Clustering and all statistical analyses were performed using R (version 3.6.1).

## Supplementary information

Supplementary information.

## Data Availability

All data generated or analyzed during this study are included in this published article (and its Supplementary Information files).
