# Supplementary material for: Comparative proteomic analysis of serum from nonhuman primates administered BIO 300: a promising radiation countermeasure
Source: Sci Rep. 2020 Nov 9;10:19343. doi: 10.1038/s41598-020-76494-4 (PMC7653926; doi:10.1038/s41598-020-76494-4)
Supplement: Supplementary file 1 — Supplementary information. [file 41598_2020_76494_MOESM1_ESM.pdf]

Comparative proteomic analysis of serum from nonhuman primates administered BIO 300, a promising radiation countermeasure

Michael Girgis,[1] Yaoxiang Li,[1] Junfeng Ma,[1] Miloslav Sanda,[1] Stephen Y. Wise,[2,3] Oluseyi O. Fatanmi,[2,3] Michael D. Kaytor,[4] Amrita K Cheema,[1,5] and Vijay K Singh[2,3*]

[1]Department of Oncology, Lombardi Comprehensive Cancer Center, Georgetown University Medical Center, Washington, DC, USA; [2]Division of Radioprotectants, Department of Pharmacology and Molecular Therapeutics, F. Edward Hébert School of Medicine, Uniformed Serices University of the Health Sciences, Bethesda, MD, USA; [3]Armed Forces Radiobiology Research Institute, Uniformed Serices University of the Health Sciences, Bethesda, MD, USA; [4]Humanetics Corporation, Edina, MN 55435, USA; [5]Department of Biochemistry, Molecular and Cellular Biology, Georgetown University Medical Center, Washington DC, USA.

1. **Supplementary Figure 1.** An overlay of QC spectra that were run throughout the entire sample set underscoring the reproducibility of data acquisition.

Supplementary Figure 1

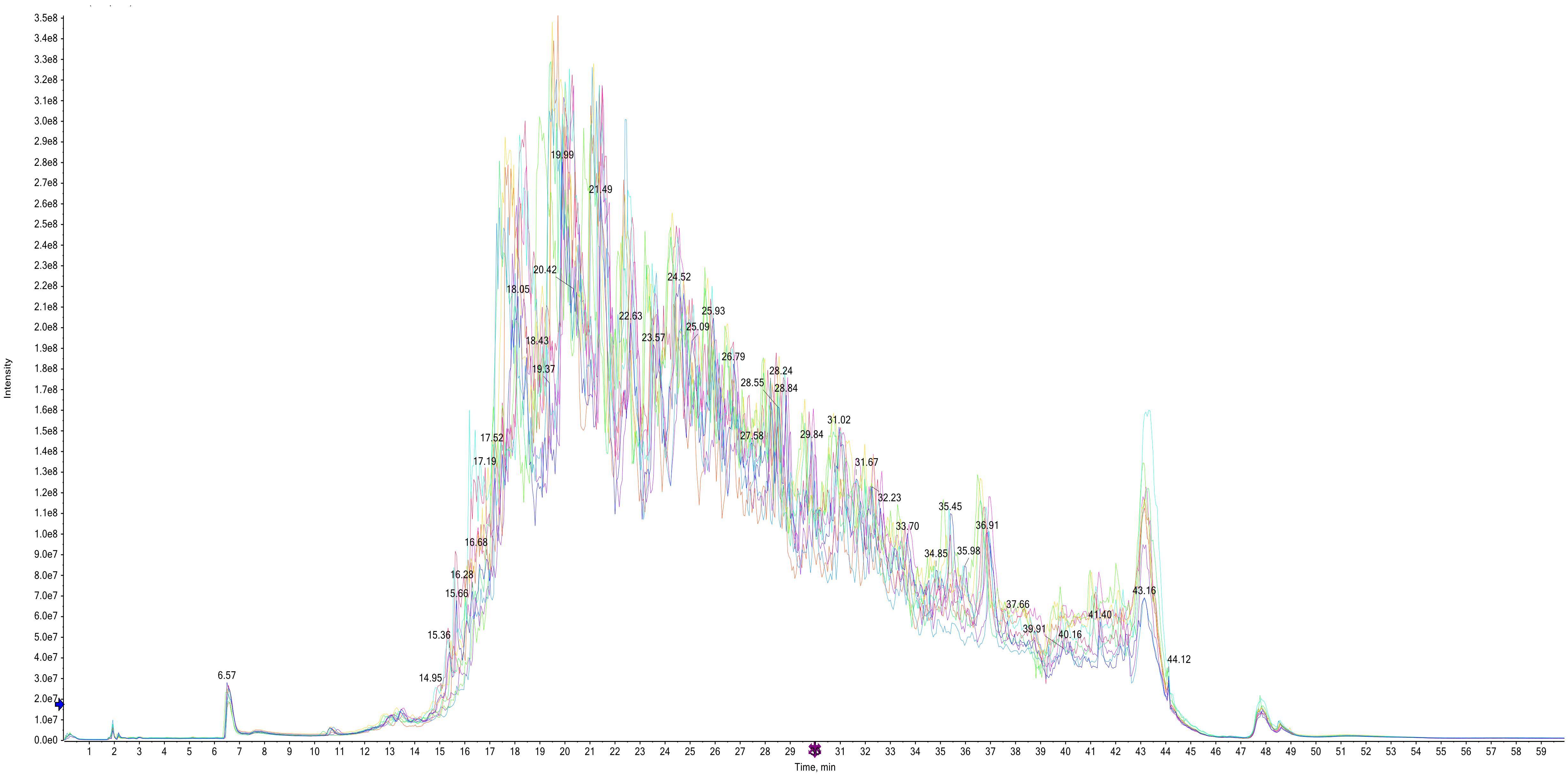

2. **Supplementary Figure 2.** PCA plot demonstrating the overall perturbation in proteins expression across all time points for both routes of administrations. These LC-MS data were searched against rhesus macaque database.

Supplementary Figure 2

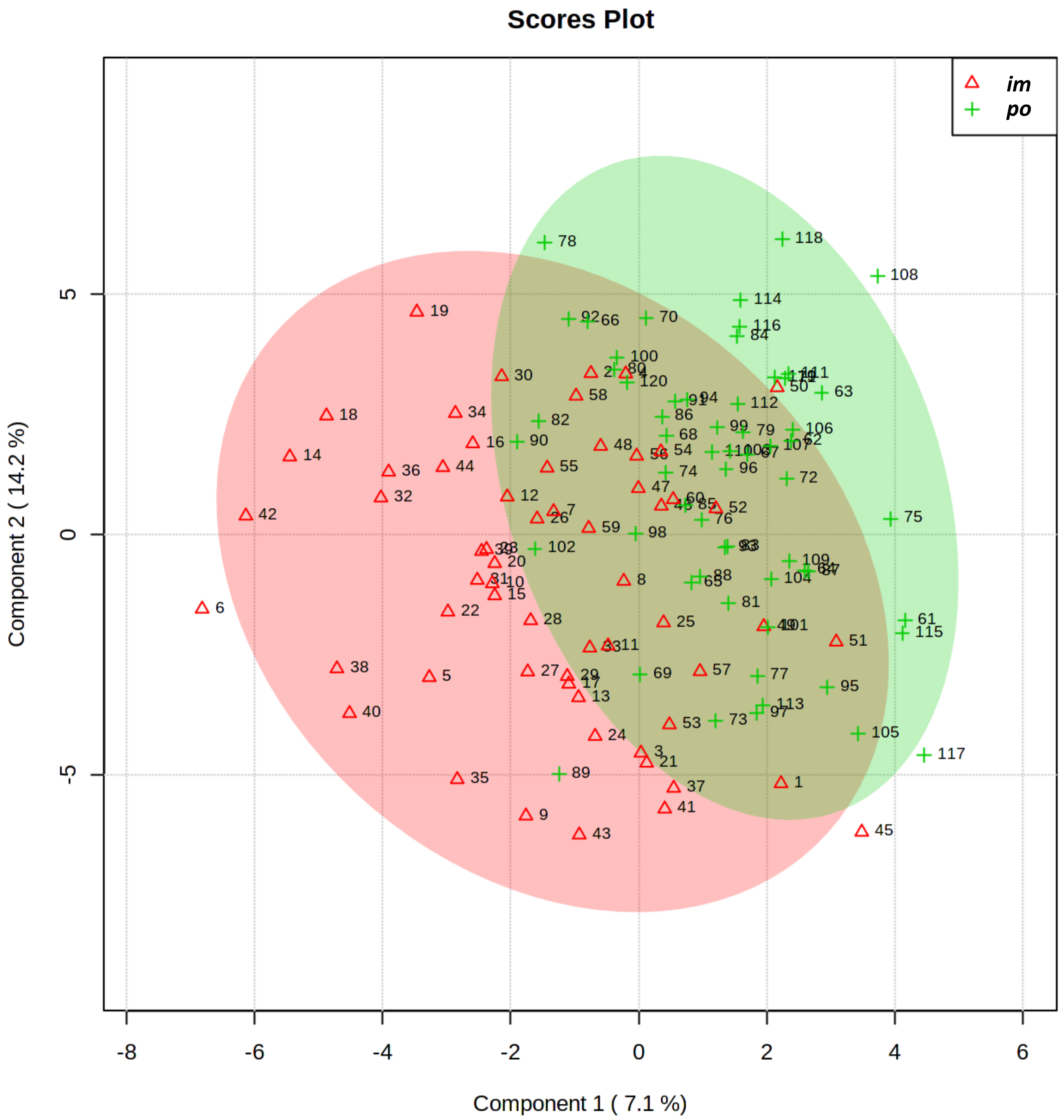

3. **Supplementary Figure 3.** Volcano plots illustrating significantly dysregulated proteins search results against Rhesus Macaque database that were selected on the basis of fold change (X-axis) and p-value (Y-axis) at one hour (panels A and D), two hours (panels B and E), and four hours post-drug administration (panels C and F) for both routes compared to pre-administration of the drug (t-1d).

Supplementary Figure 3

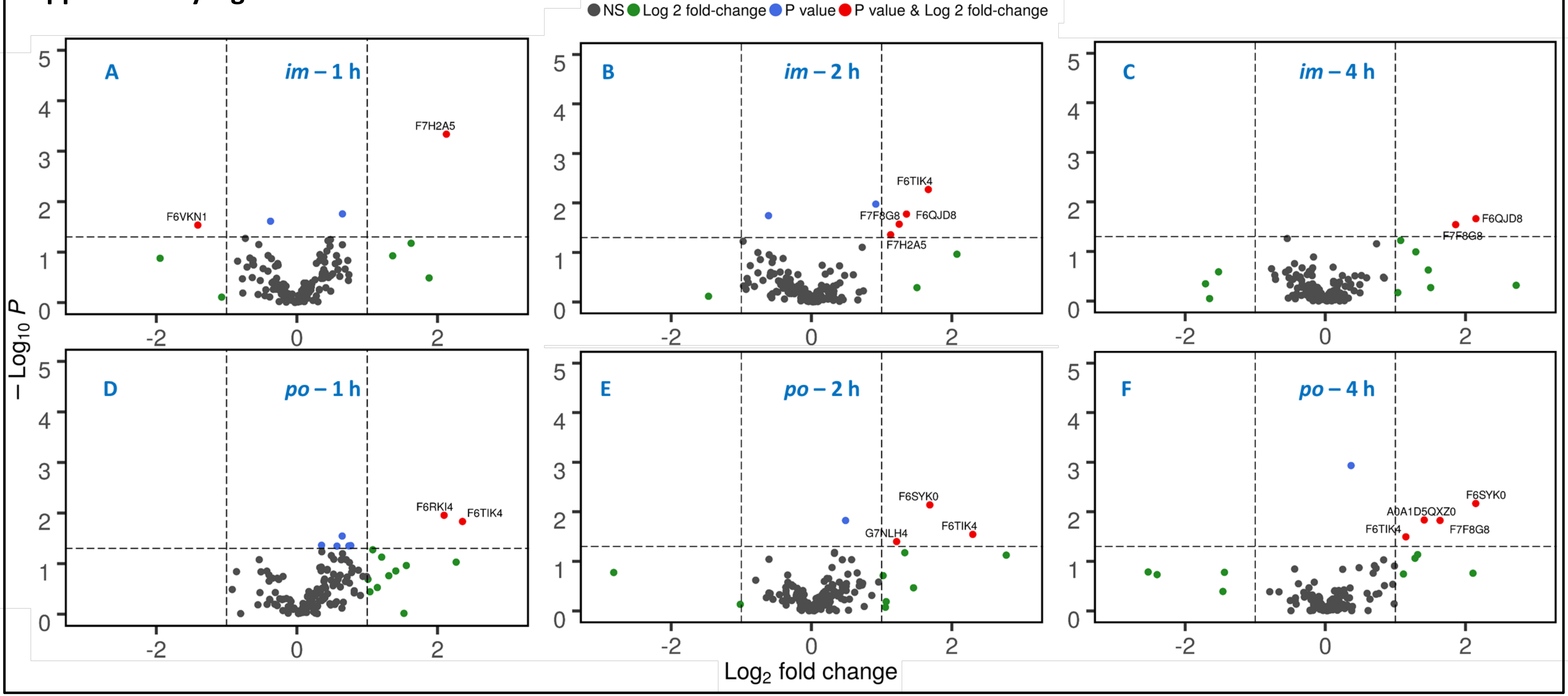

4. **Supplementary Figure 4.** Heat map showing longitudinal dysregulations in the levels of selected proteins throughout 14 different time points in both routes of administration for BIO 300, as obtained from searching Rhesus Macaque database.

Supplementary Figure 4

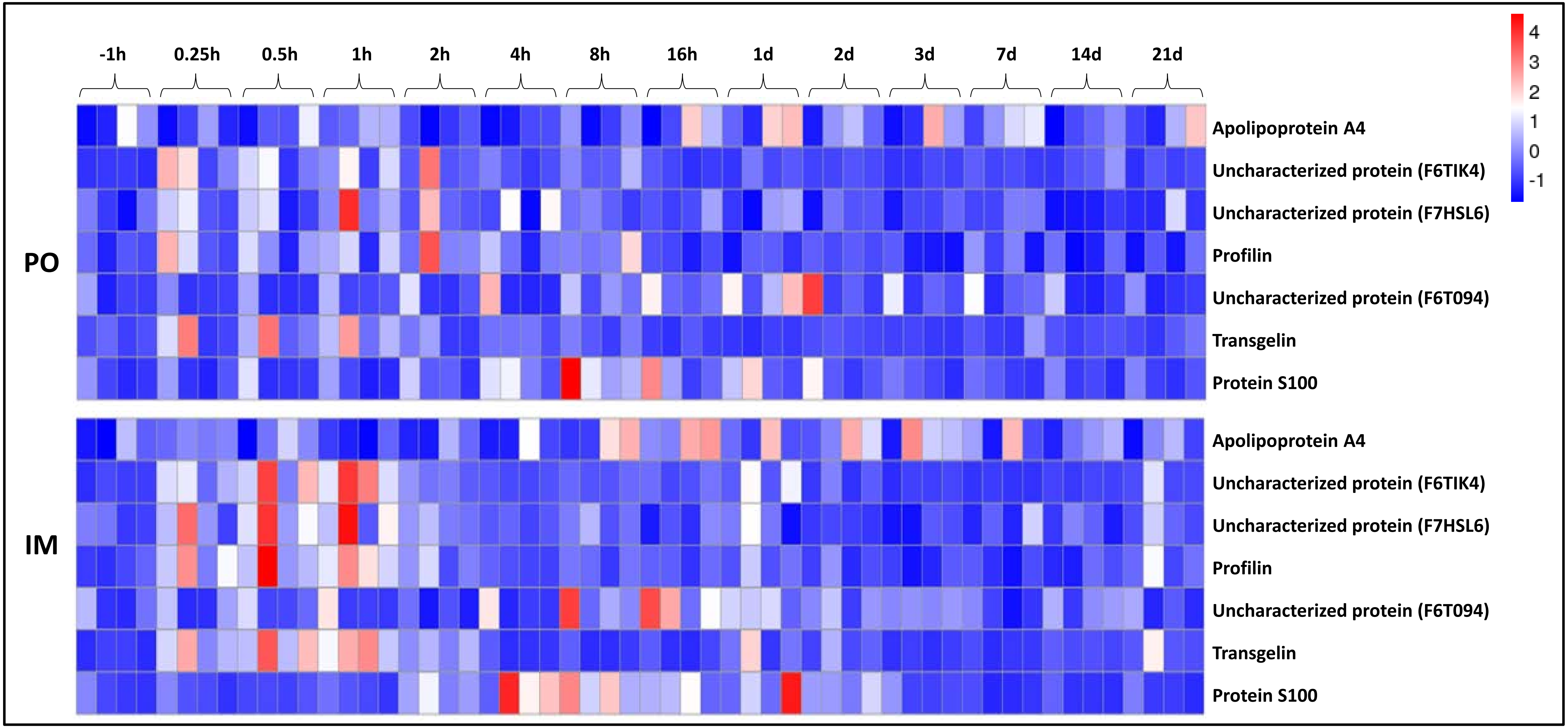

Supplementary Tables

1. **Supplementary Table 1:** contains a list of quantified protein names, their entry identifiers, the corresponding gene names and the length of their canonical sequence as obtained from the human database downloaded on 10/10/2019.
2. **Supplementary Table 2:** contains a list of quantified proteins names, their entry identifiers, the corresponding gene names and the length of their canonical sequence as obtained from the rhesus macaque database downloaded on 10/11/2019.
3. **Supplementary Table 3:** encompasses a list of quantified proteins comparing the *im* to *po* routes at each time point.
4. **Supplementary Table 4:** encompasses a list of quantified proteins comparing the level of proteins as measured at each time point to that measured one hour prior to drug administration for the *im* route.
5. **Supplementary Table 5:** encompasses a list of quantified proteins comparing the level of proteins as measured at each time point to that measured one hour prior to drug administration for the *po* route.
6. **Supplementary Table 6:** the overall effect of drug administration on either route on the level of identified proteins. This comparison was established by dividing the protein level at each time point to the measure value one hour prior to drug administration.

Supplementary Table 1. Database match (Homo sapiens)

Entry	Entry name	Status	Protein names	Gene names	Organism	Length
A0A0S2Z3D5	A0A0S2Z3D5_HUMAN	unreviewed	Apolipoprotein E isoform 1 (Fragment)	APOE	Homo sapiens (Human)	317
A0A024R3E3	A0A024R3E3_HUMAN	unreviewed	Apolipoprotein A-I, isoform CRA_a (Epididymis secretory sperm binding protein)	APOA1 hCG_41332	Homo sapiens (Human)	267
A0A384MEF1	A0A384MEF1_HUMAN	unreviewed	Epididymis secretory sperm binding protein		Homo sapiens (Human)	782
V9HWE9	V9HWE9_HUMAN	unreviewed	Epididymis secretory protein Li 22 (Glutathione S-transferase pi, isoform CRA_b)	HEL-S-22 GSTP1 hCG_21055	Homo sapiens (Human)	210
A0A140VKF3	A0A140VKF3_HUMAN	unreviewed	Testis tissue sperm-binding protein Li 70n		Homo sapiens (Human)	418
O94875	SRBS2_HUMAN	reviewed	Sorbin and SH3 domain-containing protein 2 (Arg-binding protein 2) (ArgBP2) (Arg/Abl-interacting protein 2) (Sorbin)	SORBS2 ARGBP2 KIAA0777	Homo sapiens (Human)	1100
Q6FHY5	Q6FHY5_HUMAN	unreviewed	MEOX2 protein	MEOX2	Homo sapiens (Human)	303
B2R7F8	B2R7F8_HUMAN	unreviewed	Plasminogen (EC 3.4.21.7)		Homo sapiens (Human)	810
V9HWA9	V9HWA9_HUMAN	unreviewed	Epididymis secretory sperm binding protein Li 62p	HEL-S-62p	Homo sapiens (Human)	1663
V9HW98	V9HW98_HUMAN	unreviewed	Epididymis luminal protein 2	HEL2	Homo sapiens (Human)	255
A0A024RAG6	A0A024RAG6_HUMAN	unreviewed	Adiponectin C (Complement component 1, q subcomponent, A chain, isoform CRA_a)	C1QA ADIC hCG_1981379	Homo sapiens (Human)	245
A0A0F7G8J1	A0A0F7G8J1_HUMAN	unreviewed	Plasminogen (EC 3.4.21.7)	PLG	Homo sapiens (Human)	809
V9HVY1	V9HVY1_HUMAN	unreviewed	Epididymis secretory sperm binding protein Li 78p	HEL-S-78p	Homo sapiens (Human)	491
Q9H2E1	Q9H2E1_HUMAN	unreviewed	Neuropilin	NRP1 hCG_19074	Homo sapiens (Human)	704
Q5VY30	Q5VY30_HUMAN	unreviewed	Retinol-binding protein	RBP4 hCG_37964	Homo sapiens (Human)	199
E9PIT3	E9PIT3_HUMAN	unreviewed	Prothrombin	F2	Homo sapiens (Human)	583
D9ZGG2	D9ZGG2_HUMAN	unreviewed	Vitronectin	VTN	Homo sapiens (Human)	478
Q5JP69	Q5JP69_HUMAN	unreviewed	C2 (Complement component 2, isoform CRA_b)	C2 hCG_43694	Homo sapiens (Human)	752
Q59E93	Q59E93_HUMAN	unreviewed	Aminopeptidase (EC 3.4.11.-) (Fragment)		Homo sapiens (Human)	977
Q8NBH6	Q8NBH6_HUMAN	unreviewed	Fibulin-1		Homo sapiens (Human)	638
H3BRJ9	H3BRJ9_HUMAN	unreviewed	Cholesteryl ester transfer protein	CETP	Homo sapiens (Human)	428
V9HWP0	V9HWP0_HUMAN	unreviewed	Pentaxin (Pentraxin)	HEL-S-92n APCS hCG_39427	Homo sapiens (Human)	223
B2R4M6	B2R4M6_HUMAN	unreviewed	Protein S100 (S100 calcium-binding protein)		Homo sapiens (Human)	114
D6RAR4	D6RAR4_HUMAN	unreviewed	Hepatocyte growth factor activator	HGFAC	Homo sapiens (Human)	662
A0A024R9Q1	A0A024R9Q1_HUMAN	unreviewed	Thrombospondin 1, isoform CRA_a	THBS1 hCG_1787130	Homo sapiens (Human)	1170
D0PNI1	D0PNI1_HUMAN	unreviewed	Epididymis luminal protein 4 (Epididymis secretory protein Li 3) (Epididymis secretory protein Li 93) (Tyrosine 3-monooxygenase/tryptophan 5-monooxygenase activation protein zeta polypeptide) (Tyrosine 3-monooxygenase/tryptophan 5-monooxygenase activation protein, zeta polypeptide, isoform CRA_a)	YWHAZ HEL-S-3 HEL-S-93 HEL4 hCG_2009146	Homo sapiens (Human)	245
Q9NYF3	FA53C_HUMAN	reviewed	Protein FAM53C	FAM53C C5orf6	Homo sapiens (Human)	392
V9HWE3	V9HWE3_HUMAN	unreviewed	Carbonic anhydrase I, isoform CRA_a (Epididymis secretory protein Li 11) (Epididymis secretory sperm binding protein)	HEL-S-11 CA1 hCG_20883	Homo sapiens (Human)	261
C9JEU5	C9JEU5_HUMAN	unreviewed	Fibrinogen gamma chain	FGG	Homo sapiens (Human)	445
M0R2W8	M0R2W8_HUMAN	unreviewed	N-acetylmuramoyl-L-alanine amidase (Fragment)	PGLYRP2	Homo sapiens (Human)	181
A0A0G2JPA8	A0A0G2JPA8_HUMAN	unreviewed	Alpha-2-antiplasmin	SERPINF2	Homo sapiens (Human)	427
A0A0A0MRJ7	A0A0A0MRJ7_HUMAN	unreviewed	Coagulation factor V	F5	Homo sapiens (Human)	2229
B0YJC6	B0YJC6_HUMAN	unreviewed	Vitamin K-dependent protein Z variant 1	PROZ	Homo sapiens (Human)	421
E3UN46	E3UN46_HUMAN	unreviewed	Insulin-like growth factor II transcript variant 3 isoform 1 (Fragment)	IGF2	Homo sapiens (Human)	109
P78492	P78492_HUMAN	unreviewed	Inter-alpha-trypsin inhibitor (Fragment)	ITIL	Homo sapiens (Human)	51
B4E3P1	B4E3P1_HUMAN	unreviewed	cDNA FLJ57036, highly similar to Homo sapiens tropomyosin 2 (beta) (TPM2), transcript variant 2, mRNA		Homo sapiens (Human)	303
Q96K68	Q96K68_HUMAN	unreviewed	cDNA FLJ14473 fis, clone MAMMA1001080, highly similar to Homo sapiens SNC73 protein (SNC73) mRNA		Homo sapiens (Human)	494
D3JV41	D3JV41_HUMAN	unreviewed	C-X-C motif chemokine (Fragment)		Homo sapiens (Human)	126
B4DRY0	B4DRY0_HUMAN	unreviewed	cDNA FLJ54379, highly similar to Keratin, type II cytoskeletal 6E		Homo sapiens (Human)	547
A5PL27	A5PL27_HUMAN	unreviewed	CP protein (Ceruloplasmin (Ferroxidase)) (Ceruloplasmin (Ferroxidase), isoform CRA_a)	CP hCG_1781201	Homo sapiens (Human)	1065
Q53H37	Q53H37_HUMAN	unreviewed	Calmodulin-like skin protein variant (Fragment)		Homo sapiens (Human)	146
B4E1C2	B4E1C2_HUMAN	unreviewed	Kininogen 1, isoform CRA_b (cDNA FLJ56836, highly similar to Kininogen-1)	KNG1 hCG_2021397	Homo sapiens (Human)	644
V9HWN8	V9HWN8_HUMAN	unreviewed	Glutathione peroxidase	HEL-S-75p GPX5 hCG_22448	Homo sapiens (Human)	221
A0A0J9YY65	A0A0J9YY65_HUMAN	unreviewed	Alpha-2-antiplasmin (Fragment)	SERPINF2	Homo sapiens (Human)	255
B4DRT3	B4DRT3_HUMAN	unreviewed	Pyruvate kinase (EC 2.7.1.40)		Homo sapiens (Human)	511
A0A0S2Z4I5	A0A0S2Z4I5_HUMAN	unreviewed	Complement factor properdin isoform 1 (Complement factor properdin, isoform CRA_d) (Fragment)	CFP hCG_29138	Homo sapiens (Human)	469
H6VRG1	H6VRG1_HUMAN	unreviewed	Keratin 1	KRT1	Homo sapiens (Human)	645
F8W876	F8W876_HUMAN	unreviewed	Mannan-binding lectin serine protease 1	MASP1	Homo sapiens (Human)	354
B7Z555	B7Z555_HUMAN	unreviewed	cDNA FLJ55311, highly similar to Complement component C8 beta chain		Homo sapiens (Human)	539
A0A384MDV8	A0A384MDV8_HUMAN	unreviewed	Epididymis secretory sperm binding protein		Homo sapiens (Human)	500
Q8IVC0	Q8IVC0_HUMAN	unreviewed	Serpin peptidase inhibitor, clade D (Heparin cofactor), member 1	SERPIND1	Homo sapiens (Human)	499
U6D6R7	U6D6R7_NEOVI	unreviewed	Fermitin family member 3 (Fragment)	F5H1C6	Neovison vison (American mink) (Mustela vison)	150
U6DZW2	U6DZW2_NEOVI	unreviewed	C-type lectin domain family 3, member B (Fragment)	E9PHK0	Neovison vison (American mink) (Mustela vison)	127
B3KS79	B3KS79_HUMAN	unreviewed	cDNA FLJ35730 fis, clone TESTI2003131, highly similar to ALPHA-1-ANTICHYMOTRYPSIN		Homo sapiens (Human)	448
C0JYY2	C0JYY2_HUMAN	unreviewed	Apolipoprotein B (Including Ag(X) antigen), isoform CRA_a	APOB hCG_20898	Homo sapiens (Human)	4563
A0A024R6N9	A0A024R6N9_HUMAN	unreviewed	Serpin peptidase inhibitor, clade A (Alpha-1 antiproteinase, antitrypsin), member 5, isoform CRA_a	SERPINA5 hCG_2029091	Homo sapiens (Human)	406
A2RTY6	A2RTY6_HUMAN	unreviewed	Inter-alpha (Globulin) inhibitor H2 (Inter-alpha (Globulin) inhibitor H2, isoform CRA_b) (cDNA FLJ75038, highly similar to Homo sapiens	ITIH2 hCG_25115	Homo sapiens (Human)	946
D6R934	D6R934_HUMAN	unreviewed	Complement C1q subcomponent subunit B	C1QB	Homo sapiens (Human)	251
A0A0G2JPR0	A0A0G2JPR0_HUMAN	unreviewed	Complement C4-A	C4A	Homo sapiens (Human)	1744
G3V2W1	G3V2W1_HUMAN	unreviewed	Protein Z-dependent protease inhibitor (Serpin peptidase inhibitor, clade A (Alpha-1 antiproteinase, antitrypsin), member 10, isoform CRA_	SERPINA10 hCG_22331	Homo sapiens (Human)	484
B2CIS9	B2CIS9_HUMAN	unreviewed	Caspase 14, apoptosis-related cysteine peptidase (Caspase-14) (cDNA, FLJ94644, Homo sapiens caspase 14, apoptosis-related cysteine pr	CASP14 hCG_38589	Homo sapiens (Human)	242
S4R3Y4	S4R3Y4_HUMAN	unreviewed	Protein AMBP	AMBP	Homo sapiens (Human)	238
B1AP58	B1AP58_HUMAN	unreviewed	Carboxypeptidase N catalytic chain (Fragment)	CPN1	Homo sapiens (Human)	204
A0A024RAA7	A0A024RAA7_HUMAN	unreviewed	Adiponectin B (Complement component 1, q subcomponent, C chain, isoform CRA_a)	C1QC ADIB hCG_2037016	Homo sapiens (Human)	245
I3L145	I3L145_HUMAN	unreviewed	Sex hormone-binding globulin (Sex hormone-binding globulin, isoform CRA_a)	SHBG hCG_42018	Homo sapiens (Human)	344
D3DPG0	D3DPG0_HUMAN	unreviewed	Titin, isoform CRA_a	TTN hCG_2006859	Homo sapiens (Human)	34942
B2RAN2	B2RAN2_HUMAN	unreviewed	cDNA, FLJ95014, highly similar to Homo sapiens vanin 1 (VNN1), mRNA		Homo sapiens (Human)	513
E9PHK0	E9PHK0_HUMAN	unreviewed	Tetranectin	CLEC3B	Homo sapiens (Human)	160
A8K1K1	A8K1K1_HUMAN	unreviewed	cDNA FLJ76342, highly similar to Homo sapiens carnosine dipeptidase 1 (metallopeptidase M20 family)(CNDP1), mRNA		Homo sapiens (Human)	507
A0A140VJI7	A0A140VJI7_HUMAN	unreviewed	Testicular tissue protein Li 61		Homo sapiens (Human)	540
Q59EP2	Q59EP2_HUMAN	unreviewed	Angiotensinogen variant (Fragment)		Homo sapiens (Human)	491
S4R471	S4R471_HUMAN	unreviewed	Protein AMBP (Fragment)	AMBP	Homo sapiens (Human)	193
J3K000	J3K000_HUMAN	unreviewed	PEPD protein	PEPD	Homo sapiens (Human)	493
H0YAC1	H0YAC1_HUMAN	unreviewed	Plasma kallikrein (Fragment)	KLKB1	Homo sapiens (Human)	686
Q5J875	Q5J875_HUMAN	unreviewed	GUGU gamma form	GUGU	Homo sapiens (Human)	317
A0A140VK00	A0A140VK00_HUMAN	unreviewed	Testicular tissue protein Li 227		Homo sapiens (Human)	298
A0A2Z4LCH4	A0A2Z4LCH4_HUMAN	unreviewed	Corticosteroid-binding globulin		Homo sapiens (Human)	405
Q53F26	Q53F26_HUMAN	unreviewed	Alpha-amylase (EC 3.2.1.1) (Fragment)		Homo sapiens (Human)	511
B7Z549	B7Z549_HUMAN	unreviewed	cDNA FLJ56821, highly similar to Inter-alpha-trypsin inhibitor heavy chain H1		Homo sapiens (Human)	677
X6RJP6	X6RJP6_HUMAN	unreviewed	Transgelin-2 (Fragment)	TAGLN2	Homo sapiens (Human)	187
B4DVE1	B4DVE1_HUMAN	unreviewed	cDNA FLJ53478, highly similar to Galectin-3-binding protein		Homo sapiens (Human)	573
B4DZ36	B4DZ36_HUMAN	unreviewed	cDNA FLJ58441, highly similar to Attractin		Homo sapiens (Human)	1156
B7ZKJ8	B7ZKJ8_HUMAN	unreviewed	ITIH4 protein (Inter-alpha-trypsin inhibitor heavy chain H4)	ITIH4	Homo sapiens (Human)	935
Q9UNL6	Q9UNL6_HUMAN	unreviewed	Hemoglobin gamma-G (Fragment)	HBG2	Homo sapiens (Human)	129
Q5JVE7	Q5JVE7_HUMAN	unreviewed	Coagulation factor X (Coagulation factor X, isoform CRA_a) (cDNA, FLJ94415, Homo sapiens coagulation factor X (F10), mRNA)	F10 hCG_27879	Homo sapiens (Human)	488
L8E853	L8E853_HUMAN	unreviewed	von Willebrand factor	VWF	Homo sapiens (Human)	2715
A0A024R035	A0A024R035_HUMAN	unreviewed	Complement component 9, isoform CRA_a	C9 hCG_37544	Homo sapiens (Human)	559
F8W0J6	F8W0J6_HUMAN	unreviewed	Nucleosome assembly protein 1-like 1	NAP1L1	Homo sapiens (Human)	379
J3KPA1	J3KPA1_HUMAN	unreviewed	Cysteine-rich secretory protein 3	CRISP3	Homo sapiens (Human)	276
Q53GK6	Q53GK6_HUMAN	unreviewed	Beta actin variant (Fragment)		Homo sapiens (Human)	375
V9HW68	V9HW68_HUMAN	unreviewed	Epididymis luminal protein 214	HEL-214	Homo sapiens (Human)	470
A0A087X1J7	A0A087X1J7_HUMAN	unreviewed	Glutathione peroxidase	GPX3	Homo sapiens (Human)	225
A0A024R1X6	A0A024R1X6_HUMAN	unreviewed	Keratin 14 (Epidermolysis bullosa simplex, Dowling-Meara, Koebner), isoform CRA_a	KRT14 hCG_2005483	Homo sapiens (Human)	261
Q4LE79	Q4LE79_HUMAN	unreviewed	DSP variant protein (Fragment)	DSP variant protein	Homo sapiens (Human)	2319
Q53H26	Q53H26_HUMAN	unreviewed	Transferrin variant (Fragment)		Homo sapiens (Human)	698
A0A2S0BDD1	A0A2S0BDD1_HUMAN	unreviewed	Antithrombin III isoform	ATIII-R2	Homo sapiens (Human)	505
Q13784	Q13784_HUMAN	unreviewed	APOA4 protein (Fragment)	APOA4	Homo sapiens (Human)	244
F5H1C6	F5H1C6_HUMAN	unreviewed	Fermitin family homolog 3 (Fragment)	FERMT3	Homo sapiens (Human)	286
C9JV77	C9JV77_HUMAN	unreviewed	Alpha-2-HS-glycoprotein	AHSG	Homo sapiens (Human)	368
Q9UEU7	Q9UEU7_HUMAN	unreviewed	Amine oxidase (EC 1.4.3.-) (Fragment)		Homo sapiens (Human)	64
I3L3D5	I3L3D5_HUMAN	unreviewed	Profilin (Fragment)	PFN1	Homo sapiens (Human)	165
B0YIW2	B0YIW2_HUMAN	unreviewed	Apolipoprotein C-III (Apolipoprotein C-III variant 1)	APOC3	Homo sapiens (Human)	117
D9IWP9	D9IWP9_HUMAN	unreviewed	Beta-2-glycoprotein I (Fragment)		Homo sapiens (Human)	326
E7END6	E7END6_HUMAN	unreviewed	Vitamin K-dependent protein C	PROC	Homo sapiens (Human)	495
J3QR68	J3QR68_HUMAN	unreviewed	Haptoglobin (Fragment)	HP	Homo sapiens (Human)	404
Q6MZL2	Q6MZL2_HUMAN	unreviewed	Uncharacterized protein DKFZp686M0562 (Fragment)	DKFZp686M0562	Homo sapiens (Human)	321
A8K8Z4	A8K8Z4_HUMAN	unreviewed	cDNA FLJ78071, highly similar to Human MHC class III complement component C6 mRNA		Homo sapiens (Human)	934
H0YID6	H0YID6_HUMAN	unreviewed	Keratin, type II cytoskeletal 79 (Fragment)	KRT79	Homo sapiens (Human)	119
A0N071	A0N071_HUMAN	unreviewed	Delta globin (Delta-globin chain) (Globin A2) (Hemoglobin delta) (Hemoglobin, delta)	HBD GLNA2 hCG_1641001	Homo sapiens (Human)	147
Q9BS19	Q9BS19_HUMAN	unreviewed	Epididymis secretory sperm binding protein (HPX protein) (Hemopexin)	HPX	Homo sapiens (Human)	254
Q53GN8	Q53GN8_HUMAN	unreviewed	Macrophage stimulating 1 (Hepatocyte growth factor-like) variant (Fragment)		Homo sapiens (Human)	711
Q6LAN8	Q6LAN8_HUMAN	unreviewed	Collagen type I alpha 1 (Fragment)	COL1A1	Homo sapiens (Human)	1069
B4DPQ0	B4DPQ0_HUMAN	unreviewed	Complement C1r subcomponent (cDNA FLJ54471, highly similar to Complement C1r subcomponent)	C1R	Homo sapiens (Human)	719
Q05CV2	Q05CV2_HUMAN	unreviewed	C8A protein (Fragment)	C8A	Homo sapiens (Human)	394
E7EQ64	E7EQ64_HUMAN	unreviewed	Trypsin-1	PRSS1	Homo sapiens (Human)	261
A0A3G1HEF1	A0A3G1HEF1_HUMAN	unreviewed	Mutant serpin family A member 7		Homo sapiens (Human)	415
V9HWF6	V9HWF6_HUMAN	unreviewed	Alpha-1-acid glycoprotein	HEL-S-153w	Homo sapiens (Human)	201
Q8TAY0	Q8TAY0_HUMAN	unreviewed	Insulin-like growth factor binding protein, acid labile subunit	IGFALS	Homo sapiens (Human)	605
B2R773	B2R773_HUMAN	unreviewed	cDNA, FLJ93312, highly similar to Homo sapiens adipose most abundant gene transcript 1 (APM1), mRNA		Homo sapiens (Human)	244
E9KL23	E9KL23_HUMAN	unreviewed	Epididymis secretory sperm binding protein Li 44a (Serpin peptidase inhibitor clade A member 1 isoform 1)	SERPINA1	Homo sapiens (Human)	418
B3KNX0	B3KNX0_HUMAN	unreviewed	cDNA FLJ30621 fis, clone CTONG2001681, highly similar to Complement C1s subcomponent		Homo sapiens (Human)	688
D3DNN4	D3DNN4_HUMAN	unreviewed	Carboxylic ester hydrolase (EC 3.1.1.-)	BCHE hCG_14822	Homo sapiens (Human)	643
F2RM35	F2RM35_HUMAN	unreviewed	Serine protease	factor IX F9	Homo sapiens (Human)	461
B4DPH4	B4DPH4_HUMAN	unreviewed	cDNA FLJ58778, highly similar to Plasminogen		Homo sapiens (Human)	407
F8W7L3	F8W7L3_HUMAN	unreviewed	Alpha-2-macroglobulin (Fragment)	A2M	Homo sapiens (Human)	168
B2R888	B2R888_HUMAN	unreviewed	Monocyte differentiation antigen CD14 (Myeloid cell-specific leucine-rich glycoprotein)		Homo sapiens (Human)	375
A0A024R104	A0A024R104_HUMAN	unreviewed	Contactin 1, isoform CRA_a	CNTN1 hCG_38261	Homo sapiens (Human)	1018
B2R815	B2R815_HUMAN	unreviewed	cDNA, FLJ93695, highly similar to Homo sapiens serpin peptidase inhibitor, clade A (alpha-1 antiproteinase, antitrypsin), member 4 (SERPINA4), mRNA		Homo sapiens (Human)	427
F8VV57	F8VV57_HUMAN	unreviewed	Keratin, type II cytoskeletal 5 (Fragment)	KRT5	Homo sapiens (Human)	132
A0A087WSY5	A0A087WSY5_HUMAN	unreviewed	Carboxypeptidase B2	CPB2	Homo sapiens (Human)	386
B7Z8Q5	B7Z8Q5_HUMAN	unreviewed	cDNA FLJ56762, highly similar to Hyaluronan-binding protein 2		Homo sapiens (Human)	280
Q59GS8	Q59GS8_HUMAN	unreviewed	Complement component 5 variant (Fragment)		Homo sapiens (Human)	1106
A0A3B3IS66	A0A3B3IS66_HUMAN	unreviewed	Coagulation factor XIII B chain (Fragment)	F13B	Homo sapiens (Human)	246
Q59EA3	Q59EA3_HUMAN	unreviewed	Cadherin 5, type 2 preproprotein variant (Fragment)		Homo sapiens (Human)	807
B3KT06	B3KT06_HUMAN	unreviewed	Tubulin alpha chain		Homo sapiens (Human)	416
A0A0S2Z3E8	A0A0S2Z3E8_HUMAN	unreviewed	Fibrinogen alpha chain isoform 2 (Fragment)	FGA	Homo sapiens (Human)	436
B2RA39	B2RA39_HUMAN	unreviewed	cDNA, FLJ94686, highly similar to Homo sapiens complement factor H-related 5 (CFHL5), mRNA		Homo sapiens (Human)	569
D9YZU5	D9YZU5_HUMAN	unreviewed	Beta-globin (Globin A1) (Hemoglobin subunit beta) (Hemoglobin, beta)	HBB GLNA1	Homo sapiens (Human)	147
B4DDT3	B4DDT3_HUMAN	unreviewed	cDNA FLJ54622, highly similar to Prothrombin		Homo sapiens (Human)	471
B7ZLE5	B7ZLE5_HUMAN	unreviewed	FN1 protein	FN1	Homo sapiens (Human)	2240
B4E1Z4	B4E1Z4_HUMAN	unreviewed	cDNA FLJ55673, highly similar to Complement factor B		Homo sapiens (Human)	1266
V9HWD8	V9HWD8_HUMAN	unreviewed	Epididymis secretory sperm binding protein Li 163pA	HEL-S-163pA	Homo sapiens (Human)	495
Q68CK4	Q68CK4_HUMAN	unreviewed	Leucine-rich alpha-2-glycoprotein (Leucine-rich alpha-2-glycoprotein 1) (cDNA FLJ14476 fis, clone MAMMA1001388, highly similar to Leucine-rich alpha-2-glycoprotein) (cDNA, FLJ95324, highly similar to Homo sapiens leucine-rich alpha-2-glycoprotein 1 (LRG1), mRNA)	HMFT1766 LRG1 hCG_2004087	Homo sapiens (Human)	347
K7EQQ3	K7EQQ3_HUMAN	unreviewed	Keratin, type I cytoskeletal 9	KRT9	Homo sapiens (Human)	390
A0A087WW43	A0A087WW43_HUMAN	unreviewed	Inter-alpha-trypsin inhibitor heavy chain H3	ITIH3	Homo sapiens (Human)	670
A0A024R962	A0A024R962_HUMAN	unreviewed	HCG40889, isoform CRA_b	hCG_40889	Homo sapiens (Human)	1231
A0A182DWH7	A0A182DWH7_HUMAN	unreviewed	Selenoprotein P (Fragment)	SELENOP	Homo sapiens (Human)	310
B2R950	B2R950_HUMAN	unreviewed	cDNA, FLJ94213, highly similar to Homo sapiens pregnancy-zone protein (PZP), mRNA		Homo sapiens (Human)	1482
F5GXS0	F5GXS0_HUMAN	unreviewed	Complement C4-B	C4B	Homo sapiens (Human)	1698
K7ERE3	K7ERE3_HUMAN	unreviewed	Keratin, type I cytoskeletal 13	KRT13	Homo sapiens (Human)	415
A0A1K0GXZ1	A0A1K0GXZ1_HUMAN	unreviewed	Globin C1 (Globin C2)	GLNC1 GLNC2	Homo sapiens (Human)	177
Q499G5	Q499G5_HUMAN	unreviewed	E2F3 protein	E2F3	Homo sapiens (Human)	126
B2R6W1	B2R6W1_HUMAN	unreviewed	cDNA, FLJ93143, highly similar to Homo sapiens complement component 7 (C7), mRNA		Homo sapiens (Human)	843
A0A384NKS6	A0A384NKS6_HUMAN	unreviewed	Clusterin		Homo sapiens (Human)	501
Q59EZ3	Q59EZ3_HUMAN	unreviewed	Insulin-like growth factor 2 receptor variant (Fragment)		Homo sapiens (Human)	2414
A0A3B3ISJ1	A0A3B3ISJ1_HUMAN	unreviewed	Vitamin K-dependent protein S	PROS1	Homo sapiens (Human)	662
A0A384N669	A0A384N669_HUMAN	unreviewed	Epididymis secretory sperm binding protein		Homo sapiens (Human)	338
V9GYM3	V9GYM3_HUMAN	unreviewed	Apolipoprotein A-II	APOA2	Homo sapiens (Human)	133
A6XND1	A6XND1_HUMAN	unreviewed	Insulin-like growth factor binding protein 3 isoform b (Insulin-like growth factor-binding protein 3)	IGFBP3	Homo sapiens (Human)	263

Supplementary Table 2. Database match (Macaca mulatta)

Entry	Entry name	Status	Protein names	Gene names	Organism	Length
Q5NKV6	ICAM1_MACMU	reviewed	Intercellular adhesion molecule 1 (ICAM-1) (CD antigen CD54)	ICAM1	Macaca mulatta (Rhesus macaque)	532
I2CYL7	I2CYL7_MACMU	unreviewed	Apolipoprotein E	APOE	Macaca mulatta (Rhesus macaque)	317
F6WIY2	F6WIY2_MACMU	unreviewed	Uncharacterized protein	THBS1	Macaca mulatta (Rhesus macaque)	1173
A0A1D5R9B8	A0A1D5R9B8_MACMU	unreviewed	Angiotensin-converting enzyme (EC 3.4.-.-)	ACE	Macaca mulatta (Rhesus macaque)	1307
F7HP21	F7HP21_MACMU	unreviewed	Uncharacterized protein	GSN	Macaca mulatta (Rhesus macaque)	784
O19092	CYTC_MACMU	reviewed	Cystatin-C (Cystatin-3)	CST3	Macaca mulatta (Rhesus macaque)	146
F6ZZL9	F6ZZL9_MACMU	unreviewed	Uncharacterized protein	HRG	Macaca mulatta (Rhesus macaque)	525
F6YW79	F6YW79_MACMU	unreviewed	Uncharacterized protein	C3	Macaca mulatta (Rhesus macaque)	1663
F7DHR8	F7DHR8_MACMU	unreviewed	Transferrin	TF	Macaca mulatta (Rhesus macaque)	698
F6UZ60	F6UZ60_MACMU	unreviewed	Fibrinogen alpha chain	FGA	Macaca mulatta (Rhesus macaque)	876
F7BTD3	F7BTD3_MACMU	unreviewed	SERPIN domain-containing protein	SERPINF2	Macaca mulatta (Rhesus macaque)	555
F7H2A5	F7H2A5_MACMU	unreviewed	Profilin	PFN1	Macaca mulatta (Rhesus macaque)	140
F6PLV7	F6PLV7_MACMU	unreviewed	Fibulin-1	FBLN1	Macaca mulatta (Rhesus macaque)	661
F7DX07	F7DX07_MACMU	unreviewed	Uncharacterized protein	CDH5	Macaca mulatta (Rhesus macaque)	785
F7C9A4	F7C9A4_MACMU	unreviewed	Hemopexin	HPX	Macaca mulatta (Rhesus macaque)	462
A0A1D5Q3Z5	A0A1D5Q3Z5_MACMU	unreviewed	Uncharacterized protein	CDH1	Macaca mulatta (Rhesus macaque)	882
F7DHQ1	F7DHQ1_MACMU	unreviewed	Pentaxin (Pentraxin)	CRP EGK_01483	Macaca mulatta (Rhesus macaque)	224
F7H5Q6	F7H5Q6_MACMU	unreviewed	Zn_dep_PLPC domain-containing protein	GPLD1	Macaca mulatta (Rhesus macaque)	858
A0N064	A0N064_MACMU	unreviewed	Prothrombin (EC 3.4.21.5) (Coagulation factor II)	F2	Macaca mulatta (Rhesus macaque)	627
F7FY72	F7FY72_MACMU	unreviewed	Uncharacterized protein	F12	Macaca mulatta (Rhesus macaque)	615
F7H1V9	F7H1V9_MACMU	unreviewed	Pentaxin (Pentraxin)	APCS	Macaca mulatta (Rhesus macaque)	223
F7HSL6	F7HSL6_MACMU	unreviewed	Uncharacterized protein	VCL	Macaca mulatta (Rhesus macaque)	1134
F6UM69	F6UM69_MACMU	unreviewed	Uncharacterized protein	APOH	Macaca mulatta (Rhesus macaque)	336
H9YUD6	H9YUD6_MACMU	unreviewed	L-selectin	SELL	Macaca mulatta (Rhesus macaque)	385
F6WSG1	F6WSG1_MACMU	unreviewed	Uncharacterized protein	CETP	Macaca mulatta (Rhesus macaque)	519
F7H9D9	F7H9D9_MACMU	unreviewed	Superoxide dismutase [Cu-Zn] (EC 1.15.1.1)	SOD3	Macaca mulatta (Rhesus macaque)	240
F6U5Q9	F6U5Q9_MACMU	unreviewed	MACPF domain-containing protein	C8A	Macaca mulatta (Rhesus macaque)	584
A0A1D5QYD0	A0A1D5QYD0_MACMU	unreviewed	Uncharacterized protein	ITIH4	Macaca mulatta (Rhesus macaque)	936
F7GRY2	F7GRY2_MACMU	unreviewed	MACPF domain-containing protein	C9	Macaca mulatta (Rhesus macaque)	561
A0A1D5Q7U7	A0A1D5Q7U7_MACMU	unreviewed	Uncharacterized protein	KLKB1	Macaca mulatta (Rhesus macaque)	638
F7F6V9	F7F6V9_MACMU	unreviewed	SERPIN domain-containing protein	SERPINA6	Macaca mulatta (Rhesus macaque)	404
F6U2J6	F6U2J6_MACMU	unreviewed	Uncharacterized protein	IGFBP3	Macaca mulatta (Rhesus macaque)	297
A0A1D5QS06	A0A1D5QS06_MACMU	unreviewed	Lipocln_cytosolic_FA-bd_dom domain-containing protein	C8G	Macaca mulatta (Rhesus macaque)	202
H9FXZ3	H9FXZ3_MACMU	unreviewed	Carboxylic ester hydrolase (EC 3.1.1.-)	BCHE	Macaca mulatta (Rhesus macaque)	602
F6WR34	F6WR34_MACMU	unreviewed	Uncharacterized protein	GC EGK_15808	Macaca mulatta (Rhesus macaque)	490
F6SL92	F6SL92_MACMU	unreviewed	SERPIN domain-containing protein	SERPINA3 EGK_18506	Macaca mulatta (Rhesus macaque)	448
F7F8G8	F7F8G8_MACMU	unreviewed	Protein S100 (S100 calcium-binding protein)	S100A9 EGK_01322	Macaca mulatta (Rhesus macaque)	114
F7B786	F7B786_MACMU	unreviewed	Cytokeratin-1	KRT1 EGK_03685	Macaca mulatta (Rhesus macaque)	636
F7EL64	F7EL64_MACMU	unreviewed	Chitinase-3-like protein 1	CHI3L1	Macaca mulatta (Rhesus macaque)	383
F7AV81	F7AV81_MACMU	unreviewed	Globin A1 (Hemoglobin beta chain) (Hemoglobin subunit beta)	HBB GLNA1 EGK_06585	Macaca mulatta (Rhesus macaque)	147
F6XAC5	F6XAC5_MACMU	unreviewed	Uncharacterized protein	CFHR5	Macaca mulatta (Rhesus macaque)	577
F7H7G9	F7H7G9_MACMU	unreviewed	Lipocln_cytosolic_FA-bd_dom domain-containing protein	ORM1	Macaca mulatta (Rhesus macaque)	214
F6X848	F6X848_MACMU	unreviewed	Uncharacterized protein	ATRN	Macaca mulatta (Rhesus macaque)	1383
F7EVX1	F7EVX1_MACMU	unreviewed	M20_dimer domain-containing protein	CNDP1	Macaca mulatta (Rhesus macaque)	506
A0A1D5QJP4	A0A1D5QJP4_MACMU	unreviewed	Uncharacterized protein	F5	Macaca mulatta (Rhesus macaque)	2181
F6W3M2	F6W3M2_MACMU	unreviewed	Immunoglobulin heavy constant mu	IGHM	Macaca mulatta (Rhesus macaque)	510
F6RKI4	F6RKI4_MACMU	unreviewed	Transgelin	TAGLN2	Macaca mulatta (Rhesus macaque)	226
F7DNJ5	F7DNJ5_MACMU	unreviewed	Uncharacterized protein	C1R	Macaca mulatta (Rhesus macaque)	719
G7NLH4	G7NLH4_MACMU	unreviewed	Flavin reductase (Flavin reductase (NADPH))	BLVRB EGK_10628	Macaca mulatta (Rhesus macaque)	206
F6Z7P7	F6Z7P7_MACMU	unreviewed	LAM_G_DOMAIN domain-containing protein	SHBG	Macaca mulatta (Rhesus macaque)	449
H9H2V9	H9H2V9_MACMU	unreviewed	Uncharacterized protein		Macaca mulatta (Rhesus macaque)	402
A0A1D5QSH6	A0A1D5QSH6_MACMU	unreviewed	Vitamin K-dependent protein C	PROC	Macaca mulatta (Rhesus macaque)	483
F7CNK6	F7CNK6_MACMU	unreviewed	SRCR domain-containing protein	LGALS3BP	Macaca mulatta (Rhesus macaque)	633
F7HL22	F7HL22_MACMU	unreviewed	Uncharacterized protein	TLN1	Macaca mulatta (Rhesus macaque)	2538
F6SCP6	F6SCP6_MACMU	unreviewed	C-X-C motif chemokine	PPBP EGK_15792	Macaca mulatta (Rhesus macaque)	128
F7FHW4	F7FHW4_MACMU	unreviewed	Uncharacterized protein	FN1	Macaca mulatta (Rhesus macaque)	2265
F7HF51	F7HF51_MACMU	unreviewed	Uncharacterized protein	NAGLU	Macaca mulatta (Rhesus macaque)	645
A0A1D5Q2R8	A0A1D5Q2R8_MACMU	unreviewed	Uncharacterized protein	LOC721200	Macaca mulatta (Rhesus macaque)	1734
F7HFD0	F7HFD0_MACMU	unreviewed	Fibrinogen C-terminal domain-containing protein	FCN3	Macaca mulatta (Rhesus macaque)	288
F6XIG8	F6XIG8_MACMU	unreviewed	Heparin cofactor 2	SERPIND1 EGK_02823	Macaca mulatta (Rhesus macaque)	499
A0A1D5Q4R2	A0A1D5Q4R2_MACMU	unreviewed	C-X-C motif chemokine	PF4	Macaca mulatta (Rhesus macaque)	101
F6Z0R5	F6Z0R5_MACMU	unreviewed	Uncharacterized protein	HP	Macaca mulatta (Rhesus macaque)	361
F7BCK9	F7BCK9_MACMU	unreviewed	Lysosome-associated membrane glycoprotein 2 isoform B	LAMP2	Macaca mulatta (Rhesus macaque)	410
F6VKN1	F6VKN1_MACMU	unreviewed	Aminopeptidase (EC 3.4.11.-)	ANPEP	Macaca mulatta (Rhesus macaque)	968
F7H312	F7H312_MACMU	unreviewed	IF rod domain-containing protein	KRT14	Macaca mulatta (Rhesus macaque)	475
A0A1D5R122	A0A1D5R122_MACMU	unreviewed	C-type lectin domain-containing protein	MBL2	Macaca mulatta (Rhesus macaque)	219
A0A1D5R0L5	A0A1D5R0L5_MACMU	unreviewed	Uncharacterized protein	VWF	Macaca mulatta (Rhesus macaque)	2824
F7HCH2	F7HCH2_MACMU	unreviewed	Serum albumin	ALB	Macaca mulatta (Rhesus macaque)	588
F6WG91	F6WG91_MACMU	unreviewed	Vitamin K-dependent protein S	PROS1	Macaca mulatta (Rhesus macaque)	676
A0A1D5QCC0	A0A1D5QCC0_MACMU	unreviewed	Uncharacterized protein	LOC714514	Macaca mulatta (Rhesus macaque)	1658
F6RNZ2	F6RNZ2_MACMU	unreviewed	Uncharacterized protein	C4BPA	Macaca mulatta (Rhesus macaque)	598
A0A1D5QW71	A0A1D5QW71_MACMU	unreviewed	Uncharacterized protein	FETUB	Macaca mulatta (Rhesus macaque)	382
F7ALN6	F7ALN6_MACMU	unreviewed	Uncharacterized protein	C7	Macaca mulatta (Rhesus macaque)	843
F7EI47	F7EI47_MACMU	unreviewed	Uncharacterized protein	C1S	Macaca mulatta (Rhesus macaque)	679
F7H263	F7H263_MACMU	unreviewed	C-type lectin domain-containing protein	MBP-A	Macaca mulatta (Rhesus macaque)	249
F6TIK4	F6TIK4_MACMU	unreviewed	Uncharacterized protein	ACTB	Macaca mulatta (Rhesus macaque)	375
F6WYB6	F6WYB6_MACMU	unreviewed	SERPIN domain-containing protein	SERPINA5	Macaca mulatta (Rhesus macaque)	172
F7B749	F7B749_MACMU	unreviewed	Uncharacterized protein	CPN2	Macaca mulatta (Rhesus macaque)	545
G7MVP0	G7MVP0_MACMU	unreviewed	SPARC	SPARC EGK_17045	Macaca mulatta (Rhesus macaque)	303
F6W003	F6W003_MACMU	unreviewed	Insulin-like growth factor 1 isoform 4 preproprotein	IGF1	Macaca mulatta (Rhesus macaque)	153
F7DD08	F7DD08_MACMU	unreviewed	Uncharacterized protein	ITIH2	Macaca mulatta (Rhesus macaque)	946
B3Y6B8	B3Y6B8_MACMU	unreviewed	Monocyte differentiation antigen CD14 (Myeloid cell-specific leucine-rich glycoprotein)	CD14	Macaca mulatta (Rhesus macaque)	375
F6T0W5	F6T0W5_MACMU	unreviewed	Tetranectin	CLEC3B	Macaca mulatta (Rhesus macaque)	202
F7DH19	F7DH19_MACMU	unreviewed	Uncharacterized protein	HGFAC	Macaca mulatta (Rhesus macaque)	626
F6QJD8	F6QJD8_MACMU	unreviewed	Protein S100 (S100 calcium-binding protein)	S100A8 EGK_21438	Macaca mulatta (Rhesus macaque)	93
A0A1D5QKX6	A0A1D5QKX6_MACMU	unreviewed	Vitellogenin domain-containing protein	APOB	Macaca mulatta (Rhesus macaque)	4555
F6UR30	F6UR30_MACMU	unreviewed	IF rod domain-containing protein	KRT9	Macaca mulatta (Rhesus macaque)	621
F7GHV1	F7GHV1_MACMU	unreviewed	Uncharacterized protein	C5	Macaca mulatta (Rhesus macaque)	1676
F7FJQ0	F7FJQ0_MACMU	unreviewed	Ig-like domain-containing protein	A1BG	Macaca mulatta (Rhesus macaque)	413
F6TP36	F6TP36_MACMU	unreviewed	MACPF domain-containing protein	C8B	Macaca mulatta (Rhesus macaque)	542
G7NQC3	G7NQC3_MACMU	unreviewed	Hemoglobin alpha chain (Hemoglobin subunit alpha)	HBA2 HBA EGK_12276	Macaca mulatta (Rhesus macaque)	142
F7HQV3	F7HQV3_MACMU	unreviewed	Peptidase_M14 domain-containing protein	CPB2	Macaca mulatta (Rhesus macaque)	386
F6UQI4	F6UQI4_MACMU	unreviewed	Uncharacterized protein	AMBP	Macaca mulatta (Rhesus macaque)	352
F6S885	F6S885_MACMU	unreviewed	Clusterin	CLU	Macaca mulatta (Rhesus macaque)	446
F7BQW1	F7BQW1_MACMU	unreviewed	Fibrinogen C-terminal domain-containing protein	LOC100427524	Macaca mulatta (Rhesus macaque)	312
F7HT52	F7HT52_MACMU	unreviewed	Uncharacterized protein	IL1R2	Macaca mulatta (Rhesus macaque)	395
F6YK65	F6YK65_MACMU	unreviewed	Uncharacterized protein	C2 EGK_14737	Macaca mulatta (Rhesus macaque)	752
F7GHR4	F7GHR4_MACMU	unreviewed	Uncharacterized protein	F9	Macaca mulatta (Rhesus macaque)	461
F7DIN9	F7DIN9_MACMU	unreviewed	SERPIN domain-containing protein	SERPINA7	Macaca mulatta (Rhesus macaque)	415
F7GAT4	F7GAT4_MACMU	unreviewed	Uncharacterized protein	ITIH3	Macaca mulatta (Rhesus macaque)	891
F6PRP0	F6PRP0_MACMU	unreviewed	Keratan sulfate proteoglycan lumican (Lumican)	LUM EGK_04048	Macaca mulatta (Rhesus macaque)	338
F7HEL0	F7HEL0_MACMU	unreviewed	Complement C1q subcomponent subunit C	C1QC EGK_00353	Macaca mulatta (Rhesus macaque)	245
F7F4G6	F7F4G6_MACMU	unreviewed	SERPIN domain-containing protein	SERPING1	Macaca mulatta (Rhesus macaque)	500
F6YJW2	F6YJW2_MACMU	unreviewed	Uncharacterized protein	C2	Macaca mulatta (Rhesus macaque)	1266
F6TLR3	F6TLR3_MACMU	unreviewed	SERPIN domain-containing protein	AGT	Macaca mulatta (Rhesus macaque)	485
F7DLB8	F7DLB8_MACMU	unreviewed	Uncharacterized protein	POSTN	Macaca mulatta (Rhesus macaque)	772
F7H532	F7H532_MACMU	unreviewed	Uncharacterized protein	ECM1 EGK_01229	Macaca mulatta (Rhesus macaque)	567
F6T094	F6T094_MACMU	unreviewed	Uncharacterized protein	LBP	Macaca mulatta (Rhesus macaque)	481
F6TNV9	F6TNV9_MACMU	unreviewed	Uncharacterized protein	A2M	Macaca mulatta (Rhesus macaque)	1474
A0A1D5Q6N1	A0A1D5Q6N1_MACMU	unreviewed	Uncharacterized protein	ITIH1	Macaca mulatta (Rhesus macaque)	911
H9H3P6	H9H3P6_MACMU	unreviewed	Uncharacterized protein	CFH	Macaca mulatta (Rhesus macaque)	987
F6Y7Q4	F6Y7Q4_MACMU	unreviewed	Uncharacterized protein	IGFALS	Macaca mulatta (Rhesus macaque)	652
F6PJA1	F6PJA1_MACMU	unreviewed	Uncharacterized protein	F11	Macaca mulatta (Rhesus macaque)	625
F7HQ03	F7HQ03_MACMU	unreviewed	C1q domain-containing protein	ADIPOQ	Macaca mulatta (Rhesus macaque)	249
F6YYK7	F6YYK7_MACMU	unreviewed	Uncharacterized protein	PGLYRP2	Macaca mulatta (Rhesus macaque)	624
A0A1D5QUU6	A0A1D5QUU6_MACMU	unreviewed	Uncharacterized protein	GPX3	Macaca mulatta (Rhesus macaque)	216
F7DNI9	F7DNI9_MACMU	unreviewed	Uncharacterized protein	C1RL	Macaca mulatta (Rhesus macaque)	485
F7FY55	F7FY55_MACMU	unreviewed	ShKT domain-containing protein	CRISP3	Macaca mulatta (Rhesus macaque)	275
A0A1D5QXZ0	A0A1D5QXZ0_MACMU	unreviewed	Transthyretin	TTR EGK_09613	Macaca mulatta (Rhesus macaque)	147
F6SEN6	F6SEN6_MACMU	unreviewed	Alpha-1-antitrypsin	SERPINA1 EGK_18500	Macaca mulatta (Rhesus macaque)	418
F7HG52	F7HG52_MACMU	unreviewed	Uncharacterized protein	C6	Macaca mulatta (Rhesus macaque)	934
F7HAH7	F7HAH7_MACMU	unreviewed	Uncharacterized protein	AFM	Macaca mulatta (Rhesus macaque)	599
H9FY19	H9FY19_MACMU	unreviewed	Retinol-binding protein	RBP4	Macaca mulatta (Rhesus macaque)	201
F7AEK9	F7AEK9_MACMU	unreviewed	Cytokeratin-2e	KRT2 EGK_03684	Macaca mulatta (Rhesus macaque)	641
F6SW75	F6SW75_MACMU	unreviewed	Mannan binding lectin serine peptidase 2	MASP2	Macaca mulatta (Rhesus macaque)	686
F7HR41	F7HR41_MACMU	unreviewed	Uncharacterized protein	MASP1	Macaca mulatta (Rhesus macaque)	723
F6VZ47	F6VZ47_MACMU	unreviewed	Uncharacterized protein	AHSG	Macaca mulatta (Rhesus macaque)	367
F6SG43	F6SG43_MACMU	unreviewed	Uncharacterized protein	KNG1 EGK_12191	Macaca mulatta (Rhesus macaque)	644
F6SB17	F6SB17_MACMU	unreviewed	Uncharacterized protein	PROZ	Macaca mulatta (Rhesus macaque)	422
F7HM21	F7HM21_MACMU	unreviewed	Coagulation factor X preproprotein	F10	Macaca mulatta (Rhesus macaque)	488
F7F6Y0	F7F6Y0_MACMU	unreviewed	SERPIN domain-containing protein	SERPINA10	Macaca mulatta (Rhesus macaque)	471
F7FQN1	F7FQN1_MACMU	unreviewed	Uncharacterized protein	APOC3	Macaca mulatta (Rhesus macaque)	117
F6Z0T5	F6Z0T5_MACMU	unreviewed	Peptidase S1 domain-containing protein	LOC709274	Macaca mulatta (Rhesus macaque)	340
F6YWS9	F6YWS9_MACMU	unreviewed	Ig-like domain-containing protein	AZGP1	Macaca mulatta (Rhesus macaque)	298
F7GFB4	F7GFB4_MACMU	unreviewed	Uncharacterized protein	CP	Macaca mulatta (Rhesus macaque)	1090
A0A1D5QUE8	A0A1D5QUE8_MACMU	unreviewed	Uncharacterized protein	CFI	Macaca mulatta (Rhesus macaque)	590
F7HCV8	F7HCV8_MACMU	unreviewed	LRRCT domain-containing protein	LRG1	Macaca mulatta (Rhesus macaque)	383
A0A1D5QD27	A0A1D5QD27_MACMU	unreviewed	Uncharacterized protein	LOC106996491	Macaca mulatta (Rhesus macaque)	225
F7CC91	F7CC91_MACMU	unreviewed	Plasminogen (EC 3.4.21.7)	PLG	Macaca mulatta (Rhesus macaque)	810
A0A1D5QLP0	A0A1D5QLP0_MACMU	unreviewed	Amine oxidase (EC 1.4.3.-)	LOC708053	Macaca mulatta (Rhesus macaque)	752
F6ZGR2	F6ZGR2_MACMU	unreviewed	SERPIN domain-containing protein	SERPINF1	Macaca mulatta (Rhesus macaque)	418
F6Y0N9	F6Y0N9_MACMU	unreviewed	SERPIN domain-containing protein	SERPINC1	Macaca mulatta (Rhesus macaque)	464
F6SYK0	F6SYK0_MACMU	unreviewed	Fructose-bisphosphate aldolase (EC 4.1.2.13)	ALDOA	Macaca mulatta (Rhesus macaque)	355
F6ZH99	F6ZH99_MACMU	unreviewed	Uncharacterized protein	MYH1	Macaca mulatta (Rhesus macaque)	1919
F7FPV8	F7FPV8_MACMU	unreviewed	Apolipoprotein(a)	LPA	Macaca mulatta (Rhesus macaque)	1636
A0A1D5QF45	A0A1D5QF45_MACMU	unreviewed	Uncharacterized protein	PZP	Macaca mulatta (Rhesus macaque)	1483
F7B5G7	F7B5G7_MACMU	unreviewed	IF rod domain-containing protein	KRT10	Macaca mulatta (Rhesus macaque)	566
F6SSW9	F6SSW9_MACMU	unreviewed	Uncharacterized protein	VTN	Macaca mulatta (Rhesus macaque)	448
F7B8S4	F7B8S4_MACMU	unreviewed	Uncharacterized protein	CHL1	Macaca mulatta (Rhesus macaque)	1160
F7DAC5	F7DAC5_MACMU	unreviewed	Uncharacterized protein	HABP2	Macaca mulatta (Rhesus macaque)	560
F7DXU0	F7DXU0_MACMU	unreviewed	Tubulin alpha chain	TUBA1B	Macaca mulatta (Rhesus macaque)	386
F7FQN6	F7FQN6_MACMU	unreviewed	Apolipoprotein A4	APOA4	Macaca mulatta (Rhesus macaque)	411

Supplementary Table 3. Difference between routes in each time IM vs. PO

	Pre			0.25 h			0.5 h			1 h			2 h		
**Name**	*p-value*	*FDR*[a]	*log2(FC)*	*p-value*	*FDR*	*log2(FC)*	*p-value*	*FDR*	*log2(FC)*	*p-value*	*FDR*	*log2(FC)*	*p-value*	*FDR*	*log2(FC)*
A0A0S2Z3D5	3.27E-03	NS	-2.06	2.22E-01	NS	0.66	8.58E-01	NS	-0.31	5.10E-01	NS	-0.31	4.26E-01	NS	1.20
E7EQ64	1.32E-02	NS	-1.14	5.70E-01	NS	0.25	7.65E-01	NS	-0.22	4.85E-01	NS	-0.63	6.05E-01	NS	0.39
Q5JVE7	1.42E-02	NS	-0.72	8.54E-02	NS	-0.22	6.03E-01	NS	0.11	1.90E-01	NS	-0.37	9.23E-01	NS	-0.11
B2R888	1.61E-02	NS	0.52	3.80E-01	NS	0.31	1.48E-01	NS	0.42	6.45E-01	NS	-0.47	1.60E-01	NS	-0.47
A0N071	2.28E-02	NS	2.23	5.92E-01	NS	0.03	2.56E-01	NS	1.99	1.53E-01	NS	2.25	6.97E-01	NS	0.26
B4DRY0	3.26E-02	NS	1.28	5.20E-01	NS	1.73	1.39E-01	NS	-3.98	7.86E-01	NS	-0.26	9.36E-01	NS	0.48
C9JEU5	4.27E-02	NS	1.70	3.28E-01	NS	-0.93	5.22E-01	NS	-0.51	7.01E-01	NS	-1.53	5.05E-01	NS	-0.55
V9HWN8	6.07E-02	NS	-1.16	5.84E-01	NS	-0.33	1.64E-02	NS	0.75	3.05E-01	NS	-0.38	9.04E-01	NS	0.06
H0YID6	6.76E-02	NS	-1.97	8.79E-01	NS	-0.09	4.06E-01	NS	1.43	3.28E-01	NS	-1.87	5.19E-01	NS	1.70
B7ZLE5	8.36E-02	NS	1.26	1.03E-01	NS	0.34	7.65E-01	NS	-0.06	3.91E-01	NS	-0.65	6.49E-01	NS	-0.06
B4DRT3	1.12E-01	NS	0.48	9.56E-01	NS	0.35	6.51E-01	NS	0.11	2.73E-01	NS	0.82	3.54E-01	NS	0.71
I3L145	1.18E-01	NS	0.91	5.16E-01	NS	0.41	9.20E-01	NS	-0.10	5.45E-01	NS	-0.19	2.66E-01	NS	-0.44
A0A3B3ISJ1	1.28E-01	NS	0.24	9.02E-01	NS	0.01	5.87E-01	NS	0.12	4.67E-01	NS	0.14	7.74E-01	NS	-0.04
A0A024R962	1.31E-01	NS	-1.10	9.30E-01	NS	0.35	3.19E-01	NS	-0.38	7.20E-01	NS	0.48	1.33E-01	NS	0.97
Q53H26	1.31E-01	NS	-0.76	6.73E-02	NS	1.27	1.22E-01	NS	-1.22	2.85E-01	NS	-1.16	6.58E-01	NS	-0.92
H3BRJ9	1.47E-01	NS	-1.35	5.06E-01	NS	0.15	1.19E-02	NS	2.00	5.52E-02	NS	-2.15	7.55E-01	NS	-0.47
V9HW68	1.56E-01	NS	-1.18	5.57E-01	NS	0.53	5.37E-01	NS	-0.94	1.06E-01	NS	-0.91	9.22E-01	NS	-0.49
Q68CK4	1.59E-01	NS	-0.21	7.62E-01	NS	0.02	9.80E-01	NS	0.08	1.69E-01	NS	-0.75	5.24E-01	NS	-0.17
B7ZKJ8	1.59E-01	NS	-0.40	8.73E-01	NS	0.11	9.41E-01	NS	0.02	8.81E-01	NS	-0.05	9.86E-01	NS	-0.03
Q53GK6	1.63E-01	NS	0.97	3.23E-01	NS	0.57	4.36E-02	NS	2.00	3.63E-02	NS	1.79	2.27E-01	NS	0.44
A0A024R1X6	1.68E-01	NS	1.20	6.23E-01	NS	0.75	1.05E-01	NS	-3.71	4.88E-01	NS	0.41	8.98E-01	NS	0.59
L8E853	2.02E-01	NS	-0.44	2.41E-01	NS	-0.44	3.29E-02	NS	0.72	9.35E-01	NS	-0.30	3.50E-01	NS	0.32
V9HVY1	2.10E-01	NS	0.87	6.50E-01	NS	-0.49	9.67E-01	NS	0.66	4.36E-01	NS	-0.54	4.85E-01	NS	-0.52
A0A3B3IS66	2.15E-01	NS	0.49	4.73E-03	NS	0.87	4.64E-02	NS	-0.32	9.81E-01	NS	-0.05	3.41E-01	NS	0.39
Q59E93	2.21E-01	NS	0.86	2.10E-01	NS	0.60	8.83E-01	NS	-0.03	2.66E-01	NS	-1.14	5.82E-01	NS	-0.14
Q5JP69	2.26E-01	NS	0.36	7.23E-01	NS	0.04	4.89E-01	NS	-0.20	8.05E-01	NS	0.18	6.15E-01	NS	-0.27
Q9BS19	2.27E-01	NS	-0.36	4.68E-01	NS	0.13	6.77E-01	NS	-0.11	2.62E-01	NS	-0.44	4.64E-01	NS	0.17
F8W7L3	2.29E-01	NS	-1.19	8.09E-01	NS	0.18	2.62E-01	NS	-1.20	1.49E-01	NS	-0.54	5.11E-01	NS	-0.69
A0A087WSY5	2.36E-01	NS	-0.39	2.88E-01	NS	-0.37	5.43E-01	NS	-0.10	8.62E-01	NS	0.00	5.48E-02	NS	-0.53
V9HW98	2.38E-01	NS	-0.55	1.81E-01	NS	0.62	1.95E-01	NS	-0.66	9.58E-01	NS	-0.32	4.72E-01	NS	-0.52
A0A2S0BDD1	2.49E-01	NS	-0.54	3.65E-01	NS	-0.42	4.01E-01	NS	0.30	1.43E-01	NS	-0.46	9.64E-01	NS	0.04
A0A2Z4LCH4	2.54E-01	NS	-0.85	4.24E-01	NS	-0.56	4.37E-01	NS	0.44	5.55E-01	NS	-0.37	3.07E-01	NS	-0.39
B2RAN2	2.59E-01	NS	0.31	3.87E-01	NS	0.49	9.91E-01	NS	0.00	5.24E-01	NS	0.22	6.54E-01	NS	-0.06
D9ZGG2	2.67E-01	NS	-0.23	5.78E-01	NS	-0.09	2.16E-02	NS	-0.35	7.29E-01	NS	-0.04	6.18E-01	NS	0.11
X6RJP6	2.67E-01	NS	0.79	1.08E-01	NS	1.29	5.97E-02	NS	1.33	2.45E-02	NS	1.33	2.50E-02	NS	1.97
A0A024RAA7	2.77E-01	NS	0.44	7.74E-01	NS	-0.05	9.21E-02	NS	0.33	2.15E-01	NS	0.42	9.35E-01	NS	0.02
V9HWE9	2.78E-01	NS	0.60	7.66E-02	NS	0.93	3.34E-02	NS	1.21	7.90E-01	NS	-1.02	9.69E-02	NS	1.57
Q59EP2	2.83E-01	NS	0.27	5.01E-01	NS	0.15	6.54E-03	NS	0.58	3.78E-01	NS	0.13	4.84E-01	NS	-0.15
A0A140VJI7	2.85E-01	NS	-0.48	6.58E-01	NS	0.07	2.44E-01	NS	-0.28	1.92E-01	NS	-0.59	4.11E-01	NS	0.24
A0A0G2JPR0	3.01E-01	NS	0.33	3.66E-01	NS	-0.18	1.02E-01	NS	0.42	1.01E-01	NS	0.16	2.60E-01	NS	-0.30
P78492	3.07E-01	NS	0.18	4.89E-02	NS	-0.39	9.55E-01	NS	0.02	1.37E-01	NS	0.34	7.75E-01	NS	0.03
A0A024R3E3	3.08E-01	NS	-1.72	2.72E-01	NS	1.11	6.31E-01	NS	-2.11	8.44E-01	NS	-0.64	7.58E-01	NS	-0.64
B2RA39	3.16E-01	NS	2.14	1.86E-05	NS	1.03	7.01E-02	NS	-1.01	7.28E-01	NS	-1.14	1.45E-01	NS	1.93
B2CIS9	3.16E-01	NS	0.42	2.09E-01	NS	-0.64	1.04E-01	NS	-0.64	3.91E-01	NS	-0.89	8.97E-01	NS	-0.22
E3UN46	3.35E-01	NS	0.69	4.86E-02	NS	0.55	4.78E-02	NS	-0.54	1.54E-01	NS	-0.55	2.99E-01	NS	0.51
S4R471	3.66E-01	NS	0.28	3.23E-01	NS	0.49	4.49E-01	NS	0.23	8.52E-01	NS	0.14	9.71E-02	NS	-0.86
F2RM35	3.75E-01	NS	-0.20	6.75E-01	NS	0.13	3.26E-01	NS	-0.43	6.45E-01	NS	-0.22	7.19E-01	NS	0.12
A0A384MDV8	3.78E-01	NS	0.17	5.06E-01	NS	0.15	3.05E-01	NS	-0.35	7.21E-01	NS	-0.08	5.58E-01	NS	-0.33
E9KL23	3.87E-01	NS	-0.49	2.04E-01	NS	1.14	5.19E-01	NS	1.33	4.51E-01	NS	-0.24	9.89E-01	NS	-0.25
Q96K68	3.93E-01	NS	-0.53	5.65E-01	NS	0.18	4.06E-01	NS	-0.63	1.62E-02	NS	-1.75	8.44E-01	NS	-0.21
B4DVE1	3.95E-01	NS	-0.47	2.21E-01	NS	-0.63	9.11E-01	NS	-0.02	9.15E-01	NS	-0.60	8.65E-01	NS	-0.08
B2R4M6	4.05E-01	NS	0.64	8.04E-02	NS	1.19	2.92E-01	NS	-0.75	9.71E-01	NS	-0.15	2.15E-01	NS	0.82
A5PL27	4.10E-01	NS	-0.74	6.26E-01	NS	-0.36	3.08E-02	NS	1.06	1.39E-01	NS	0.37	8.23E-01	NS	-0.81
B4E3P1	4.19E-01	NS	1.07	7.83E-02	NS	1.49	2.52E-01	NS	0.72	4.27E-03	NS	2.03	1.78E-01	NS	1.42
D3DNN4	4.22E-01	NS	-0.47	6.77E-02	NS	-1.06	5.81E-01	NS	0.87	5.78E-01	NS	-0.38	7.24E-01	NS	0.05
F8W876	4.27E-01	NS	-0.32	9.62E-01	NS	-0.07	5.47E-01	NS	0.20	6.49E-01	NS	-0.23	7.56E-01	NS	0.15
A0A024RAG6	4.38E-01	NS	0.40	5.25E-01	NS	-0.36	8.39E-01	NS	0.15	8.70E-01	NS	-0.06	5.34E-01	NS	-0.20
V9HWA9	4.62E-01	NS	0.36	1.66E-01	NS	-0.38	5.68E-02	NS	0.54	3.54E-01	NS	0.26	2.69E-01	NS	-0.39
C0JYY2	4.64E-01	NS	-0.22	2.78E-01	NS	-0.42	2.29E-01	NS	0.23	2.71E-01	NS	-0.42	6.50E-01	NS	-0.17
A0A0A0MRJ7	4.64E-01	NS	-0.19	2.56E-01	NS	0.34	3.36E-02	NS	-0.51	1.75E-01	NS	-0.50	8.58E-01	NS	0.03
Q8NBH6	4.73E-01	NS	0.22	8.72E-01	NS	0.08	4.84E-01	NS	0.33	5.99E-01	NS	-0.30	5.34E-01	NS	-0.37
Q05CV2	4.73E-01	NS	0.31	4.33E-01	NS	-0.24	1.67E-01	NS	0.50	7.58E-01	NS	0.21	8.84E-02	NS	-0.42
F5GXS0	4.77E-01	NS	0.33	4.02E-01	NS	-0.29	2.53E-02	NS	0.49	4.18E-01	NS	0.26	1.56E-01	NS	-0.52
Q6MZL2	4.77E-01	NS	0.14	9.85E-01	NS	0.00	8.51E-01	NS	-0.03	7.32E-01	NS	-0.58	6.01E-01	NS	0.14
A0A0J9YY65	4.79E-01	NS	-0.19	5.15E-01	NS	-0.25	3.90E-01	NS	0.26	7.27E-01	NS	-0.13	1.77E-01	NS	-0.42
B3KNX0	4.81E-01	NS	-0.26	2.00E-01	NS	0.66	3.76E-01	NS	0.64	8.72E-03	NS	-1.84	8.99E-01	NS	0.04
H6VRG1	4.92E-01	NS	0.44	5.13E-01	NS	-0.22	5.54E-01	NS	-0.55	1.85E-01	NS	-0.59	5.13E-01	NS	0.31
B7Z8Q5	5.00E-01	NS	0.55	1.37E-01	NS	1.25	2.36E-01	NS	-1.36	3.85E-01	NS	-0.20	5.84E-01	NS	0.09
B1AP58	5.00E-01	NS	0.35	5.98E-01	NS	-0.16	2.53E-01	NS	0.71	9.75E-02	NS	0.42	3.67E-02	NS	-1.00
B4DPH4	5.05E-01	NS	-0.15	3.83E-01	NS	-0.18	6.14E-01	NS	-0.08	8.62E-01	NS	0.13	4.99E-01	NS	-0.08
A0A140VKF3	5.09E-01	NS	0.40	5.55E-01	NS	-0.17	2.00E-02	NS	0.47	8.35E-01	NS	-0.21	5.79E-01	NS	-0.13
A0A024R9Q1	5.18E-01	NS	0.35	5.03E-01	NS	0.20	7.44E-01	NS	0.29	4.60E-01	NS	0.38	7.06E-01	NS	0.09
A0A0G2JPA8	5.29E-01	NS	0.16	9.80E-01	NS	0.01	4.43E-01	NS	0.16	3.01E-01	NS	-0.34	1.01E-01	NS	-0.33
H0YAC1	5.31E-01	NS	-0.13	4.19E-01	NS	0.21	9.68E-01	NS	-0.03	3.52E-01	NS	-0.21	1.35E-01	NS	0.39
Q59GS8	5.43E-01	NS	0.20	1.07E-01	NS	-0.53	2.59E-01	NS	0.21	6.87E-01	NS	0.14	2.42E-01	NS	-0.33
B3KT06	5.44E-01	NS	0.04	1.86E-01	NS	0.77	6.19E-01	NS	-1.53	2.73E-01	NS	0.43	7.47E-01	NS	0.83
J3K000	5.49E-01	NS	0.36	5.94E-01	NS	-0.32	1.38E-01	NS	0.73	9.91E-01	NS	#N/A	5.54E-01	NS	-0.20
D9YZU5	5.59E-01	NS	1.91	8.83E-01	NS	-1.43	5.31E-02	NS	3.12	2.88E-02	NS	2.38	7.63E-01	NS	-0.25
K7ERE3	5.70E-01	NS	0.07	8.62E-01	NS	-0.11	9.75E-01	NS	-2.75	4.19E-01	NS	-0.90	1.77E-01	NS	1.20
G3V2W1	5.78E-01	NS	0.19	5.04E-01	NS	-0.19	7.25E-01	NS	0.09	4.95E-01	NS	0.28	8.40E-01	NS	-0.32
Q9H2E1	5.85E-01	NS	-0.03	2.51E-01	NS	2.49	7.46E-01	NS	-0.49	7.87E-01	NS	-0.07	2.85E-01	NS	0.38
A0A1B0GUU9	6.06E-01	NS	0.47	3.59E-01	NS	-0.29	2.86E-01	NS	0.52	4.54E-01	NS	-0.49	2.80E-01	NS	-0.59
D0PNI1	6.16E-01	NS	0.86	3.19E-02	NS	2.29	4.52E-01	NS	0.50	3.66E-02	NS	1.45	6.54E-02	NS	1.24
Q59EZ3	6.19E-01	NS	0.01	3.48E-01	NS	0.84	8.77E-03	NS	2.28	6.69E-01	NS	-0.98	9.91E-01	NS	-0.12
Q6LAN8	6.35E-01	NS	0.22	5.06E-01	NS	0.57	8.96E-01	NS	-0.18	4.88E-01	NS	0.82	3.02E-01	NS	1.61
D3JV41	6.40E-01	NS	0.19	1.03E-01	NS	0.87	8.62E-01	NS	0.04	7.69E-01	NS	0.06	7.44E-01	NS	-0.25
F8W0J6	6.41E-01	NS	0.21	3.93E-01	NS	0.42	6.75E-01	NS	-0.13	5.85E-01	NS	0.01	8.13E-01	NS	0.05
V9HWF6	6.44E-01	NS	0.11	3.96E-01	NS	1.03	2.48E-01	NS	-0.63	9.49E-01	NS	-0.36	9.66E-01	NS	-0.42
B4DZ36	6.45E-01	NS	0.14	8.51E-01	NS	0.19	8.06E-01	NS	-0.09	8.19E-01	NS	0.12	7.16E-01	NS	0.19
B4DPQ0	6.48E-01	NS	0.24	2.41E-01	NS	0.33	4.61E-02	NS	0.28	4.02E-01	NS	-0.18	7.64E-01	NS	-0.03
B7Z549	6.50E-01	NS	0.23	1.69E-01	NS	-0.39	1.42E-01	NS	0.57	8.59E-01	NS	0.01	4.53E-01	NS	-0.37
B4DDT3	6.51E-01	NS	-0.14	7.77E-01	NS	-0.22	3.18E-01	NS	0.55	5.87E-01	NS	-0.60	4.70E-01	NS	-0.26
D3DPG0	6.62E-01	NS	0.40	4.02E-01	NS	-0.43	9.53E-02	NS	0.60	5.93E-01	NS	0.21	2.41E-01	NS	-0.48
I3L3D5	6.63E-01	NS	0.42	1.03E-01	NS	1.26	8.42E-02	NS	1.66	9.56E-03	NS	1.53	2.76E-01	NS	0.53
F8VV57	6.66E-01	NS	0.46	1.33E-01	NS	-0.79	3.37E-01	NS	-1.93	4.75E-01	NS	-0.27	9.65E-01	NS	0.03
A0A182DWH7	6.70E-01	NS	-0.15	5.18E-01	NS	0.15	1.72E-01	NS	-0.38	3.42E-01	NS	-0.43	8.51E-01	NS	-0.02
Q13784	6.90E-01	NS	-0.22	3.75E-01	NS	0.53	9.85E-01	NS	0.21	4.31E-01	NS	-0.30	3.59E-01	NS	0.50
Q9UNL6	6.97E-01	NS	1.16	7.97E-01	NS	-1.10	1.14E-01	NS	1.47	8.36E-02	NS	0.62	9.22E-01	NS	-0.25
A0A0U1RR20	7.09E-01	NS	-0.12	2.26E-01	NS	-0.52	4.93E-01	NS	0.18	3.16E-01	NS	0.80	6.93E-01	NS	-0.31
A0A0S2Z3E8	7.12E-01	NS	-0.40	1.17E-01	NS	-1.89	1.15E-01	NS	-1.94	3.58E-01	NS	-0.32	7.94E-01	NS	0.21
A0A024R6N9	7.15E-01	NS	-0.06	6.18E-01	NS	0.11	6.96E-01	NS	0.15	7.44E-01	NS	-0.08	8.86E-01	NS	-0.12
A0A0F7G8J1	7.16E-01	NS	-0.16	3.67E-01	NS	-0.21	5.26E-01	NS	0.25	1.77E-01	NS	-0.41	8.80E-01	NS	0.08
E9PHK0	7.18E-01	NS	0.14	2.73E-02	NS	0.85	1.62E-01	NS	-0.41	5.96E-01	NS	-0.26	6.90E-01	NS	0.04
Q59EA3	7.20E-01	NS	0.37	7.80E-01	NS	0.60	7.84E-01	NS	-0.21	6.05E-01	NS	0.14	4.33E-01	NS	0.97
B2R773	7.32E-01	NS	0.19	9.36E-01	NS	-0.25	7.63E-01	NS	0.13	4.57E-01	NS	-0.62	7.47E-01	NS	-0.16
B0YJC6	7.35E-01	NS	0.14	7.65E-01	NS	0.17	4.25E-01	NS	-0.33	5.60E-01	NS	-0.33	6.74E-01	NS	0.16
B4E1Z4	7.39E-01	NS	0.05	1.74E-01	NS	-0.17	6.36E-01	NS	-0.13	5.46E-01	NS	-0.10	5.36E-01	NS	0.12
V9HWP0	7.49E-01	NS	0.22	4.23E-01	NS	0.48	4.94E-01	NS	0.24	8.54E-01	NS	0.11	6.63E-03	NS	-0.70
A8K1K1	7.53E-01	NS	-0.11	4.31E-01	NS	-0.35	4.60E-01	NS	0.96	9.80E-01	NS	-0.20	2.89E-01	NS	-0.78
A0A384NKS6	7.57E-01	NS	0.05	1.11E-01	NS	0.51	9.14E-02	NS	0.34	2.27E-01	NS	-0.35	1.97E-01	NS	-0.31
Q9UEU7	7.58E-01	NS	0.17	7.97E-01	NS	-0.49	7.84E-01	NS	-0.22	9.07E-01	NS	0.08	9.88E-01	NS	0.14
Q499G5	7.71E-01	NS	0.13	1.25E-01	NS	-0.98	3.03E-01	NS	0.60	1.73E-01	NS	0.92	4.29E-02	NS	-0.96
C9JV77	7.72E-01	NS	0.02	6.91E-01	NS	0.21	5.12E-02	NS	-0.81	2.12E-01	NS	0.40	8.52E-01	NS	0.18
Q53F26	7.90E-01	NS	0.22	1.30E-01	NS	-1.15	6.75E-02	NS	-0.73	7.52E-01	NS	-0.63	8.57E-01	NS	-0.13
B2R950	7.94E-01	NS	0.37	6.93E-01	NS	-0.05	6.19E-01	NS	0.32	8.00E-01	NS	-0.15	2.45E-01	NS	-0.57
D6RAR4	8.00E-01	NS	0.11	5.08E-01	NS	0.37	4.66E-01	NS	-0.32	7.69E-01	NS	0.16	7.00E-01	NS	0.17
A0A024R104	8.02E-01	NS	0.05	1.59E-01	NS	1.05	8.95E-01	NS	-0.14	4.69E-01	NS	0.10	7.82E-01	NS	-0.02
A0A3G1HEF1	8.09E-01	NS	-0.35	3.54E-01	NS	-0.47	4.48E-01	NS	0.25	8.06E-02	NS	-0.61	1.55E-01	NS	-0.48
S4R3Y4	8.10E-01	NS	-0.04	2.93E-02	NS	0.53	6.12E-02	NS	-0.46	2.27E-01	NS	0.60	6.85E-01	NS	-0.05
B3KS79	8.42E-01	NS	-0.08	6.57E-01	NS	-0.10	1.31E-01	NS	0.50	4.25E-01	NS	-0.19	5.24E-01	NS	-0.20
Q8TAY0	8.53E-01	NS	0.08	6.78E-01	NS	-0.30	4.51E-02	NS	0.71	7.07E-01	NS	0.13	2.52E-01	NS	-0.42
M0R2W8	8.60E-01	NS	0.42	5.06E-01	NS	0.92	2.77E-01	NS	0.45	9.96E-01	NS	0.16	2.40E-03	NS	-1.45
A6XND1	8.68E-01	NS	0.02	1.91E-01	NS	0.45	1.80E-01	NS	-0.38	9.94E-01	NS	-0.02	2.84E-01	NS	0.36
V9HWD8	8.72E-01	NS	-0.03	5.91E-01	NS	0.35	8.81E-02	NS	-0.67	5.70E-01	NS	0.23	4.80E-01	NS	0.44
Q53H37	8.76E-01	NS	-0.26	3.64E-01	NS	1.40	5.43E-01	NS	-0.99	7.13E-01	NS	-0.43	3.43E-01	NS	1.48
B7Z555	8.78E-01	NS	-0.01	4.52E-01	NS	-0.24	4.84E-02	NS	0.65	8.18E-01	NS	-0.18	4.35E-01	NS	-0.29
F5H1C6	8.83E-01	NS	-0.70	2.16E-01	NS	0.25	9.93E-01	NS	0.06	3.70E-01	NS	-1.30	6.34E-01	NS	-0.21
B2R6W1	8.84E-01	NS	0.02	8.24E-01	NS	-0.07	3.93E-01	NS	0.24	2.43E-01	NS	-0.22	1.43E-01	NS	-0.32
Q5VY30	8.86E-01	NS	0.11	3.88E-01	NS	0.78	1.18E-01	NS	-0.49	2.47E-01	NS	0.46	2.66E-01	NS	0.41
A0A0S2Z4I5	8.86E-01	NS	-0.12	4.23E-01	NS	0.45	9.96E-01	NS	-0.23	6.24E-01	NS	-0.14	7.20E-01	NS	0.04
A0A087WW43	8.87E-01	NS	0.10	3.65E-01	NS	-0.27	3.93E-01	NS	0.19	2.13E-01	NS	0.25	1.62E-01	NS	-0.39
A0A384N669	8.89E-01	NS	-0.01	2.33E-01	NS	0.55	7.98E-01	NS	0.14	8.18E-01	NS	0.14	4.89E-01	NS	0.36
A8K8Z4	8.96E-01	NS	0.05	4.95E-01	NS	0.19	5.18E-01	NS	-0.12	4.65E-01	NS	-0.21	5.71E-01	NS	-0.13
A0A384MEF1	8.96E-01	NS	0.03	7.61E-01	NS	-0.21	3.52E-01	NS	0.43	8.54E-01	NS	0.03	9.10E-01	NS	0.03
Q5SQ08	9.01E-01	NS	0.06	1.31E-01	NS	-0.39	2.52E-01	NS	0.34	2.97E-01	NS	0.21	1.40E-01	NS	-0.35
Q5J875	9.03E-01	NS	-0.06	2.69E-01	NS	0.43	2.28E-01	NS	-0.43	3.24E-01	NS	-0.31	5.47E-01	NS	-0.30
Q8IVC0	9.11E-01	NS	-0.03	1.81E-01	NS	-0.37	4.81E-02	NS	0.52	3.40E-01	NS	-0.20	1.19E-01	NS	-0.59
J3KPA1	9.12E-01	NS	-0.10	1.21E-01	NS	1.06	3.10E-01	NS	-0.52	5.07E-01	NS	-0.59	5.72E-01	NS	0.09
B2R7F8	9.19E-01	NS	0.01	1.82E-01	NS	-0.26	7.08E-01	NS	0.10	6.67E-01	NS	0.21	5.55E-01	NS	0.17
A2RTY6	9.21E-01	NS	0.16	5.68E-01	NS	-0.17	7.88E-03	NS	0.71	7.20E-01	NS	0.06	1.19E-01	NS	-0.63
Q4LE79	9.23E-01	NS	0.49	5.16E-01	NS	0.10	3.59E-01	NS	-1.77	6.34E-01	NS	-1.29	1.60E-01	NS	-1.22
RT-Cal protein	9.27E-01	NS	0.00	3.52E-01	NS	0.08	1.12E-01	NS	-0.21	8.45E-01	NS	-0.03	7.60E-01	NS	0.04
E7END6	9.29E-01	NS	-0.02	1.50E-01	NS	0.39	5.01E-01	NS	-0.25	6.52E-01	NS	-0.06	9.24E-01	NS	0.04
D9IWP9	9.38E-01	NS	0.13	2.29E-01	NS	0.40	1.26E-01	NS	-0.50	1.47E-01	NS	-0.52	5.95E-01	NS	0.17
B2R815	9.39E-01	NS	0.14	5.30E-01	NS	-0.21	2.59E-01	NS	0.45	4.51E-01	NS	-0.23	8.27E-02	NS	-0.58
V9GYM3	9.39E-01	NS	-0.10	2.31E-01	NS	1.81	8.39E-01	NS	-1.57	9.64E-01	NS	-0.57	1.81E-01	NS	-0.77
D6R934	9.39E-01	NS	0.08	5.55E-01	NS	-0.21	4.55E-03	NS	0.81	8.50E-01	NS	-0.16	3.36E-01	NS	-0.39
A0A087X1J7	9.50E-01	NS	0.09	9.60E-01	NS	0.02	9.57E-02	NS	0.63	8.37E-01	NS	-0.16	2.69E-01	NS	-0.43
E9PIT3	9.51E-01	NS	-0.02	9.38E-01	NS	0.17	1.51E-02	NS	-1.26	3.41E-01	NS	0.97	1.51E-01	NS	1.21
A0A024R035	9.53E-01	NS	-0.03	9.51E-01	NS	0.07	6.49E-01	NS	0.16	6.79E-01	NS	-0.10	6.21E-01	NS	-0.18
K7EQQ3	9.59E-01	NS	0.24	6.78E-01	NS	0.00	2.32E-01	NS	-1.16	5.59E-01	NS	-0.42	4.95E-01	NS	0.29
A0A140VK00	9.69E-01	NS	0.01	6.33E-03	NS	0.59	5.64E-01	NS	-0.10	7.66E-01	NS	-0.31	8.77E-01	NS	-0.05
V9HWE3	9.69E-01	NS	-0.20	4.40E-01	NS	0.29	1.28E-02	NS	2.35	3.85E-01	NS	0.35	5.59E-01	NS	-0.40
Q53GN8	9.75E-01	NS	-0.06	8.77E-01	NS	0.00	5.54E-01	NS	-0.10	6.07E-01	NS	0.13	9.77E-01	NS	-0.06
B4E1C2	9.79E-01	NS	-0.05	1.22E-01	NS	0.44	2.28E-01	NS	-0.30	4.64E-01	NS	-0.27	8.17E-01	NS	0.14
J3QR68	9.87E-01	NS	-0.39	9.48E-01	NS	-0.33	8.30E-01	NS	1.04	6.40E-01	NS	-0.55	7.95E-01	NS	-0.67
B0YIW2	9.94E-01	NS	0.01	6.08E-01	NS	0.23	3.68E-01	NS	-0.96	8.25E-01	NS	0.46	5.45E-01	NS	0.80
A0A1K0GXZ1	9.97E-01	NS	1.02	9.06E-01	NS	-1.47	4.06E-02	NS	3.07	3.74E-02	NS	1.54	9.17E-01	NS	0.26

	4 h			8 h			16 h			1 d			2 d		
**Name**	*p-value*	*FDR*	*log2(FC)*	*p-value*	*FDR*	*log2(FC)*	*p-value*	*FDR*	*log2(FC)*	*p-value*	*FDR*	*log2(FC)*	*p-value*	*FDR*	*log2(FC)*
A0A0S2Z3D5	5.44E-01	NS	-0.53	4.19E-02	NS	1.45	3.06E-01	NS	0.29	1.18E-01	NS	1.08	8.47E-01	NS	-0.36
E7EQ64	1.15E-01	NS	-0.80	5.12E-01	NS	-0.22	8.76E-01	NS	-0.04	6.80E-01	NS	0.44	6.47E-01	NS	0.14
Q5JVE7	3.63E-01	NS	-0.15	7.16E-01	NS	0.11	6.72E-01	NS	-0.07	3.80E-01	NS	0.26	7.47E-02	NS	-0.65
B2R888	8.86E-01	NS	0.02	8.98E-01	NS	-0.07	3.02E-01	NS	0.48	1.76E-01	NS	-0.21	6.63E-01	NS	-0.11
A0N071	3.50E-02	NS	2.24	5.09E-01	NS	1.34	2.19E-01	NS	1.69	8.50E-01	NS	-0.62	9.61E-01	NS	1.00
B4DRY0	3.68E-01	NS	0.92	4.84E-01	NS	1.25	3.66E-02	NS	1.07	4.71E-01	NS	1.71	3.87E-01	NS	1.86
C9JEU5	9.67E-01	NS	-0.31	9.21E-01	NS	-0.07	7.86E-01	NS	0.31	5.25E-01	NS	-0.09	4.80E-01	NS	-0.21
V9HWN8	4.46E-01	NS	-0.28	2.40E-01	NS	0.46	6.99E-01	NS	0.14	7.01E-01	NS	0.07	9.71E-01	NS	0.02
H0YID6	7.05E-01	NS	-0.89	8.21E-01	NS	0.34	7.74E-01	NS	0.98	5.97E-01	NS	-0.17	5.11E-01	NS	1.47
B7ZLE5	6.38E-01	NS	0.12	7.77E-02	NS	0.76	4.64E-01	NS	-0.19	5.31E-01	NS	-0.12	8.35E-01	NS	0.28
B4DRT3	1.47E-01	NS	0.61	1.14E-01	NS	0.64	5.79E-01	NS	0.71	1.19E-01	NS	1.22	4.66E-01	NS	0.48
I3L145	7.98E-01	NS	0.06	6.04E-01	NS	0.18	3.67E-01	NS	0.51	6.83E-01	NS	-0.01	6.56E-01	NS	0.25
A0A3B3ISJ1	4.48E-02	NS	0.51	6.63E-01	NS	0.07	1.64E-01	NS	0.36	2.80E-01	NS	0.21	5.25E-01	NS	-0.10
A0A024R962	7.18E-01	NS	-0.02	1.86E-01	NS	0.47	4.37E-01	NS	-0.89	1.36E-02	NS	1.68	2.45E-01	NS	-1.27
Q53H26	8.83E-01	NS	0.12	1.96E-01	NS	-0.69	5.83E-01	NS	-0.03	9.43E-01	NS	0.48	7.21E-01	NS	0.43
H3BRJ9	4.48E-01	NS	-0.40	1.00E-01	NS	0.82	1.92E-01	NS	0.54	7.81E-01	NS	-0.15	7.80E-01	NS	-0.04
V9HW68	9.81E-01	NS	1.00	5.12E-01	NS	0.15	7.80E-02	NS	0.71	8.23E-01	NS	0.27	4.72E-01	NS	0.75
Q68CK4	9.47E-01	NS	-0.04	2.23E-01	NS	-0.29	8.08E-01	NS	0.01	3.52E-01	NS	-0.23	5.17E-01	NS	0.27
B7ZKJ8	6.45E-01	NS	-0.12	8.57E-01	NS	-0.12	9.86E-01	NS	-0.06	8.00E-01	NS	0.15	3.60E-01	NS	-0.31
Q53GK6	3.35E-01	NS	0.26	6.60E-01	NS	0.06	3.27E-02	NS	1.73	1.69E-02	NS	2.39	5.37E-02	NS	1.63
A0A024R1X6	7.00E-01	NS	0.61	4.99E-01	NS	0.92	7.83E-02	NS	1.21	2.30E-01	NS	1.68	9.55E-01	NS	0.88
L8E853	6.42E-01	NS	-0.12	4.09E-01	NS	0.40	7.37E-01	NS	0.26	4.33E-02	NS	1.15	7.74E-01	NS	0.04
V9HVY1	1.66E-01	NS	-0.95	8.50E-01	NS	0.31	7.37E-01	NS	-0.05	8.91E-01	NS	-0.13	5.30E-01	NS	0.45
A0A3B3IS66	1.82E-01	NS	0.42	8.50E-01	NS	0.03	7.74E-01	NS	0.02	5.62E-01	NS	0.26	8.08E-01	NS	-0.11
Q59E93	9.89E-01	NS	-0.02	6.25E-01	NS	-0.14	8.65E-01	NS	0.67	2.91E-01	NS	-1.00	5.36E-01	NS	0.00
Q5JP69	8.11E-01	NS	0.10	9.12E-02	NS	0.48	1.32E-01	NS	-0.66	1.49E-01	NS	0.75	3.60E-01	NS	-0.20
Q9BS19	3.14E-01	NS	-0.27	8.71E-01	NS	0.03	1.88E-01	NS	-0.45	5.21E-01	NS	0.24	3.42E-01	NS	0.49
F8W7L3	2.96E-01	NS	0.75	8.05E-01	NS	-0.39	4.93E-01	NS	-0.15	6.91E-02	NS	1.68	3.54E-01	NS	0.64
A0A087WSY5	7.26E-01	NS	0.05	6.25E-01	NS	-0.24	5.90E-01	NS	-0.16	9.03E-01	NS	-0.05	3.46E-01	NS	0.34
V9HW98	7.50E-01	NS	0.16	7.73E-01	NS	0.04	5.01E-01	NS	0.46	8.38E-01	NS	0.08	5.33E-01	NS	0.20
A0A2S0BDD1	9.26E-02	NS	-0.30	1.28E-01	NS	0.48	3.24E-01	NS	-0.39	8.73E-01	NS	0.00	4.27E-01	NS	-0.23
A0A2Z4LCH4	2.11E-01	NS	-0.43	6.44E-01	NS	-0.32	6.18E-01	NS	-0.20	7.78E-01	NS	0.09	8.34E-01	NS	-0.21
B2RAN2	9.15E-01	NS	-0.03	8.31E-01	NS	0.08	2.65E-01	NS	-0.26	3.06E-01	NS	0.56	9.15E-01	NS	0.03
D9ZGG2	6.50E-01	NS	-0.11	2.88E-01	NS	-0.16	7.22E-02	NS	-0.40	6.99E-01	NS	-0.05	2.99E-01	NS	-0.20
X6RJP6	4.14E-01	NS	-0.29	2.16E-01	NS	-0.68	5.03E-01	NS	0.97	2.37E-02	NS	3.26	2.69E-02	NS	1.69
A0A024RAA7	9.07E-01	NS	0.02	5.78E-01	NS	0.19	5.58E-01	NS	0.29	1.75E-01	NS	-0.86	8.32E-01	NS	0.22
V9HWE9	8.56E-01	NS	-0.14	5.11E-01	NS	-1.67	5.09E-01	NS	0.98	5.94E-01	NS	-0.40	2.96E-01	NS	-0.84
Q59EP2	3.67E-01	NS	0.33	6.80E-02	NS	0.24	2.60E-01	NS	0.45	9.92E-01	NS	0.00	6.54E-01	NS	0.08
A0A140VJI7	3.56E-01	NS	-0.28	4.85E-01	NS	-0.16	6.66E-01	NS	-0.25	7.61E-01	NS	0.04	4.16E-01	NS	-0.36
A0A0G2JPR0	9.88E-01	NS	0.02	6.51E-01	NS	0.12	8.06E-01	NS	0.13	7.89E-01	NS	-0.16	7.39E-01	NS	0.06
P78492	8.60E-01	NS	-0.02	1.60E-01	NS	0.30	8.45E-01	NS	0.04	5.74E-01	NS	0.09	6.08E-01	NS	-0.15
A0A024R3E3	6.71E-01	NS	1.27	2.64E-01	NS	-1.01	3.75E-01	NS	1.68	9.33E-01	NS	1.70	4.71E-01	NS	0.87
B2RA39	9.45E-01	NS	0.07	1.30E-01	NS	1.60	1.46E-01	NS	1.07	1.40E-01	NS	-1.46	7.38E-01	NS	0.40
B2CIS9	4.67E-01	NS	0.19	7.59E-01	NS	-0.13	4.57E-02	NS	1.89	8.96E-01	NS	-0.50	6.91E-01	NS	0.23
E3UN46	8.27E-01	NS	0.02	7.11E-01	NS	-0.10	4.99E-01	NS	-0.17	3.02E-02	NS	-0.66	6.92E-01	NS	-0.03
S4R471	9.62E-01	NS	-0.06	4.74E-01	NS	-0.26	5.65E-01	NS	-0.18	1.15E-01	NS	-0.63	2.82E-01	NS	-0.45
F2RM35	2.73E-01	NS	-0.17	2.05E-01	NS	-0.42	7.23E-01	NS	-0.11	7.95E-01	NS	-0.07	6.10E-02	NS	-0.67
A0A384MDV8	2.77E-01	NS	0.17	8.86E-01	NS	-0.05	3.74E-01	NS	-0.36	6.44E-01	NS	0.15	5.39E-01	NS	-0.24
E9KL23	8.36E-01	NS	-0.11	5.78E-01	NS	0.04	7.80E-01	NS	0.73	8.67E-01	NS	0.16	8.16E-01	NS	0.18
Q96K68	6.25E-01	NS	0.02	1.17E-01	NS	-0.95	4.16E-01	NS	-0.41	4.90E-01	NS	-1.63	3.86E-01	NS	0.47
B4DVE1	5.65E-02	NS	-0.88	6.23E-01	NS	-1.05	9.42E-01	NS	0.19	5.44E-01	NS	-1.23	8.90E-01	NS	-0.20
B2R4M6	2.99E-01	NS	1.03	2.15E-01	NS	1.04	1.32E-01	NS	1.24	5.12E-01	NS	1.39	2.04E-01	NS	1.67
A5PL27	9.39E-01	NS	-0.25	2.49E-01	NS	0.80	7.48E-01	NS	0.45	2.55E-01	NS	0.66	8.76E-01	NS	-0.08
B4E3P1	2.55E-01	NS	0.38	4.88E-01	NS	0.31	3.20E-01	NS	0.81	6.89E-02	NS	2.24	5.39E-02	NS	1.27
D3DNN4	4.43E-01	NS	0.45	8.99E-01	NS	0.25	9.93E-01	NS	0.45	9.89E-01	NS	0.77	9.07E-01	NS	-0.05
F8W876	2.14E-01	NS	-0.25	5.06E-01	NS	0.17	6.67E-01	NS	0.11	8.11E-01	NS	0.03	8.75E-01	NS	0.04
A0A024RAG6	4.72E-01	NS	-0.15	4.78E-01	NS	0.18	3.11E-01	NS	0.46	7.12E-01	NS	-0.22	7.38E-01	NS	0.17
V9HWA9	8.75E-01	NS	0.02	5.77E-01	NS	0.12	6.45E-01	NS	0.29	8.10E-01	NS	-0.10	5.32E-01	NS	-0.17
C0JYY2	2.68E-01	NS	-0.31	7.24E-01	NS	0.04	9.22E-01	NS	0.00	2.42E-01	NS	-0.34	8.04E-01	NS	0.03
A0A0A0MRJ7	1.82E-01	NS	-0.43	8.46E-03	NS	-0.49	2.49E-01	NS	-0.55	3.92E-01	NS	-0.24	7.16E-01	NS	-0.16
Q8NBH6	5.45E-01	NS	0.22	6.09E-01	NS	0.14	3.30E-01	NS	0.44	7.70E-01	NS	-0.12	9.59E-01	NS	0.10
Q05CV2	8.09E-01	NS	-0.12	2.56E-01	NS	-0.42	7.40E-01	NS	0.23	4.50E-01	NS	-0.39	9.04E-01	NS	0.18
F5GXS0	8.95E-01	NS	0.03	2.20E-01	NS	0.25	6.56E-01	NS	0.16	9.66E-01	NS	0.02	8.26E-01	NS	-0.11
Q6MZL2	7.23E-01	NS	-0.07	9.37E-01	NS	-0.16	4.13E-01	NS	0.17	3.04E-01	NS	-0.27	6.32E-01	NS	0.11
A0A0J9YY65	5.04E-01	NS	-0.20	8.01E-01	NS	-0.09	4.12E-01	NS	0.36	6.93E-02	NS	-0.72	9.51E-01	NS	-0.05
B3KNX0	1.91E-01	NS	-0.61	7.57E-01	NS	0.00	2.83E-01	NS	0.55	5.45E-01	NS	0.18	6.92E-01	NS	-0.10
H6VRG1	7.09E-01	NS	-0.30	4.17E-01	NS	-0.42	2.09E-01	NS	0.62	4.32E-01	NS	0.81	6.47E-01	NS	1.48
B7Z8Q5	5.01E-01	NS	-0.38	9.33E-01	NS	-0.08	3.81E-01	NS	0.58	5.03E-01	NS	-0.32	6.16E-01	NS	-0.47
B1AP58	9.36E-01	NS	0.05	4.41E-01	NS	-0.40	1.09E-01	NS	0.63	7.59E-01	NS	-0.31	6.78E-01	NS	-0.09
B4DPH4	8.00E-01	NS	-0.07	7.94E-01	NS	-0.13	9.91E-01	NS	0.03	9.21E-01	NS	-0.03	4.11E-01	NS	-0.30
A0A140VKF3	5.53E-01	NS	-0.17	9.05E-01	NS	-0.07	3.39E-01	NS	0.40	9.81E-01	NS	-0.16	6.27E-01	NS	0.29
A0A024R9Q1	6.56E-01	NS	0.05	6.58E-01	NS	-0.05	3.52E-01	NS	0.52	3.18E-01	NS	0.60	8.49E-01	NS	-0.24
A0A0G2JPA8	2.38E-01	NS	-0.18	7.75E-01	NS	-0.07	9.24E-01	NS	-0.02	1.49E-01	NS	-0.43	4.78E-01	NS	-0.26
H0YAC1	9.42E-01	NS	-0.04	1.81E-01	NS	0.30	3.25E-01	NS	-0.45	2.75E-01	NS	0.24	4.29E-01	NS	-0.31
Q59GS8	9.94E-01	NS	0.09	4.42E-01	NS	0.22	8.51E-01	NS	0.11	6.84E-01	NS	-0.10	8.39E-01	NS	-0.20
B3KT06	4.70E-01	NS	0.08	6.69E-02	NS	2.61	6.18E-01	NS	0.55	4.11E-01	NS	-0.55	4.15E-01	NS	-1.75
J3K000	4.33E-01	NS	0.18	1.02E-01	NS	0.64	2.79E-01	NS	0.53	9.85E-01	NS	0.00	9.87E-01	NS	-0.07
D9YZU5	9.68E-02	NS	2.59	6.74E-01	NS	-0.09	1.92E-01	NS	2.13	7.61E-01	NS	-1.02	5.79E-01	NS	1.58
K7ERE3	7.28E-01	NS	0.14	2.86E-01	NS	-1.36	7.24E-01	NS	1.04	4.34E-02	NS	1.31	9.30E-01	NS	1.45
G3V2W1	3.71E-01	NS	0.19	9.46E-01	NS	0.21	4.42E-01	NS	0.37	3.35E-01	NS	-0.53	7.11E-01	NS	0.09
Q9H2E1	2.19E-01	NS	3.33	3.04E-01	NS	-0.93	4.55E-01	NS	-0.32	4.93E-01	NS	0.70	3.97E-01	NS	-0.49
A0A1B0GUU9	3.09E-01	NS	-0.42	2.35E-01	NS	-0.88	5.68E-01	NS	-0.52	5.34E-02	NS	-1.03	3.52E-01	NS	0.49
D0PNI1	5.87E-02	NS	1.19	4.98E-02	NS	0.70	2.16E-01	NS	-0.75	3.04E-01	NS	1.04	1.75E-01	NS	1.09
Q59EZ3	3.81E-01	NS	-0.94	2.35E-02	NS	-2.03	4.15E-01	NS	0.47	3.82E-01	NS	-0.76	9.79E-01	NS	-0.08
Q6LAN8	7.81E-01	NS	0.55	7.79E-01	NS	-0.11	7.12E-01	NS	0.34	5.17E-01	NS	0.94	5.32E-01	NS	0.32
D3JV41	2.25E-01	NS	0.39	5.08E-01	NS	-0.22	5.36E-01	NS	0.35	1.27E-02	NS	1.09	2.25E-01	NS	-0.69
F8W0J6	2.33E-01	NS	-0.93	8.61E-01	NS	0.38	9.27E-01	NS	-0.54	7.01E-01	NS	-0.24	3.09E-01	NS	0.72
V9HWF6	7.22E-01	NS	-0.18	6.89E-01	NS	0.23	6.65E-01	NS	-0.04	9.06E-01	NS	0.28	7.99E-01	NS	-0.86
B4DZ36	8.72E-01	NS	0.02	5.36E-01	NS	0.38	8.52E-01	NS	0.02	8.61E-01	NS	-0.18	8.25E-01	NS	0.12
B4DPQ0	7.32E-01	NS	0.05	8.03E-01	NS	-0.06	8.02E-01	NS	0.13	3.75E-01	NS	-0.31	3.82E-01	NS	0.24
B7Z549	5.44E-01	NS	0.31	7.43E-01	NS	0.07	7.02E-01	NS	0.27	9.90E-01	NS	0.01	6.99E-01	NS	-0.23
B4DDT3	5.81E-01	NS	-0.28	8.70E-01	NS	-0.43	7.04E-01	NS	0.39	1.77E-01	NS	-0.91	2.44E-01	NS	0.28
D3DPG0	4.40E-01	NS	0.15	9.15E-01	NS	-0.10	2.90E-01	NS	0.50	4.63E-01	NS	-0.34	8.61E-01	NS	0.02
I3L3D5	2.55E-01	NS	0.28	9.53E-01	NS	0.03	1.62E-02	NS	1.95	2.16E-02	NS	2.61	1.64E-01	NS	1.18
F8VV57	8.89E-01	NS	0.30	2.14E-01	NS	-0.60	8.73E-01	NS	0.04	4.59E-01	NS	0.94	8.16E-01	NS	0.90
A0A182DWH7	4.75E-01	NS	-0.28	1.95E-01	NS	-0.53	6.69E-01	NS	-0.24	3.79E-01	NS	-0.23	9.29E-01	NS	0.01
Q13784	4.23E-01	NS	0.66	4.95E-01	NS	0.59	5.98E-01	NS	0.56	6.87E-01	NS	0.14	8.05E-01	NS	0.20
Q9UNL6	1.77E-01	NS	1.67	7.76E-01	NS	0.22	2.74E-01	NS	1.00	9.66E-01	NS	-0.44	8.88E-01	NS	1.37
A0A0U1RR20	9.47E-01	NS	0.25	3.77E-01	NS	0.27	7.21E-01	NS	0.02	4.37E-01	NS	-0.28	4.08E-01	NS	-0.46
A0A0S2Z3E8	1.12E-01	NS	1.06	2.38E-01	NS	1.45	8.26E-01	NS	0.20	8.31E-01	NS	-0.68	6.57E-01	NS	-0.97
A0A024R6N9	1.65E-01	NS	-0.40	6.87E-01	NS	0.19	7.55E-01	NS	0.08	7.95E-01	NS	0.08	5.51E-01	NS	0.15
A0A0F7G8J1	7.13E-01	NS	0.12	8.67E-01	NS	0.06	6.52E-01	NS	0.22	1.27E-01	NS	0.65	3.81E-01	NS	-0.38
E9PHK0	5.17E-01	NS	0.32	7.65E-01	NS	-0.04	8.05E-01	NS	0.23	8.15E-01	NS	-0.12	9.58E-01	NS	-0.08
Q59EA3	1.96E-01	NS	-0.74	1.14E-01	NS	0.78	1.37E-01	NS	1.13	9.47E-02	NS	0.92	9.77E-01	NS	-0.74
B2R773	3.71E-01	NS	-0.48	7.88E-01	NS	-0.34	8.62E-01	NS	0.17	8.69E-01	NS	-0.48	3.50E-01	NS	2.88
B0YJC6	8.12E-01	NS	0.02	8.78E-01	NS	0.20	5.76E-01	NS	-0.20	6.60E-01	NS	0.13	9.91E-01	NS	0.03
B4E1Z4	4.60E-01	NS	-0.18	2.44E-01	NS	0.21	7.61E-01	NS	-0.04	3.37E-01	NS	-0.27	6.74E-01	NS	0.07
V9HWP0	8.07E-01	NS	-0.18	7.07E-01	NS	-0.20	9.00E-01	NS	0.10	6.42E-01	NS	-0.54	6.82E-01	NS	-0.31
A8K1K1	9.29E-01	NS	-0.13	4.86E-01	NS	-0.61	9.27E-01	NS	0.02	9.88E-01	NS	-0.32	8.89E-01	NS	0.35
A0A384NKS6	5.93E-01	NS	0.10	8.65E-01	NS	-0.08	5.64E-01	NS	0.15	7.08E-02	NS	-0.75	6.85E-02	NS	-0.20
Q9UEU7	6.27E-01	NS	0.27	2.82E-01	NS	0.53	6.75E-01	NS	-0.66	3.86E-01	NS	0.94	8.75E-01	NS	-0.75
Q499G5	7.06E-01	NS	0.23	2.27E-01	NS	0.29	5.34E-01	NS	-0.21	3.62E-01	NS	0.38	2.71E-01	NS	-0.71
C9JV77	9.94E-01	NS	0.02	5.47E-01	NS	-0.37	5.68E-01	NS	-0.35	9.57E-01	NS	0.07	8.55E-01	NS	-0.21
Q53F26	8.20E-01	NS	-0.08	5.01E-01	NS	0.46	6.84E-01	NS	-1.05	5.57E-01	NS	-0.16	8.68E-01	NS	-0.44
B2R950	7.16E-01	NS	0.17	6.77E-01	NS	0.11	7.16E-01	NS	0.26	8.05E-01	NS	-0.20	4.79E-01	NS	0.35
D6RAR4	8.00E-01	NS	0.05	8.87E-01	NS	-0.12	9.46E-01	NS	-0.10	4.43E-01	NS	0.32	6.81E-01	NS	-0.28
A0A024R104	7.62E-01	NS	-0.04	9.11E-01	NS	-0.08	3.94E-01	NS	-0.51	3.55E-01	NS	-0.39	5.07E-01	NS	0.20
A0A3G1HEF1	7.50E-01	NS	-0.17	2.58E-01	NS	-0.45	3.18E-01	NS	-0.30	3.23E-01	NS	0.31	6.00E-01	NS	-0.18
S4R3Y4	7.68E-01	NS	0.07	4.75E-01	NS	-0.16	2.98E-01	NS	-0.43	5.04E-01	NS	0.14	2.35E-01	NS	-0.47
B3KS79	6.60E-01	NS	-0.23	6.79E-01	NS	0.09	4.81E-01	NS	0.21	6.20E-01	NS	-0.25	5.56E-01	NS	0.12
Q8TAY0	8.18E-01	NS	0.09	4.26E-01	NS	0.17	2.26E-01	NS	0.38	9.95E-01	NS	0.04	8.04E-01	NS	0.03
M0R2W8	9.91E-01	NS	-0.10	4.17E-01	NS	-0.34	4.25E-01	NS	0.23	4.01E-01	NS	-0.49	8.21E-01	NS	-0.10
A6XND1	9.66E-01	NS	-0.05	3.65E-01	NS	-0.25	7.24E-01	NS	-0.16	7.99E-01	NS	0.04	5.45E-01	NS	-0.13
V9HWD8	8.97E-01	NS	-0.15	3.93E-01	NS	-0.38	3.90E-01	NS	-0.51	8.66E-01	NS	-0.03	9.58E-01	NS	-0.10
Q53H37	6.71E-01	NS	0.61	1.79E-01	NS	2.35	7.77E-01	NS	1.37	5.77E-01	NS	1.21	3.22E-01	NS	1.03
B7Z555	9.65E-01	NS	-0.02	1.07E-01	NS	0.32	5.91E-01	NS	0.35	8.08E-01	NS	-0.18	8.67E-01	NS	0.04
F5H1C6	4.16E-01	NS	0.27	2.61E-01	NS	-0.50	3.89E-01	NS	-0.74	3.37E-02	NS	-1.41	3.24E-01	NS	0.69
B2R6W1	3.93E-01	NS	0.04	6.97E-01	NS	-0.17	8.98E-01	NS	0.04	5.00E-01	NS	-0.25	3.56E-01	NS	-0.16
Q5VY30	8.43E-01	NS	-0.07	8.94E-01	NS	0.08	3.77E-01	NS	-0.59	2.65E-01	NS	0.38	2.31E-01	NS	-0.77
A0A0S2Z4I5	5.15E-01	NS	-0.17	6.13E-01	NS	0.27	6.47E-01	NS	0.11	8.45E-01	NS	-0.52	7.87E-01	NS	-0.05
A0A087WW43	7.43E-01	NS	-0.15	7.94E-01	NS	-0.01	9.70E-01	NS	0.06	7.37E-01	NS	-0.09	5.80E-01	NS	-0.14
A0A384N669	6.50E-01	NS	0.29	8.09E-01	NS	0.08	7.13E-01	NS	0.08	7.84E-01	NS	0.16	7.06E-01	NS	0.03
A8K8Z4	6.12E-01	NS	0.09	6.14E-01	NS	-0.09	9.55E-01	NS	0.00	5.60E-01	NS	0.16	8.21E-01	NS	-0.01
A0A384MEF1	5.55E-01	NS	0.10	4.75E-01	NS	0.18	1.71E-01	NS	0.45	8.02E-01	NS	0.08	7.56E-01	NS	-0.11
Q5SQ08	6.39E-01	NS	0.18	4.21E-01	NS	-0.17	9.77E-01	NS	0.14	6.34E-01	NS	0.08	4.01E-01	NS	-0.22
Q5J875	9.16E-01	NS	0.00	1.64E-01	NS	-0.35	3.91E-01	NS	-0.37	8.17E-01	NS	-0.20	1.00E-01	NS	-0.67
Q8IVC0	5.03E-01	NS	-0.23	7.15E-01	NS	-0.06	6.72E-01	NS	0.11	2.47E-01	NS	-0.38	9.86E-01	NS	-0.08
J3KPA1	7.94E-01	NS	-0.14	1.35E-01	NS	-0.74	6.32E-01	NS	-0.08	7.64E-01	NS	-0.30	6.82E-01	NS	0.19
B2R7F8	7.61E-01	NS	-0.07	9.14E-01	NS	0.03	7.53E-01	NS	-0.04	9.80E-01	NS	-0.03	7.74E-01	NS	-0.04
A2RTY6	8.75E-01	NS	-0.05	8.78E-01	NS	0.00	5.90E-01	NS	0.29	9.02E-01	NS	-0.11	2.95E-01	NS	-0.36
Q4LE79	8.79E-02	NS	-1.53	8.22E-01	NS	0.13	5.64E-01	NS	-0.24	2.24E-01	NS	0.65	2.36E-01	NS	-0.59
RT-Cal protein	8.80E-01	NS	-0.02	6.65E-01	NS	0.04	2.00E-01	NS	-0.23	1.74E-01	NS	0.29	1.99E-01	NS	-0.19
E7END6	8.32E-01	NS	-0.13	5.93E-01	NS	-0.11	4.92E-01	NS	-0.17	7.78E-01	NS	-0.01	4.38E-01	NS	-0.26
D9IWP9	9.14E-01	NS	-0.14	2.96E-01	NS	-0.22	5.45E-01	NS	-0.31	3.78E-01	NS	-0.32	5.13E-01	NS	-0.10
B2R815	7.52E-01	NS	0.02	6.38E-01	NS	-0.24	2.70E-01	NS	0.36	7.33E-01	NS	-0.22	7.70E-01	NS	0.18
V9GYM3	3.05E-01	NS	2.37	9.33E-01	NS	-1.03	2.85E-01	NS	0.65	2.65E-01	NS	2.38	6.71E-01	NS	0.90
D6R934	6.53E-01	NS	-0.18	8.27E-01	NS	0.04	5.19E-01	NS	0.40	3.22E-01	NS	-0.67	8.53E-01	NS	-0.01
A0A087X1J7	7.04E-01	NS	0.07	8.79E-01	NS	-0.10	9.09E-02	NS	0.29	2.57E-01	NS	-0.54	4.24E-01	NS	-0.32
E9PIT3	7.20E-01	NS	-0.03	3.07E-01	NS	0.59	4.27E-01	NS	-0.52	1.27E-01	NS	0.81	4.65E-01	NS	-0.60
A0A024R035	9.88E-01	NS	0.03	9.61E-01	NS	0.07	6.25E-01	NS	0.21	6.36E-01	NS	0.14	9.35E-01	NS	-0.06
K7EQQ3	6.95E-01	NS	-0.66	5.72E-01	NS	-0.37	3.12E-01	NS	0.51	5.38E-01	NS	1.10	8.14E-01	NS	0.80
A0A140VK00	8.30E-01	NS	0.13	4.20E-01	NS	-0.20	9.80E-01	NS	0.17	9.13E-01	NS	-0.01	7.49E-01	NS	0.24
V9HWE3	9.73E-01	NS	0.21	5.59E-01	NS	-0.33	2.53E-01	NS	-0.52	2.38E-01	NS	-1.32	7.57E-01	NS	0.50
Q53GN8	7.87E-01	NS	-0.11	9.48E-01	NS	0.02	4.58E-01	NS	-0.45	4.92E-01	NS	0.14	2.80E-01	NS	-0.52
B4E1C2	5.06E-01	NS	-0.19	2.04E-01	NS	-0.39	9.17E-01	NS	-0.13	4.25E-01	NS	-0.37	7.10E-01	NS	0.06
J3QR68	5.36E-01	NS	-0.07	2.89E-01	NS	-2.04	3.74E-01	NS	-0.84	6.99E-01	NS	-0.99	9.11E-01	NS	0.32
B0YIW2	2.21E-01	NS	0.57	3.68E-01	NS	-1.28	5.13E-01	NS	0.25	1.01E-01	NS	1.04	4.44E-01	NS	-0.58
A0A1K0GXZ1	1.59E-01	NS	2.15	5.94E-01	NS	0.39	3.64E-01	NS	1.41	7.19E-01	NS	-0.98	5.27E-01	NS	1.87

	3 d			7 d			14 d			21 d			All times		
**Name**	*p-value*	*FDR*	*log2(FC)*	*p-value*	*FDR*	*log2(FC)*	*p-value*	*FDR*	*log2(FC)*	*p-value*	*FDR*	*log2(FC)*	*p-value*	*FDR*	*log2(FC)*
A0A0S2Z3D5	3.75E-01	NS	0.36	3.72E-01	NS	0.47	8.42E-02	NS	-0.93	3.09E-01	NS	-0.46	5.03E-01	NS	0.19
E7EQ64	8.90E-01	NS	0.24	4.57E-01	NS	0.32	4.28E-01	NS	0.58	3.31E-01	NS	0.97	5.62E-01	NS	-0.08
Q5JVE7	9.42E-01	NS	-0.03	5.69E-01	NS	0.22	1.52E-02	NS	-0.92	1.24E-01	NS	-0.38	2.37E-03	NS	-0.20
B2R888	3.18E-01	NS	0.23	3.33E-01	NS	-0.29	6.86E-01	NS	-0.05	2.69E-01	NS	-0.25	9.67E-01	NS	-0.02
A0N071	2.62E-01	NS	-1.09	8.42E-01	NS	-0.19	2.72E-01	NS	0.92	2.53E-01	NS	0.53	6.28E-03	NS	1.18
B4DRY0	1.68E-02	NS	1.20	2.82E-01	NS	-0.47	9.74E-01	NS	0.18	7.27E-02	NS	-1.10	3.11E-01	NS	-0.07
C9JEU5	5.16E-01	NS	-0.78	5.26E-01	NS	0.96	2.96E-01	NS	-0.97	6.87E-01	NS	-0.25	2.57E-01	NS	-0.27
V9HWN8	8.03E-01	NS	0.14	9.58E-01	NS	0.01	7.68E-01	NS	0.07	8.78E-01	NS	0.05	8.71E-01	NS	-0.04
H0YID6	1.33E-01	NS	2.01	4.03E-02	NS	-1.75	8.70E-01	NS	0.22	5.91E-01	NS	-0.43	9.94E-01	NS	0.04
B7ZLE5	8.04E-02	NS	-0.47	9.02E-01	NS	-0.18	2.19E-01	NS	-0.33	1.40E-01	NS	-1.03	7.85E-01	NS	-0.02
B4DRT3	5.43E-02	NS	1.33	6.77E-01	NS	0.72	4.17E-01	NS	-0.25	1.97E-01	NS	-0.54	3.83E-03	NS	0.67
I3L145	5.07E-01	NS	0.28	4.73E-01	NS	-0.24	1.54E-01	NS	0.79	8.46E-01	NS	0.00	1.01E-01	NS	0.15
A0A3B3ISJ1	5.80E-01	NS	-0.06	4.40E-01	NS	-0.14	2.57E-01	NS	-0.15	1.46E-01	NS	-0.19	1.59E-01	NS	0.07
A0A024R962	1.27E-01	NS	-0.76	7.91E-02	NS	1.26	2.06E-02	NS	-1.10	9.61E-01	NS	0.14	8.36E-01	NS	0.04
Q53H26	2.64E-01	NS	0.65	2.36E-01	NS	-1.39	5.95E-01	NS	-0.36	2.85E-01	NS	0.50	4.05E-01	NS	-0.20
H3BRJ9	7.43E-01	NS	0.57	5.66E-01	NS	-0.63	6.97E-01	NS	0.84	5.29E-01	NS	0.44	5.73E-01	NS	-0.07
V9HW68	3.27E-01	NS	0.65	1.57E-02	NS	-2.02	4.54E-02	NS	1.55	7.41E-02	NS	1.10	8.34E-01	NS	0.05
Q68CK4	7.04E-01	NS	-0.01	4.49E-01	NS	0.25	3.96E-01	NS	0.28	3.34E-01	NS	0.41	4.78E-01	NS	-0.04
B7ZKJ8	3.03E-01	NS	-0.27	3.07E-01	NS	0.30	6.38E-01	NS	-0.15	7.90E-01	NS	0.06	4.61E-01	NS	-0.07
Q53GK6	5.09E-01	NS	0.40	2.71E-01	NS	-0.40	2.52E-01	NS	0.30	3.08E-02	NS	1.87	2.04E-05	NS	1.14
A0A024R1X6	2.54E-01	NS	0.79	7.60E-01	NS	-0.21	1.19E-01	NS	-0.59	2.64E-01	NS	-0.64	3.35E-01	NS	-0.18
L8E853	3.92E-01	NS	-0.31	5.16E-01	NS	0.23	1.29E-01	NS	-0.73	8.48E-01	NS	-0.18	4.12E-01	NS	0.02
V9HVY1	7.03E-01	NS	-0.27	9.93E-01	NS	0.07	3.25E-01	NS	-0.94	3.42E-01	NS	0.06	4.71E-01	NS	-0.06
A0A3B3IS66	8.68E-01	NS	0.05	8.72E-01	NS	0.08	7.80E-01	NS	-0.15	3.83E-01	NS	0.19	1.00E-01	NS	0.16
Q59E93	5.02E-01	NS	0.17	1.45E-01	NS	-0.92	2.00E-01	NS	0.42	2.03E-01	NS	0.42	8.97E-01	NS	-0.07
Q5JP69	6.95E-02	NS	-0.72	8.93E-01	NS	0.03	9.55E-01	NS	-0.08	5.33E-01	NS	-0.16	9.72E-01	NS	0.01
Q9BS19	5.32E-01	NS	0.13	5.43E-01	NS	0.26	6.12E-01	NS	0.14	9.44E-01	NS	-0.03	9.31E-01	NS	0.00
F8W7L3	2.36E-01	NS	-0.41	7.17E-01	NS	-0.66	3.29E-01	NS	0.61	6.36E-01	NS	-0.17	7.77E-01	NS	-0.07
A0A087WSY5	5.76E-01	NS	-0.12	2.58E-01	NS	-0.31	3.87E-01	NS	0.30	9.91E-01	NS	-0.07	2.17E-01	NS	-0.14
V9HW98	1.48E-01	NS	-0.76	1.83E-01	NS	-0.64	2.01E-01	NS	-0.88	5.32E-01	NS	-0.26	5.62E-01	NS	-0.12
A0A2S0BDD1	5.66E-01	NS	0.18	1.30E-02	NS	0.64	3.79E-01	NS	-0.31	8.99E-01	NS	-0.18	5.25E-01	NS	-0.08
A0A2Z4LCH4	8.45E-01	NS	-0.13	7.73E-01	NS	-0.29	9.64E-01	NS	0.01	8.05E-01	NS	0.05	1.46E-01	NS	-0.23
B2RAN2	7.00E-01	NS	-0.08	1.60E-01	NS	0.40	2.97E-01	NS	0.41	6.51E-01	NS	-0.10	2.11E-01	NS	0.15
D9ZGG2	1.24E-01	NS	-0.27	4.63E-01	NS	0.12	2.76E-01	NS	-0.13	9.36E-01	NS	0.02	5.38E-03	NS	-0.11
X6RJP6	1.45E-01	NS	0.78	7.98E-01	NS	-0.43	1.76E-02	NS	1.08	2.88E-02	NS	2.38	2.96E-04	NS	1.23
A0A024RAA7	1.66E-01	NS	0.75	4.33E-01	NS	-0.39	1.95E-01	NS	0.49	3.19E-01	NS	0.40	1.80E-01	NS	0.16
V9HWE9	4.78E-01	NS	0.31	1.33E-01	NS	-1.07	1.40E-01	NS	0.96	1.28E-01	NS	0.78	8.33E-02	NS	0.08
Q59EP2	8.79E-01	NS	0.02	4.95E-01	NS	-0.20	1.68E-01	NS	0.33	3.00E-01	NS	0.23	2.22E-02	NS	0.18
A0A140VJI7	4.35E-01	NS	0.12	2.65E-01	NS	0.49	1.32E-01	NS	-0.63	9.25E-01	NS	0.05	6.61E-02	NS	-0.16
A0A0G2JPR0	8.90E-01	NS	0.16	4.29E-01	NS	-0.27	4.78E-01	NS	0.15	1.11E-01	NS	0.38	4.54E-01	NS	0.07
P78492	1.86E-01	NS	-0.34	1.68E-01	NS	0.25	2.82E-01	NS	-0.35	6.97E-01	NS	-0.08	9.87E-01	NS	-0.01
A0A024R3E3	7.00E-02	NS	1.28	4.04E-01	NS	-2.64	1.03E-01	NS	1.56	3.12E-03	NS	2.82	2.98E-01	NS	0.28
B2RA39	1.42E-01	NS	1.28	7.93E-01	NS	0.24	2.17E-01	NS	1.42	2.44E-01	NS	-2.25	2.57E-01	NS	0.27
B2CIS9	7.20E-01	NS	0.11	2.96E-01	NS	0.66	9.32E-02	NS	0.57	8.55E-01	NS	-0.24	4.58E-01	NS	0.00
E3UN46	5.92E-01	NS	0.24	9.04E-01	NS	0.04	6.57E-01	NS	0.38	9.48E-01	NS	-0.16	9.80E-01	NS	0.01
S4R471	7.15E-01	NS	0.14	2.65E-01	NS	-0.32	2.05E-01	NS	0.49	6.63E-01	NS	0.21	4.80E-01	NS	-0.03
F2RM35	5.24E-01	NS	-0.19	6.12E-01	NS	0.07	7.53E-01	NS	-0.05	4.10E-01	NS	-0.28	2.43E-02	NS	-0.18
A0A384MDV8	4.20E-01	NS	0.37	8.16E-02	NS	-0.47	1.85E-01	NS	0.52	3.58E-01	NS	0.36	8.67E-01	NS	-0.03
E9KL23	3.40E-01	NS	0.19	6.98E-01	NS	-0.09	1.85E-01	NS	0.58	1.09E-01	NS	1.16	3.25E-01	NS	0.24
Q96K68	6.21E-01	NS	0.18	5.72E-01	NS	-0.54	1.39E-01	NS	0.73	1.86E-01	NS	0.72	1.04E-01	NS	-0.47
B4DVE1	4.44E-01	NS	0.35	7.28E-02	NS	0.56	5.05E-01	NS	-0.32	8.37E-01	NS	0.15	2.27E-01	NS	-0.32
B2R4M6	3.36E-01	NS	0.59	8.09E-02	NS	-0.69	7.80E-01	NS	-0.31	6.21E-01	NS	-0.50	2.67E-02	NS	1.03
A5PL27	8.21E-01	NS	0.01	4.93E-01	NS	0.21	5.03E-01	NS	0.36	9.48E-01	NS	-0.18	1.82E-01	NS	0.13
B4E3P1	3.28E-01	NS	0.49	2.56E-01	NS	-0.72	2.57E-01	NS	0.52	4.00E-03	NS	2.36	3.52E-05	NS	1.28
D3DNN4	6.87E-01	NS	0.07	3.26E-01	NS	0.20	3.18E-01	NS	0.46	7.37E-01	NS	-0.54	9.82E-01	NS	0.13
F8W876	6.69E-01	NS	0.16	5.21E-01	NS	0.14	5.32E-01	NS	-0.13	6.98E-01	NS	-0.01	9.16E-01	NS	-0.01
A0A024RAG6	2.46E-01	NS	0.67	1.26E-01	NS	-0.93	1.30E-02	NS	1.09	3.03E-01	NS	0.47	2.65E-01	NS	0.08
V9HWA9	9.82E-01	NS	0.07	1.26E-01	NS	-0.49	3.18E-01	NS	0.29	7.87E-01	NS	-0.19	9.52E-01	NS	0.00
C0JYY2	2.68E-01	NS	0.35	1.20E-02	NS	-0.60	3.77E-02	NS	0.32	2.51E-01	NS	0.24	2.38E-01	NS	-0.10
A0A0A0MRJ7	7.34E-01	NS	0.13	7.52E-01	NS	-0.13	8.48E-01	NS	-0.07	3.89E-01	NS	0.22	2.90E-02	NS	-0.18
Q8NBH6	7.53E-01	NS	0.05	4.30E-01	NS	-0.33	9.76E-01	NS	-0.07	9.36E-01	NS	-0.02	5.44E-01	NS	0.03
Q05CV2	4.50E-01	NS	0.30	6.26E-01	NS	0.15	3.37E-01	NS	0.47	5.85E-01	NS	-0.27	7.73E-01	NS	0.03
F5GXS0	7.64E-01	NS	-0.04	3.65E-01	NS	-0.26	6.25E-01	NS	0.17	5.72E-01	NS	0.17	7.28E-01	NS	0.04
Q6MZL2	3.79E-01	NS	0.22	4.00E-01	NS	0.31	3.28E-01	NS	-0.54	2.57E-01	NS	0.58	9.92E-01	NS	-0.02
A0A0J9YY65	4.19E-01	NS	0.41	2.07E-01	NS	-0.31	1.94E-02	NS	0.39	9.02E-01	NS	-0.11	2.41E-01	NS	-0.10
B3KNX0	6.69E-01	NS	0.10	4.68E-01	NS	-0.25	3.49E-01	NS	0.31	5.19E-01	NS	-0.30	3.07E-01	NS	-0.12
H6VRG1	2.97E-02	NS	1.45	4.64E-01	NS	-0.28	8.48E-01	NS	-0.06	7.02E-01	NS	-0.33	5.73E-01	NS	0.28
B7Z8Q5	6.89E-01	NS	-0.07	1.20E-01	NS	-0.90	3.26E-01	NS	0.31	2.06E-01	NS	0.75	8.23E-01	NS	0.05
B1AP58	9.21E-01	NS	-0.12	5.39E-02	NS	-0.72	2.86E-01	NS	0.38	7.88E-01	NS	-0.21	8.54E-01	NS	-0.02
B4DPH4	5.95E-01	NS	0.24	2.95E-01	NS	0.31	8.63E-01	NS	-0.07	4.49E-01	NS	0.17	7.33E-01	NS	-0.01
A0A140VKF3	7.86E-01	NS	0.23	1.19E-01	NS	-0.45	8.30E-02	NS	0.56	7.31E-01	NS	0.08	5.28E-01	NS	0.07
A0A024R9Q1	8.37E-01	NS	-0.22	3.69E-01	NS	0.37	1.51E-01	NS	0.63	2.66E-01	NS	0.75	1.15E-02	NS	0.29
A0A0G2JPA8	7.26E-01	NS	-0.08	1.87E-01	NS	-0.34	8.53E-02	NS	0.35	5.29E-01	NS	0.12	1.62E-01	NS	-0.09
H0YAC1	5.00E-01	NS	-0.09	3.09E-01	NS	0.46	3.91E-01	NS	-0.19	6.65E-01	NS	0.15	4.96E-01	NS	0.04
Q59GS8	9.81E-01	NS	0.07	9.24E-02	NS	-0.46	9.76E-01	NS	-0.07	8.24E-01	NS	0.04	4.85E-01	NS	-0.05
B3KT06	7.97E-01	NS	0.17	9.96E-01	NS	-0.39	3.06E-01	NS	-2.75	6.97E-01	NS	-1.71	2.04E-01	NS	-0.20
J3K000	9.22E-01	NS	0.19	3.34E-01	NS	-0.44	3.27E-01	NS	0.51	7.51E-01	NS	-0.22	2.46E-01	NS	0.14
D9YZU5	4.03E-01	NS	-0.33	3.16E-01	NS	-0.35	7.32E-02	NS	2.09	4.98E-01	NS	0.66	9.74E-03	NS	0.95
K7ERE3	4.17E-03	NS	1.20	8.21E-01	NS	0.95	2.30E-01	NS	-1.82	5.03E-01	NS	-1.07	7.46E-01	NS	-0.01
G3V2W1	3.73E-01	NS	0.15	1.22E-01	NS	-0.29	6.44E-01	NS	-0.19	2.34E-01	NS	0.20	8.15E-01	NS	0.02
Q9H2E1	5.31E-02	NS	-2.03	2.04E-01	NS	-2.37	9.28E-01	NS	-0.40	3.30E-01	NS	1.50	6.70E-01	NS	0.31
A0A1B0GUU9	1.91E-01	NS	1.00	1.84E-02	NS	-1.83	4.03E-01	NS	0.72	3.04E-01	NS	0.53	4.96E-01	NS	-0.16
D0PNI1	2.34E-01	NS	0.59	4.66E-01	NS	0.12	8.12E-02	NS	0.93	1.32E-01	NS	1.38	2.50E-05	NS	1.03
Q59EZ3	5.00E-01	NS	0.94	7.36E-01	NS	0.05	2.18E-01	NS	1.65	6.54E-01	NS	0.57	5.09E-01	NS	0.05
Q6LAN8	4.26E-01	NS	0.50	4.09E-01	NS	1.04	6.73E-01	NS	-0.37	7.05E-01	NS	0.36	2.54E-02	NS	0.53
D3JV41	6.26E-02	NS	0.41	4.36E-01	NS	-0.32	4.35E-02	NS	0.88	9.32E-01	NS	-0.06	1.05E-01	NS	0.18
F8W0J6	5.55E-01	NS	-0.29	8.76E-01	NS	0.28	8.19E-01	NS	-0.07	1.10E-01	NS	1.44	5.61E-01	NS	0.09
V9HWF6	8.44E-01	NS	-0.07	5.96E-01	NS	-1.49	6.44E-01	NS	-0.88	8.21E-01	NS	-2.30	9.91E-01	NS	-0.28
B4DZ36	3.66E-01	NS	-0.53	5.39E-01	NS	0.25	9.95E-01	NS	-0.05	6.70E-01	NS	-0.26	7.48E-01	NS	0.04
B4DPQ0	2.74E-02	NS	0.43	2.47E-01	NS	-0.31	5.88E-02	NS	0.42	4.92E-01	NS	0.17	1.61E-01	NS	0.09
B7Z549	8.00E-01	NS	-0.08	1.80E-01	NS	-0.43	7.52E-01	NS	0.00	9.05E-01	NS	-0.20	8.02E-01	NS	-0.04
B4DDT3	3.59E-01	NS	0.39	7.88E-02	NS	-0.43	3.43E-01	NS	0.23	8.88E-01	NS	0.09	6.14E-01	NS	-0.12
D3DPG0	7.94E-01	NS	0.17	2.30E-01	NS	-0.43	4.25E-01	NS	0.38	6.42E-01	NS	0.14	7.33E-01	NS	0.05
I3L3D5	4.98E-02	NS	1.15	6.15E-01	NS	-0.57	4.61E-01	NS	1.59	1.02E-02	NS	2.77	7.77E-05	NS	1.19
F8VV57	7.84E-02	NS	0.82	9.38E-01	NS	0.00	2.85E-01	NS	1.05	3.63E-01	NS	-0.48	6.03E-01	NS	-0.11
A0A182DWH7	1.92E-02	NS	0.65	5.57E-01	NS	0.16	5.63E-01	NS	0.12	1.88E-01	NS	0.40	3.31E-01	NS	-0.08
Q13784	3.28E-01	NS	0.50	7.71E-01	NS	-0.04	9.30E-01	NS	-0.15	4.68E-01	NS	0.47	1.08E-01	NS	0.30
Q9UNL6	6.48E-01	NS	0.15	3.52E-01	NS	-0.35	2.46E-02	NS	1.20	2.42E-01	NS	0.44	3.04E-02	NS	0.52
A0A0U1RR20	2.87E-01	NS	-0.71	1.25E-01	NS	-1.20	4.85E-01	NS	1.03	4.42E-01	NS	0.27	2.61E-01	NS	-0.09
A0A0S2Z3E8	7.67E-01	NS	-0.56	9.50E-01	NS	-0.46	5.15E-01	NS	-0.28	3.82E-01	NS	-2.37	4.37E-02	NS	-0.69
A0A024R6N9	2.47E-01	NS	0.36	4.06E-01	NS	-0.31	4.81E-02	NS	0.61	5.63E-01	NS	0.27	4.28E-01	NS	0.07
A0A0F7G8J1	7.39E-01	NS	0.12	3.62E-01	NS	0.15	6.85E-01	NS	0.11	8.82E-01	NS	-0.09	9.99E-01	NS	0.03
E9PHK0	2.73E-01	NS	0.29	9.94E-01	NS	0.07	5.50E-01	NS	0.14	2.25E-01	NS	0.31	2.35E-01	NS	0.09
Q59EA3	2.51E-01	NS	0.85	7.26E-02	NS	0.85	6.20E-01	NS	0.04	5.46E-01	NS	0.54	7.70E-02	NS	0.42
B2R773	3.83E-01	NS	0.51	8.99E-01	NS	-0.11	3.44E-01	NS	0.77	7.69E-01	NS	0.15	8.01E-01	NS	0.41
B0YJC6	1.46E-01	NS	-0.79	4.79E-01	NS	0.20	1.33E-01	NS	-0.43	8.35E-01	NS	-0.08	4.08E-01	NS	-0.07
B4E1Z4	2.07E-01	NS	-0.17	5.16E-01	NS	0.27	6.48E-01	NS	-0.11	7.11E-01	NS	-0.09	2.89E-01	NS	-0.04
V9HWP0	5.56E-01	NS	0.41	9.28E-02	NS	-0.71	5.29E-02	NS	0.90	2.14E-01	NS	0.49	8.60E-01	NS	0.01
A8K1K1	6.78E-01	NS	-0.44	3.59E-01	NS	-0.96	9.16E-01	NS	-0.01	9.47E-01	NS	-0.46	3.20E-01	NS	-0.21
A0A384NKS6	7.23E-01	NS	0.05	6.14E-02	NS	-0.71	2.24E-01	NS	0.38	5.30E-01	NS	0.20	6.71E-01	NS	-0.04
Q9UEU7	9.76E-02	NS	-0.78	3.59E-01	NS	0.61	2.05E-01	NS	-0.53	6.34E-01	NS	-0.45	7.22E-01	NS	0.00
Q499G5	9.33E-02	NS	-0.77	4.40E-01	NS	0.21	3.99E-01	NS	-0.48	8.83E-01	NS	0.04	7.75E-01	NS	-0.07
C9JV77	4.85E-01	NS	-0.62	6.57E-01	NS	0.20	1.23E-01	NS	-0.77	8.02E-01	NS	0.01	5.23E-01	NS	-0.14
Q53F26	6.72E-02	NS	-1.14	8.81E-02	NS	1.16	2.21E-01	NS	-0.60	7.25E-01	NS	0.18	5.71E-01	NS	-0.18
B2R950	8.09E-01	NS	0.21	4.15E-01	NS	-0.55	5.35E-01	NS	0.31	7.89E-01	NS	0.08	9.48E-01	NS	0.02
D6RAR4	7.36E-01	NS	0.12	9.09E-01	NS	0.26	8.38E-01	NS	-0.24	4.81E-01	NS	0.32	4.68E-01	NS	0.06
A0A024R104	8.39E-01	NS	0.04	5.44E-01	NS	0.21	7.81E-01	NS	-0.11	8.89E-01	NS	-0.05	2.04E-01	NS	0.12
A0A3G1HEF1	6.56E-01	NS	0.11	6.94E-01	NS	0.16	2.21E-01	NS	0.30	1.61E-01	NS	-0.65	1.14E-01	NS	-0.17
S4R3Y4	2.08E-01	NS	-0.52	9.95E-02	NS	1.15	5.32E-01	NS	0.13	5.26E-01	NS	0.14	2.89E-01	NS	0.03
B3KS79	2.53E-01	NS	0.41	5.02E-01	NS	0.22	8.23E-02	NS	0.50	3.82E-01	NS	0.19	2.69E-01	NS	0.07
Q8TAY0	9.91E-01	NS	0.13	1.68E-01	NS	-0.43	7.17E-01	NS	0.14	9.59E-01	NS	-0.17	7.40E-01	NS	0.04
M0R2W8	1.96E-01	NS	0.85	5.85E-02	NS	-1.71	2.22E-02	NS	1.24	1.56E-01	NS	0.54	9.53E-01	NS	0.02
A6XND1	2.75E-01	NS	0.38	3.06E-01	NS	0.50	5.29E-01	NS	-0.32	3.18E-01	NS	0.22	6.47E-01	NS	0.05
V9HWD8	6.75E-01	NS	-0.29	4.81E-01	NS	0.32	6.19E-01	NS	-0.37	4.77E-01	NS	0.25	9.90E-01	NS	-0.05
Q53H37	9.62E-01	NS	0.30	4.10E-01	NS	-0.17	3.29E-02	NS	3.26	1.06E-02	NS	-3.42	4.41E-01	NS	0.61
B7Z555	6.74E-01	NS	0.20	5.30E-01	NS	-0.10	4.28E-01	NS	0.25	6.78E-01	NS	-0.24	6.56E-01	NS	0.02
F5H1C6	3.14E-01	NS	-0.61	2.31E-01	NS	-0.83	7.78E-01	NS	-0.32	8.00E-01	NS	-0.11	5.72E-02	NS	-0.44
B2R6W1	4.16E-01	NS	0.23	6.21E-01	NS	-0.18	3.40E-02	NS	0.52	8.81E-01	NS	0.04	7.75E-01	NS	-0.03
Q5VY30	1.98E-01	NS	-0.76	1.88E-02	NS	0.76	8.57E-03	NS	-0.92	7.17E-01	NS	-0.07	8.62E-01	NS	0.00
A0A0S2Z4I5	6.77E-01	NS	0.03	6.21E-01	NS	0.12	2.27E-01	NS	0.54	1.13E-01	NS	0.60	2.53E-01	NS	0.05
A0A087WW43	5.58E-01	NS	0.11	8.65E-01	NS	-0.01	2.10E-01	NS	0.34	8.08E-01	NS	-0.09	5.97E-01	NS	-0.01
A0A384N669	1.93E-01	NS	0.42	8.41E-01	NS	0.12	8.70E-01	NS	0.03	5.11E-01	NS	0.30	7.32E-02	NS	0.17
A8K8Z4	1.91E-01	NS	0.28	2.31E-01	NS	0.14	1.38E-01	NS	0.28	7.24E-01	NS	0.08	4.71E-01	NS	0.05
A0A384MEF1	7.44E-01	NS	0.31	8.45E-01	NS	-0.08	5.57E-01	NS	0.24	8.99E-01	NS	-0.21	3.39E-01	NS	0.08
Q5SQ08	7.54E-01	NS	0.12	4.16E-01	NS	0.32	7.50E-01	NS	-0.01	8.76E-01	NS	-0.15	9.38E-01	NS	0.00
Q5J875	7.39E-01	NS	0.14	7.78E-01	NS	-0.08	7.88E-01	NS	-0.05	4.60E-01	NS	-0.32	3.47E-02	NS	-0.20
Q8IVC0	8.56E-01	NS	-0.09	1.50E-01	NS	-0.63	3.76E-01	NS	0.27	4.15E-01	NS	0.17	1.26E-01	NS	-0.13
J3KPA1	4.55E-01	NS	0.18	5.69E-01	NS	0.21	5.52E-01	NS	0.24	4.00E-01	NS	0.34	7.37E-01	NS	0.00
B2R7F8	9.23E-01	NS	-0.02	3.58E-02	NS	0.43	4.38E-01	NS	-0.18	3.52E-01	NS	0.29	6.59E-01	NS	0.04
A2RTY6	8.76E-01	NS	-0.05	3.16E-02	NS	-0.67	5.09E-01	NS	0.17	9.25E-01	NS	-0.17	4.85E-01	NS	-0.06
Q4LE79	9.75E-01	NS	0.05	6.40E-01	NS	0.20	3.18E-01	NS	-0.62	7.35E-01	NS	0.00	1.44E-01	NS	-0.40
RT-Cal protein	1.11E-01	NS	-0.16	4.39E-01	NS	0.11	7.78E-01	NS	0.03	9.57E-01	NS	0.00	7.92E-01	NS	-0.01
E7END6	3.41E-01	NS	-0.22	2.99E-01	NS	0.22	3.30E-02	NS	-0.63	2.94E-01	NS	0.36	3.39E-01	NS	-0.06
D9IWP9	8.20E-01	NS	0.05	4.69E-01	NS	0.35	5.53E-01	NS	-0.31	6.35E-01	NS	0.17	2.57E-01	NS	-0.07
B2R815	6.39E-01	NS	0.18	1.92E-01	NS	-0.56	1.68E-01	NS	0.50	9.49E-01	NS	-0.10	6.93E-01	NS	-0.05
V9GYM3	6.93E-01	NS	0.10	2.00E-01	NS	-2.69	3.33E-02	NS	3.24	3.87E-01	NS	1.37	1.97E-01	NS	0.37
D6R934	1.61E-01	NS	0.80	2.92E-01	NS	-0.45	3.31E-02	NS	0.79	2.48E-01	NS	0.48	5.36E-01	NS	0.10
A0A087X1J7	6.40E-01	NS	0.27	2.65E-01	NS	-0.39	2.24E-01	NS	0.43	5.83E-01	NS	0.19	8.54E-01	NS	0.00
E9PIT3	1.62E-01	NS	-0.71	2.07E-02	NS	1.36	3.14E-02	NS	-1.43	1.00E+00	NS	0.18	5.36E-01	NS	0.12
A0A024R035	4.09E-01	NS	0.14	8.96E-01	NS	-0.11	5.28E-01	NS	0.21	9.89E-01	NS	0.02	7.61E-01	NS	0.03
K7EQQ3	1.61E-01	NS	1.24	5.83E-01	NS	-0.10	5.73E-01	NS	-0.23	7.61E-01	NS	-0.30	8.13E-01	NS	0.02
A0A140VK00	7.24E-02	NS	0.59	4.46E-01	NS	0.23	6.08E-01	NS	-0.01	6.20E-01	NS	0.20	3.27E-01	NS	0.09
V9HWE3	8.31E-01	NS	0.11	4.17E-01	NS	0.28	2.02E-01	NS	1.13	6.11E-01	NS	0.41	2.41E-01	NS	0.24
Q53GN8	4.11E-01	NS	-0.66	8.59E-01	NS	0.50	5.95E-01	NS	-0.20	8.73E-01	NS	0.21	4.90E-01	NS	-0.08
B4E1C2	5.88E-01	NS	0.24	7.00E-01	NS	0.09	8.45E-01	NS	-0.15	4.60E-02	NS	0.38	6.51E-01	NS	-0.06
J3QR68	9.43E-01	NS	-0.37	6.59E-01	NS	-0.20	8.89E-01	NS	-0.02	7.15E-01	NS	-0.58	3.24E-01	NS	-0.37
B0YIW2	1.77E-01	NS	-0.80	1.28E-01	NS	-1.19	9.40E-01	NS	-0.12	4.81E-01	NS	-0.18	7.42E-01	NS	-0.17
A0A1K0GXZ1	3.90E-01	NS	-0.41	6.53E-01	NS	0.09	9.28E-02	NS	1.80	2.55E-01	NS	0.94	1.21E-02	NS	0.95

[a] Numbers are FDR adjusted *P* values. NS = not significant (FDR, *P* value > 0.05).

Supplementary Table 4.

	0.25 h/Pre				0.5 h/Pre				1 h/Pre				2 h/Pre				4 h/Pre				8 h/Pre				16 h/Pre				1 d/Pre				2 d/Pre				3 d/Pre				7 d/Pre				14 d/Pre				21 d/Pre			
**Uniprot Symbol**	*p-value*	*FDR*		*log2(FC)*	*p-value*	*FDR*		*log2(FC)*	*p-value*	*FDR*		*log2(FC)*	*p-value*	*FDR*		*log2(FC)*	*p-value*	*FDR*		*log2(FC)*	*p-value*	*FDR*		*log2(FC)*	*p-value*	*FDR*		*log2(FC)*	*p-value*	*FDR*		*log2(FC)*	*p-value*	*FDR*		*log2(FC)*	*p-value*	*FDR*		*log2(FC)*	*p-value*	*FDR*		*log2(FC)*	*p-value*	*FDR*		*log2(FC)*	*p-value*	*FDR*		*log2(FC)*
X6RJP6	1.01E-03	NS	↑	3.40	5.99E-04	NS	↑	3.76	3.17E-04	NS	↑	3.75	1.49E-03	NS	↑	2.85	5.54E-01	NS	↑	0.80	8.50E-01	NS	↑	0.24	8.27E-01	NS	↑	0.58	6.43E-02	NS	↑	2.67	3.59E-02	NS	↑	1.83	9.27E-01	NS	↓	-0.17	9.12E-01	NS	↑	0.06	4.01E-02	NS	↑	1.20	2.79E-02	NS	↑	2.36
Q53GK6	6.38E-03	NS	↑	2.33	1.90E-03	NS	↑	3.25	8.25E-04	NS	↑	3.40	1.09E-02	NS	↑	1.57	1.78E-01	NS	↑	0.52	3.98E-02	NS	↑	1.05	1.63E-01	NS	↑	1.09	2.49E-02	NS	↑	2.37	2.79E-01	NS	↑	0.93	9.17E-01	NS	↑	0.01	4.12E-01	NS	↓	-0.58	7.33E-01	NS	↓	-0.01	8.59E-02	NS	↑	1.36
I3L3D5	1.93E-03	NS	↑	3.21	2.29E-03	NS	↑	3.33	8.35E-04	NS	↑	3.35	7.53E-03	NS	↑	2.62	6.69E-02	NS	↑	0.97	2.04E-01	NS	↑	0.87	9.74E-02	NS	↑	0.99	4.09E-02	NS	↑	2.14	2.21E-01	NS	↑	1.05	7.77E-01	NS	↓	-0.42	7.62E-01	NS	↓	-0.47	5.88E-01	NS	↓	-0.11	6.55E-02	NS	↑	1.96
B3KT06	1.96E-04	NS	↑	3.01	2.57E-03	NS	↑	2.88	4.87E-05	NS	↑	3.29	8.47E-02	NS	↑	3.13	2.34E-02	NS	↑	0.66	2.72E-02	NS	↑	3.01	2.34E-01	NS	↑	2.25	6.63E-02	NS	↑	3.69	4.47E-01	NS	↑	1.32	1.99E-01	NS	↓	-0.41	4.55E-01	NS	↑	2.70	9.19E-01	NS	↑	0.14	9.77E-01	NS	↑	0.78
V9HWN8	1.06E-01	NS	↑	0.54	5.42E-03	NS	↑	1.18	7.61E-03	NS	↑	1.01	3.11E-01	NS	↑	0.70	5.19E-02	NS	↑	0.76	6.43E-03	NS	↑	1.18	3.51E-02	NS	↑	0.91	6.58E-02	NS	↑	0.74	4.61E-01	NS	↑	0.69	4.31E-02	NS	↑	0.99	1.62E-02	NS	↑	1.01	1.96E-02	NS	↑	0.96	2.43E-02	NS	↑	1.21
F8W876	1.60E-01	NS	↑	0.56	4.66E-02	NS	↑	0.68	2.21E-02	NS	↑	0.77	2.40E-01	NS	↑	0.48	2.45E-02	NS	↑	0.42	6.15E-02	NS	↑	0.52	4.26E-02	NS	↑	0.61	6.99E-01	NS	↑	0.36	6.67E-01	NS	↑	0.20	1.99E-01	NS	↑	0.43	5.31E-02	NS	↑	0.50	2.78E-01	NS	↑	0.32	5.28E-02	NS	↑	0.50
B4E3P1	5.64E-03	NS	↑	3.02	1.07E-02	NS	↑	2.45	4.47E-03	NS	↑	2.57	3.22E-02	NS	↑	2.69	1.55E-01	NS	↑	0.68	2.11E-01	NS	↑	0.61	3.80E-01	NS	↑	0.56	5.55E-02	NS	↑	2.34	2.24E-01	NS	↑	0.63	9.18E-01	NS	↓	-0.50	3.13E-01	NS	↓	-1.25	9.31E-01	NS	↓	-0.39	8.61E-02	NS	↑	1.29
A0A0S2Z3D5	9.54E-03	NS	↑	1.99	1.51E-02	NS	↑	1.50	1.14E-01	NS	↑	1.60	1.85E-01	NS	↑	1.98	8.78E-01	NS	↑	0.92	4.84E-03	NS	↑	2.50	1.40E-02	NS	↑	1.50	2.09E-03	NS	↑	2.87	4.45E-03	NS	↑	1.86	1.67E-03	NS	↑	2.39	3.06E-03	NS	↑	2.61	5.33E-02	NS	↑	1.45	3.55E-01	NS	↑	1.45
A5PL27	4.38E-02	NS	↑	0.96	7.59E-03	NS	↑	1.65	3.74E-03	NS	↑	1.76	5.48E-01	NS	↑	0.31	2.02E-01	NS	↑	1.02	3.27E-02	NS	↑	1.52	1.40E-01	NS	↑	1.29	2.78E-02	NS	↑	1.50	4.69E-01	NS	↑	0.67	6.65E-02	NS	↑	1.20	2.71E-02	NS	↑	1.31	3.42E-02	NS	↑	1.46	1.12E-01	NS	↑	0.80
A0A182DWH7	2.06E-01	NS	↓	-0.26	8.40E-02	NS	↓	-0.58	2.13E-01	NS	↓	-0.61	3.16E-01	NS	↓	-0.27	9.03E-02	NS	↓	-0.38	6.21E-02	NS	↓	-0.56	9.45E-02	NS	↓	-0.49	1.85E-01	NS	↓	-0.40	4.25E-01	NS	↓	-0.20	4.84E-01	NS	↓	-0.10	7.83E-02	NS	↓	-0.40	2.57E-01	NS	↓	-0.20	3.92E-01	NS	↓	-0.15
F2RM35	4.42E-01	NS	↓	-0.24	1.19E-01	NS	↓	-0.49	7.08E-02	NS	↓	-0.47	9.29E-01	NS	↓	0.00	5.91E-01	NS	↓	-0.15	1.70E-01	NS	↓	-0.49	8.51E-01	NS	↑	0.14	9.67E-01	NS	↑	0.02	2.78E-01	NS	↓	-0.31	5.83E-01	NS	↓	-0.15	5.44E-01	NS	↓	-0.17	3.89E-01	NS	↓	-0.26	3.39E-01	NS	↓	-0.36
V9HWE9	3.69E-02	NS	↑	1.34	1.29E-02	NS	↑	1.67	4.42E-02	NS	↑	1.12	1.18E-01	NS	↑	1.08	1.76E-01	NS	↑	0.65	9.77E-01	NS	↓	-0.04	9.16E-01	NS	↑	0.51	1.98E-01	NS	↑	1.04	5.77E-01	NS	↓	-0.46	4.55E-01	NS	↓	-0.59	3.58E-01	NS	↓	-0.49	3.71E-01	NS	↑	0.48	3.08E-01	NS	↑	0.49
D0PNI1	3.03E-02	NS	↑	1.95	7.15E-02	NS	↑	1.75	1.35E-01	NS	↑	1.29	1.01E-01	NS	↑	1.35	2.19E-01	NS	↑	0.63	2.84E-01	NS	↑	0.46	3.07E-01	NS	↓	-1.17	1.82E-01	NS	↑	1.39	9.41E-01	NS	↓	-0.46	3.85E-01	NS	↓	-1.20	7.84E-01	NS	↓	-0.26	7.35E-01	NS	↓	-0.13	5.10E-01	NS	↑	0.53
B4DRT3	8.34E-01	NS	↑	0.23	4.51E-02	NS	↑	0.74	3.08E-01	NS	↑	0.76	2.68E-01	NS	↑	0.82	1.05E-03	NS	↑	0.92	1.95E-04	NS	↑	1.98	1.14E-01	NS	↑	1.69	4.87E-03	NS	↑	2.33	4.18E-02	NS	↑	1.42	1.03E-01	NS	↑	1.09	8.96E-01	NS	↑	0.52	8.19E-02	NS	↓	-0.47	4.42E-01	NS	↓	-0.19
Q9H2E1	3.61E-01	NS	↑	1.61	4.34E-01	NS	↑	0.01	7.14E-01	NS	↓	-0.67	1.74E-01	NS	↑	0.30	3.64E-01	NS	↑	1.81	8.35E-01	NS	↓	-0.61	9.55E-01	NS	↓	-0.35	2.72E-01	NS	↑	0.61	8.57E-01	NS	↓	-0.73	7.49E-01	NS	↓	-1.59	8.73E-01	NS	↓	-0.60	4.36E-01	NS	↓	-0.02	3.18E-01	NS	↑	0.82
H3BRJ9	3.94E-01	NS	↑	0.50	7.06E-02	NS	↑	1.64	2.45E-01	NS	↑	0.64	6.26E-01	NS	↑	0.53	2.57E-01	NS	↑	0.77	6.94E-02	NS	↑	1.48	8.20E-02	NS	↑	1.49	3.42E-01	NS	↑	0.76	8.75E-01	NS	↑	0.67	3.69E-01	NS	↑	1.51	2.39E-01	NS	↑	0.73	2.48E-01	NS	↑	1.59	1.44E-01	NS	↑	1.60
B4E1C2	7.67E-01	NS	↑	0.06	1.26E-01	NS	↓	-0.44	1.33E-01	NS	↓	-0.37	6.90E-01	NS	↓	-0.05	6.18E-01	NS	↓	-0.09	9.11E-02	NS	↓	-0.37	2.23E-01	NS	↓	-0.29	3.49E-01	NS	↓	-0.29	4.02E-01	NS	↓	-0.17	2.27E-01	NS	↓	-0.33	1.56E-01	NS	↓	-0.31	7.84E-02	NS	↓	-0.36	8.13E-01	NS	↓	-0.04
B4DVE1	7.18E-01	NS	↓	-0.11	2.33E-01	NS	↑	0.40	5.72E-02	NS	↑	0.71	2.57E-01	NS	↑	0.35	7.76E-01	NS	↓	-0.08	1.74E-01	NS	↑	0.63	6.00E-01	NS	↑	0.57	9.78E-01	NS	↓	-0.01	8.60E-01	NS	↑	0.03	2.76E-01	NS	↑	0.49	6.06E-03	NS	↑	0.93	4.60E-01	NS	↑	0.43	2.88E-01	NS	↑	0.57
V9HVY1	8.14E-01	NS	↑	0.24	9.65E-01	NS	↑	0.71	8.21E-01	NS	↓	-0.15	2.04E-01	NS	↑	0.63	9.25E-01	NS	↓	-0.05	9.57E-01	NS	↑	0.34	8.36E-01	NS	↑	0.20	7.71E-01	NS	↑	0.10	9.09E-01	NS	↑	0.19	7.03E-01	NS	↑	0.11	3.46E-01	NS	↑	0.45	7.11E-01	NS	↑	0.14	8.90E-01	NS	↓	-0.06
F8W0J6	2.53E-01	NS	↑	0.58	9.38E-01	NS	↑	0.45	2.28E-01	NS	↑	0.44	2.44E-01	NS	↑	0.83	8.65E-01	NS	↑	0.19	8.48E-01	NS	↑	0.74	3.51E-01	NS	↑	0.41	1.07E-01	NS	↑	0.78	4.10E-01	NS	↑	0.70	9.77E-01	NS	↑	0.29	5.32E-01	NS	↑	0.55	3.50E-01	NS	↑	0.58	1.74E-01	NS	↑	1.25
E3UN46	6.19E-01	NS	↓	-0.45	1.24E-01	NS	↓	-1.05	2.07E-01	NS	↓	-0.87	9.31E-01	NS	↓	-0.16	3.39E-01	NS	↓	-0.69	5.42E-01	NS	↓	-0.50	2.79E-01	NS	↓	-0.76	5.30E-01	NS	↓	-0.52	6.20E-01	NS	↓	-0.28	4.88E-01	NS	↓	-0.53	6.51E-01	NS	↓	-0.42	4.06E-01	NS	↓	-0.54	4.89E-01	NS	↓	-0.54
B2RA39	5.00E-01	NS	↓	-1.79	1.65E-01	NS	↓	-2.59	3.17E-01	NS	↓	-2.14	8.28E-01	NS	↓	-0.25	2.67E-01	NS	↓	-2.25	5.75E-01	NS	↓	-0.04	7.49E-01	NS	↓	-1.22	7.53E-01	NS	↓	-1.27	8.90E-01	NS	↓	-0.18	5.75E-01	NS	↓	-1.42	9.52E-01	NS	↓	-0.82	5.25E-01	NS	↓	-1.37	2.48E-01	NS	↓	-2.31
V9HW98	8.79E-02	NS	↑	0.92	2.86E-01	NS	↑	0.46	2.49E-01	NS	↑	0.44	6.16E-01	NS	↑	0.20	3.81E-01	NS	↑	0.71	6.22E-01	NS	↑	0.13	3.88E-01	NS	↑	0.66	2.05E-01	NS	↑	0.70	3.17E-01	NS	↑	0.45	7.70E-01	NS	↓	-0.14	5.13E-01	NS	↓	-0.39	7.12E-01	NS	↑	0.04	6.22E-01	NS	↓	-0.20
Q53H26	8.25E-01	NS	↑	0.19	1.25E-01	NS	↓	-1.01	1.43E-01	NS	↓	-1.03	2.61E-01	NS	↓	-0.63	9.46E-01	NS	↑	0.53	3.38E-01	NS	↓	-0.47	6.20E-01	NS	↓	-0.11	5.98E-01	NS	↑	0.94	6.68E-01	NS	↑	0.77	2.96E-01	NS	↑	0.52	6.22E-01	NS	↓	-0.30	4.22E-01	NS	↑	0.41	7.36E-01	NS	↑	0.23
B4DDT3	9.16E-01	NS	↓	-0.05	7.27E-01	NS	↑	0.25	2.38E-01	NS	↑	0.47	7.23E-01	NS	↓	-0.11	3.48E-01	NS	↓	-0.33	2.02E-01	NS	↑	0.56	7.95E-01	NS	↑	0.30	3.84E-01	NS	↓	-0.29	7.77E-01	NS	↑	0.05	6.61E-01	NS	↑	0.19	3.80E-01	NS	↓	-0.29	1.49E-01	NS	↑	0.41	7.85E-01	NS	↑	0.15
B7ZKJ8	1.61E-01	NS	↑	0.61	9.44E-02	NS	↑	0.64	9.16E-02	NS	↑	0.58	2.43E-01	NS	↑	0.43	2.10E-01	NS	↑	0.49	1.89E-01	NS	↑	0.45	1.95E-01	NS	↑	0.41	4.06E-01	NS	↑	0.35	7.92E-01	NS	↑	0.15	2.67E-01	NS	↑	0.36	5.53E-02	NS	↑	0.65	1.99E-01	NS	↑	0.47	1.53E-01	NS	↑	0.46
Q53H37	4.42E-01	NS	↓	-0.01	2.79E-01	NS	↓	-0.74	2.96E-01	NS	↓	-0.63	7.36E-01	NS	↑	0.58	6.04E-01	NS	↑	0.03	5.27E-01	NS	↑	1.44	6.83E-01	NS	↑	0.41	3.85E-01	NS	↑	1.98	9.92E-01	NS	↑	0.21	1.29E-01	NS	↓	-1.13	2.03E-01	NS	↓	-0.59	1.79E-01	NS	↓	-0.91	7.38E-03	NS	↓	-2.76
A0A2S0BDD1	6.98E-01	NS	↑	0.11	2.05E-01	NS	↑	0.47	4.30E-01	NS	↑	0.21	6.31E-01	NS	↑	0.20	3.31E-01	NS	↑	0.22	1.80E-02	NS	↑	0.82	1.27E-01	NS	↑	0.45	2.73E-01	NS	↑	0.36	9.69E-01	NS	↑	0.01	6.60E-02	NS	↑	0.67	3.83E-03	NS	↑	0.97	1.02E-01	NS	↑	0.36	1.57E-01	NS	↑	0.47
L8E853	8.88E-01	NS	↑	0.14	5.36E-02	NS	↑	0.85	6.26E-02	NS	↑	0.94	4.37E-02	NS	↑	0.90	2.51E-01	NS	↑	0.69	8.53E-02	NS	↑	1.11	2.65E-01	NS	↑	0.64	4.86E-02	NS	↑	0.84	2.04E-01	NS	↑	0.53	3.08E-01	NS	↑	0.32	1.41E-01	NS	↑	0.61	3.84E-01	NS	↑	0.23	6.76E-02	NS	↑	0.65
B7Z555	8.44E-01	NS	↓	-0.06	2.33E-01	NS	↑	0.37	9.27E-02	NS	↑	0.45	7.47E-01	NS	↓	-0.09	9.72E-01	NS	↓	-0.03	9.60E-02	NS	↑	0.42	5.51E-01	NS	↑	0.36	6.45E-01	NS	↑	0.14	7.47E-01	NS	↑	0.18	4.53E-01	NS	↑	0.34	5.08E-01	NS	↑	0.26	1.69E-01	NS	↑	0.39	8.18E-01	NS	↑	0.08
H0YID6	5.03E-02	NS	↑	2.62	1.30E-01	NS	↑	2.45	9.13E-02	NS	↑	2.04	3.97E-01	NS	↑	2.45	4.74E-01	NS	↑	1.71	8.26E-02	NS	↑	2.83	1.39E-01	NS	↑	2.77	4.88E-01	NS	↑	1.92	2.23E-01	NS	↑	2.93	1.31E-01	NS	↑	2.01	5.62E-01	NS	↓	-0.27	5.28E-02	NS	↑	2.29	1.75E-02	NS	↑	2.69
A0A0S2Z3E8	3.93E-01	NS	↓	-1.65	4.81E-01	NS	↓	-1.01	5.15E-01	NS	↓	-0.99	8.79E-01	NS	↓	-0.67	4.15E-01	NS	↑	0.05	9.32E-01	NS	↓	-0.53	8.72E-01	NS	↓	-0.72	9.91E-01	NS	↓	-0.59	8.09E-01	NS	↓	-0.70	6.05E-01	NS	↑	0.30	5.61E-01	NS	↑	0.27	6.64E-01	NS	↓	-0.19	5.12E-01	NS	↓	-1.21
A0A024R104	2.41E-01	NS	↑	0.82	9.33E-01	NS	↑	0.03	2.11E-01	NS	↓	-0.37	4.36E-01	NS	↑	0.16	4.41E-01	NS	↓	-0.27	4.01E-01	NS	↓	-0.30	1.08E-01	NS	↓	-0.49	1.32E-01	NS	↓	-0.61	1.97E-01	NS	↓	-0.41	5.10E-01	NS	↓	-0.20	8.94E-01	NS	↓	-0.03	6.46E-01	NS	↓	-0.12	5.57E-01	NS	↓	-0.18
A0A087WSY5	6.13E-01	NS	↑	0.15	8.62E-01	NS	↑	0.17	2.98E-01	NS	↑	0.35	5.06E-01	NS	↑	0.15	2.28E-01	NS	↑	0.35	2.57E-01	NS	↑	0.32	5.06E-01	NS	↑	0.18	4.91E-01	NS	↑	0.19	3.61E-01	NS	↑	0.31	3.66E-01	NS	↑	0.31	4.35E-01	NS	↑	0.24	2.02E-01	NS	↑	0.39	3.03E-01	NS	↑	0.36
E9PHK0	7.04E-01	NS	↑	0.13	2.69E-01	NS	↓	-0.42	2.46E-01	NS	↓	-0.42	6.29E-01	NS	↓	-0.18	4.73E-01	NS	↑	0.35	9.11E-01	NS	↓	-0.07	9.64E-01	NS	↑	0.13	8.27E-01	NS	↓	-0.09	6.63E-01	NS	↓	-0.16	6.10E-01	NS	↓	-0.19	4.96E-01	NS	↓	-0.25	6.58E-01	NS	↓	-0.17	9.98E-01	NS	↓	-0.04
D3DNN4	6.85E-01	NS	↑	0.10	2.09E-01	NS	↑	1.48	9.19E-02	NS	↑	1.07	8.65E-01	NS	↑	0.70	6.12E-01	NS	↑	0.09	3.01E-01	NS	↑	1.07	4.38E-01	NS	↑	1.17	5.97E-01	NS	↑	1.21	5.59E-01	NS	↑	0.22	2.07E-01	NS	↑	1.20	2.27E-01	NS	↑	0.50	1.45E-01	NS	↑	0.99	2.91E-01	NS	↑	0.53
A0A2Z4LCH4	5.07E-01	NS	↑	0.25	1.74E-01	NS	↑	0.82	1.72E-01	NS	↑	0.57	8.34E-01	NS	↑	0.14	4.69E-01	NS	↑	0.23	2.64E-01	NS	↑	0.39	3.65E-01	NS	↑	0.42	3.80E-01	NS	↑	0.39	8.57E-01	NS	↑	0.13	3.98E-01	NS	↑	0.56	2.46E-01	NS	↑	0.48	1.50E-01	NS	↑	0.69	1.92E-01	NS	↑	0.86
A0A3G1HEF1	4.71E-02	NS	↑	0.39	9.95E-02	NS	↑	0.54	3.71E-01	NS	↑	0.33	9.15E-01	NS	↑	0.04	6.40E-01	NS	↑	0.19	9.95E-02	NS	↑	0.51	8.43E-01	NS	↑	0.18	4.91E-02	NS	↑	0.56	1.74E-01	NS	↑	0.26	1.27E-01	NS	↑	0.47	5.22E-02	NS	↑	0.73	5.95E-02	NS	↑	0.55	8.45E-01	NS	↑	0.02
A0A140VK00	4.35E-01	NS	↑	0.16	5.09E-01	NS	↓	-0.20	2.60E-01	NS	↓	-0.42	2.93E-01	NS	↓	-0.32	5.93E-01	NS	↓	-0.18	4.14E-01	NS	↓	-0.30	7.40E-01	NS	↓	0.00	5.92E-01	NS	↓	-0.18	9.19E-01	NS	↑	0.05	8.62E-01	NS	↑	0.01	3.74E-01	NS	↓	-0.30	5.39E-01	NS	↓	-0.21	6.64E-01	NS	↓	-0.13
I3L145	4.07E-01	NS	↓	-0.48	2.66E-01	NS	↓	-0.67	4.81E-01	NS	↓	-0.44	8.46E-02	NS	↓	-1.04	1.67E-01	NS	↓	-0.81	5.90E-01	NS	↓	-0.35	5.57E-01	NS	↓	-0.37	1.56E-01	NS	↓	-0.81	3.47E-01	NS	↓	-0.57	4.91E-01	NS	↓	-0.43	2.16E-01	NS	↓	-0.76	8.38E-01	NS	↓	-0.20	6.05E-01	NS	↓	-0.36
B7ZLE5	5.65E-01	NS	↓	-0.53	2.43E-01	NS	↓	-0.89	3.58E-01	NS	↓	-0.72	3.70E-01	NS	↓	-0.71	3.32E-01	NS	↓	-0.75	5.51E-01	NS	↑	0.18	2.70E-01	NS	↓	-0.86	3.54E-01	NS	↓	-0.59	4.72E-01	NS	↓	-0.52	2.78E-01	NS	↓	-0.82	6.92E-01	NS	↓	-0.43	1.31E-01	NS	↓	-1.09	2.57E-01	NS	↓	-0.85
A0A0U1RR20	2.00E-01	NS	↓	-0.51	9.15E-01	NS	↑	0.06	9.04E-01	NS	↑	0.18	2.33E-01	NS	↓	-0.39	4.10E-01	NS	↓	-0.29	4.39E-01	NS	↑	0.18	2.80E-01	NS	↑	0.26	4.83E-01	NS	↓	-0.17	3.94E-01	NS	↓	-0.24	1.61E-01	NS	↓	-1.08	1.70E-01	NS	↓	-0.89	2.72E-01	NS	↓	-0.51	3.18E-01	NS	↓	-0.30
A0A024R9Q1	2.35E-01	NS	↑	0.46	7.30E-01	NS	↑	0.30	3.91E-01	NS	↑	0.44	6.53E-01	NS	↑	0.23	8.27E-01	NS	↓	-0.21	6.21E-01	NS	↓	-0.30	5.69E-01	NS	↓	-0.30	5.44E-01	NS	↑	0.33	2.62E-01	NS	↓	-0.61	3.23E-01	NS	↓	-0.53	6.36E-01	NS	↓	-0.28	5.86E-01	NS	↑	0.22	2.50E-01	NS	↑	0.75
Q53F26	2.41E-01	NS	↓	-0.63	5.12E-01	NS	↓	-0.44	8.48E-01	NS	↓	-0.02	7.88E-01	NS	↑	0.11	1.21E-01	NS	↑	0.67	3.70E-01	NS	↑	0.77	7.56E-01	NS	↓	-0.03	6.01E-01	NS	↑	0.32	7.78E-01	NS	↓	-0.25	7.10E-01	NS	↓	-0.32	8.65E-02	NS	↑	1.01	3.86E-01	NS	↑	0.47	5.82E-01	NS	↑	0.37
A0A3B3IS66	7.06E-01	NS	↑	0.07	3.39E-01	NS	↓	-0.38	3.16E-01	NS	↓	-0.40	9.19E-01	NS	↓	-0.05	8.86E-01	NS	↑	0.01	2.01E-01	NS	↓	-0.50	2.66E-01	NS	↓	-0.51	5.58E-01	NS	↓	-0.25	3.91E-01	NS	↓	-0.36	2.49E-01	NS	↓	-0.49	1.69E-01	NS	↓	-0.74	9.53E-02	NS	↓	-0.63	1.87E-01	NS	↓	-0.51
C9JEU5	2.87E-01	NS	↓	-0.66	8.13E-01	NS	↑	0.02	2.89E-01	NS	↓	-0.77	2.80E-01	NS	↓	-0.65	5.60E-01	NS	↓	-0.42	9.69E-01	NS	↑	0.16	6.69E-01	NS	↓	-0.27	8.55E-01	NS	↑	0.18	3.60E-01	NS	↑	0.34	6.21E-01	NS	↓	-0.38	9.48E-01	NS	↑	0.22	1.91E-01	NS	↓	-1.04	4.56E-01	NS	↓	-0.47
V9HWE3	2.05E-01	NS	↑	0.48	4.84E-02	NS	↑	1.73	5.06E-02	NS	↑	0.52	4.92E-01	NS	↑	0.34	1.38E-01	NS	↑	1.04	1.65E-01	NS	↑	0.46	9.05E-01	NS	↑	0.37	8.12E-02	NS	↓	-0.53	7.16E-01	NS	↑	0.80	9.48E-01	NS	↑	0.35	7.72E-01	NS	↓	-0.01	4.31E-01	NS	↑	0.80	4.44E-01	NS	↑	0.68
Q5J875	5.26E-01	NS	↑	0.26	4.99E-01	NS	↓	-0.29	5.73E-01	NS	↓	-0.26	5.91E-01	NS	↓	-0.23	8.68E-01	NS	↓	-0.02	4.44E-01	NS	↓	-0.33	5.53E-01	NS	↓	-0.23	4.97E-01	NS	↓	-0.30	1.78E-01	NS	↓	-0.63	4.07E-01	NS	↓	-0.38	4.92E-01	NS	↓	-0.29	7.96E-01	NS	↑	0.05	9.68E-01	NS	↓	-0.03
Q499G5	2.90E-01	NS	↓	-0.57	4.24E-01	NS	↑	0.49	3.23E-01	NS	↑	0.24	2.11E-01	NS	↓	-0.48	8.56E-01	NS	↑	0.13	3.27E-01	NS	↑	0.24	4.89E-01	NS	↓	-0.25	9.07E-01	NS	↓	-0.08	2.86E-01	NS	↓	-0.60	4.60E-01	NS	↓	-0.26	6.31E-01	NS	↑	0.07	5.16E-01	NS	↓	-0.13	6.99E-01	NS	↑	0.18
B2R888	5.26E-01	NS	↓	-0.14	3.89E-01	NS	↓	-0.16	7.53E-01	NS	↑	0.05	7.48E-02	NS	↓	-0.54	3.61E-01	NS	↓	-0.17	5.16E-01	NS	↑	0.16	7.43E-01	NS	↑	0.18	8.19E-01	NS	↓	-0.04	9.83E-01	NS	↑	0.02	3.85E-01	NS	↑	0.19	7.68E-02	NS	↓	-0.39	2.72E-01	NS	↓	-0.34	1.91E-02	NS	↓	-0.50
J3QR68	5.24E-01	NS	↑	0.05	6.54E-01	NS	↑	0.35	2.41E-01	NS	↓	-0.85	3.10E-01	NS	↓	-0.74	4.03E-01	NS	↓	-0.49	6.55E-02	NS	↓	-1.83	1.17E-01	NS	↓	-1.08	5.51E-01	NS	↓	-0.13	5.36E-01	NS	↑	0.12	4.51E-01	NS	↓	-0.34	3.13E-01	NS	↓	-0.55	2.66E-01	NS	↓	-0.75	4.99E-01	NS	↓	-0.40
A0A024R962	2.39E-01	NS	↑	1.18	2.54E-01	NS	↑	0.73	4.72E-01	NS	↑	0.62	1.05E-01	NS	↑	1.12	7.24E-02	NS	↑	1.03	2.80E-01	NS	↑	0.37	6.78E-01	NS	↑	0.21	5.69E-02	NS	↑	1.21	7.66E-01	NS	↓	-0.34	6.73E-01	NS	↑	0.25	8.77E-02	NS	↑	1.30	7.91E-01	NS	↑	0.01	3.35E-01	NS	↑	0.65
E7END6	4.75E-01	NS	↑	0.15	5.01E-01	NS	↓	-0.17	2.86E-01	NS	↓	-0.32	8.42E-01	NS	↓	-0.04	8.62E-01	NS	↓	-0.04	6.59E-02	NS	↓	-0.45	2.91E-01	NS	↓	-0.30	5.23E-01	NS	↓	-0.18	3.74E-01	NS	↓	-0.29	2.75E-01	NS	↓	-0.34	5.64E-01	NS	↓	-0.15	8.89E-02	NS	↓	-0.42	8.99E-01	NS	↑	0.06
A0A384MDV8	5.53E-01	NS	↓	-0.09	2.11E-01	NS	↓	-0.37	2.41E-01	NS	↓	-0.45	9.75E-02	NS	↓	-0.42	1.55E-01	NS	↓	-0.20	6.91E-01	NS	↓	-0.02	7.35E-02	NS	↓	-0.42	7.64E-01	NS	↑	0.11	9.04E-01	NS	↓	-0.01	9.31E-01	NS	↑	0.10	1.72E-01	NS	↓	-0.27	2.50E-01	NS	↑	0.24	9.76E-01	NS	↑	0.03
A0A3B3ISJ1	7.86E-01	NS	↓	-0.03	6.20E-01	NS	↑	0.09	1.92E-01	NS	↑	0.16	9.57E-01	NS	↑	0.04	7.00E-01	NS	↑	0.09	6.21E-01	NS	↓	-0.06	6.66E-01	NS	↑	0.07	2.19E-01	NS	↓	-0.14	1.59E-01	NS	↓	-0.29	2.70E-01	NS	↓	-0.14	2.08E-01	NS	↓	-0.24	4.35E-02	NS	↓	-0.30	2.14E-02	NS	↓	-0.30
A0A0G2JPA8	1.88E-01	NS	↓	-0.31	4.26E-01	NS	↓	-0.18	6.14E-02	NS	↓	-0.57	7.09E-02	NS	↓	-0.46	1.77E-01	NS	↓	-0.30	7.23E-01	NS	↓	-0.09	7.09E-01	NS	↓	-0.08	1.10E-01	NS	↓	-0.42	2.34E-01	NS	↓	-0.29	8.53E-01	NS	↓	-0.04	2.05E-01	NS	↓	-0.27	4.51E-01	NS	↑	0.17	4.10E-01	NS	↑	0.14
B4DPH4	7.07E-01	NS	↑	0.06	9.43E-01	NS	↑	0.05	3.60E-01	NS	↑	0.37	2.36E-01	NS	↑	0.22	9.18E-01	NS	↑	0.00	7.63E-01	NS	↑	0.07	7.69E-01	NS	↑	0.12	8.25E-01	NS	↓	-0.04	3.82E-01	NS	↓	-0.21	4.23E-01	NS	↑	0.33	2.85E-01	NS	↑	0.31	5.22E-01	NS	↑	0.10	9.72E-01	NS	↑	0.01
D6R934	7.71E-01	NS	↓	-0.19	4.25E-01	NS	↑	0.22	2.96E-01	NS	↑	0.35	3.26E-01	NS	↓	-0.61	8.11E-01	NS	↓	-0.18	6.22E-01	NS	↑	0.12	8.44E-01	NS	↑	0.12	4.29E-01	NS	↓	-0.49	8.97E-01	NS	↓	-0.12	3.27E-01	NS	↑	0.55	5.08E-01	NS	↓	-0.41	1.38E-01	NS	↑	0.60	4.77E-01	NS	↑	0.25
A0A024RAG6	5.15E-01	NS	↓	-0.34	3.77E-01	NS	↓	-0.47	6.29E-01	NS	↓	-0.26	2.03E-01	NS	↓	-0.83	2.50E-01	NS	↓	-0.60	6.68E-01	NS	↓	-0.26	2.53E-01	NS	↓	-0.60	2.04E-01	NS	↓	-0.64	5.99E-01	NS	↓	-0.27	9.86E-01	NS	↓	-0.03	1.34E-01	NS	↓	-0.95	4.96E-01	NS	↑	0.20	8.12E-01	NS	↓	-0.16
B1AP58	9.49E-01	NS	↑	0.01	6.24E-01	NS	↑	0.32	1.57E-01	NS	↑	0.51	2.16E-01	NS	↓	-0.62	7.35E-01	NS	↓	-0.12	6.80E-01	NS	↑	0.08	1.34E-01	NS	↑	0.51	6.42E-01	NS	↑	0.08	9.35E-01	NS	↑	0.07	8.63E-01	NS	↑	0.05	3.58E-01	NS	↓	-0.46	2.08E-01	NS	↑	0.39	7.82E-01	NS	↓	-0.18
A8K1K1	9.46E-01	NS	↓	-0.09	4.75E-01	NS	↑	0.77	3.55E-01	NS	↑	0.37	8.80E-01	NS	↓	-0.36	9.84E-01	NS	↓	-0.38	6.44E-01	NS	↑	0.07	8.99E-01	NS	↓	-0.11	8.03E-01	NS	↓	-0.07	7.78E-01	NS	↑	0.33	9.79E-01	NS	↓	-0.06	6.87E-01	NS	↓	-0.60	5.10E-01	NS	↑	0.56	6.82E-01	NS	↑	0.22
A0A140VJI7	9.44E-01	NS	↓	-0.03	6.38E-01	NS	↓	-0.16	2.42E-01	NS	↓	-0.40	9.07E-01	NS	↑	0.02	4.94E-01	NS	↑	0.15	5.69E-01	NS	↓	-0.20	8.23E-01	NS	↑	0.16	4.70E-01	NS	↑	0.15	3.35E-01	NS	↓	-0.34	5.59E-01	NS	↑	0.12	5.59E-01	NS	↑	0.26	4.91E-01	NS	↓	-0.23	5.96E-01	NS	↑	0.16
V9GYM3	6.69E-01	NS	↓	-0.11	6.68E-01	NS	↓	-1.43	5.62E-01	NS	↓	-2.03	4.03E-01	NS	↓	-2.20	7.43E-01	NS	↑	0.45	9.94E-01	NS	↓	-0.64	9.55E-01	NS	↓	-1.35	3.87E-01	NS	↑	1.24	5.52E-01	NS	↑	1.01	4.80E-01	NS	↓	-2.17	4.11E-01	NS	↓	-1.72	7.77E-01	NS	↓	-0.14	6.54E-01	NS	↓	-0.08
B2CIS9	1.35E-01	NS	↓	-0.94	1.11E-01	NS	↓	-0.83	3.07E-01	NS	↓	-0.48	9.05E-01	NS	↑	0.07	6.04E-01	NS	↓	-0.23	7.06E-01	NS	↓	-0.18	6.89E-01	NS	↑	0.46	8.49E-01	NS	↓	-0.05	4.30E-01	NS	↓	-0.34	9.27E-01	NS	↓	-0.07	6.95E-01	NS	↓	-0.11	9.18E-01	NS	↓	-0.05	5.19E-01	NS	↓	-0.27
C9JV77	9.25E-01	NS	↑	0.02	2.14E-01	NS	↓	-0.41	6.75E-01	NS	↓	-0.17	8.65E-01	NS	↑	0.02	7.86E-01	NS	↑	0.22	1.13E-01	NS	↓	-0.67	2.92E-01	NS	↓	-0.41	5.68E-01	NS	↓	-0.16	7.42E-02	NS	↓	-0.60	1.40E-01	NS	↓	-0.58	3.70E-01	NS	↓	-0.37	1.85E-01	NS	↓	-0.50	6.17E-01	NS	↓	-0.18
Q9BS19	4.67E-01	NS	↑	0.18	5.94E-01	NS	↑	0.11	8.83E-01	NS	↓	-0.07	3.61E-01	NS	↑	0.25	9.17E-01	NS	↓	-0.08	7.01E-01	NS	↑	0.12	9.78E-01	NS	↓	-0.05	3.96E-01	NS	↑	0.33	3.71E-01	NS	↑	0.49	5.76E-01	NS	↑	0.12	2.21E-01	NS	↑	0.55	4.97E-01	NS	↑	0.22	5.14E-01	NS	↑	0.20
Q5JVE7	1.85E-01	NS	↑	0.33	1.37E-01	NS	↑	0.43	1.34E-01	NS	↑	0.36	2.21E-01	NS	↑	0.28	1.16E-01	NS	↑	0.42	2.17E-01	NS	↑	0.41	7.73E-02	NS	↑	0.58	4.77E-02	NS	↑	0.61	4.33E-01	NS	↓	-0.26	5.81E-02	NS	↑	0.52	2.05E-01	NS	↑	0.49	2.77E-01	NS	↓	-0.41	8.81E-01	NS	↓	-0.05
J3K000	6.30E-01	NS	↓	-0.26	6.57E-01	NS	↑	0.20	8.56E-01	NS	↑	0.10	8.77E-01	NS	↓	-0.06	9.31E-01	NS	↓	-0.16	2.49E-01	NS	↑	0.46	6.92E-01	NS	↑	0.20	8.17E-01	NS	↑	0.33	8.53E-01	NS	↓	-0.14	6.96E-01	NS	↑	0.34	5.23E-01	NS	↓	-0.39	1.00E+00	NS	↓	-0.05	9.94E-01	NS	↓	-0.05
B2R4M6	2.21E-01	NS	↑	0.77	6.53E-01	NS	↓	-0.44	6.54E-01	NS	↑	0.11	8.77E-02	NS	↑	1.20	5.50E-02	NS	↑	1.96	1.38E-02	NS	↑	2.53	3.62E-02	NS	↑	1.94	1.28E-01	NS	↑	3.07	6.93E-02	NS	↑	2.36	7.07E-01	NS	↑	0.16	1.89E-01	NS	↓	-0.99	4.06E-01	NS	↓	-0.65	6.80E-01	NS	↓	-0.34
D9IWP9	7.25E-01	NS	↑	0.04	3.89E-01	NS	↓	-0.54	1.52E-01	NS	↓	-0.90	9.59E-01	NS	↓	-0.14	9.64E-01	NS	↓	-0.16	2.90E-01	NS	↓	-0.64	6.25E-01	NS	↓	-0.23	4.19E-01	NS	↓	-0.51	7.95E-01	NS	↓	-0.06	9.26E-01	NS	↓	-0.18	8.16E-01	NS	↓	-0.18	2.82E-01	NS	↓	-0.64	7.26E-01	NS	↓	-0.28
A2RTY6	8.49E-01	NS	↓	-0.01	3.14E-01	NS	↑	0.34	2.73E-01	NS	↑	0.36	5.53E-01	NS	↓	-0.38	9.35E-01	NS	↓	-0.16	4.77E-01	NS	↑	0.16	7.37E-01	NS	↑	0.13	9.33E-01	NS	↓	-0.16	7.18E-01	NS	↓	-0.27	7.38E-01	NS	↑	0.12	7.27E-01	NS	↓	-0.27	3.47E-01	NS	↑	0.30	8.06E-01	NS	↑	0.02
S4R3Y4	1.02E-01	NS	↑	0.58	9.17E-01	NS	↑	0.01	5.94E-01	NS	↑	0.12	8.99E-01	NS	↑	0.01	4.07E-01	NS	↑	0.24	7.97E-01	NS	↓	-0.14	8.57E-01	NS	↓	-0.09	5.08E-01	NS	↑	0.15	6.98E-01	NS	↓	-0.15	3.11E-01	NS	↓	-0.47	4.89E-01	NS	↑	0.17	5.83E-01	NS	↑	0.12	7.68E-01	NS	↑	0.05
V9HWD8	9.69E-01	NS	↑	0.11	1.80E-01	NS	↓	-0.51	2.79E-01	NS	↓	-0.42	8.13E-01	NS	↑	0.23	8.62E-01	NS	↑	0.01	8.45E-02	NS	↓	-0.75	2.63E-01	NS	↓	-0.46	4.19E-01	NS	↓	-0.30	3.85E-01	NS	↓	-0.31	1.10E-01	NS	↓	-0.51	6.16E-01	NS	↓	-0.17	1.66E-01	NS	↓	-0.48	6.16E-01	NS	↓	-0.18
B4E1Z4	1.98E-01	NS	↓	-0.20	3.01E-01	NS	↓	-0.20	6.71E-01	NS	↓	-0.08	6.26E-01	NS	↑	0.10	6.05E-01	NS	↓	-0.10	8.22E-01	NS	↑	0.05	6.33E-01	NS	↓	-0.10	4.58E-01	NS	↓	-0.16	3.54E-01	NS	↓	-0.16	8.50E-02	NS	↓	-0.27	6.71E-01	NS	↑	0.20	6.83E-01	NS	↓	-0.07	8.38E-01	NS	↓	-0.04
A0A384N669	9.13E-01	NS	↓	-0.08	6.92E-01	NS	↓	-0.22	5.51E-01	NS	↓	-0.32	8.71E-01	NS	↓	-0.10	8.80E-01	NS	↓	-0.07	5.95E-01	NS	↓	-0.30	8.65E-01	NS	↓	-0.12	9.99E-01	NS	↓	-0.02	9.00E-01	NS	↓	-0.13	7.12E-01	NS	↓	-0.24	4.69E-01	NS	↓	-0.39	6.42E-01	NS	↓	-0.27	7.72E-01	NS	↓	-0.18
F8W7L3	3.78E-01	NS	↑	0.72	3.44E-01	NS	↑	0.33	2.97E-01	NS	↑	0.32	3.48E-01	NS	↑	0.38	5.99E-02	NS	↑	1.37	5.76E-01	NS	↑	0.27	4.65E-01	NS	↑	0.28	4.98E-02	NS	↑	1.66	2.00E-02	NS	↑	1.87	6.55E-01	NS	↑	0.19	2.14E-01	NS	↑	0.64	2.39E-01	NS	↑	0.74	4.53E-01	NS	↑	0.58
B0YIW2	4.94E-01	NS	↑	0.52	7.28E-01	NS	↑	0.02	8.49E-01	NS	↑	0.10	8.53E-01	NS	↑	0.22	3.93E-01	NS	↑	0.37	1.67E-01	NS	↓	-0.81	8.20E-01	NS	↑	0.02	7.15E-01	NS	↓	-0.23	6.20E-01	NS	↓	-0.25	2.55E-01	NS	↓	-0.70	2.30E-01	NS	↓	-0.65	9.84E-01	NS	↑	0.15	6.31E-01	NS	↓	-0.15
Q5SQ08	7.63E-01	NS	↓	-0.22	4.41E-01	NS	↑	0.25	2.50E-01	NS	↑	0.38	8.22E-01	NS	↓	-0.01	9.33E-01	NS	↓	-0.09	7.79E-01	NS	↑	0.01	8.87E-01	NS	↑	0.11	9.61E-01	NS	↓	-0.07	9.37E-01	NS	↓	-0.08	3.19E-01	NS	↑	0.43	3.72E-01	NS	↑	0.39	3.82E-01	NS	↑	0.25	8.72E-01	NS	↓	-0.02
Q8TAY0	9.49E-01	NS	↓	-0.07	3.26E-01	NS	↑	0.37	3.36E-01	NS	↑	0.37	5.42E-01	NS	↓	-0.24	8.04E-01	NS	↓	-0.16	4.51E-01	NS	↑	0.17	8.20E-01	NS	↑	0.04	9.27E-01	NS	↑	0.02	9.06E-01	NS	↑	0.00	7.92E-01	NS	↑	0.26	8.85E-01	NS	↓	-0.14	5.71E-01	NS	↑	0.24	8.56E-01	NS	↑	0.05
A0A0A0MRJ7	2.95E-01	NS	↑	0.21	1.94E-01	NS	↓	-0.32	1.86E-01	NS	↓	-0.42	9.16E-01	NS	↑	0.05	5.61E-01	NS	↓	-0.14	2.51E-02	NS	↓	-0.56	2.45E-01	NS	↓	-0.32	4.75E-01	NS	↓	-0.19	8.94E-01	NS	↓	-0.02	6.05E-01	NS	↓	-0.12	2.05E-01	NS	↓	-0.35	4.04E-01	NS	↓	-0.17	8.02E-01	NS	↑	0.04
A0A1K0GXZ1	4.79E-01	NS	↓	-1.71	3.04E-01	NS	↑	1.04	4.79E-01	NS	↓	-0.06	8.30E-01	NS	↑	0.24	2.51E-01	NS	↑	1.44	2.16E-01	NS	↑	0.93	1.97E-01	NS	↑	1.53	5.76E-01	NS	↑	0.14	5.08E-01	NS	↑	2.08	5.96E-01	NS	↓	-1.23	3.78E-01	NS	↓	-1.65	7.34E-01	NS	↓	-0.20	7.86E-01	NS	↓	-1.05
D3JV41	6.20E-01	NS	↑	0.28	6.91E-01	NS	↓	-0.15	7.59E-01	NS	↑	0.09	6.01E-01	NS	↓	-0.21	4.97E-01	NS	↓	-0.26	5.26E-01	NS	↓	-0.17	6.03E-01	NS	↓	-0.19	5.84E-01	NS	↑	0.22	2.09E-01	NS	↓	-0.54	5.88E-01	NS	↓	-0.21	3.73E-01	NS	↓	-0.36	3.33E-01	NS	↑	0.37	4.91E-01	NS	↓	-0.26
A0N071	2.64E-01	NS	↓	-0.98	6.86E-01	NS	↑	1.04	3.57E-01	NS	↑	1.52	7.06E-01	NS	↓	-0.40	1.57E-01	NS	↑	1.32	2.77E-01	NS	↑	2.02	1.22E-01	NS	↑	2.33	8.98E-01	NS	↑	0.01	7.52E-01	NS	↑	0.70	9.06E-02	NS	↓	-1.69	2.08E-02	NS	↓	-2.14	3.09E-01	NS	↓	-0.86	2.58E-01	NS	↓	-0.98
C0JYY2	5.91E-01	NS	↓	-0.19	5.44E-01	NS	↑	0.10	6.50E-01	NS	↑	0.09	7.25E-01	NS	↓	-0.13	6.69E-01	NS	↓	-0.16	1.64E-01	NS	↑	0.34	8.78E-01	NS	↑	0.05	7.33E-01	NS	↓	-0.14	9.68E-01	NS	↓	-0.05	3.61E-01	NS	↑	0.27	8.54E-01	NS	↓	-0.09	9.15E-02	NS	↑	0.43	1.41E-01	NS	↑	0.40
B3KS79	9.46E-01	NS	↓	-0.02	3.92E-01	NS	↑	0.20	4.79E-01	NS	↑	0.22	6.93E-01	NS	↓	-0.12	8.87E-01	NS	↓	-0.05	2.12E-01	NS	↑	0.35	1.47E-01	NS	↑	0.48	3.47E-01	NS	↑	0.18	1.63E-01	NS	↑	0.40	1.25E-01	NS	↑	0.59	2.77E-01	NS	↑	0.37	1.92E-01	NS	↑	0.33	4.42E-01	NS	↑	0.19
H0YAC1	3.42E-01	NS	↑	0.26	3.89E-01	NS	↑	0.14	8.48E-01	NS	↑	0.04	1.81E-01	NS	↑	0.37	6.24E-01	NS	↑	0.07	3.72E-01	NS	↑	0.18	6.20E-01	NS	↑	0.09	1.15E-01	NS	↑	0.37	7.19E-01	NS	↓	-0.10	8.73E-01	NS	↑	0.00	2.35E-01	NS	↑	0.54	9.12E-01	NS	↓	-0.05	3.97E-01	NS	↑	0.27
B7Z549	5.52E-01	NS	↓	-0.27	5.48E-01	NS	↑	0.24	2.46E-01	NS	↑	0.35	8.80E-01	NS	↓	-0.06	8.65E-01	NS	↓	-0.05	3.68E-01	NS	↑	0.30	9.73E-01	NS	↑	0.07	8.13E-01	NS	↓	-0.11	8.25E-01	NS	↓	-0.12	5.43E-01	NS	↑	0.36	8.89E-01	NS	↓	-0.08	3.73E-01	NS	↑	0.26	8.92E-01	NS	↑	0.01
Q59EP2	7.84E-01	NS	↑	0.05	1.05E-01	NS	↑	0.27	2.27E-01	NS	↑	0.19	5.13E-01	NS	↓	-0.13	9.49E-01	NS	↑	0.04	3.37E-02	NS	↑	0.38	1.71E-01	NS	↑	0.49	8.60E-02	NS	↑	0.49	4.80E-02	NS	↑	0.39	3.02E-01	NS	↑	0.36	7.83E-01	NS	↓	-0.03	6.49E-01	NS	↑	0.07	5.89E-01	NS	↓	-0.10
F5H1C6	6.70E-01	NS	↑	0.08	3.53E-01	NS	↑	0.54	9.08E-01	NS	↑	0.32	9.09E-01	NS	↓	-0.02	3.89E-01	NS	↑	0.28	5.68E-01	NS	↑	0.18	3.16E-01	NS	↓	-0.38	8.90E-01	NS	↓	-0.05	4.19E-01	NS	↑	0.60	8.38E-01	NS	↓	-0.03	6.65E-01	NS	↓	-0.06	3.78E-01	NS	↑	0.31	8.30E-01	NS	↑	0.04
A0A0S2Z4I5	6.33E-01	NS	↑	0.30	4.75E-01	NS	↓	-0.18	9.73E-01	NS	↑	0.00	2.61E-01	NS	↓	-0.24	3.38E-01	NS	↑	0.27	4.04E-01	NS	↑	0.42	3.80E-01	NS	↑	0.21	5.34E-01	NS	↓	-0.14	8.27E-01	NS	↑	0.02	5.87E-01	NS	↓	-0.12	9.47E-01	NS	↑	0.02	9.14E-01	NS	↑	0.03	8.21E-01	NS	↑	0.06
Q96K68	9.54E-01	NS	↓	-0.08	4.73E-01	NS	↓	-0.38	2.92E-01	NS	↓	-0.55	7.11E-01	NS	↓	-0.20	7.97E-01	NS	↑	0.10	2.64E-01	NS	↓	-0.60	7.86E-01	NS	↓	-0.04	5.79E-01	NS	↑	0.57	4.61E-01	NS	↑	0.40	8.65E-01	NS	↑	0.04	7.97E-01	NS	↓	-0.10	3.91E-01	NS	↑	0.37	5.32E-01	NS	↑	0.26
H6VRG1	5.74E-01	NS	↓	-0.27	6.02E-01	NS	↓	-0.38	6.32E-01	NS	↓	-0.30	8.20E-01	NS	↓	-0.15	9.81E-01	NS	↑	0.16	9.70E-01	NS	↑	0.16	4.76E-01	NS	↑	0.30	5.57E-01	NS	↑	0.62	7.40E-01	NS	↑	1.26	4.79E-01	NS	↑	0.34	2.42E-01	NS	↓	-0.78	2.41E-01	NS	↓	-0.74	6.07E-01	NS	↑	0.41
F8VV57	2.99E-01	NS	↓	-0.81	7.90E-01	NS	↑	0.22	6.14E-01	NS	↓	-0.37	5.70E-01	NS	↓	-0.46	6.75E-01	NS	↓	-0.02	4.42E-01	NS	↓	-0.54	7.70E-01	NS	↓	-0.04	4.96E-01	NS	↑	0.76	9.40E-01	NS	↑	0.63	5.79E-01	NS	↑	0.18	4.25E-01	NS	↓	-0.65	4.65E-01	NS	↓	-0.46	9.88E-01	NS	↓	-0.07
A0A1B0GUU9	7.10E-01	NS	↓	-0.47	8.47E-01	NS	↓	-0.40	7.35E-01	NS	↓	-0.57	3.11E-01	NS	↓	-1.06	4.10E-01	NS	↓	-0.84	7.18E-01	NS	↓	-0.49	5.20E-01	NS	↓	-0.74	7.49E-01	NS	↓	-0.50	5.94E-01	NS	↑	0.20	5.06E-01	NS	↑	0.41	2.02E-01	NS	↓	-1.31	6.75E-01	NS	↓	-0.44	9.06E-01	NS	↓	-0.32
B2R6W1	3.28E-01	NS	↓	-0.25	6.47E-01	NS	↓	-0.11	9.33E-01	NS	↑	0.00	3.20E-01	NS	↓	-0.23	1.24E-01	NS	↓	-0.31	8.78E-01	NS	↓	-0.04	9.58E-01	NS	↑	0.03	2.13E-01	NS	↓	-0.29	1.87E-01	NS	↓	-0.27	6.73E-01	NS	↑	0.11	5.61E-01	NS	↓	-0.14	3.82E-01	NS	↑	0.20	6.75E-01	NS	↓	-0.10
Q59EA3	5.62E-01	NS	↓	-0.44	6.87E-01	NS	↓	-0.43	6.78E-01	NS	↓	-0.14	7.22E-01	NS	↑	0.51	4.61E-01	NS	↓	-0.64	5.65E-01	NS	↓	-0.04	4.96E-01	NS	↑	0.26	4.31E-01	NS	↑	0.23	4.27E-01	NS	↓	-1.10	7.66E-01	NS	↓	-0.17	3.90E-01	NS	↑	0.38	9.48E-01	NS	↓	-0.46	8.46E-01	NS	↓	-0.01
G3V2W1	6.22E-01	NS	↓	-0.14	7.40E-01	NS	↓	-0.12	3.95E-01	NS	↓	-0.31	3.39E-01	NS	↓	-0.49	8.74E-01	NS	↓	-0.08	4.21E-01	NS	↓	-0.29	6.34E-01	NS	↓	-0.14	2.43E-01	NS	↓	-0.89	7.19E-01	NS	↓	-0.13	7.71E-01	NS	↑	0.03	2.11E-01	NS	↓	-0.41	2.26E-01	NS	↓	-0.62	7.46E-01	NS	↑	0.04
A0A024R1X6	6.85E-01	NS	↓	-0.10	4.34E-01	NS	↓	-0.66	8.33E-01	NS	↓	-0.25	7.28E-01	NS	↑	0.22	9.15E-01	NS	↑	0.04	8.98E-01	NS	↑	0.51	7.58E-01	NS	↑	0.12	8.24E-01	NS	↑	0.61	7.98E-01	NS	↑	0.60	9.35E-01	NS	↓	-0.14	1.57E-01	NS	↓	-1.07	1.94E-01	NS	↓	-0.99	7.70E-01	NS	↓	-0.34
V9HW68	8.13E-01	NS	↑	0.32	5.14E-01	NS	↓	-0.23	5.81E-01	NS	↓	-0.11	3.63E-01	NS	↓	-0.39	9.29E-01	NS	↑	1.12	4.93E-02	NS	↑	0.70	7.66E-02	NS	↑	0.70	1.20E-01	NS	↑	1.64	2.09E-01	NS	↑	1.51	5.15E-01	NS	↑	0.45	2.93E-01	NS	↓	-0.47	5.52E-02	NS	↑	0.96	2.76E-01	NS	↑	0.53
A6XND1	7.56E-01	NS	↑	0.07	4.67E-01	NS	↓	-0.36	4.40E-01	NS	↓	-0.40	6.91E-01	NS	↑	0.09	9.65E-01	NS	↓	-0.07	2.17E-01	NS	↓	-0.63	4.97E-01	NS	↓	-0.34	5.46E-01	NS	↓	-0.33	4.50E-01	NS	↓	-0.38	8.56E-01	NS	↓	-0.14	7.32E-01	NS	↓	-0.18	2.55E-01	NS	↓	-0.54	5.96E-01	NS	↓	-0.30
A0A087WW43	9.09E-01	NS	↓	-0.05	5.39E-01	NS	↑	0.17	9.27E-02	NS	↑	0.57	9.09E-01	NS	↓	-0.07	9.24E-01	NS	↓	-0.03	5.65E-01	NS	↑	0.24	7.09E-01	NS	↑	0.28	9.27E-01	NS	↓	-0.01	8.69E-01	NS	↓	-0.02	4.36E-01	NS	↑	0.20	5.73E-01	NS	↑	0.15	1.28E-01	NS	↑	0.43	5.29E-01	NS	↑	0.12
Q13784	8.35E-01	NS	↑	0.06	9.52E-01	NS	↑	0.11	5.14E-01	NS	↓	-0.47	7.19E-01	NS	↑	0.13	6.28E-01	NS	↑	0.40	7.68E-01	NS	↑	0.26	2.99E-01	NS	↑	0.99	2.95E-01	NS	↑	0.49	6.09E-01	NS	↑	0.37	1.75E-01	NS	↑	0.76	9.91E-01	NS	↑	0.09	8.27E-01	NS	↓	-0.06	9.29E-01	NS	↑	0.01
A0A024R6N9	7.42E-01	NS	↓	-0.10	3.50E-01	NS	↓	-0.30	4.11E-01	NS	↓	-0.28	1.60E-01	NS	↓	-0.41	1.60E-01	NS	↓	-0.37	9.74E-01	NS	↑	0.05	6.88E-01	NS	↓	-0.11	6.28E-01	NS	↓	-0.14	9.64E-01	NS	↓	-0.03	9.10E-01	NS	↓	-0.06	2.75E-01	NS	↓	-0.38	2.99E-01	NS	↑	0.21	9.58E-01	NS	↑	0.07
D6RAR4	9.79E-01	NS	↓	-0.01	5.25E-01	NS	↓	-0.34	7.13E-01	NS	↓	-0.20	9.86E-01	NS	↓	0.00	9.98E-01	NS	↓	0.00	5.00E-01	NS	↓	-0.36	5.22E-01	NS	↓	-0.34	7.28E-01	NS	↓	-0.21	3.44E-01	NS	↓	-0.49	4.86E-01	NS	↓	-0.37	5.38E-01	NS	↓	-0.28	5.19E-01	NS	↓	-0.35	7.42E-01	NS	↓	-0.19
A8K8Z4	9.37E-01	NS	↓	-0.02	3.49E-01	NS	↓	-0.24	5.04E-01	NS	↓	-0.18	7.10E-01	NS	↓	-0.10	5.99E-01	NS	↓	-0.14	7.19E-01	NS	↓	-0.09	7.41E-01	NS	↓	-0.09	8.36E-01	NS	↑	0.06	9.06E-01	NS	↓	-0.01	7.02E-01	NS	↑	0.06	9.60E-01	NS	↓	-0.04	5.70E-01	NS	↑	0.09	7.07E-01	NS	↓	-0.10
Q53GN8	6.09E-01	NS	↑	0.25	9.64E-01	NS	↑	0.15	9.86E-01	NS	↓	-0.02	9.46E-01	NS	↑	0.21	5.74E-01	NS	↑	0.36	4.64E-01	NS	↓	-0.28	3.29E-01	NS	↓	-0.38	7.50E-01	NS	↓	-0.15	2.84E-01	NS	↓	-0.47	3.92E-01	NS	↓	-0.32	7.15E-01	NS	↑	0.32	6.79E-01	NS	↑	0.15	9.96E-01	NS	↑	0.15
RT-Cal protein	8.41E-01	NS	↑	0.01	4.19E-01	NS	↓	-0.13	5.23E-01	NS	↓	-0.09	4.45E-01	NS	↑	0.10	8.02E-01	NS	↓	-0.04	7.42E-01	NS	↓	-0.05	4.06E-01	NS	↓	-0.15	4.14E-01	NS	↓	-0.12	2.68E-01	NS	↓	-0.21	2.61E-01	NS	↓	-0.16	6.74E-01	NS	↑	0.06	7.32E-01	NS	↑	0.04	9.66E-01	NS	↑	0.01
A0A384NKS6	7.45E-01	NS	↓	-0.06	3.91E-01	NS	↓	-0.17	2.27E-01	NS	↓	-0.20	1.16E-01	NS	↓	-0.37	3.37E-01	NS	↓	-0.18	2.51E-01	NS	↓	-0.20	2.38E-01	NS	↓	-0.26	7.84E-03	NS	↓	-0.84	1.49E-02	NS	↓	-0.46	2.53E-01	NS	↓	-0.23	5.88E-02	NS	↓	-0.61	8.13E-01	NS	↑	0.10	5.05E-01	NS	↓	-0.14
D9ZGG2	8.52E-01	NS	↓	-0.05	2.92E-01	NS	↓	-0.23	4.24E-01	NS	↓	-0.16	4.98E-01	NS	↑	0.15	7.36E-01	NS	↓	-0.08	1.79E-01	NS	↓	-0.27	3.56E-01	NS	↓	-0.20	2.52E-01	NS	↓	-0.27	2.20E-01	NS	↓	-0.31	2.35E-01	NS	↓	-0.29	8.67E-01	NS	↓	-0.04	9.51E-01	NS	↓	-0.03	6.75E-01	NS	↑	0.09
Q68CK4	2.24E-01	NS	↓	-0.23	5.30E-01	NS	↓	-0.14	7.14E-02	NS	↓	-0.53	2.94E-01	NS	↓	-0.25	6.29E-01	NS	↓	-0.09	4.62E-01	NS	↓	-0.17	8.83E-01	NS	↑	0.14	8.33E-01	NS	↓	-0.02	4.64E-01	NS	↑	0.30	2.81E-01	NS	↓	-0.44	3.89E-01	NS	↓	-0.20	8.06E-01	NS	↑	0.10	8.87E-01	NS	↑	0.11
A0A0J9YY65	2.98E-01	NS	↓	-0.25	7.19E-01	NS	↓	-0.08	2.88E-01	NS	↑	0.48	6.25E-01	NS	↓	-0.11	8.49E-01	NS	↓	-0.05	1.30E-01	NS	↑	0.51	1.58E-01	NS	↑	0.58	7.90E-01	NS	↓	-0.06	7.45E-01	NS	↑	0.09	4.16E-01	NS	↑	0.40	7.35E-01	NS	↑	0.10	1.75E-01	NS	↑	0.26	3.29E-01	NS	↑	0.39
F5GXS0	7.26E-01	NS	↓	-0.20	5.13E-01	NS	↑	0.14	4.11E-01	NS	↑	0.28	3.73E-01	NS	↓	-0.41	8.22E-01	NS	↓	-0.15	4.25E-01	NS	↑	0.17	9.67E-01	NS	↓	-0.02	8.82E-01	NS	↓	-0.06	8.31E-01	NS	↑	0.05	7.02E-01	NS	↑	0.24	8.59E-01	NS	↑	0.11	2.87E-01	NS	↑	0.41	5.00E-01	NS	↑	0.21
A0A384MEF1	7.59E-01	NS	↓	-0.12	7.11E-01	NS	↑	0.18	4.18E-01	NS	↑	0.29	8.48E-01	NS	↑	0.18	7.91E-01	NS	↑	0.03	1.12E-01	NS	↑	0.49	4.40E-01	NS	↑	0.27	8.12E-01	NS	↑	0.13	9.59E-01	NS	↑	0.01	5.20E-01	NS	↑	0.55	3.71E-01	NS	↑	0.28	3.81E-01	NS	↑	0.40	7.20E-01	NS	↑	0.18
Q5JP69	4.72E-01	NS	↑	0.09	6.26E-01	NS	↓	-0.06	8.61E-01	NS	↑	0.06	7.41E-01	NS	↓	-0.02	5.37E-01	NS	↓	-0.12	2.87E-01	NS	↑	0.29	1.09E-01	NS	↓	-0.62	8.10E-01	NS	↑	0.12	6.01E-01	NS	↓	-0.08	8.83E-02	NS	↓	-0.55	8.54E-01	NS	↑	0.08	7.28E-01	NS	↓	-0.05	5.56E-01	NS	↓	-0.10
Q8IVC0	5.67E-01	NS	↓	-0.18	4.39E-01	NS	↑	0.15	8.24E-01	NS	↑	0.09	2.67E-01	NS	↓	-0.40	5.86E-01	NS	↓	-0.14	9.26E-01	NS	↑	0.05	5.61E-01	NS	↓	-0.17	1.66E-01	NS	↓	-0.42	6.19E-01	NS	↓	-0.15	6.14E-01	NS	↑	0.10	3.33E-01	NS	↓	-0.38	3.31E-01	NS	↑	0.25	4.03E-01	NS	↑	0.17
B7Z8Q5	8.78E-01	NS	↑	0.08	2.00E-01	NS	↓	-1.18	4.92E-01	NS	↓	-0.29	2.74E-01	NS	↑	0.36	9.49E-01	NS	↑	0.04	6.73E-01	NS	↓	-0.38	6.91E-01	NS	↑	0.18	5.45E-01	NS	↓	-0.41	2.99E-01	NS	↓	-0.88	7.50E-01	NS	↓	-0.40	1.78E-01	NS	↓	-1.15	9.91E-01	NS	↓	-0.29	3.65E-01	NS	↑	0.29
Q4LE79	7.33E-01	NS	↓	-0.18	7.98E-01	NS	↓	-0.48	5.03E-01	NS	↓	-0.92	6.28E-01	NS	↓	-0.76	1.85E-01	NS	↓	-1.52	8.62E-01	NS	↓	-0.25	4.95E-01	NS	↓	-0.89	6.39E-01	NS	↓	-0.02	6.62E-01	NS	↓	-0.60	8.95E-01	NS	↓	-0.07	9.91E-01	NS	↓	-0.39	3.62E-01	NS	↓	-1.14	9.66E-01	NS	↓	-0.27
Q9UEU7	8.78E-01	NS	↑	0.31	4.26E-01	NS	↑	0.22	8.71E-01	NS	↓	-0.11	7.50E-01	NS	↑	0.58	6.88E-01	NS	↑	0.24	5.46E-01	NS	↑	0.25	4.97E-01	NS	↓	-0.42	8.30E-01	NS	↑	0.53	6.62E-01	NS	↓	-0.22	5.26E-01	NS	↓	-0.33	3.51E-01	NS	↑	0.62	8.87E-01	NS	↓	-0.07	9.74E-01	NS	↓	-0.15
Q5VY30	5.10E-01	NS	↑	0.64	8.58E-01	NS	↓	-0.12	8.58E-01	NS	↓	-0.11	5.63E-01	NS	↑	0.19	9.26E-01	NS	↑	0.13	3.99E-01	NS	↓	-0.41	3.77E-01	NS	↓	-0.43	7.94E-01	NS	↓	-0.18	1.89E-01	NS	↓	-0.63	2.83E-01	NS	↓	-0.53	3.12E-01	NS	↑	0.32	1.91E-01	NS	↓	-0.63	7.98E-01	NS	↓	-0.11
A0A024R3E3	4.34E-01	NS	↑	0.90	2.04E-01	NS	↓	-0.92	3.07E-01	NS	↓	-0.60	2.42E-01	NS	↓	-0.90	6.24E-01	NS	↑	1.64	2.74E-01	NS	↓	-0.74	8.80E-01	NS	↑	0.44	7.85E-01	NS	↑	2.28	1.95E-01	NS	↑	2.25	1.49E-01	NS	↑	0.88	7.83E-01	NS	↓	-0.26	1.23E-01	NS	↑	1.48	6.42E-02	NS	↑	1.34
K7ERE3	6.80E-01	NS	↓	-0.18	8.28E-01	NS	↓	-0.37	6.91E-01	NS	↓	-0.50	6.28E-01	NS	↑	0.36	8.68E-01	NS	↑	0.54	5.21E-01	NS	↓	-0.45	9.88E-01	NS	↑	0.49	9.90E-02	NS	↑	1.14	6.86E-01	NS	↑	1.76	3.43E-01	NS	↑	0.29	6.62E-01	NS	↑	1.18	1.51E-01	NS	↓	-1.69	9.93E-01	NS	↑	0.14
B4DPQ0	8.49E-01	NS	↑	0.02	6.92E-01	NS	↑	0.05	7.44E-01	NS	↑	0.03	9.74E-01	NS	↓	-0.07	6.76E-01	NS	↓	-0.24	6.75E-01	NS	↑	0.06	8.11E-01	NS	↑	0.07	5.76E-01	NS	↓	-0.29	8.84E-01	NS	↓	-0.02	3.72E-01	NS	↑	0.23	5.52E-01	NS	↓	-0.30	4.05E-01	NS	↑	0.24	9.43E-01	NS	↓	-0.05
Q59EZ3	1.05E-01	NS	↑	1.52	3.81E-01	NS	↑	0.56	3.83E-01	NS	↑	0.60	3.92E-01	NS	↑	0.73	7.67E-01	NS	↓	-0.59	5.68E-01	NS	↓	-0.76	2.23E-01	NS	↑	0.78	2.89E-01	NS	↑	0.36	2.42E-01	NS	↑	0.60	8.22E-01	NS	↑	0.24	6.87E-01	NS	↑	0.30	9.79E-01	NS	↑	0.18	6.92E-01	NS	↓	-0.31
B2R950	6.83E-01	NS	↓	-0.37	9.31E-01	NS	↓	-0.05	8.90E-01	NS	↓	-0.10	4.62E-01	NS	↓	-0.65	7.94E-01	NS	↓	-0.33	8.84E-01	NS	↓	-0.10	8.60E-01	NS	↓	-0.21	9.78E-01	NS	↓	-0.13	8.24E-01	NS	↑	0.00	8.70E-01	NS	↑	0.09	6.85E-01	NS	↓	-0.34	7.38E-01	NS	↑	0.10	8.46E-01	NS	↓	-0.05
V9HWP0	7.79E-01	NS	↑	0.14	4.81E-01	NS	↑	0.27	7.44E-01	NS	↑	0.23	2.27E-01	NS	↓	-0.73	4.09E-01	NS	↓	-0.53	6.88E-01	NS	↓	-0.30	7.90E-01	NS	↓	-0.18	9.20E-01	NS	↓	-0.16	9.88E-01	NS	↓	-0.12	8.76E-01	NS	↑	0.09	3.28E-01	NS	↓	-0.61	3.41E-01	NS	↑	0.38	6.78E-01	NS	↓	-0.32
A0A0G2JPR0	5.04E-01	NS	↓	-0.20	4.38E-01	NS	↑	0.19	8.60E-01	NS	↑	0.00	2.62E-01	NS	↓	-0.37	5.10E-01	NS	↓	-0.19	7.16E-01	NS	↑	0.10	7.08E-01	NS	↓	-0.10	3.18E-01	NS	↓	-0.29	6.05E-01	NS	↑	0.13	3.56E-01	NS	↑	0.48	8.79E-01	NS	↓	-0.02	1.26E-01	NS	↑	0.38	4.00E-01	NS	↑	0.17
J3KPA1	2.66E-01	NS	↑	0.80	4.67E-01	NS	↓	-0.26	4.10E-01	NS	↓	-0.30	9.80E-01	NS	↑	0.08	9.42E-01	NS	↑	0.20	3.54E-01	NS	↓	-0.38	8.39E-01	NS	↑	0.11	6.99E-01	NS	↓	-0.11	8.52E-01	NS	↑	0.11	5.27E-01	NS	↓	-0.23	7.48E-01	NS	↓	-0.11	8.02E-01	NS	↓	-0.06	9.56E-01	NS	↑	0.04
Q59GS8	2.32E-01	NS	↓	-0.38	6.97E-01	NS	↑	0.05	6.07E-01	NS	↑	0.16	3.72E-01	NS	↓	-0.31	7.79E-01	NS	↓	-0.02	6.61E-01	NS	↑	0.12	7.25E-01	NS	↓	-0.08	6.80E-01	NS	↓	-0.13	8.27E-01	NS	↑	0.01	3.35E-01	NS	↑	0.35	5.20E-01	NS	↓	-0.21	5.54E-01	NS	↑	0.12	7.09E-01	NS	↑	0.10
A0A0F7G8J1	9.67E-01	NS	↑	0.02	7.63E-01	NS	↑	0.15	5.19E-01	NS	↑	0.22	5.00E-01	NS	↑	0.43	5.39E-01	NS	↓	-0.12	7.69E-01	NS	↑	0.32	4.22E-01	NS	↑	0.33	1.72E-01	NS	↑	0.51	5.77E-01	NS	↓	-0.10	2.10E-01	NS	↑	0.41	6.97E-03	NS	↑	0.41	2.41E-01	NS	↑	0.29	7.56E-01	NS	↓	-0.03
B2R815	8.26E-01	NS	↓	-0.17	7.00E-01	NS	↑	0.11	6.17E-01	NS	↑	0.10	4.13E-01	NS	↓	-0.49	7.18E-01	NS	↓	-0.30	7.85E-01	NS	↓	-0.02	7.94E-01	NS	↑	0.01	9.00E-01	NS	↓	-0.16	8.38E-01	NS	↑	0.05	6.62E-01	NS	↑	0.13	7.62E-01	NS	↓	-0.27	5.10E-01	NS	↑	0.21	8.94E-01	NS	↓	-0.04
S4R471	4.39E-01	NS	↑	0.38	2.44E-01	NS	↑	0.36	7.86E-01	NS	↑	0.25	2.08E-01	NS	↓	-0.58	7.51E-01	NS	↓	-0.09	7.02E-01	NS	↓	-0.10	6.45E-01	NS	↓	-0.09	1.31E-01	NS	↓	-0.36	4.32E-01	NS	↓	-0.22	9.12E-01	NS	↑	0.00	3.29E-01	NS	↓	-0.30	3.24E-01	NS	↑	0.40	6.95E-01	NS	↓	-0.09
B3KNX0	6.53E-01	NS	↑	0.13	4.20E-01	NS	↑	0.58	1.83E-01	NS	↓	-0.81	6.30E-01	NS	↑	0.09	9.40E-01	NS	↓	0.00	7.28E-01	NS	↓	-0.03	2.79E-01	NS	↑	0.46	3.10E-01	NS	↑	0.35	7.39E-01	NS	↑	0.14	2.13E-01	NS	↑	0.43	9.11E-01	NS	↑	0.08	1.25E-01	NS	↑	0.61	5.55E-01	NS	↑	0.33
P78492	3.99E-01	NS	↓	-0.11	9.57E-01	NS	↑	0.04	1.88E-01	NS	↑	0.23	2.01E-01	NS	↑	0.15	6.71E-01	NS	↑	0.11	2.20E-01	NS	↑	0.20	6.81E-01	NS	↑	0.09	2.85E-01	NS	↑	0.12	5.05E-01	NS	↓	-0.11	7.43E-01	NS	↓	-0.04	3.01E-01	NS	↑	0.15	6.10E-01	NS	↓	-0.07	3.25E-01	NS	↑	0.19
B4DRY0	9.59E-01	NS	↑	1.02	2.77E-01	NS	↓	-0.63	5.11E-01	NS	↓	-0.37	8.84E-01	NS	↑	0.41	7.87E-01	NS	↑	0.40	4.11E-01	NS	↑	1.32	4.73E-01	NS	↑	0.36	6.71E-01	NS	↑	1.30	7.09E-01	NS	↑	1.24	8.41E-01	NS	↓	-0.17	5.66E-02	NS	↓	-1.20	3.90E-01	NS	↓	-0.49	5.16E-01	NS	↓	-0.38
Q6MZL2	9.90E-01	NS	↑	0.01	8.09E-01	NS	↓	-0.04	8.46E-01	NS	↑	0.15	5.13E-01	NS	↑	0.17	5.32E-01	NS	↑	0.10	9.30E-01	NS	↑	0.03	5.05E-01	NS	↑	0.13	6.74E-01	NS	↓	-0.09	7.58E-01	NS	↓	-0.07	9.65E-01	NS	↓	-0.01	7.37E-01	NS	↓	-0.07	3.36E-01	NS	↓	-0.49	5.72E-01	NS	↑	0.19
Q59E93	9.93E-01	NS	↓	-0.16	3.02E-01	NS	↓	-0.77	6.19E-01	NS	↓	-0.22	7.09E-01	NS	↓	-0.36	8.11E-01	NS	↓	-0.21	3.31E-01	NS	↑	0.37	9.06E-01	NS	↑	0.27	5.14E-01	NS	↓	-0.44	5.58E-01	NS	↓	-0.17	9.25E-01	NS	↓	-0.21	1.47E-01	NS	↓	-1.08	7.57E-01	NS	↓	-0.02	7.77E-01	NS	↑	0.00
Q9UNL6	3.50E-01	NS	↓	-1.67	8.52E-01	NS	↓	-0.26	9.32E-01	NS	↓	-0.80	9.70E-01	NS	↓	-0.63	7.42E-01	NS	↑	0.20	3.70E-01	NS	↑	0.92	4.50E-01	NS	↑	0.37	8.11E-01	NS	↓	-0.38	9.08E-01	NS	↑	0.67	6.47E-01	NS	↓	-0.80	4.78E-01	NS	↓	-1.45	6.89E-01	NS	↓	-0.34	4.44E-01	NS	↓	-1.54
V9HWF6	6.29E-01	NS	↓	-0.06	6.71E-01	NS	↓	-0.87	6.65E-01	NS	↓	-0.42	7.51E-01	NS	↓	-0.67	8.79E-01	NS	↓	-1.36	7.18E-01	NS	↓	-0.41	9.59E-01	NS	↓	-0.55	6.81E-01	NS	↑	0.66	5.53E-01	NS	↑	0.23	7.97E-01	NS	↑	0.68	7.56E-01	NS	↓	-2.21	8.36E-01	NS	↓	-1.14	7.04E-01	NS	↓	-2.07
B2R773	8.52E-01	NS	↓	-0.20	8.37E-01	NS	↑	0.08	9.63E-01	NS	↓	-0.27	9.37E-01	NS	↑	0.03	3.16E-01	NS	↓	-0.75	9.96E-01	NS	↑	0.13	8.29E-01	NS	↑	0.14	9.24E-01	NS	↑	0.00	4.10E-01	NS	↑	2.66	3.47E-01	NS	↑	0.62	8.82E-01	NS	↓	-0.02	8.41E-01	NS	↑	0.19	9.68E-01	NS	↑	0.13
V9HWA9	6.81E-01	NS	↓	-0.21	5.09E-01	NS	↑	0.17	3.27E-01	NS	↑	0.30	5.13E-01	NS	↓	-0.30	5.44E-01	NS	↓	-0.30	4.85E-01	NS	↑	0.17	9.17E-01	NS	↓	-0.01	7.85E-01	NS	↓	-0.16	8.04E-01	NS	↓	-0.15	8.04E-01	NS	↑	0.12	8.66E-01	NS	↓	-0.12	4.38E-01	NS	↑	0.22	9.28E-01	NS	↓	-0.06
M0R2W8	9.14E-01	NS	↑	0.16	8.29E-01	NS	↓	-0.51	9.30E-01	NS	↓	-0.10	1.01E-01	NS	↓	-1.59	6.30E-01	NS	↓	-0.69	9.78E-01	NS	↓	-0.20	7.30E-01	NS	↓	-0.10	9.71E-01	NS	↓	-0.33	9.22E-01	NS	↓	-0.16	6.24E-01	NS	↑	0.13	1.41E-01	NS	↓	-1.48	3.68E-01	NS	↑	0.31	8.55E-01	NS	↓	-0.29
D3DPG0	4.39E-01	NS	↓	-0.60	7.18E-01	NS	↑	0.05	4.22E-01	NS	↑	0.33	3.94E-01	NS	↓	-0.64	6.79E-01	NS	↓	-0.42	7.86E-01	NS	↓	-0.08	8.17E-01	NS	↑	0.01	7.62E-01	NS	↓	-0.35	7.99E-01	NS	↓	-0.28	8.24E-01	NS	↑	0.04	4.08E-01	NS	↓	-0.64	6.96E-01	NS	↑	0.12	8.84E-01	NS	↓	-0.06
D9YZU5	3.64E-01	NS	↓	-2.14	5.44E-01	NS	↑	0.33	5.03E-01	NS	↑	0.02	7.70E-01	NS	↓	-0.80	3.92E-01	NS	↑	0.84	2.98E-01	NS	↑	0.46	2.30E-01	NS	↑	1.42	7.61E-01	NS	↓	-0.40	7.22E-01	NS	↑	0.88	3.88E-01	NS	↓	-2.01	1.26E-01	NS	↓	-3.05	9.23E-01	NS	↓	-0.92	4.22E-01	NS	↓	-1.98
Q8NBH6	5.29E-01	NS	↓	-0.18	8.70E-01	NS	↑	0.00	9.31E-01	NS	↓	-0.03	3.10E-01	NS	↓	-0.33	7.99E-01	NS	↓	-0.07	6.83E-01	NS	↑	0.12	9.20E-01	NS	↑	0.08	3.37E-01	NS	↓	-0.29	4.44E-01	NS	↓	-0.24	7.54E-01	NS	↓	-0.10	5.15E-01	NS	↓	-0.19	7.02E-01	NS	↑	0.16	7.76E-01	NS	↓	-0.09
E9PIT3	8.25E-01	NS	↑	0.27	4.23E-01	NS	↓	-0.83	5.67E-01	NS	↓	-0.51	2.45E-01	NS	↑	0.87	4.01E-01	NS	↑	0.42	7.24E-01	NS	↓	-0.48	4.00E-01	NS	↓	-0.67	5.47E-01	NS	↑	0.12	6.35E-01	NS	↓	-0.44	3.33E-01	NS	↓	-0.65	3.08E-01	NS	↑	0.42	2.99E-01	NS	↓	-1.04	9.64E-01	NS	↓	-0.03
B2R7F8	7.67E-01	NS	↑	0.04	8.83E-01	NS	↑	0.01	5.93E-01	NS	↑	0.23	3.88E-01	NS	↑	0.28	9.60E-01	NS	↓	-0.07	7.93E-01	NS	↑	0.07	1.00E+00	NS	↓	-0.05	8.14E-01	NS	↑	0.06	9.54E-01	NS	↓	-0.05	8.84E-01	NS	↓	-0.07	1.33E-01	NS	↑	0.37	6.32E-01	NS	↓	-0.18	6.58E-01	NS	↑	0.13
K7EQQ3	9.63E-01	NS	↑	0.04	7.33E-01	NS	↓	-0.48	9.30E-01	NS	↓	-0.24	8.98E-01	NS	↓	-0.02	7.60E-01	NS	↑	0.19	9.61E-01	NS	↑	0.00	6.98E-01	NS	↑	0.07	7.58E-01	NS	↑	0.64	9.54E-01	NS	↑	0.59	5.58E-01	NS	↑	0.50	4.04E-01	NS	↓	-0.69	5.49E-01	NS	↓	-0.61	5.43E-01	NS	↑	0.48
B2RAN2	5.67E-01	NS	↑	0.35	7.10E-01	NS	↑	0.11	4.25E-01	NS	↓	-0.21	5.65E-01	NS	↓	-0.10	8.67E-01	NS	↑	0.02	2.64E-01	NS	↓	-0.30	1.91E-01	NS	↓	-0.41	7.31E-01	NS	↓	-0.04	3.28E-01	NS	↓	-0.26	7.88E-02	NS	↓	-0.56	4.35E-01	NS	↓	-0.17	5.34E-01	NS	↓	-0.15	1.73E-01	NS	↓	-0.48
B4DZ36	9.54E-01	NS	↑	0.16	9.10E-01	NS	↑	0.04	7.00E-01	NS	↑	0.39	6.94E-01	NS	↑	0.25	8.12E-01	NS	↓	-0.08	8.15E-01	NS	↑	0.20	7.51E-01	NS	↓	-0.01	8.46E-01	NS	↓	-0.05	7.06E-01	NS	↓	-0.15	2.04E-01	NS	↓	-0.53	6.48E-01	NS	↑	0.19	8.29E-01	NS	↓	-0.02	8.94E-01	NS	↑	0.06
A0A024RAA7	2.78E-01	NS	↓	-0.46	5.34E-01	NS	↓	-0.27	8.26E-01	NS	↑	0.04	3.03E-01	NS	↓	-0.44	4.94E-01	NS	↓	-0.29	7.17E-01	NS	↓	-0.17	4.13E-01	NS	↓	-0.35	5.40E-02	NS	↓	-0.90	6.29E-01	NS	↓	-0.15	6.94E-01	NS	↑	0.21	2.31E-01	NS	↓	-0.57	6.25E-01	NS	↑	0.18	7.71E-01	NS	↑	0.10
E7EQ64	9.31E-02	NS	↑	0.76	5.99E-01	NS	↑	0.40	9.95E-01	NS	↑	0.25	7.74E-01	NS	↑	0.27	5.09E-01	NS	↑	0.42	5.77E-01	NS	↑	0.46	3.35E-01	NS	↑	0.60	3.67E-01	NS	↑	0.96	7.62E-01	NS	↑	0.13	9.71E-01	NS	↑	0.50	3.91E-01	NS	↑	0.36	3.11E-01	NS	↑	0.73	4.00E-01	NS	↑	0.90
A0A087X1J7	7.61E-01	NS	↓	-0.19	7.35E-01	NS	↑	0.04	8.57E-01	NS	↓	-0.02	5.61E-01	NS	↓	-0.30	9.55E-01	NS	↓	-0.12	4.16E-01	NS	↑	0.21	7.11E-01	NS	↑	0.04	5.77E-01	NS	↓	-0.31	4.04E-01	NS	↓	-0.44	6.88E-01	NS	↑	0.20	7.73E-01	NS	↓	-0.20	4.31E-01	NS	↑	0.29	6.70E-01	NS	↑	0.13
Q05CV2	5.23E-01	NS	↓	-0.27	9.81E-01	NS	↓	-0.04	6.67E-01	NS	↑	0.30	6.51E-01	NS	↓	-0.20	3.78E-01	NS	↓	-0.36	6.87E-01	NS	↓	-0.17	7.49E-01	NS	↓	-0.11	2.94E-01	NS	↓	-0.43	6.69E-01	NS	↓	-0.14	6.57E-01	NS	↑	0.17	6.29E-01	NS	↑	0.14	7.69E-01	NS	↓	-0.11	4.86E-01	NS	↓	-0.30
B0YJC6	6.78E-01	NS	↑	0.20	4.93E-01	NS	↓	-0.31	9.45E-01	NS	↓	-0.02	8.17E-01	NS	↑	0.05	6.39E-01	NS	↑	0.28	6.34E-01	NS	↓	-0.16	7.06E-01	NS	↓	-0.21	9.03E-01	NS	↓	-0.07	3.72E-01	NS	↓	-0.42	2.24E-01	NS	↓	-0.60	8.79E-01	NS	↓	-0.03	4.09E-01	NS	↓	-0.39	9.32E-01	NS	↓	-0.04
Q6LAN8	9.07E-01	NS	↑	0.25	7.22E-01	NS	↓	-0.28	7.70E-01	NS	↓	-0.12	8.08E-01	NS	↑	0.66	9.92E-01	NS	↑	0.33	4.07E-01	NS	↓	-0.82	8.61E-01	NS	↓	-0.03	9.56E-01	NS	↑	0.22	8.93E-01	NS	↓	-0.10	7.85E-01	NS	↓	-0.32	8.62E-01	NS	↑	0.06	3.01E-01	NS	↓	-0.95	4.76E-01	NS	↓	-0.70
E9KL23	7.12E-01	NS	↑	0.29	9.93E-01	NS	↑	0.43	5.54E-01	NS	↑	0.05	6.06E-01	NS	↓	-0.29	9.77E-01	NS	↓	-0.24	7.13E-01	NS	↑	0.26	6.11E-01	NS	↑	0.69	4.98E-01	NS	↑	0.83	6.03E-01	NS	↑	0.28	4.19E-02	NS	↑	1.18	4.57E-01	NS	↑	0.32	2.07E-01	NS	↑	0.65	5.84E-01	NS	↑	0.25
A0A140VKF3	4.05E-01	NS	↓	-0.47	7.61E-01	NS	↓	-0.01	9.78E-01	NS	↓	-0.11	5.46E-01	NS	↓	-0.34	5.05E-01	NS	↓	-0.40	6.05E-01	NS	↑	0.07	7.68E-01	NS	↑	0.06	7.02E-01	NS	↓	-0.28	8.77E-01	NS	↓	-0.10	8.42E-01	NS	↑	0.10	4.61E-01	NS	↓	-0.43	3.69E-01	NS	↑	0.27	8.86E-01	NS	↓	-0.04
A0A024R035	7.67E-01	NS	↓	-0.07	9.53E-01	NS	↑	0.03	9.87E-01	NS	↑	0.05	9.14E-01	NS	↓	-0.02	9.68E-01	NS	↑	0.10	7.29E-01	NS	↑	0.17	5.88E-01	NS	↑	0.23	5.35E-01	NS	↑	0.18	7.27E-01	NS	↑	0.09	4.14E-01	NS	↑	0.17	7.22E-01	NS	↑	0.06	6.27E-01	NS	↑	0.16	9.44E-01	NS	↓	-0.03

Supplementary Table 4 (continue). Drug effects for IM across all time points versus pre-radiation (macaque database match)

	0.25 h/Pre			0.5 h/Pre			1 h/Pre			2 h/Pre			4 h/Pre			8 h/Pre			16 h/Pre			1 d/Pre			2 d/Pre			3 d/Pre			7 d/Pre			14 d/Pre			21 d/Pre		
**Uniprot Symbol**	*p-value*	*FDR*	*log2(FC)*	*p-value*	*FDR*	*log2(FC)*	*p-value*	*FDR*	*log2(FC)*	*p-value*	*FDR*	*log2(FC)*	*p-value*	*FDR*	*log2(FC)*	*p-value*	*FDR*	*log2(FC)*	*p-value*	*FDR*	*log2(FC)*	*p-value*	*FDR*	*log2(FC)*	*p-value*	*FDR*	*log2(FC)*	*p-value*	*FDR*	*log2(FC)*	*p-value*	*FDR*	*log2(FC)*	*p-value*	*FDR*	*log2(FC)*	*p-value*	*FDR*	*log2(FC)*
F6RKI4	2.54E-03	NS	3.61	1.47E-03	NS	4.05	1.02E-03	NS	4.10	3.34E-03	NS	3.03	6.84E-01	NS	0.50	7.79E-01	NS	0.53	7.22E-01	NS	0.67	5.20E-02	NS	2.87	4.17E-02	NS	2.14	6.27E-01	NS	0.10	5.97E-01	NS	0.36	4.20E-02	NS	1.47	2.91E-02	NS	2.64
F6TIK4	2.66E-03	NS	2.44	2.01E-03	NS	3.13	3.22E-04	NS	3.38	5.35E-03	NS	1.66	7.00E-02	NS	0.73	1.89E-02	NS	1.13	4.28E-02	NS	1.13	2.06E-02	NS	2.30	4.30E-01	NS	0.82	9.86E-01	NS	0.00	4.16E-01	NS	-0.43	4.48E-01	NS	0.21	1.06E-01	NS	1.52
F7H2A5	4.87E-03	NS	1.89	6.96E-03	NS	2.04	4.60E-04	NS	2.13	4.38E-02	NS	1.13	4.69E-01	NS	0.18	1.91E-01	NS	0.57	3.79E-01	NS	0.36	1.04E-01	NS	0.86	7.69E-01	NS	0.29	3.06E-01	NS	-0.48	4.22E-01	NS	-0.32	5.54E-01	NS	-0.16	1.05E-01	NS	1.05
F7HSL6	1.87E-01	NS	1.24	1.24E-02	NS	1.69	6.68E-02	NS	1.62	7.87E-02	NS	0.72	9.99E-01	NS	-0.10	2.45E-01	NS	0.51	4.14E-01	NS	-0.36	7.69E-01	NS	0.54	1.56E-01	NS	-0.67	1.49E-01	NS	-1.00	6.54E-01	NS	0.05	5.27E-01	NS	-0.25	5.56E-01	NS	0.36
F7CC91	1.25E-01	NS	-0.48	2.65E-01	NS	-0.38	9.10E-01	NS	0.04	1.32E-01	NS	-0.55	5.48E-02	NS	-0.54	5.96E-01	NS	0.10	9.06E-01	NS	0.03	4.57E-01	NS	-0.21	6.46E-01	NS	-0.13	7.46E-01	NS	-0.05	3.04E-01	NS	-0.31	3.60E-01	NS	-0.25	5.33E-01	NS	-0.17
F6S885	3.31E-01	NS	0.11	1.10E-01	NS	0.24	4.10E-01	NS	0.10	8.55E-01	NS	0.04	2.08E-01	NS	0.11	5.59E-01	NS	0.07	9.95E-01	NS	0.01	2.24E-02	NS	-0.57	1.09E-01	NS	-0.26	3.10E-01	NS	0.28	2.11E-01	NS	-0.28	1.69E-01	NS	0.27	4.94E-01	NS	0.18
F7EL64	7.68E-01	NS	0.23	3.76E-01	NS	0.23	3.29E-01	NS	0.38	5.40E-01	NS	-0.14	7.46E-01	NS	0.11	9.50E-01	NS	0.19	6.91E-01	NS	0.35	6.92E-01	NS	0.19	8.13E-01	NS	0.40	6.84E-02	NS	0.53	7.51E-01	NS	0.21	4.18E-01	NS	0.36	4.08E-01	NS	-0.30
F7GHR4	2.83E-01	NS	-0.24	4.87E-02	NS	-0.53	1.17E-01	NS	-0.41	9.48E-01	NS	0.05	8.59E-01	NS	-0.06	2.31E-01	NS	-0.43	7.39E-01	NS	-0.08	4.95E-01	NS	-0.17	3.08E-01	NS	-0.24	6.39E-01	NS	-0.12	7.47E-01	NS	-0.09	1.05E-01	NS	-0.57	3.94E-01	NS	-0.28
F6SG43	8.03E-01	NS	0.06	1.24E-01	NS	-0.38	1.68E-01	NS	-0.27	5.45E-01	NS	-0.10	5.31E-01	NS	-0.11	1.68E-01	NS	-0.26	2.08E-01	NS	-0.23	2.89E-01	NS	-0.30	2.05E-01	NS	-0.18	2.71E-01	NS	-0.26	1.98E-01	NS	-0.24	7.55E-02	NS	-0.30	8.00E-01	NS	-0.01
F6ZH99	2.46E-01	NS	1.33	5.54E-01	NS	0.38	1.18E-01	NS	1.36	1.09E-01	NS	2.07	1.02E-01	NS	1.29	5.56E-02	NS	2.32	6.63E-02	NS	2.27	5.25E-02	NS	1.81	1.32E-01	NS	1.31	2.46E-01	NS	0.93	1.12E-01	NS	1.79	1.86E-01	NS	0.97	7.40E-02	NS	1.54
A0A1D5QXZ0	4.86E-01	NS	0.48	8.71E-01	NS	0.15	6.31E-01	NS	0.17	2.39E-01	NS	0.27	9.78E-01	NS	0.04	2.59E-02	NS	-0.89	4.34E-01	NS	-0.30	4.36E-01	NS	-0.28	5.77E-01	NS	-0.08	6.69E-01	NS	-0.16	7.56E-01	NS	0.02	2.87E-01	NS	-0.47	5.75E-01	NS	-0.09
F7FQN1	5.37E-01	NS	0.47	8.90E-01	NS	0.15	5.50E-01	NS	0.32	9.69E-01	NS	0.40	7.96E-01	NS	0.13	6.10E-02	NS	-1.10	6.74E-01	NS	0.04	4.01E-01	NS	-0.34	6.62E-02	NS	-1.01	8.43E-02	NS	-0.85	1.58E-01	NS	-0.61	7.18E-01	NS	0.15	6.75E-01	NS	-0.06
F7DAC5	9.14E-01	NS	-0.02	5.07E-01	NS	-0.15	4.88E-01	NS	-0.18	1.32E-01	NS	-0.40	5.24E-01	NS	-0.15	3.16E-01	NS	-0.21	4.36E-01	NS	-0.18	3.31E-01	NS	-0.24	2.55E-01	NS	-0.31	7.30E-01	NS	0.10	2.28E-01	NS	-0.39	7.54E-01	NS	-0.03	8.46E-01	NS	0.01
F7DHR8	8.95E-01	NS	-0.06	1.21E-01	NS	-0.93	1.51E-01	NS	-0.84	2.70E-01	NS	-0.53	8.93E-01	NS	0.14	4.39E-01	NS	-0.28	6.40E-01	NS	-0.16	7.97E-01	NS	0.39	9.01E-01	NS	0.23	2.48E-01	NS	0.48	7.43E-01	NS	-0.18	4.14E-01	NS	0.39	6.73E-01	NS	0.15
F6T0W5	7.02E-01	NS	0.12	3.68E-01	NS	-0.27	3.29E-01	NS	-0.29	6.81E-01	NS	-0.12	3.10E-01	NS	0.42	6.03E-01	NS	-0.15	9.03E-01	NS	0.04	7.64E-01	NS	-0.09	2.25E-01	NS	-0.35	9.60E-01	NS	-0.02	4.27E-01	NS	-0.29	5.05E-01	NS	-0.20	7.30E-01	NS	-0.11
F6WIY2	3.74E-01	NS	0.34	6.81E-01	NS	0.25	2.55E-01	NS	0.40	7.33E-01	NS	0.09	4.73E-01	NS	-0.24	4.69E-01	NS	-0.24	3.17E-01	NS	-0.37	7.83E-01	NS	0.17	5.28E-01	NS	-0.22	2.89E-01	NS	-0.34	4.99E-01	NS	-0.23	6.44E-01	NS	0.19	3.09E-01	NS	0.56
A0A1D5QYD0	1.04E-01	NS	0.47	9.70E-02	NS	0.51	5.64E-02	NS	0.48	5.48E-01	NS	0.25	3.67E-01	NS	0.33	1.96E-01	NS	0.32	2.37E-01	NS	0.31	8.46E-01	NS	0.09	8.69E-01	NS	0.16	1.61E-01	NS	0.29	5.21E-02	NS	0.46	1.14E-01	NS	0.38	1.92E-01	NS	0.29
F6WR34	8.49E-01	NS	0.01	2.00E-01	NS	-0.38	7.07E-02	NS	-0.54	9.34E-01	NS	-0.04	5.36E-01	NS	-0.18	1.01E-01	NS	-0.48	3.63E-01	NS	-0.29	4.64E-01	NS	-0.20	3.55E-01	NS	-0.34	4.05E-01	NS	-0.23	5.52E-01	NS	-0.17	1.73E-01	NS	-0.35	6.09E-01	NS	-0.15
F6ZZL9	7.16E-01	NS	-0.06	2.52E-02	NS	-0.40	1.35E-01	NS	-0.36	9.42E-01	NS	0.01	6.40E-01	NS	-0.09	2.44E-01	NS	-0.27	3.17E-02	NS	-0.51	1.57E-01	NS	-0.23	1.16E-01	NS	-0.41	7.68E-02	NS	-0.41	5.41E-01	NS	-0.11	9.39E-02	NS	-0.35	2.06E-01	NS	-0.22
F7FJQ0	9.02E-01	NS	0.01	5.56E-02	NS	-0.51	4.71E-01	NS	-0.15	6.52E-01	NS	-0.08	4.51E-01	NS	-0.18	1.53E-02	NS	-0.43	2.03E-01	NS	-0.35	3.40E-01	NS	-0.20	3.42E-01	NS	-0.21	9.51E-02	NS	-0.37	3.73E-02	NS	-0.35	2.12E-01	NS	-0.26	1.90E-01	NS	-0.27
F7DNI9	6.96E-01	NS	0.08	1.24E-01	NS	0.67	2.91E-01	NS	0.48	2.83E-01	NS	0.60	5.29E-01	NS	0.35	2.44E-01	NS	0.40	9.72E-01	NS	-0.05	3.78E-01	NS	0.28	6.71E-01	NS	0.11	6.55E-01	NS	0.27	3.05E-01	NS	0.41	7.59E-01	NS	0.31	1.83E-01	NS	0.56
F6YK65	6.05E-01	NS	0.12	1.31E-01	NS	0.49	9.53E-02	NS	0.43	7.90E-01	NS	0.10	8.50E-01	NS	0.10	2.20E-01	NS	0.28	5.09E-01	NS	0.29	7.81E-01	NS	0.09	6.98E-01	NS	0.17	2.87E-01	NS	0.38	1.61E-01	NS	0.40	2.26E-01	NS	0.33	2.54E-01	NS	0.32
F6UR30	2.87E-01	NS	-0.54	1.27E-01	NS	-0.79	5.54E-01	NS	-0.26	1.01E-01	NS	-0.76	4.64E-01	NS	-0.33	7.00E-01	NS	-0.10	6.90E-01	NS	-0.20	9.75E-01	NS	0.21	6.35E-01	NS	0.49	5.99E-01	NS	0.27	7.20E-01	NS	-0.21	1.31E-01	NS	-0.74	4.26E-01	NS	0.39
F7FQN6	2.08E-01	NS	0.49	5.82E-01	NS	0.41	7.72E-01	NS	-0.39	7.42E-01	NS	0.11	7.49E-01	NS	0.22	2.58E-01	NS	0.93	6.51E-02	NS	1.26	3.34E-01	NS	0.62	1.25E-01	NS	0.98	2.29E-01	NS	1.01	4.11E-01	NS	0.66	3.66E-01	NS	0.44	6.44E-01	NS	0.25
A0A1D5QUU6	8.36E-01	NS	0.12	8.55E-02	NS	0.49	2.21E-01	NS	0.38	9.81E-01	NS	0.09	4.14E-01	NS	0.20	7.52E-02	NS	0.59	2.79E-01	NS	0.35	7.60E-01	NS	-0.10	8.14E-01	NS	-0.03	3.89E-01	NS	0.43	2.71E-01	NS	0.31	1.27E-01	NS	0.55	2.01E-01	NS	0.54
F6YW79	8.01E-01	NS	0.02	2.07E-01	NS	0.57	1.17E-01	NS	0.56	9.84E-01	NS	0.05	7.92E-01	NS	0.15	1.65E-01	NS	0.53	7.46E-01	NS	0.26	7.93E-01	NS	0.09	7.32E-01	NS	-0.14	6.05E-01	NS	0.23	3.58E-01	NS	0.30	2.67E-01	NS	0.35	3.27E-01	NS	0.29
A0A1D5QUE8	5.26E-01	NS	-0.22	2.05E-01	NS	-0.41	8.94E-01	NS	-0.07	9.64E-01	NS	-0.01	1.76E-01	NS	-0.44	6.41E-01	NS	0.12	6.72E-01	NS	-0.16	6.96E-01	NS	-0.15	8.23E-01	NS	-0.08	9.02E-01	NS	-0.05	7.09E-01	NS	0.05	5.72E-01	NS	-0.19	4.36E-01	NS	-0.28
F7GFB4	7.54E-01	NS	0.09	1.41E-01	NS	0.57	7.14E-02	NS	0.65	6.38E-01	NS	-0.18	9.48E-01	NS	0.10	1.91E-01	NS	0.50	5.38E-01	NS	0.41	4.15E-01	NS	0.28	3.35E-01	NS	0.32	3.44E-01	NS	0.43	3.00E-01	NS	0.40	9.54E-02	NS	0.57	4.60E-01	NS	0.18
A0A1D5QLP0	8.08E-01	NS	0.11	1.33E-01	NS	0.51	7.62E-02	NS	0.47	5.85E-01	NS	0.12	4.66E-01	NS	0.49	3.19E-02	NS	0.54	3.94E-01	NS	0.25	5.68E-01	NS	0.54	1.61E-01	NS	0.26	3.95E-01	NS	0.54	1.77E-01	NS	0.35	1.52E-01	NS	0.58	3.34E-01	NS	0.35
F6Y0N9	7.97E-01	NS	-0.08	3.55E-01	NS	0.31	5.25E-01	NS	0.09	9.23E-01	NS	0.08	8.66E-01	NS	0.00	5.31E-02	NS	0.69	1.40E-01	NS	0.39	4.50E-01	NS	0.27	8.02E-01	NS	0.08	1.69E-01	NS	0.65	6.94E-03	NS	0.77	3.11E-01	NS	0.23	7.17E-01	NS	0.10
F6U5Q9	9.62E-01	NS	0.01	1.22E-01	NS	0.55	1.54E-01	NS	0.59	5.48E-01	NS	0.18	5.05E-01	NS	0.28	2.90E-01	NS	0.55	4.27E-01	NS	0.94	9.51E-01	NS	-0.01	9.82E-01	NS	0.07	4.50E-01	NS	0.28	3.41E-02	NS	0.73	2.09E-01	NS	0.49	5.61E-01	NS	0.17
F7HF51	6.02E-01	NS	-0.19	2.34E-01	NS	0.52	2.22E-01	NS	0.58	6.74E-01	NS	-0.31	4.19E-01	NS	0.14	3.03E-01	NS	0.34	3.77E-01	NS	0.29	7.13E-01	NS	-0.26	8.60E-01	NS	-0.14	2.05E-01	NS	0.73	6.48E-01	NS	-0.08	5.63E-01	NS	0.28	3.26E-01	NS	0.32
F6W003	9.96E-01	NS	-0.04	5.46E-01	NS	-0.21	8.97E-01	NS	-0.08	1.59E-01	NS	-0.56	5.67E-01	NS	-0.23	6.10E-01	NS	-0.20	9.56E-01	NS	0.05	5.30E-01	NS	-0.25	1.29E-01	NS	-0.67	2.08E-01	NS	-0.51	3.35E-01	NS	-0.39	2.59E-01	NS	-0.50	2.43E-01	NS	-0.50
F7F6V9	5.75E-01	NS	0.25	2.28E-01	NS	0.65	3.05E-01	NS	0.45	9.93E-01	NS	0.03	6.19E-01	NS	0.12	3.69E-01	NS	0.34	4.94E-01	NS	0.31	6.20E-01	NS	0.21	9.41E-01	NS	0.09	4.83E-01	NS	0.42	3.17E-01	NS	0.34	1.95E-01	NS	0.61	2.61E-01	NS	0.67
F7H9D9	8.05E-01	NS	-0.08	5.83E-01	NS	-0.19	9.57E-01	NS	0.10	4.16E-01	NS	-0.35	6.83E-01	NS	-0.12	5.36E-01	NS	-0.24	9.58E-01	NS	-0.03	4.93E-01	NS	-0.27	3.20E-01	NS	-0.41	4.49E-01	NS	-0.31	4.40E-01	NS	-0.30	5.62E-01	NS	-0.20	5.18E-01	NS	-0.25
O19092	1.88E-01	NS	1.00	5.21E-01	NS	0.40	2.16E-01	NS	0.69	6.35E-01	NS	0.68	6.00E-02	NS	1.07	9.37E-01	NS	0.42	8.13E-01	NS	0.41	2.90E-01	NS	0.65	1.43E-01	NS	-0.98	2.95E-01	NS	0.32	1.26E-01	NS	1.11	3.31E-01	NS	0.33	5.76E-01	NS	0.60
F7BTD3	2.24E-01	NS	-0.29	7.23E-02	NS	-0.38	2.44E-02	NS	-0.37	1.80E-02	NS	-0.61	2.53E-01	NS	-0.23	3.22E-01	NS	-0.15	9.79E-01	NS	0.02	9.39E-01	NS	-0.01	1.37E-01	NS	-0.30	9.35E-01	NS	-0.01	1.89E-01	NS	-0.27	9.40E-01	NS	0.00	9.21E-01	NS	0.00
F7HM21	3.64E-01	NS	0.17	3.85E-01	NS	0.25	1.47E-01	NS	0.32	6.42E-01	NS	0.10	3.07E-01	NS	0.24	2.25E-01	NS	0.38	6.36E-02	NS	0.43	9.77E-02	NS	0.44	3.13E-01	NS	-0.34	4.14E-02	NS	0.47	2.27E-01	NS	0.42	2.53E-01	NS	-0.42	8.28E-01	NS	-0.06
F6UZ60	4.73E-01	NS	-1.11	6.65E-01	NS	-0.34	6.46E-01	NS	-0.77	4.75E-01	NS	-0.97	8.59E-01	NS	-0.06	7.93E-01	NS	-0.05	8.40E-01	NS	0.41	9.51E-01	NS	-0.25	7.94E-01	NS	-0.58	6.22E-01	NS	0.56	9.23E-01	NS	0.41	9.82E-01	NS	0.23	6.84E-01	NS	-0.85
F6XAC5	7.33E-01	NS	-0.15	3.29E-01	NS	-0.39	2.12E-01	NS	-0.48	6.43E-01	NS	0.24	7.28E-01	NS	-0.13	4.01E-01	NS	-0.31	1.83E-01	NS	-0.54	7.71E-01	NS	-0.14	5.00E-01	NS	-0.25	7.58E-01	NS	0.03	5.71E-01	NS	-0.20	3.03E-01	NS	-0.41	3.81E-01	NS	-0.35
F6VKN1	1.86E-01	NS	-0.85	2.20E-01	NS	-0.83	2.91E-02	NS	-1.41	2.65E-01	NS	-0.58	2.23E-01	NS	-0.77	3.27E-01	NS	-0.51	9.32E-01	NS	0.01	6.86E-02	NS	-1.20	1.73E-01	NS	-0.74	5.19E-01	NS	-0.21	6.61E-01	NS	0.35	7.84E-02	NS	-1.19	7.44E-01	NS	-0.22
F6RNZ2	8.33E-01	NS	-0.04	9.67E-01	NS	0.01	6.07E-01	NS	0.15	6.04E-01	NS	0.32	5.95E-01	NS	-0.13	9.65E-01	NS	0.03	8.99E-01	NS	-0.01	7.58E-01	NS	-0.06	8.90E-01	NS	0.07	9.40E-01	NS	0.01	7.67E-01	NS	-0.05	8.84E-01	NS	0.02	2.25E-01	NS	-0.27
A0A1D5R122	2.38E-01	NS	1.42	1.56E-01	NS	0.80	3.64E-01	NS	0.73	5.98E-01	NS	0.74	3.06E-01	NS	0.52	2.66E-01	NS	0.90	2.52E-01	NS	0.42	3.32E-01	NS	0.64	8.85E-01	NS	-0.07	5.68E-01	NS	0.40	3.11E-01	NS	1.16	5.63E-01	NS	0.43	3.95E-01	NS	0.76
F6SW75	9.08E-01	NS	0.05	4.28E-01	NS	0.30	2.75E-01	NS	0.37	4.78E-01	NS	0.31	4.38E-01	NS	0.12	4.09E-01	NS	0.25	6.82E-01	NS	0.13	8.70E-01	NS	0.02	6.31E-01	NS	-0.13	9.33E-01	NS	0.06	3.15E-01	NS	0.29	6.56E-01	NS	0.14	8.16E-01	NS	0.12
F7HT52	3.12E-01	NS	0.35	4.56E-01	NS	-0.35	3.65E-01	NS	-0.52	2.98E-01	NS	-0.59	9.32E-01	NS	-0.05	2.74E-01	NS	-0.63	7.82E-02	NS	-1.09	8.74E-01	NS	-0.01	4.93E-01	NS	-0.39	4.92E-01	NS	-0.38	6.00E-01	NS	-0.14	6.19E-01	NS	-0.29	2.32E-01	NS	-0.70
F7EVX1	9.52E-01	NS	-0.13	4.63E-01	NS	0.79	5.47E-01	NS	0.09	8.71E-01	NS	-0.38	8.72E-01	NS	-0.53	7.18E-01	NS	0.09	8.35E-01	NS	-0.01	8.05E-01	NS	-0.13	5.77E-01	NS	0.41	9.03E-01	NS	0.03	7.43E-01	NS	-0.61	4.85E-01	NS	0.60	6.49E-01	NS	0.19
F6WG91	7.99E-01	NS	-0.13	4.33E-01	NS	-0.30	5.34E-02	NS	-0.73	6.33E-01	NS	-0.18	3.85E-01	NS	-0.34	3.10E-01	NS	-0.39	2.61E-01	NS	-0.46	1.44E-01	NS	-0.54	6.30E-01	NS	-0.19	6.11E-01	NS	-0.20	2.37E-01	NS	-0.43	1.03E-01	NS	-0.61	4.62E-01	NS	-0.28
F6TLR3	8.59E-01	NS	0.05	7.05E-02	NS	0.39	1.12E-01	NS	0.33	6.89E-01	NS	-0.04	9.30E-01	NS	0.06	2.69E-02	NS	0.47	1.72E-01	NS	0.55	1.85E-01	NS	0.47	2.31E-01	NS	0.33	2.12E-01	NS	0.48	9.02E-01	NS	0.09	2.73E-01	NS	0.19	9.83E-01	NS	-0.01
F6YWS9	8.44E-01	NS	-0.13	6.97E-01	NS	-0.15	3.86E-01	NS	-0.33	9.61E-01	NS	-0.06	3.38E-01	NS	-0.38	7.74E-01	NS	-0.13	7.84E-01	NS	-0.13	7.08E-01	NS	-0.18	9.96E-01	NS	-0.05	6.76E-01	NS	0.08	7.53E-01	NS	-0.16	4.79E-01	NS	-0.29	5.29E-01	NS	-0.26
F7HL22	8.16E-01	NS	0.14	3.60E-01	NS	0.46	7.35E-01	NS	0.28	1.85E-01	NS	-0.87	7.31E-01	NS	0.27	4.35E-01	NS	-0.46	6.05E-01	NS	1.34	6.47E-01	NS	1.01	3.25E-01	NS	0.96	3.35E-01	NS	-0.59	2.16E-01	NS	-0.73	2.34E-01	NS	1.29	2.87E-01	NS	1.26
Q5NKV6	7.79E-01	NS	0.09	2.75E-01	NS	-0.31	1.31E-01	NS	-0.67	9.33E-01	NS	0.08	7.20E-01	NS	-0.08	1.88E-01	NS	-0.39	3.12E-01	NS	-0.35	2.19E-01	NS	-0.49	1.64E-01	NS	-0.39	3.45E-01	NS	-0.24	7.56E-01	NS	0.05	3.13E-01	NS	-0.31	9.71E-01	NS	0.02
I2CYL7	6.85E-01	NS	-0.10	3.89E-01	NS	-0.22	7.15E-01	NS	0.06	4.71E-01	NS	-0.19	2.93E-01	NS	-0.26	3.75E-01	NS	0.19	7.79E-01	NS	0.08	7.02E-01	NS	-0.10	4.14E-01	NS	-0.20	2.37E-01	NS	0.29	2.68E-01	NS	-0.33	4.43E-01	NS	-0.22	1.47E-01	NS	-0.35
A0A1D5Q6N1	9.12E-01	NS	-0.18	3.35E-01	NS	0.39	1.69E-01	NS	0.55	8.20E-01	NS	-0.15	9.03E-01	NS	-0.14	2.03E-01	NS	0.46	6.76E-01	NS	0.22	9.78E-01	NS	-0.09	9.44E-01	NS	-0.08	5.79E-01	NS	0.31	8.00E-01	NS	0.02	3.29E-01	NS	0.29	5.69E-01	NS	0.15
F6PJA1	4.21E-01	NS	-0.18	5.59E-01	NS	0.38	6.78E-01	NS	-0.07	9.10E-01	NS	0.66	2.35E-01	NS	-0.52	7.56E-01	NS	0.23	1.35E-01	NS	-0.42	8.13E-01	NS	0.06	4.61E-01	NS	-0.21	8.59E-01	NS	-0.03	3.64E-01	NS	-0.21	6.09E-01	NS	-0.12	2.90E-01	NS	0.80
F7HP21	9.95E-01	NS	-0.07	3.56E-01	NS	0.43	2.69E-01	NS	0.51	8.42E-01	NS	0.21	6.61E-01	NS	0.07	9.97E-02	NS	0.66	2.93E-01	NS	0.48	6.74E-01	NS	0.26	9.20E-01	NS	0.13	3.65E-01	NS	0.76	2.26E-01	NS	0.40	2.86E-01	NS	0.51	4.63E-01	NS	0.37
F7AEK9	3.67E-01	NS	-0.39	5.63E-01	NS	-0.19	1.65E-01	NS	-0.63	5.08E-01	NS	-0.27	5.89E-01	NS	-0.21	6.24E-01	NS	-0.13	8.06E-01	NS	0.09	7.73E-01	NS	0.20	9.29E-01	NS	0.66	3.65E-01	NS	-0.42	1.59E-01	NS	-0.61	1.44E-01	NS	-0.76	7.05E-01	NS	0.16
F6UM69	6.96E-01	NS	0.08	3.19E-01	NS	-0.45	1.80E-01	NS	-0.62	8.26E-01	NS	-0.15	7.99E-01	NS	-0.16	3.40E-01	NS	-0.43	5.98E-01	NS	-0.22	3.56E-01	NS	-0.42	5.19E-01	NS	-0.28	9.15E-01	NS	-0.04	6.23E-01	NS	-0.23	2.77E-01	NS	-0.46	6.20E-01	NS	-0.25
F7H312	7.30E-01	NS	0.41	1.92E-01	NS	-1.10	8.07E-01	NS	0.07	8.08E-01	NS	0.43	3.31E-01	NS	0.83	6.99E-01	NS	0.25	4.22E-01	NS	0.27	9.02E-01	NS	0.58	2.69E-01	NS	0.49	7.71E-01	NS	0.05	3.05E-01	NS	-0.63	4.50E-01	NS	-0.48	4.09E-01	NS	0.34
F7H532	9.33E-01	NS	-0.09	6.37E-01	NS	-0.19	4.56E-01	NS	-0.29	8.94E-01	NS	0.02	7.15E-01	NS	0.05	9.81E-01	NS	-0.03	8.48E-01	NS	0.06	5.95E-01	NS	0.10	5.11E-01	NS	-0.26	7.18E-01	NS	0.03	3.69E-01	NS	0.27	3.48E-01	NS	-0.34	7.91E-01	NS	0.06
F6YYK7	9.79E-01	NS	-0.05	3.79E-01	NS	0.27	4.89E-01	NS	0.19	6.19E-01	NS	-0.21	9.71E-01	NS	-0.08	2.38E-01	NS	0.35	6.86E-01	NS	0.11	8.37E-01	NS	-0.12	7.26E-01	NS	-0.17	6.03E-01	NS	0.27	7.76E-01	NS	-0.18	3.60E-01	NS	0.35	7.78E-01	NS	0.11
F7GHV1	4.87E-01	NS	-0.27	3.37E-01	NS	0.23	2.44E-01	NS	0.39	4.93E-01	NS	-0.26	7.91E-01	NS	-0.03	3.41E-01	NS	0.29	7.27E-01	NS	0.24	8.42E-01	NS	-0.04	7.57E-01	NS	0.12	3.35E-01	NS	0.45	8.85E-01	NS	0.03	2.26E-01	NS	0.32	4.90E-01	NS	0.20
F7HR41	5.12E-01	NS	-0.14	3.64E-01	NS	0.25	3.19E-01	NS	0.45	4.62E-01	NS	0.14	5.67E-01	NS	0.16	3.31E-01	NS	0.33	7.65E-01	NS	0.07	8.37E-01	NS	0.13	5.20E-01	NS	-0.13	8.46E-01	NS	0.09	9.62E-01	NS	-0.01	7.72E-01	NS	0.05	7.33E-01	NS	0.12
F7GAT4	4.45E-01	NS	-0.14	5.77E-01	NS	0.15	1.75E-02	NS	0.65	8.16E-01	NS	0.08	5.88E-01	NS	-0.09	5.75E-02	NS	0.54	7.99E-01	NS	0.32	8.38E-01	NS	0.07	9.19E-01	NS	0.05	4.46E-01	NS	0.24	1.76E-01	NS	0.44	5.93E-02	NS	0.38	8.73E-01	NS	0.02
F7F8G8	1.49E-01	NS	0.71	8.67E-01	NS	-0.07	1.46E-01	NS	0.72	2.68E-02	NS	1.25	2.87E-02	NS	1.86	3.94E-03	NS	2.68	1.68E-02	NS	2.00	8.03E-02	NS	2.78	3.09E-02	NS	2.00	3.64E-01	NS	0.27	3.67E-02	NS	-1.27	2.34E-01	NS	-0.46	3.80E-01	NS	-0.31
B3Y6B8	5.89E-01	NS	-0.13	9.96E-01	NS	-0.01	5.85E-01	NS	0.07	2.52E-01	NS	-0.33	7.05E-01	NS	-0.07	5.73E-01	NS	0.22	4.28E-01	NS	0.27	4.51E-01	NS	-0.15	8.86E-01	NS	0.02	4.34E-01	NS	0.24	3.12E-01	NS	-0.23	1.79E-01	NS	-0.46	2.89E-01	NS	-0.24
A0A1D5Q2R8	7.44E-01	NS	-0.09	2.49E-01	NS	0.37	5.94E-02	NS	0.44	1.11E-01	NS	-0.60	9.36E-01	NS	-0.01	9.15E-01	NS	0.16	8.91E-01	NS	0.01	8.42E-01	NS	-0.04	8.91E-01	NS	0.11	1.98E-01	NS	0.59	9.08E-01	NS	0.11	2.48E-02	NS	0.66	9.53E-01	NS	-0.02
F6Z0R5	6.48E-01	NS	-0.34	7.19E-01	NS	0.31	3.19E-01	NS	-0.63	3.83E-01	NS	-0.45	3.66E-01	NS	-0.71	9.33E-02	NS	-1.47	7.22E-02	NS	-1.56	1.61E-01	NS	-0.87	3.54E-01	NS	-0.65	4.06E-01	NS	-0.58	2.80E-01	NS	-0.86	4.68E-01	NS	-0.55	5.03E-01	NS	-0.58
A0A1D5QSH6	4.99E-01	NS	0.10	6.43E-01	NS	-0.11	1.89E-01	NS	-0.30	5.97E-01	NS	-0.13	5.03E-01	NS	-0.16	1.88E-01	NS	-0.34	5.84E-01	NS	-0.14	3.76E-01	NS	-0.24	3.62E-01	NS	-0.25	6.38E-01	NS	-0.11	9.65E-01	NS	0.00	2.03E-01	NS	-0.34	2.66E-01	NS	0.44
F7DIN9	8.72E-01	NS	0.03	2.65E-01	NS	0.28	2.60E-01	NS	0.36	6.28E-01	NS	-0.15	7.14E-01	NS	0.08	5.81E-01	NS	0.15	4.44E-01	NS	0.40	2.02E-01	NS	0.35	8.51E-01	NS	0.07	2.17E-01	NS	0.45	2.18E-01	NS	0.32	7.93E-01	NS	0.07	7.12E-01	NS	0.09
G7NQC3	5.17E-01	NS	-1.61	2.58E-01	NS	1.20	4.11E-01	NS	0.13	8.07E-01	NS	0.13	2.37E-01	NS	1.47	1.91E-01	NS	1.14	1.75E-01	NS	1.64	5.30E-01	NS	0.17	5.02E-01	NS	2.40	6.56E-01	NS	-1.07	4.39E-01	NS	-1.43	6.74E-01	NS	-0.08	8.40E-01	NS	-0.96
H9H3P6	9.61E-01	NS	-0.08	3.75E-01	NS	-0.32	2.38E-01	NS	-0.41	9.15E-01	NS	-0.08	8.12E-01	NS	-0.11	2.76E-01	NS	-0.38	6.46E-01	NS	-0.17	6.67E-01	NS	-0.18	7.31E-01	NS	-0.13	8.43E-01	NS	-0.11	6.27E-01	NS	-0.19	2.31E-01	NS	-0.40	6.98E-01	NS	-0.16
F7HQ03	9.83E-01	NS	-0.32	8.77E-01	NS	-0.20	9.54E-01	NS	-0.25	8.98E-01	NS	0.45	7.71E-01	NS	-0.47	4.72E-01	NS	0.21	8.36E-01	NS	0.06	7.10E-01	NS	-0.34	3.99E-01	NS	1.56	6.37E-01	NS	0.33	8.57E-01	NS	0.17	5.42E-01	NS	0.09	6.86E-01	NS	-0.38
F7HAH7	7.96E-01	NS	0.12	5.35E-01	NS	-0.25	4.46E-01	NS	-0.31	8.89E-01	NS	0.01	9.28E-01	NS	0.09	2.26E-01	NS	-0.51	4.54E-01	NS	-0.29	8.53E-01	NS	-0.05	5.94E-01	NS	-0.22	3.03E-01	NS	-0.39	6.39E-01	NS	-0.17	7.21E-01	NS	-0.13	6.48E-01	NS	-0.19
F7H263	4.45E-01	NS	0.55	1.04E-01	NS	0.35	4.37E-01	NS	0.19	1.06E-02	NS	0.92	4.58E-01	NS	0.36	6.24E-01	NS	0.23	6.20E-01	NS	-0.08	9.05E-01	NS	0.02	1.11E-02	NS	-0.39	6.49E-01	NS	0.07	6.18E-01	NS	0.39	9.99E-01	NS	0.04	9.21E-01	NS	0.15
A0A1D5QW71	6.72E-01	NS	0.14	6.22E-01	NS	-0.19	7.23E-01	NS	-0.15	9.80E-01	NS	-0.04	9.85E-01	NS	0.01	7.74E-01	NS	-0.11	7.99E-01	NS	-0.07	5.25E-01	NS	-0.25	1.95E-01	NS	-0.52	3.93E-01	NS	-0.33	9.97E-01	NS	-0.01	6.39E-01	NS	0.16	3.38E-01	NS	0.29
F6PRP0	9.17E-01	NS	-0.06	7.60E-01	NS	-0.17	6.79E-01	NS	-0.22	6.83E-01	NS	-0.24	8.44E-01	NS	-0.09	6.93E-01	NS	-0.22	9.92E-01	NS	-0.04	9.37E-01	NS	-0.04	9.52E-01	NS	-0.11	8.26E-01	NS	-0.17	4.69E-01	NS	-0.40	8.32E-01	NS	-0.12	7.44E-01	NS	-0.20
F6VZ47	8.97E-01	NS	0.09	3.46E-01	NS	-0.27	7.37E-01	NS	0.07	9.61E-01	NS	0.10	8.29E-01	NS	0.19	2.78E-01	NS	-0.34	5.52E-01	NS	-0.17	6.93E-01	NS	-0.06	2.16E-01	NS	-0.32	1.33E-01	NS	-0.45	4.80E-01	NS	-0.23	3.68E-01	NS	-0.27	9.85E-01	NS	0.02
F6Y7Q4	8.47E-01	NS	-0.15	4.82E-01	NS	0.24	3.32E-01	NS	0.35	7.96E-01	NS	-0.10	7.78E-01	NS	-0.18	4.94E-01	NS	0.18	7.72E-01	NS	0.03	8.68E-01	NS	0.14	8.58E-01	NS	-0.13	5.00E-01	NS	0.36	9.27E-01	NS	-0.06	6.41E-01	NS	0.12	8.85E-01	NS	-0.10
F7B8S4	7.37E-01	NS	0.11	8.96E-01	NS	-0.02	9.18E-01	NS	-0.15	3.35E-01	NS	-0.92	9.37E-01	NS	0.08	4.63E-01	NS	0.13	5.31E-01	NS	0.14	5.12E-01	NS	-0.37	6.96E-01	NS	-0.01	8.16E-01	NS	-0.23	5.24E-01	NS	0.27	5.70E-01	NS	-0.36	4.90E-01	NS	0.26
A0A1D5Q7U7	5.83E-01	NS	0.06	4.81E-01	NS	-0.07	9.73E-01	NS	0.02	1.82E-01	NS	0.15	1.29E-01	NS	-0.17	6.63E-01	NS	0.10	1.91E-01	NS	0.15	2.32E-01	NS	0.22	9.36E-02	NS	-0.17	5.37E-01	NS	0.08	5.10E-01	NS	0.29	8.62E-01	NS	-0.02	6.33E-01	NS	0.10
F7H1V9	6.15E-01	NS	-0.15	4.83E-01	NS	0.37	1.25E-01	NS	0.38	9.29E-01	NS	-0.05	9.40E-01	NS	0.06	7.25E-01	NS	0.24	8.04E-01	NS	-0.05	6.73E-01	NS	0.18	8.59E-01	NS	0.08	9.27E-01	NS	0.03	6.31E-01	NS	0.11	6.71E-01	NS	-0.13	7.57E-01	NS	-0.08
F7H5Q6	5.83E-01	NS	-0.35	6.39E-01	NS	0.07	4.22E-01	NS	0.21	2.58E-01	NS	-0.76	5.50E-01	NS	-0.40	4.63E-01	NS	0.16	7.89E-01	NS	0.05	5.24E-01	NS	-0.42	9.60E-01	NS	-0.09	6.60E-01	NS	-0.02	5.80E-01	NS	-0.37	1.92E-01	NS	0.47	5.36E-01	NS	0.31
F6SEN6	9.97E-01	NS	0.22	6.61E-01	NS	0.03	4.28E-01	NS	-0.20	3.93E-01	NS	-0.38	7.57E-01	NS	-0.26	7.42E-01	NS	0.12	7.86E-01	NS	0.41	8.22E-01	NS	0.50	3.51E-01	NS	0.43	4.23E-02	NS	0.92	7.18E-01	NS	0.11	2.74E-01	NS	0.42	9.52E-01	NS	0.00
F6UQI4	8.95E-01	NS	0.03	6.74E-01	NS	0.08	1.67E-01	NS	0.28	7.28E-01	NS	-0.08	8.60E-01	NS	-0.05	8.14E-01	NS	0.02	9.38E-01	NS	0.02	3.17E-01	NS	-0.19	4.59E-01	NS	-0.16	9.71E-01	NS	0.00	7.04E-01	NS	-0.09	6.21E-01	NS	0.07	9.85E-01	NS	0.00
A0A1D5QS06	3.52E-01	NS	-0.33	9.35E-01	NS	-0.07	7.12E-01	NS	0.10	7.07E-01	NS	-0.15	3.27E-01	NS	-0.36	8.18E-01	NS	-0.10	7.01E-01	NS	-0.12	1.99E-01	NS	-0.45	5.47E-01	NS	-0.21	7.20E-01	NS	-0.13	6.85E-01	NS	0.09	7.85E-01	NS	0.04	6.18E-01	NS	-0.18
F7DXU0	6.91E-01	NS	-0.78	8.61E-01	NS	-1.02	7.79E-01	NS	-1.07	7.68E-01	NS	-1.47	4.49E-01	NS	-1.71	2.88E-01	NS	-2.82	8.98E-01	NS	-0.69	8.05E-01	NS	-0.57	2.98E-01	NS	-2.65	9.52E-01	NS	0.33	3.11E-01	NS	-2.17	5.78E-01	NS	-0.28	6.41E-01	NS	-1.20
F6ZGR2	6.47E-01	NS	-0.27	6.68E-01	NS	0.08	7.22E-01	NS	0.02	4.95E-01	NS	-0.37	8.76E-01	NS	-0.17	3.25E-01	NS	0.27	8.44E-01	NS	0.01	8.99E-01	NS	-0.15	9.85E-01	NS	0.02	8.21E-01	NS	0.14	8.33E-01	NS	-0.19	3.20E-01	NS	0.29	7.03E-01	NS	0.06
F7DD08	7.09E-01	NS	-0.26	4.04E-01	NS	0.28	2.76E-01	NS	0.36	4.49E-01	NS	-0.44	7.24E-01	NS	-0.24	5.74E-01	NS	0.08	9.24E-01	NS	0.03	5.97E-01	NS	-0.33	6.88E-01	NS	-0.25	7.37E-01	NS	0.16	5.26E-01	NS	-0.38	4.35E-01	NS	0.21	9.05E-01	NS	-0.05
F6SSW9	6.84E-01	NS	0.08	8.73E-01	NS	0.04	6.60E-01	NS	0.06	1.86E-01	NS	0.40	6.42E-01	NS	0.15	9.15E-01	NS	0.00	7.59E-01	NS	-0.12	8.44E-01	NS	-0.09	3.91E-01	NS	-0.30	5.55E-01	NS	-0.21	1.84E-01	NS	0.34	9.53E-01	NS	-0.02	3.97E-01	NS	0.28
F7B786	7.85E-01	NS	-0.10	6.35E-01	NS	-0.33	8.53E-01	NS	-0.16	8.89E-01	NS	-0.09	8.68E-01	NS	0.23	7.02E-01	NS	0.18	4.06E-01	NS	0.39	4.19E-01	NS	0.57	5.80E-01	NS	1.22	2.47E-01	NS	0.45	3.56E-01	NS	-0.55	5.94E-01	NS	-0.35	5.70E-01	NS	0.32
F7FPV8	5.66E-01	NS	-0.37	6.12E-01	NS	0.18	5.32E-01	NS	0.19	9.27E-01	NS	0.31	4.73E-01	NS	-0.50	2.42E-01	NS	0.53	6.37E-01	NS	0.22	6.91E-01	NS	0.45	8.05E-01	NS	-0.04	3.53E-01	NS	0.84	8.65E-01	NS	-0.20	9.06E-01	NS	0.03	5.44E-01	NS	0.49
A0A1D5QKX6	8.77E-01	NS	-0.15	4.69E-01	NS	0.18	4.00E-01	NS	0.22	5.30E-01	NS	-0.40	7.23E-01	NS	-0.28	3.27E-01	NS	0.30	7.71E-01	NS	0.15	7.62E-01	NS	-0.26	9.18E-01	NS	-0.10	5.04E-01	NS	0.26	5.64E-01	NS	-0.39	1.23E-01	NS	0.63	2.28E-01	NS	0.50
A0A1D5QJP4	2.87E-01	NS	0.15	2.91E-01	NS	-0.16	1.82E-01	NS	-0.27	2.80E-01	NS	-0.19	3.07E-01	NS	-0.19	6.30E-03	NS	-0.42	2.22E-01	NS	-0.22	1.25E-01	NS	-0.30	1.03E-01	NS	-0.24	4.63E-01	NS	-0.12	4.19E-01	NS	-0.11	4.01E-01	NS	-0.12	7.50E-01	NS	-0.04
F6U2J6	8.90E-01	NS	0.03	4.76E-01	NS	-0.26	4.34E-01	NS	-0.27	7.20E-01	NS	0.05	9.25E-01	NS	-0.07	2.13E-01	NS	-0.46	3.86E-01	NS	-0.32	3.40E-01	NS	-0.32	2.60E-01	NS	-0.44	8.85E-01	NS	-0.08	7.05E-01	NS	-0.13	1.69E-01	NS	-0.45	4.27E-01	NS	-0.28
F7FY72	8.75E-01	NS	-0.05	1.02E-01	NS	-0.40	4.31E-01	NS	-0.18	9.19E-01	NS	0.04	9.57E-01	NS	0.03	1.57E-01	NS	-0.32	3.12E-01	NS	-0.21	6.40E-01	NS	-0.11	2.03E-01	NS	-0.38	2.48E-01	NS	-0.32	7.91E-01	NS	0.01	5.87E-01	NS	-0.13	8.32E-01	NS	-0.01
H9H2V9	7.30E-01	NS	-0.18	5.82E-01	NS	-0.32	1.98E-01	NS	-0.71	5.57E-01	NS	-0.28	3.40E-01	NS	-0.52	2.84E-01	NS	-0.66	5.51E-01	NS	-0.25	7.92E-01	NS	-0.14	6.19E-01	NS	-0.25	6.16E-01	NS	-0.28	7.88E-01	NS	-0.08	7.64E-01	NS	0.24	6.91E-01	NS	0.25
F6QJD8	7.13E-01	NS	0.17	8.91E-01	NS	-0.11	7.30E-01	NS	0.08	1.68E-02	NS	1.35	2.18E-02	NS	2.15	4.35E-03	NS	2.04	2.53E-02	NS	1.42	6.25E-02	NS	1.78	1.94E-02	NS	1.25	6.03E-01	NS	0.25	2.19E-01	NS	-0.77	4.77E-01	NS	-0.44	7.80E-01	NS	-0.15
G7MVP0	2.08E-01	NS	-1.69	2.35E-01	NS	-1.57	1.32E-01	NS	-1.94	5.56E-01	NS	-0.93	2.58E-01	NS	-1.52	4.91E-01	NS	-0.71	6.21E-01	NS	-0.63	4.60E-01	NS	-1.11	5.39E-01	NS	-0.68	9.62E-01	NS	0.40	2.12E-01	NS	-1.53	2.54E-01	NS	-1.47	5.86E-01	NS	-0.45
F6PLV7	5.38E-01	NS	0.48	2.94E-01	NS	0.55	3.03E-01	NS	0.61	7.16E-01	NS	0.08	9.88E-01	NS	-0.18	3.66E-01	NS	0.42	8.69E-01	NS	0.19	9.90E-01	NS	0.01	6.40E-01	NS	0.38	5.93E-01	NS	0.26	6.39E-01	NS	0.04	4.66E-01	NS	0.67	7.94E-01	NS	0.14
H9FXZ3	9.56E-02	NS	1.08	4.11E-01	NS	0.85	9.95E-01	NS	-0.03	2.91E-01	NS	0.47	3.40E-01	NS	0.59	7.89E-01	NS	-0.29	1.17E-01	NS	0.76	1.04E-01	NS	0.89	6.90E-01	NS	-0.13	5.41E-01	NS	0.27	3.01E-01	NS	0.50	9.83E-02	NS	1.06	6.07E-01	NS	-0.32
A0A1D5R9B8	7.92E-01	NS	0.49	5.59E-01	NS	-0.51	7.28E-01	NS	-0.45	6.33E-01	NS	-0.33	6.22E-01	NS	0.41	5.14E-01	NS	-0.58	9.56E-01	NS	-0.29	2.26E-01	NS	-1.02	2.34E-01	NS	-1.13	6.34E-01	NS	-0.44	5.72E-01	NS	0.10	6.51E-01	NS	-0.33	2.24E-01	NS	0.75
F7C9A4	6.33E-01	NS	0.12	7.72E-01	NS	0.06	9.32E-01	NS	-0.01	2.74E-01	NS	0.20	5.87E-01	NS	-0.13	9.48E-01	NS	0.01	6.93E-01	NS	-0.08	6.04E-01	NS	0.17	3.75E-01	NS	0.24	5.38E-01	NS	0.15	2.14E-01	NS	0.40	5.96E-01	NS	0.13	6.44E-01	NS	0.11
F7B749	5.94E-01	NS	0.12	3.94E-01	NS	0.22	5.61E-01	NS	0.32	9.79E-01	NS	0.04	8.41E-01	NS	-0.10	9.09E-01	NS	-0.01	5.73E-01	NS	0.14	6.68E-01	NS	0.08	7.42E-01	NS	-0.13	7.82E-01	NS	0.13	3.39E-01	NS	0.22	6.33E-01	NS	0.08	9.36E-01	NS	-0.01
F7B5G7	3.81E-01	NS	-0.49	8.68E-01	NS	-0.17	5.57E-01	NS	-0.44	9.02E-01	NS	0.17	9.85E-01	NS	0.27	7.28E-01	NS	0.36	3.70E-01	NS	0.55	3.69E-01	NS	0.56	5.43E-01	NS	1.81	7.76E-01	NS	-0.28	4.19E-01	NS	-0.55	3.03E-01	NS	-0.70	5.70E-01	NS	0.32
F7HCH2	6.04E-01	NS	1.55	4.82E-01	NS	-0.86	9.62E-01	NS	0.30	4.86E-01	NS	-0.81	4.82E-01	NS	2.72	9.86E-01	NS	-0.04	3.38E-01	NS	1.15	3.23E-01	NS	3.49	1.39E-01	NS	3.11	9.28E-01	NS	0.04	2.21E-01	NS	-1.34	4.14E-01	NS	2.14	5.31E-01	NS	2.05
F7CNK6	4.98E-01	NS	-0.23	7.29E-01	NS	-0.08	1.58E-01	NS	0.40	3.52E-01	NS	-0.30	2.95E-01	NS	-0.41	8.41E-01	NS	0.11	7.83E-01	NS	0.19	2.11E-01	NS	-0.41	7.19E-01	NS	-0.10	9.81E-01	NS	-0.01	7.89E-01	NS	0.13	2.54E-01	NS	0.30	3.96E-01	NS	0.31
A0A1D5Q3Z5	6.86E-01	NS	0.11	6.32E-01	NS	-0.13	1.44E-01	NS	-0.62	7.39E-01	NS	0.14	8.95E-01	NS	-0.01	2.62E-01	NS	-0.39	1.12E-01	NS	-0.54	3.69E-01	NS	-0.31	1.62E-01	NS	-0.51	6.26E-01	NS	-0.15	7.45E-01	NS	0.14	3.50E-01	NS	-0.31	6.89E-01	NS	-0.01
F7DNJ5	6.29E-01	NS	-0.12	4.34E-01	NS	0.13	3.54E-01	NS	0.26	7.25E-01	NS	-0.08	2.14E-01	NS	-0.33	3.21E-01	NS	0.31	6.19E-01	NS	0.15	8.31E-01	NS	0.13	9.76E-01	NS	0.04	2.51E-01	NS	0.33	5.19E-01	NS	-0.18	4.54E-01	NS	0.17	9.19E-01	NS	0.07
F7DH19	9.63E-01	NS	-0.04	6.81E-01	NS	-0.21	8.75E-01	NS	-0.08	9.09E-01	NS	0.04	9.14E-01	NS	-0.07	5.45E-01	NS	-0.32	6.65E-01	NS	-0.25	7.19E-01	NS	-0.20	3.56E-01	NS	-0.49	7.56E-01	NS	-0.19	6.08E-01	NS	-0.22	6.92E-01	NS	-0.22	7.47E-01	NS	-0.17
F7DX07	7.90E-01	NS	-0.02	5.70E-01	NS	0.14	5.97E-01	NS	0.11	9.35E-01	NS	0.41	3.00E-01	NS	-0.72	8.37E-01	NS	-0.09	7.54E-01	NS	0.19	3.97E-01	NS	0.27	2.16E-01	NS	-0.91	9.54E-01	NS	-0.18	3.09E-01	NS	0.45	7.73E-01	NS	-0.28	9.57E-01	NS	0.03
F6Z7P7	9.33E-01	NS	0.02	6.91E-01	NS	0.12	5.50E-01	NS	0.16	6.31E-01	NS	-0.48	7.71E-01	NS	-0.34	6.00E-01	NS	0.12	8.20E-01	NS	0.08	6.07E-01	NS	-0.52	9.67E-01	NS	0.12	5.10E-01	NS	0.35	9.45E-01	NS	-0.16	5.63E-01	NS	0.27	7.54E-01	NS	0.01
F6W3M2	6.90E-01	NS	-0.22	7.28E-01	NS	-0.23	5.46E-01	NS	-0.34	1.39E-01	NS	-0.73	2.62E-01	NS	-0.57	5.09E-01	NS	-0.35	4.67E-01	NS	-0.38	3.26E-01	NS	-0.50	8.94E-01	NS	-0.04	5.56E-01	NS	0.39	2.79E-01	NS	-0.59	9.70E-01	NS	-0.04	7.63E-01	NS	-0.11
F6WSG1	9.44E-01	NS	0.10	6.44E-01	NS	0.20	6.93E-01	NS	0.09	5.95E-02	NS	-0.97	7.16E-01	NS	-0.19	9.15E-01	NS	0.10	9.75E-01	NS	-0.05	3.43E-01	NS	-0.44	4.78E-01	NS	-0.33	3.43E-01	NS	0.57	1.81E-01	NS	-0.66	3.08E-01	NS	0.64	8.28E-01	NS	0.15
A0A1D5Q4R2	9.62E-01	NS	-0.03	6.94E-01	NS	-0.11	7.27E-01	NS	-0.11	4.57E-01	NS	0.36	6.48E-01	NS	0.11	9.80E-02	NS	-0.74	4.60E-01	NS	-0.26	5.16E-01	NS	-0.23	6.12E-01	NS	-0.16	5.28E-01	NS	-0.17	1.80E-01	NS	-0.58	9.36E-01	NS	0.08	4.16E-01	NS	0.18
F7BQW1	7.14E-01	NS	-0.12	8.10E-01	NS	0.26	7.90E-01	NS	-0.18	8.73E-01	NS	0.34	8.54E-01	NS	0.24	2.45E-01	NS	0.74	5.89E-01	NS	-0.28	7.24E-01	NS	0.28	8.37E-01	NS	0.46	3.70E-01	NS	1.06	6.46E-01	NS	0.15	2.85E-01	NS	0.61	5.81E-01	NS	0.46
F7FY55	5.57E-01	NS	0.50	6.17E-01	NS	-0.17	5.92E-01	NS	-0.18	7.31E-01	NS	-0.11	7.08E-01	NS	0.16	6.12E-01	NS	-0.17	9.36E-01	NS	0.03	6.94E-01	NS	-0.13	7.91E-01	NS	-0.09	5.36E-01	NS	-0.20	6.42E-01	NS	0.18	4.30E-01	NS	-0.27	9.61E-01	NS	0.00
F6XIG8	6.08E-01	NS	-0.18	6.87E-01	NS	0.07	8.31E-01	NS	0.04	4.09E-01	NS	-0.31	6.25E-01	NS	-0.15	5.69E-01	NS	0.12	5.99E-01	NS	-0.18	1.03E-01	NS	-0.56	4.47E-01	NS	-0.28	8.36E-01	NS	0.05	3.70E-01	NS	-0.32	3.26E-01	NS	0.22	5.56E-01	NS	0.11
F6Z0T5	8.59E-01	NS	0.50	1.10E-01	NS	2.85	3.24E-01	NS	1.88	5.13E-01	NS	1.50	5.36E-01	NS	1.50	8.51E-01	NS	0.72	4.96E-01	NS	0.80	1.93E-01	NS	-1.56	2.06E-01	NS	-1.50	5.25E-01	NS	1.00	1.93E-01	NS	1.49	8.75E-01	NS	0.71	1.33E-01	NS	1.91
F7DHQ1	2.29E-01	NS	0.57	4.10E-01	NS	0.92	2.80E-01	NS	0.74	6.86E-01	NS	0.31	6.75E-01	NS	1.03	2.54E-01	NS	1.59	3.30E-02	NS	2.15	6.40E-02	NS	1.55	4.64E-01	NS	1.99	2.35E-01	NS	0.70	3.27E-01	NS	0.53	2.15E-01	NS	-1.71	5.66E-01	NS	0.63
F6TNV9	5.90E-01	NS	-0.20	5.95E-01	NS	-0.20	5.67E-01	NS	-0.21	2.49E-01	NS	-0.45	5.30E-01	NS	-0.23	6.21E-01	NS	-0.19	3.47E-01	NS	-0.33	8.00E-01	NS	-0.13	7.64E-01	NS	-0.13	6.66E-01	NS	0.18	4.08E-01	NS	-0.32	4.44E-01	NS	0.29	6.21E-01	NS	0.10
F7HG52	9.69E-01	NS	-0.04	3.83E-01	NS	-0.26	5.71E-01	NS	-0.17	9.61E-01	NS	-0.03	6.07E-01	NS	-0.16	8.88E-01	NS	-0.07	7.23E-01	NS	-0.12	9.51E-01	NS	0.03	7.61E-01	NS	-0.11	8.43E-01	NS	0.01	3.59E-01	NS	0.18	8.25E-01	NS	-0.09	6.45E-01	NS	-0.15
F6TP36	9.55E-01	NS	-0.10	8.63E-01	NS	-0.04	4.92E-01	NS	0.20	5.69E-01	NS	-0.34	6.81E-01	NS	-0.28	7.42E-01	NS	0.00	9.82E-01	NS	0.04	5.78E-01	NS	-0.34	8.71E-01	NS	-0.06	9.33E-01	NS	-0.03	9.07E-01	NS	-0.11	4.77E-01	NS	0.18	8.30E-01	NS	-0.17
F7BCK9	6.17E-01	NS	0.36	5.88E-01	NS	-0.25	5.41E-01	NS	-0.30	8.50E-01	NS	0.29	9.99E-01	NS	0.21	4.27E-01	NS	-0.40	5.02E-01	NS	-0.31	8.92E-01	NS	-0.04	6.70E-01	NS	-0.22	2.95E-01	NS	-0.50	7.68E-01	NS	-0.16	6.54E-01	NS	-0.18	7.62E-01	NS	-0.16
G7NLH4	8.87E-01	NS	-0.34	2.79E-01	NS	0.61	8.96E-01	NS	-0.14	6.85E-01	NS	-0.56	7.26E-01	NS	-0.11	7.94E-01	NS	-0.42	6.24E-01	NS	-0.55	6.67E-02	NS	-1.78	7.53E-01	NS	-0.35	2.12E-01	NS	-1.20	3.10E-01	NS	-1.00	9.20E-01	NS	-0.33	3.96E-01	NS	-0.85
F7FHW4	5.32E-01	NS	-0.40	7.17E-01	NS	-0.22	9.79E-01	NS	-0.15	7.28E-01	NS	0.10	8.90E-01	NS	0.02	5.70E-01	NS	-0.37	3.91E-01	NS	-0.52	1.30E-01	NS	-0.90	1.74E-01	NS	-0.80	8.16E-01	NS	-0.25	8.96E-01	NS	-0.09	3.64E-01	NS	-0.54	8.32E-01	NS	-0.14
F7HFD0	9.07E-01	NS	0.20	9.69E-01	NS	0.34	9.25E-01	NS	-0.01	8.72E-01	NS	0.41	8.73E-01	NS	0.33	8.18E-01	NS	0.39	8.02E-01	NS	-0.02	6.21E-01	NS	0.05	8.23E-01	NS	-0.13	6.78E-01	NS	0.47	7.33E-01	NS	0.60	9.85E-01	NS	0.19	8.65E-01	NS	0.05
F6SCP6	4.81E-01	NS	0.35	9.15E-01	NS	-0.06	8.21E-01	NS	0.15	8.86E-01	NS	-0.12	6.91E-01	NS	-0.20	8.04E-01	NS	0.02	6.98E-01	NS	-0.16	5.69E-01	NS	0.26	2.32E-01	NS	-0.57	9.45E-01	NS	-0.06	4.61E-01	NS	-0.32	2.41E-01	NS	0.52	8.01E-01	NS	-0.17
F6SYK0	6.39E-01	NS	0.41	6.86E-02	NS	0.64	5.94E-01	NS	0.48	3.24E-01	NS	-0.36	6.95E-01	NS	-0.06	9.66E-01	NS	0.65	3.73E-02	NS	0.77	3.21E-02	NS	1.88	4.10E-01	NS	1.02	2.38E-01	NS	1.22	7.91E-01	NS	0.06	1.78E-01	NS	-0.50	1.02E-01	NS	-0.48
F6YJW2	3.33E-01	NS	-0.18	8.43E-01	NS	-0.04	5.42E-01	NS	0.15	8.90E-01	NS	0.05	5.60E-01	NS	-0.11	1.00E-01	NS	0.28	4.85E-01	NS	0.14	5.99E-01	NS	-0.10	6.84E-01	NS	-0.06	4.21E-01	NS	-0.12	6.86E-01	NS	0.22	4.32E-01	NS	0.13	8.65E-01	NS	-0.03
F7HQV3	6.04E-01	NS	-0.32	8.86E-01	NS	0.03	9.03E-01	NS	-0.16	6.75E-01	NS	-0.29	7.10E-01	NS	-0.27	9.35E-01	NS	-0.16	6.11E-01	NS	-0.31	5.46E-01	NS	-0.37	8.27E-01	NS	-0.18	9.80E-01	NS	-0.12	9.09E-01	NS	-0.16	7.56E-01	NS	0.00	8.72E-01	NS	-0.04
A0A1D5QD27	8.13E-01	NS	-0.02	6.74E-01	NS	-0.38	3.38E-01	NS	-0.77	8.83E-01	NS	-0.03	8.63E-01	NS	-0.03	6.27E-01	NS	-0.42	8.33E-01	NS	-0.06	8.66E-01	NS	-0.28	8.10E-01	NS	-0.19	6.28E-01	NS	0.07	9.12E-01	NS	-0.26	8.70E-01	NS	-0.32	7.68E-01	NS	-0.37
F7HCV8	5.05E-01	NS	0.20	8.74E-01	NS	0.10	4.04E-01	NS	-0.35	4.69E-01	NS	-0.31	9.53E-01	NS	0.13	9.56E-01	NS	0.04	7.30E-01	NS	0.29	7.85E-01	NS	0.15	5.99E-01	NS	0.35	8.68E-01	NS	-0.06	9.40E-01	NS	0.07	6.04E-01	NS	0.28	8.59E-01	NS	0.00
A0A1D5QCC0	7.23E-01	NS	-0.30	7.87E-01	NS	0.05	5.63E-01	NS	0.20	9.50E-01	NS	-0.15	8.71E-01	NS	-0.23	7.72E-01	NS	0.11	8.74E-01	NS	0.15	7.24E-01	NS	-0.37	8.26E-01	NS	-0.05	7.23E-01	NS	0.08	8.39E-01	NS	0.02	8.00E-01	NS	0.05	7.17E-01	NS	0.06
F6X848	9.79E-01	NS	0.09	8.64E-01	NS	0.04	5.06E-01	NS	0.41	5.51E-01	NS	0.15	8.34E-01	NS	-0.07	7.57E-01	NS	0.30	8.38E-01	NS	-0.02	6.60E-01	NS	-0.13	9.10E-01	NS	0.07	4.03E-01	NS	-0.28	9.67E-01	NS	0.02	6.33E-01	NS	-0.15	9.55E-01	NS	0.08
F7GRY2	8.73E-01	NS	-0.03	9.54E-01	NS	0.03	9.48E-01	NS	0.05	8.56E-01	NS	-0.02	9.94E-01	NS	0.09	6.60E-01	NS	0.17	5.05E-01	NS	0.23	7.12E-01	NS	0.09	6.62E-01	NS	0.14	2.42E-01	NS	0.27	6.46E-01	NS	0.11	5.84E-01	NS	0.18	9.76E-01	NS	-0.02
F7ALN6	2.94E-01	NS	-0.22	8.76E-01	NS	-0.02	6.07E-01	NS	0.09	4.29E-01	NS	-0.16	2.40E-01	NS	-0.20	6.37E-01	NS	0.06	9.67E-01	NS	0.02	2.02E-01	NS	-0.30	3.60E-01	NS	-0.19	9.20E-01	NS	0.02	8.19E-01	NS	-0.03	7.14E-01	NS	0.06	8.44E-01	NS	-0.03
F6WYB6	9.08E-01	NS	-0.07	7.50E-01	NS	-0.32	5.91E-01	NS	0.13	8.77E-01	NS	0.24	8.74E-01	NS	0.13	7.77E-01	NS	-0.63	7.64E-01	NS	-0.48	8.44E-01	NS	-0.26	7.02E-01	NS	-0.46	3.93E-01	NS	-1.29	8.09E-01	NS	-0.40	9.33E-01	NS	-0.10	8.12E-01	NS	-0.42
F6T094	8.84E-01	NS	0.19	6.34E-01	NS	0.23	9.06E-01	NS	0.23	3.67E-01	NS	-0.69	9.19E-01	NS	0.14	1.41E-01	NS	1.14	4.92E-02	NS	1.55	1.10E-01	NS	0.89	3.55E-01	NS	0.48	1.63E-01	NS	0.55	4.70E-01	NS	-0.53	3.96E-01	NS	0.44	7.75E-01	NS	-0.20
F7DLB8	7.58E-01	NS	-0.04	7.21E-01	NS	0.04	6.96E-01	NS	0.14	4.08E-01	NS	-0.90	9.01E-01	NS	-0.43	6.51E-01	NS	0.05	8.68E-01	NS	-0.02	5.21E-01	NS	-0.69	5.49E-01	NS	-0.42	9.90E-01	NS	-0.08	5.16E-01	NS	-0.78	9.10E-01	NS	-0.03	8.12E-01	NS	-0.48
F6SB17	7.17E-01	NS	0.15	7.26E-01	NS	-0.11	7.49E-01	NS	-0.11	8.55E-01	NS	-0.06	8.60E-01	NS	0.07	5.59E-01	NS	-0.19	6.11E-01	NS	-0.17	5.34E-01	NS	-0.20	2.95E-01	NS	-0.34	1.98E-01	NS	-0.48	9.47E-01	NS	0.00	5.77E-01	NS	-0.18	9.23E-01	NS	0.01
A0A1D5QF45	7.22E-01	NS	-0.32	8.69E-01	NS	0.08	8.55E-01	NS	-0.08	6.12E-01	NS	-0.50	7.15E-01	NS	-0.41	6.13E-01	NS	0.15	9.68E-01	NS	0.01	7.07E-01	NS	-0.40	8.18E-01	NS	-0.24	9.07E-01	NS	0.04	8.47E-01	NS	-0.23	9.12E-01	NS	0.04	8.53E-01	NS	-0.26
H9YUD6	5.72E-01	NS	0.28	5.27E-01	NS	-0.31	6.67E-01	NS	-0.12	9.12E-01	NS	0.05	6.89E-01	NS	0.21	5.59E-01	NS	-0.29	8.06E-01	NS	0.14	8.85E-01	NS	-0.01	8.56E-01	NS	0.06	2.61E-01	NS	-0.58	9.16E-01	NS	0.04	5.59E-01	NS	-0.29	9.50E-01	NS	-0.08
F7HEL0	3.12E-01	NS	-0.52	7.11E-01	NS	-0.25	6.63E-01	NS	0.09	3.75E-01	NS	-0.47	5.28E-01	NS	-0.33	8.99E-01	NS	-0.13	5.19E-01	NS	-0.34	6.63E-02	NS	-0.97	6.45E-01	NS	-0.17	6.55E-01	NS	0.27	2.91E-01	NS	-0.56	6.01E-01	NS	0.20	8.01E-01	NS	0.08
F7F4G6	8.35E-01	NS	0.05	5.35E-01	NS	-0.16	9.05E-01	NS	0.01	6.22E-01	NS	0.08	4.00E-01	NS	0.12	8.82E-01	NS	0.11	7.74E-01	NS	-0.07	2.51E-01	NS	0.26	6.72E-01	NS	0.10	2.11E-01	NS	0.32	4.99E-01	NS	0.11	2.56E-01	NS	0.32	1.77E-01	NS	0.27
F6SL92	5.09E-01	NS	-0.22	4.68E-01	NS	0.14	5.39E-01	NS	0.15	5.05E-01	NS	-0.22	6.46E-01	NS	-0.15	2.52E-01	NS	0.29	3.17E-01	NS	0.40	4.52E-01	NS	0.16	3.00E-01	NS	0.38	1.76E-01	NS	0.54	5.72E-01	NS	0.15	1.66E-01	NS	0.34	5.73E-01	NS	0.11
H9FY19	5.25E-01	NS	0.56	8.88E-01	NS	-0.04	7.74E-01	NS	0.14	4.94E-01	NS	0.22	8.22E-01	NS	0.16	5.45E-01	NS	-0.22	4.45E-01	NS	-0.29	8.75E-01	NS	-0.09	1.07E-01	NS	-0.59	3.90E-01	NS	-0.33	1.95E-01	NS	0.35	1.81E-01	NS	-0.49	8.82E-01	NS	-0.05
F7H7G9	6.60E-01	NS	0.07	7.02E-01	NS	-0.84	6.91E-01	NS	-0.55	7.44E-01	NS	-0.59	8.92E-01	NS	-1.65	7.30E-01	NS	-0.45	9.37E-01	NS	-0.67	6.44E-01	NS	0.62	5.49E-01	NS	0.23	6.61E-01	NS	0.65	9.04E-01	NS	-1.92	9.43E-01	NS	-1.03	7.84E-01	NS	-1.44
F7AV81	3.87E-01	NS	-2.01	5.61E-01	NS	0.42	5.59E-01	NS	0.10	8.24E-01	NS	-0.57	3.45E-01	NS	0.84	3.01E-01	NS	0.72	2.25E-01	NS	1.30	8.49E-01	NS	-0.53	7.68E-01	NS	1.08	3.63E-01	NS	-1.89	1.65E-01	NS	-2.54	8.95E-01	NS	-0.92	3.81E-01	NS	-1.95
F7F6Y0	6.41E-01	NS	0.11	7.79E-01	NS	-0.14	7.53E-01	NS	0.05	5.73E-01	NS	-0.24	8.67E-01	NS	-0.11	4.71E-01	NS	-0.31	3.67E-01	NS	-0.39	1.65E-01	NS	-0.58	3.84E-01	NS	-0.40	3.63E-01	NS	-0.37	8.02E-01	NS	-0.14	9.70E-01	NS	-0.04	7.37E-01	NS	-0.16
A0N064	6.03E-01	NS	0.14	9.34E-01	NS	-0.03	8.30E-01	NS	-0.08	4.12E-01	NS	0.40	9.97E-01	NS	0.02	5.34E-01	NS	-0.19	4.28E-01	NS	-0.24	9.39E-01	NS	0.01	4.92E-01	NS	-0.20	4.87E-01	NS	-0.21	6.36E-01	NS	0.10	7.21E-01	NS	-0.10	4.63E-01	NS	0.14
F7EI47	2.46E-01	NS	-0.23	1.71E-01	NS	-0.22	7.45E-01	NS	0.03	9.97E-01	NS	0.02	2.14E-01	NS	-0.18	3.72E-01	NS	0.24	9.54E-01	NS	0.02	2.25E-01	NS	-0.22	5.18E-01	NS	-0.11	3.10E-01	NS	-0.20	6.90E-02	NS	-0.25	4.44E-01	NS	-0.13	2.92E-01	NS	-0.17
A0A1D5R0L5	4.96E-01	NS	-0.36	8.17E-01	NS	0.08	6.82E-01	NS	0.08	2.53E-01	NS	-0.58	3.64E-01	NS	-0.46	8.95E-01	NS	-0.04	9.14E-01	NS	0.00	3.23E-01	NS	-0.48	4.83E-01	NS	-0.37	7.62E-01	NS	0.12	4.88E-01	NS	-0.36	9.79E-01	NS	-0.11	8.47E-01	NS	0.06

[a] Numbers are FDR adjusted *P* values. NS = not significant (FDR, *P* value > 0.05).

Supplementary Table 5.

Uniprot Symbol	0.25 h/Pre			0.5 h/Pre			1 h/Pre			2 h/Pre			4 h/Pre			8 h/Pre		
	p-value	*FDR*	*log2(FC)*	*p-value*	*FDR*	*log2(FC)*	*p-value*	*FDR*	*log2(FC)*	*p-value*	*FDR*	*log2(FC)*	*p-value*	*FDR*	*log2(FC)*	*p-value*	*FDR*	*log2(FC)*
B4E3P1	1.69E-02	NS	2.60	1.10E-02	NS	2.79	4.33E-02	NS	1.61	6.92E-02	NS	2.33	1.23E-02	NS	1.37	5.44E-02	NS	1.37
X6RJP6	1.57E-01	NS	2.90	7.69E-03	NS	3.22	2.16E-03	NS	3.21	2.16E-01	NS	1.68	7.27E-03	NS	1.88	2.33E-02	NS	1.72
I3L3D5	7.27E-02	NS	2.38	1.72E-01	NS	2.10	9.33E-03	NS	2.24	5.74E-02	NS	2.51	5.77E-02	NS	1.11	6.23E-02	NS	1.26
D0PNI1	7.37E-01	NS	0.52	2.36E-02	NS	2.12	4.96E-02	NS	0.70	5.62E-01	NS	0.97	9.38E-01	NS	0.30	2.01E-01	NS	0.63
Q53GK6	1.28E-02	NS	2.73	7.94E-02	NS	2.23	2.20E-02	NS	2.58	3.32E-02	NS	2.10	4.37E-02	NS	1.24	6.46E-03	NS	1.96
F2RM35	9.70E-02	NS	-0.56	3.39E-01	NS	-0.26	1.37E-01	NS	-0.45	2.81E-01	NS	-0.32	3.29E-01	NS	-0.18	2.42E-01	NS	-0.27
B4DRY0	9.23E-02	NS	0.57	7.57E-02	NS	4.64	1.17E-01	NS	1.18	1.58E-01	NS	1.22	3.96E-02	NS	0.76	7.43E-03	NS	1.36
Q53H26	5.73E-03	NS	-1.84	2.49E-01	NS	-0.55	2.35E-01	NS	-0.63	3.07E-01	NS	-0.47	3.47E-01	NS	-0.34	2.27E-01	NS	-0.54
RT-Cal protein	4.90E-01	NS	-0.07	4.19E-01	NS	0.08	5.39E-01	NS	-0.07	6.17E-01	NS	0.06	7.99E-01	NS	-0.03	4.33E-01	NS	-0.10
A0A0S2Z3D5	9.76E-02	NS	-0.73	3.17E-01	NS	-0.25	5.36E-01	NS	-0.15	4.03E-02	NS	-1.29	2.26E-01	NS	-0.62	4.56E-02	NS	-1.02
A0A024RAG6	4.53E-01	NS	0.42	3.38E-01	NS	-0.21	6.44E-01	NS	0.20	4.16E-01	NS	-0.23	8.80E-01	NS	-0.04	5.54E-01	NS	-0.03
A0A087WW43	1.88E-01	NS	0.32	5.64E-01	NS	0.08	1.19E-02	NS	0.43	6.49E-02	NS	0.42	6.57E-01	NS	0.22	3.13E-02	NS	0.35
B1AP58	1.57E-01	NS	0.52	9.19E-01	NS	-0.04	1.25E-01	NS	0.43	9.51E-02	NS	0.73	7.39E-01	NS	0.17	1.04E-01	NS	0.82
B2R888	8.70E-01	NS	0.07	7.05E-01	NS	-0.06	1.49E-01	NS	1.03	9.52E-02	NS	0.45	1.32E-01	NS	0.32	3.81E-02	NS	0.75
A0A024R962	7.43E-01	NS	-0.27	7.78E-01	NS	0.01	1.50E-01	NS	-0.96	1.73E-01	NS	-0.94	8.35E-01	NS	-0.04	9.11E-02	NS	-1.21
D9ZGG2	2.93E-01	NS	-0.19	4.76E-01	NS	-0.12	5.83E-02	NS	-0.36	2.75E-01	NS	-0.19	3.44E-01	NS	-0.20	8.87E-02	NS	-0.35
B7Z549	2.08E-01	NS	0.34	5.95E-01	NS	-0.10	9.85E-02	NS	0.56	2.61E-01	NS	0.54	4.76E-01	NS	-0.14	3.63E-01	NS	0.46
A0A024RAA7	8.68E-01	NS	0.03	5.23E-01	NS	-0.16	9.72E-01	NS	0.06	9.44E-01	NS	-0.03	5.50E-01	NS	0.12	9.58E-01	NS	0.08
D6R934	8.37E-01	NS	0.10	8.15E-02	NS	-0.51	2.28E-01	NS	0.58	6.37E-01	NS	-0.14	9.10E-01	NS	0.08	7.42E-01	NS	0.15
Q8IVC0	3.87E-01	NS	0.17	1.98E-01	NS	-0.40	1.74E-01	NS	0.26	5.31E-01	NS	0.16	9.06E-01	NS	0.06	6.72E-01	NS	0.09
A0A140VKF3	7.90E-01	NS	0.10	6.97E-01	NS	-0.08	3.73E-01	NS	0.50	4.55E-01	NS	0.19	5.48E-01	NS	0.17	9.13E-02	NS	0.54
B3KT06	3.72E-02	NS	2.28	1.96E-02	NS	4.45	9.45E-02	NS	2.91	6.52E-02	NS	2.34	6.29E-01	NS	0.63	8.09E-01	NS	0.44
E9PIT3	9.15E-01	NS	0.07	3.26E-01	NS	0.41	1.16E-01	NS	-1.50	6.66E-01	NS	-0.36	8.41E-01	NS	0.43	2.44E-01	NS	-1.09
V9HWA9	4.99E-02	NS	0.53	9.78E-01	NS	-0.01	3.49E-01	NS	0.40	1.80E-01	NS	0.45	9.40E-01	NS	0.03	1.33E-01	NS	0.42
D3DPG0	6.87E-01	NS	0.23	7.43E-01	NS	-0.15	3.10E-01	NS	0.52	5.06E-01	NS	0.24	6.87E-01	NS	-0.17	3.09E-01	NS	0.42
Q5VY30	9.01E-01	NS	-0.04	9.62E-02	NS	0.48	2.07E-01	NS	-0.46	7.33E-01	NS	-0.11	6.33E-01	NS	0.31	3.00E-01	NS	-0.38
V9HW98	4.53E-01	NS	-0.26	2.72E-01	NS	0.56	8.78E-01	NS	0.20	9.60E-01	NS	0.16	6.69E-01	NS	0.00	2.46E-01	NS	-0.46
V9HWE9	1.02E-01	NS	1.00	1.18E-01	NS	1.05	1.65E-01	NS	2.73	9.05E-01	NS	0.10	5.44E-02	NS	1.39	2.02E-01	NS	2.22
A5PL27	3.13E-01	NS	0.57	9.60E-01	NS	-0.16	1.44E-01	NS	0.64	9.84E-01	NS	0.37	7.90E-01	NS	0.53	1.00E+00	NS	-0.03
G3V2W1	3.91E-01	NS	0.24	9.23E-01	NS	-0.02	3.77E-01	NS	-0.39	6.37E-01	NS	0.03	9.46E-01	NS	-0.07	4.33E-01	NS	-0.32
M0R2W8	5.26E-01	NS	-0.35	3.28E-01	NS	-0.54	9.57E-01	NS	0.16	4.62E-01	NS	0.28	7.11E-01	NS	-0.18	2.19E-01	NS	0.56
B2R815	6.16E-01	NS	0.18	5.49E-01	NS	-0.20	1.68E-01	NS	0.47	4.26E-01	NS	0.23	5.71E-01	NS	-0.18	4.36E-01	NS	0.36
A0A0G2JPR0	1.95E-01	NS	0.31	6.81E-01	NS	0.09	3.87E-01	NS	0.17	3.04E-01	NS	0.26	6.13E-01	NS	0.12	4.06E-01	NS	0.31
B4DDT3	9.05E-01	NS	0.03	1.25E-01	NS	-0.45	3.04E-01	NS	0.92	9.84E-01	NS	0.01	5.30E-01	NS	-0.20	4.52E-01	NS	0.85
B7Z555	5.74E-01	NS	0.17	2.09E-01	NS	-0.28	1.77E-01	NS	0.62	6.24E-01	NS	0.19	8.21E-01	NS	-0.02	6.79E-01	NS	0.09
B4DPQ0	9.12E-01	NS	-0.06	8.20E-01	NS	0.01	1.16E-01	NS	0.45	3.01E-01	NS	0.20	8.96E-01	NS	-0.04	1.88E-01	NS	0.36
Q59GS8	3.39E-01	NS	0.35	7.82E-01	NS	0.04	3.92E-01	NS	0.23	4.22E-01	NS	0.23	7.83E-01	NS	0.10	6.29E-01	NS	0.11
F8W876	6.00E-01	NS	0.32	5.14E-01	NS	0.17	1.56E-01	NS	0.68	9.15E-01	NS	0.01	2.60E-01	NS	0.35	8.53E-01	NS	0.03
F5GXS0	2.76E-01	NS	0.42	9.38E-01	NS	-0.02	2.57E-01	NS	0.35	1.56E-01	NS	0.44	5.80E-01	NS	0.15	4.20E-01	NS	0.25
A2RTY6	3.22E-01	NS	0.32	4.85E-01	NS	-0.22	1.08E-01	NS	0.45	2.52E-01	NS	0.40	8.61E-01	NS	0.04	3.29E-01	NS	0.32
A0A024R3E3	2.09E-01	NS	-1.94	3.29E-01	NS	-0.53	2.29E-01	NS	-1.68	1.80E-01	NS	-1.99	3.67E-01	NS	-1.36	3.71E-01	NS	-1.46
B2R4M6	9.70E-01	NS	0.22	1.75E-01	NS	0.95	1.86E-01	NS	0.89	2.41E-01	NS	1.02	2.35E-02	NS	1.57	6.71E-03	NS	2.12
B3KNX0	1.59E-01	NS	-0.79	3.88E-01	NS	-0.33	2.96E-02	NS	0.76	6.02E-01	NS	-0.21	3.07E-01	NS	0.35	4.34E-01	NS	-0.29
L8E853	5.14E-01	NS	0.14	2.07E-01	NS	-0.32	6.47E-01	NS	0.80	8.32E-01	NS	0.14	2.86E-01	NS	0.37	8.09E-01	NS	0.28
Q59E93	5.72E-01	NS	0.10	9.16E-01	NS	0.12	6.96E-02	NS	1.78	8.73E-02	NS	0.64	2.62E-01	NS	0.67	2.98E-01	NS	1.37
B3KS79	9.20E-01	NS	-0.01	3.36E-01	NS	-0.38	2.48E-01	NS	0.33	9.54E-01	NS	0.01	8.85E-01	NS	0.11	3.99E-01	NS	0.18
A0A384MDV8	7.59E-01	NS	-0.07	5.89E-01	NS	0.15	4.90E-01	NS	-0.20	9.97E-01	NS	0.09	4.68E-01	NS	-0.20	7.93E-01	NS	0.20
B2RA39	2.19E-02	NS	-0.68	2.34E-01	NS	0.56	7.20E-01	NS	1.13	6.91E-01	NS	-0.04	5.02E-01	NS	-0.19	1.23E-01	NS	0.49
B2CIS9	5.67E-01	NS	0.11	3.58E-01	NS	0.23	3.83E-01	NS	0.83	4.26E-01	NS	0.70	8.23E-01	NS	0.00	3.29E-01	NS	0.37
H0YAC1	6.78E-01	NS	-0.08	8.86E-01	NS	0.04	5.74E-01	NS	0.12	4.42E-01	NS	-0.16	8.84E-01	NS	-0.02	2.79E-01	NS	-0.25
Q8TAY0	6.22E-01	NS	0.31	6.69E-01	NS	-0.25	3.63E-01	NS	0.32	4.03E-01	NS	0.26	8.30E-01	NS	-0.17	8.10E-01	NS	0.08
H6VRG1	4.05E-01	NS	0.39	4.62E-01	NS	0.61	9.29E-02	NS	0.73	7.26E-01	NS	-0.02	3.15E-01	NS	0.89	1.12E-01	NS	1.02
B7ZLE5	4.44E-02	NS	0.39	1.52E-01	NS	0.43	8.62E-02	NS	1.19	4.95E-03	NS	0.61	5.88E-01	NS	0.38	4.04E-02	NS	0.68
A6XND1	1.94E-01	NS	-0.35	7.39E-01	NS	0.04	2.13E-01	NS	-0.36	3.87E-01	NS	-0.24	8.67E-01	NS	0.01	2.05E-01	NS	-0.35
F8W0J6	6.63E-01	NS	0.37	2.80E-01	NS	0.79	5.93E-01	NS	0.65	3.11E-01	NS	1.00	8.70E-02	NS	1.34	2.69E-01	NS	0.57
A0A140VJI7	1.73E-01	NS	-0.58	4.11E-01	NS	-0.36	5.13E-01	NS	-0.29	1.16E-01	NS	-0.70	9.78E-01	NS	-0.05	2.20E-01	NS	-0.53
A0A087WSY5	7.13E-01	NS	0.13	5.92E-01	NS	-0.12	7.54E-01	NS	-0.05	2.88E-01	NS	0.28	7.12E-01	NS	-0.09	7.60E-01	NS	0.17
A0N071	3.09E-01	NS	1.22	3.94E-01	NS	1.28	4.57E-02	NS	1.50	5.86E-02	NS	1.57	1.03E-01	NS	1.31	4.06E-02	NS	2.91
Q53H37	1.81E-01	NS	-1.68	9.46E-01	NS	-0.02	7.43E-01	NS	-0.46	2.46E-01	NS	-1.16	4.62E-01	NS	-0.84	3.87E-01	NS	-1.17
J3QR68	5.77E-01	NS	-0.01	4.55E-01	NS	-1.07	5.72E-01	NS	-0.70	6.15E-01	NS	-0.46	7.41E-01	NS	-0.81	6.64E-01	NS	-0.18
A8K1K1	6.57E-01	NS	0.16	7.84E-01	NS	-0.29	5.88E-01	NS	0.46	6.43E-01	NS	0.31	7.32E-01	NS	-0.36	4.76E-01	NS	0.57
A0A024R9Q1	2.25E-01	NS	0.60	5.18E-01	NS	0.36	5.22E-01	NS	0.40	6.50E-01	NS	0.48	8.60E-01	NS	0.08	8.17E-01	NS	0.09
E7END6	3.45E-01	NS	-0.27	9.42E-01	NS	0.06	2.08E-01	NS	-0.29	6.64E-01	NS	-0.11	9.82E-01	NS	0.07	1.61E-01	NS	-0.36
B2R950	8.59E-01	NS	0.05	9.03E-01	NS	0.00	4.09E-01	NS	0.42	5.96E-01	NS	0.29	6.44E-01	NS	-0.13	9.50E-01	NS	0.16
A0A024R1X6	5.78E-01	NS	0.35	6.08E-02	NS	4.25	3.03E-01	NS	0.54	2.12E-01	NS	0.83	2.49E-01	NS	0.63	6.11E-01	NS	0.78
I3L145	9.53E-01	NS	0.02	5.34E-01	NS	0.34	5.54E-02	NS	0.66	4.23E-01	NS	0.31	9.76E-01	NS	0.04	4.60E-01	NS	0.37
Q4LE79	7.89E-01	NS	0.20	4.00E-01	NS	1.78	9.87E-01	NS	0.86	2.94E-01	NS	0.94	7.05E-01	NS	0.50	8.78E-01	NS	0.11
Q499G5	4.50E-03	NS	0.53	9.84E-01	NS	0.01	2.82E-01	NS	-0.56	1.16E-01	NS	0.60	8.95E-01	NS	0.03	8.15E-01	NS	0.08
S4R471	6.61E-01	NS	0.17	2.58E-01	NS	0.41	4.40E-01	NS	0.39	1.16E-01	NS	0.57	5.84E-01	NS	0.25	2.63E-01	NS	0.45
V9HWF6	4.14E-01	NS	-0.97	8.04E-01	NS	-0.12	4.42E-01	NS	0.05	4.82E-01	NS	-0.13	5.03E-01	NS	-1.07	6.35E-01	NS	-0.53
B7Z8Q5	6.40E-01	NS	-0.63	5.47E-01	NS	0.74	3.12E-01	NS	0.46	2.05E-01	NS	0.83	2.01E-01	NS	0.97	8.34E-01	NS	0.25
P78492	5.66E-02	NS	0.45	3.95E-01	NS	0.19	8.02E-01	NS	0.06	3.60E-01	NS	0.30	1.66E-01	NS	0.31	7.21E-01	NS	0.08
V9HWP0	9.48E-01	NS	-0.11	7.58E-01	NS	0.26	6.45E-01	NS	0.33	4.33E-01	NS	0.19	8.05E-01	NS	-0.13	7.77E-01	NS	0.12
V9HW68	9.48E-02	NS	-1.39	4.00E-01	NS	-0.48	8.94E-01	NS	-0.38	2.49E-01	NS	-1.08	2.07E-01	NS	-1.05	4.94E-01	NS	-0.64
V9HWE3	9.21E-01	NS	-0.01	1.92E-01	NS	-0.82	6.47E-01	NS	-0.04	3.99E-01	NS	0.54	1.20E-01	NS	0.62	2.81E-01	NS	0.59
C9JEU5	9.82E-02	NS	1.97	1.24E-02	NS	2.22	3.50E-01	NS	2.46	1.80E-01	NS	1.60	2.14E-01	NS	1.59	5.62E-02	NS	1.93
K7ERE3	7.00E-01	NS	0.00	8.28E-01	NS	2.46	4.14E-01	NS	0.47	9.60E-01	NS	-0.77	3.03E-01	NS	0.47	3.43E-01	NS	0.98
A0A0U1RR20	6.72E-01	NS	-0.11	4.26E-01	NS	-0.25	2.08E-01	NS	-0.75	5.30E-01	NS	-0.20	2.16E-01	NS	-0.67	4.64E-01	NS	-0.21
E3UN46	4.39E-01	NS	-0.31	4.60E-01	NS	0.18	3.41E-01	NS	0.38	9.30E-01	NS	0.02	9.44E-01	NS	-0.01	3.37E-01	NS	0.28
Q5JVE7	7.48E-03	NS	-0.17	2.37E-02	NS	-0.40	8.79E-01	NS	0.01	2.02E-01	NS	-0.33	2.05E-01	NS	-0.15	4.91E-02	NS	-0.42
J3K000	5.16E-01	NS	0.42	7.30E-01	NS	-0.17	4.47E-01	NS	0.45	2.35E-01	NS	0.50	7.52E-01	NS	0.02	6.53E-01	NS	0.18
Q59EP2	6.30E-01	NS	0.17	9.90E-01	NS	-0.04	2.25E-01	NS	0.32	2.48E-01	NS	0.29	9.82E-01	NS	-0.03	1.09E-01	NS	0.41
K7EQQ3	5.49E-01	NS	0.28	3.67E-01	NS	0.92	5.64E-01	NS	0.43	5.71E-01	NS	-0.07	4.62E-01	NS	1.09	4.82E-01	NS	0.62
H3BRJ9	2.18E-01	NS	-1.00	4.05E-02	NS	-1.71	2.13E-01	NS	1.45	5.11E-01	NS	-0.35	8.96E-01	NS	-0.17	2.36E-01	NS	-0.69
Q5SQ08	2.80E-01	NS	0.24	7.97E-01	NS	-0.02	2.60E-01	NS	0.22	7.19E-02	NS	0.40	2.11E-01	NS	-0.21	1.91E-01	NS	0.25
Q6MZL2	5.69E-01	NS	0.15	5.36E-01	NS	0.12	5.07E-01	NS	0.86	4.52E-01	NS	0.17	9.45E-02	NS	0.31	7.65E-01	NS	0.32
Q13784	2.87E-01	NS	-0.68	6.75E-01	NS	-0.32	5.93E-01	NS	-0.39	3.71E-01	NS	-0.59	4.56E-01	NS	-0.48	4.06E-01	NS	-0.54
C0JYY2	9.60E-01	NS	0.01	2.47E-01	NS	-0.35	4.87E-01	NS	0.28	5.38E-01	NS	-0.18	8.11E-01	NS	-0.07	7.65E-01	NS	0.08
A0A0S2Z4I5	5.55E-01	NS	-0.27	7.43E-01	NS	-0.06	8.17E-01	NS	0.03	3.67E-01	NS	-0.41	2.98E-01	NS	0.32	9.01E-01	NS	0.04
D9IWP9	5.54E-01	NS	-0.23	6.80E-01	NS	0.09	5.33E-01	NS	-0.25	6.56E-01	NS	-0.18	8.92E-01	NS	0.11	4.29E-01	NS	-0.29
B2R773	8.13E-01	NS	0.24	7.86E-01	NS	0.14	4.09E-01	NS	0.55	5.59E-01	NS	0.38	9.22E-01	NS	-0.08	5.62E-01	NS	0.66
E9PHK0	6.92E-02	NS	-0.58	5.15E-01	NS	0.12	8.44E-01	NS	-0.02	7.01E-01	NS	-0.08	7.96E-01	NS	0.18	5.54E-01	NS	0.10
B7ZKJ8	4.36E-01	NS	0.10	4.63E-01	NS	0.23	3.75E-01	NS	0.23	9.62E-01	NS	0.07	3.92E-01	NS	0.22	8.85E-01	NS	0.17
D3JV41	1.48E-01	NS	-0.40	8.23E-01	NS	0.00	7.22E-01	NS	0.22	7.74E-01	NS	0.23	1.26E-01	NS	-0.46	5.96E-01	NS	0.24
A0A140VK00	9.74E-02	NS	-0.43	7.47E-01	NS	-0.08	6.15E-01	NS	-0.10	4.31E-01	NS	-0.26	3.09E-01	NS	-0.29	7.65E-01	NS	-0.08
F8VV57	1.50E-01	NS	0.44	1.76E-01	NS	2.61	4.23E-01	NS	0.36	7.34E-01	NS	-0.03	8.87E-01	NS	0.15	1.01E-01	NS	0.52
V9HWN8	6.53E-01	NS	-0.28	2.09E-01	NS	-0.73	5.01E-01	NS	0.23	3.93E-01	NS	-0.52	9.95E-01	NS	-0.11	4.64E-01	NS	-0.44
A0A0G2JPA8	6.13E-01	NS	-0.17	5.65E-01	NS	-0.19	8.20E-01	NS	-0.07	8.03E-01	NS	0.03	7.60E-01	NS	0.04	5.67E-01	NS	0.14
A0A182DWH7	7.70E-02	NS	-0.56	2.45E-01	NS	-0.34	3.33E-01	NS	-0.33	2.25E-01	NS	-0.40	4.54E-01	NS	-0.25	5.72E-01	NS	-0.18
S4R3Y4	8.05E-01	NS	0.02	3.48E-02	NS	0.44	2.56E-01	NS	-0.51	8.02E-01	NS	0.03	5.47E-01	NS	0.13	9.44E-01	NS	-0.02
E7EQ64	1.81E-01	NS	-0.64	2.87E-01	NS	-0.53	4.88E-01	NS	-0.26	5.73E-03	NS	-1.26	8.36E-01	NS	0.08	2.52E-01	NS	-0.46
Q59EA3	9.16E-01	NS	-0.68	8.20E-01	NS	0.14	5.67E-01	NS	0.09	9.51E-01	NS	-0.10	3.34E-01	NS	0.47	9.49E-01	NS	-0.45
A0A3B3IS66	1.40E-01	NS	-0.31	2.48E-02	NS	0.43	7.85E-01	NS	0.13	9.57E-01	NS	0.05	7.37E-01	NS	0.09	8.13E-01	NS	-0.04
A0A384MEF1	8.72E-01	NS	0.12	6.21E-01	NS	-0.22	5.62E-01	NS	0.29	6.42E-01	NS	0.17	9.39E-01	NS	-0.04	4.98E-01	NS	0.33
V9GYM3	7.17E-01	NS	-2.01	9.46E-01	NS	0.04	7.57E-01	NS	-1.56	9.56E-01	NS	-1.53	6.29E-01	NS	-2.02	8.93E-01	NS	0.30
Q68CK4	1.80E-01	NS	-0.46	5.18E-02	NS	-0.43	7.74E-01	NS	0.00	1.49E-01	NS	-0.29	2.46E-01	NS	-0.27	3.33E-01	NS	-0.10
Q53F26	2.43E-01	NS	0.75	1.52E-01	NS	0.51	7.11E-01	NS	0.83	8.34E-01	NS	0.46	3.52E-02	NS	0.97	7.39E-01	NS	0.54
V9HWD8	7.50E-01	NS	-0.27	6.17E-01	NS	0.14	2.77E-01	NS	-0.69	6.91E-01	NS	-0.24	9.04E-01	NS	0.14	5.55E-01	NS	-0.39
B2R6W1	6.17E-01	NS	-0.15	2.72E-01	NS	-0.33	3.02E-01	NS	0.24	6.05E-01	NS	0.10	2.09E-01	NS	-0.33	7.21E-01	NS	0.15
B4E1Z4	8.12E-01	NS	0.01	9.91E-01	NS	-0.02	6.07E-01	NS	0.08	7.34E-01	NS	0.03	5.35E-01	NS	0.13	7.66E-01	NS	-0.12
A0A3G1HEF1	3.18E-01	NS	0.51	7.82E-01	NS	-0.06	1.60E-01	NS	0.59	4.67E-01	NS	0.17	7.91E-01	NS	0.01	1.96E-01	NS	0.61
E9KL23	1.07E-01	NS	-1.35	4.38E-02	NS	-1.40	6.29E-01	NS	-0.20	2.84E-01	NS	-0.53	3.51E-01	NS	-0.62	6.27E-01	NS	-0.27
A0A0J9YY65	5.09E-01	NS	-0.19	4.34E-02	NS	-0.53	2.73E-01	NS	0.42	5.10E-01	NS	0.12	8.69E-01	NS	-0.04	2.32E-01	NS	0.41
Q59EZ3	3.38E-01	NS	0.69	3.28E-03	NS	-1.70	3.49E-01	NS	1.59	5.52E-01	NS	0.86	9.52E-01	NS	0.36	1.17E-01	NS	1.28
V9HVY1	1.08E-01	NS	1.59	1.60E-01	NS	0.91	9.76E-02	NS	1.25	3.18E-02	NS	2.02	4.35E-02	NS	1.77	4.35E-01	NS	0.90
B4E1C2	2.29E-01	NS	-0.43	5.73E-01	NS	-0.19	6.50E-01	NS	-0.15	4.78E-01	NS	-0.24	7.98E-01	NS	0.06	9.48E-01	NS	-0.03
D3DNN4	2.00E-01	NS	0.69	7.17E-01	NS	0.15	1.52E-01	NS	0.99	9.27E-01	NS	0.18	2.67E-01	NS	-0.83	7.41E-01	NS	0.35
Q96K68	1.98E-01	NS	-0.78	6.02E-01	NS	-0.28	2.60E-01	NS	0.68	4.03E-01	NS	-0.52	4.71E-01	NS	-0.45	7.82E-01	NS	-0.18
A0A3B3ISJ1	4.91E-01	NS	0.20	5.45E-01	NS	0.21	4.08E-01	NS	0.26	8.43E-02	NS	0.32	2.90E-01	NS	-0.18	6.37E-01	NS	0.10
J3KPA1	5.09E-01	NS	-0.35	7.35E-01	NS	0.16	9.54E-01	NS	0.19	5.35E-01	NS	-0.10	7.69E-01	NS	0.24	5.37E-01	NS	0.27
F8W7L3	5.33E-01	NS	-0.65	7.56E-01	NS	0.34	9.59E-01	NS	-0.33	9.14E-01	NS	-0.11	7.35E-01	NS	-0.57	5.69E-01	NS	-0.52
A8K8Z4	3.76E-01	NS	-0.16	6.76E-01	NS	-0.06	8.09E-01	NS	0.08	7.28E-01	NS	0.08	2.48E-01	NS	-0.17	7.19E-01	NS	0.06
A0A024R6N9	3.15E-01	NS	-0.28	3.66E-02	NS	-0.50	3.17E-01	NS	-0.26	2.22E-01	NS	-0.35	7.94E-01	NS	-0.03	2.87E-01	NS	-0.20
Q9BS19	1.10E-01	NS	-0.31	5.05E-01	NS	-0.14	8.84E-01	NS	0.01	2.77E-01	NS	-0.28	4.19E-01	NS	-0.17	3.21E-01	NS	-0.27
D6RAR4	6.90E-01	NS	-0.27	7.49E-01	NS	0.10	7.27E-01	NS	-0.25	9.05E-01	NS	-0.05	9.61E-01	NS	0.06	8.43E-01	NS	-0.13
A0A2Z4LCH4	9.94E-01	NS	-0.05	5.36E-01	NS	-0.47	7.93E-01	NS	0.09	7.83E-01	NS	-0.32	9.71E-01	NS	-0.19	9.21E-01	NS	-0.14
A0A0A0MRJ7	3.27E-01	NS	-0.32	9.34E-01	NS	-0.01	6.87E-01	NS	-0.11	4.98E-01	NS	-0.18	7.22E-01	NS	0.10	2.85E-01	NS	-0.26
A0A024R035	8.32E-01	NS	-0.17	7.47E-01	NS	-0.16	6.37E-01	NS	0.12	6.71E-01	NS	0.14	9.41E-01	NS	0.04	7.26E-01	NS	0.08
B4DRT3	2.07E-01	NS	0.37	1.72E-01	NS	1.12	4.59E-01	NS	0.43	1.38E-01	NS	0.59	2.34E-01	NS	0.80	4.37E-03	NS	1.82
Q9UEU7	5.33E-01	NS	0.96	6.37E-01	NS	0.61	9.37E-01	NS	-0.02	5.85E-01	NS	0.61	8.53E-01	NS	0.14	8.79E-01	NS	-0.11
Q05CV2	3.50E-01	NS	0.28	4.23E-01	NS	-0.23	4.77E-01	NS	0.39	1.75E-02	NS	0.53	9.21E-01	NS	0.07	1.03E-01	NS	0.55
A0A384N669	4.26E-01	NS	-0.64	6.97E-01	NS	-0.37	6.16E-01	NS	-0.47	6.05E-01	NS	-0.47	7.03E-01	NS	-0.37	6.83E-01	NS	-0.39
F5H1C6	7.38E-01	NS	-0.87	7.09E-01	NS	-0.22	4.39E-01	NS	0.92	9.66E-01	NS	-0.51	8.91E-01	NS	-0.69	5.27E-01	NS	-0.02
Q53GN8	7.97E-01	NS	0.19	4.67E-01	NS	0.18	6.51E-01	NS	-0.21	9.11E-01	NS	0.21	4.25E-01	NS	0.41	5.09E-01	NS	-0.37
A0A2S0BDD1	9.87E-01	NS	-0.01	4.14E-01	NS	-0.38	6.30E-01	NS	0.13	4.26E-01	NS	-0.39	8.19E-01	NS	-0.02	7.09E-01	NS	-0.20
B2RAN2	7.08E-01	NS	0.17	2.13E-01	NS	0.42	6.79E-01	NS	-0.12	4.52E-01	NS	0.26	4.18E-01	NS	0.35	8.87E-01	NS	-0.07
Q6LAN8	8.85E-01	NS	-0.11	7.96E-01	NS	0.12	6.06E-01	NS	-0.72	6.57E-01	NS	-0.73	8.76E-01	NS	0.00	6.50E-01	NS	-0.49
C9JV77	8.91E-01	NS	-0.17	3.54E-01	NS	0.42	4.19E-01	NS	-0.54	9.37E-01	NS	-0.14	6.41E-01	NS	0.22	6.83E-01	NS	-0.28
B2R7F8	1.26E-01	NS	0.31	7.28E-01	NS	-0.07	7.48E-01	NS	0.03	3.88E-01	NS	0.12	9.63E-01	NS	0.01	9.37E-01	NS	0.06
Q5JP69	3.11E-01	NS	0.42	1.80E-01	NS	0.50	6.03E-01	NS	0.25	2.71E-01	NS	0.61	6.93E-01	NS	0.14	4.88E-01	NS	0.17
B0YJC6	6.21E-01	NS	0.17	6.37E-01	NS	0.16	4.06E-01	NS	0.45	8.01E-01	NS	0.04	2.35E-01	NS	0.41	6.97E-01	NS	-0.21
H0YID6	6.40E-01	NS	0.75	3.39E-01	NS	-0.95	3.49E-01	NS	1.95	2.23E-01	NS	-1.21	3.79E-01	NS	0.63	4.21E-01	NS	0.52
B4DPH4	6.51E-01	NS	0.10	9.90E-01	NS	-0.02	5.74E-01	NS	0.09	3.20E-01	NS	0.15	7.32E-01	NS	-0.08	9.92E-01	NS	0.04
A0A087X1J7	7.18E-01	NS	-0.12	2.20E-01	NS	-0.50	6.70E-01	NS	0.23	5.63E-01	NS	0.22	8.12E-01	NS	-0.11	3.16E-01	NS	0.40
B4DVE1	8.94E-01	NS	0.05	9.62E-01	NS	-0.05	6.20E-01	NS	0.84	9.38E-01	NS	-0.04	3.92E-01	NS	0.34	4.94E-01	NS	1.21
Q9H2E1	3.76E-01	NS	-0.91	5.88E-01	NS	0.48	6.15E-01	NS	-0.63	9.75E-01	NS	-0.10	2.65E-01	NS	-1.55	6.11E-01	NS	0.29
A0A384NKS6	1.34E-01	NS	-0.51	7.04E-02	NS	-0.46	5.38E-01	NS	0.20	9.72E-01	NS	-0.01	4.27E-01	NS	-0.23	7.81E-01	NS	-0.08
Q8NBH6	9.97E-01	NS	-0.03	7.26E-01	NS	-0.11	3.42E-01	NS	0.50	6.97E-01	NS	0.27	9.12E-01	NS	-0.07	6.97E-01	NS	0.21
Q9UNL6	6.91E-01	NS	0.59	9.56E-02	NS	-0.57	6.44E-02	NS	-0.26	5.90E-01	NS	0.79	3.49E-01	NS	-0.31	1.64E-01	NS	1.86
A0A024R104	6.36E-01	NS	-0.18	7.15E-01	NS	0.22	3.22E-01	NS	-0.42	7.11E-01	NS	0.23	5.56E-01	NS	-0.18	5.56E-01	NS	-0.18
A0A0F7G8J1	7.15E-01	NS	0.07	4.90E-01	NS	-0.26	1.56E-01	NS	0.47	4.07E-01	NS	0.19	2.90E-01	NS	-0.40	6.99E-01	NS	0.10
A0A0S2Z3E8	8.63E-01	NS	-0.16	5.82E-01	NS	0.53	7.39E-01	NS	-1.08	4.52E-01	NS	-1.28	4.45E-01	NS	-1.41	1.71E-01	NS	-2.39
B0YIW2	9.20E-01	NS	0.30	4.14E-01	NS	0.98	5.07E-01	NS	-0.35	2.77E-01	NS	-0.57	7.26E-01	NS	-0.19	9.86E-01	NS	0.47
A0A1B0GUU9	3.95E-01	NS	0.30	7.70E-01	NS	-0.45	4.71E-01	NS	0.39	6.54E-01	NS	0.00	6.14E-01	NS	0.06	2.76E-01	NS	0.87
B4DZ36	7.90E-01	NS	0.11	5.74E-01	NS	0.27	6.76E-01	NS	0.41	7.05E-01	NS	0.20	9.38E-01	NS	0.05	9.56E-01	NS	-0.04
A0A1K0GXZ1	4.92E-01	NS	0.78	6.59E-02	NS	-1.02	9.61E-02	NS	-0.58	8.54E-01	NS	0.99	9.90E-01	NS	0.31	2.16E-01	NS	1.56
Q5J875	6.62E-01	NS	-0.23	6.69E-01	NS	0.07	9.23E-01	NS	-0.01	9.91E-01	NS	0.01	8.49E-01	NS	-0.08	9.24E-01	NS	-0.04
D9YZU5	5.92E-01	NS	1.20	1.18E-01	NS	-0.88	1.65E-01	NS	-0.46	4.66E-01	NS	1.36	7.98E-01	NS	0.15	1.78E-01	NS	2.46

Uniprot Symbol	16 h/Pre			1 d/Pre			2 d/Pre			3 d/Pre			7 d/Pre			14 d/Pre			21 d/Pre		
	p-value	*FDR*	*log2(FC)*	*p-value*	*FDR*	*log2(FC)*	*p-value*	*FDR*	*log2(FC)*	*p-value*	*FDR*	*log2(FC)*	*p-value*	*FDR*	*log2(FC)*	*p-value*	*FDR*	*log2(FC)*	*p-value*	*FDR*	*log2(FC)*
B4E3P1	1.47E-01	NS	0.82	1.70E-01	NS	1.17	5.16E-01	NS	0.43	9.47E-01	NS	0.08	4.20E-01	NS	0.53	9.94E-01	NS	0.16	9.68E-01	NS	0.00
X6RJP6	7.29E-01	NS	0.41	4.42E-01	NS	0.20	6.76E-02	NS	0.93	8.42E-01	NS	-0.17	2.98E-01	NS	1.28	8.35E-02	NS	0.91	4.31E-01	NS	0.77
I3L3D5	4.53E-01	NS	-0.53	8.83E-01	NS	-0.04	7.47E-01	NS	0.30	1.36E-01	NS	-1.14	5.59E-01	NS	0.52	9.65E-02	NS	-1.28	6.57E-01	NS	-0.39
D0PNI1	4.28E-01	NS	0.45	3.40E-02	NS	1.22	2.56E-01	NS	-0.69	1.08E-01	NS	-0.94	1.76E-01	NS	0.48	5.95E-01	NS	-0.19	8.32E-01	NS	0.01
Q53GK6	5.13E-01	NS	0.33	4.62E-02	NS	0.96	5.06E-01	NS	0.27	1.73E-01	NS	0.58	7.33E-02	NS	0.79	1.32E-01	NS	0.66	5.15E-01	NS	0.46
F2RM35	9.79E-01	NS	0.05	5.77E-01	NS	-0.11	6.59E-01	NS	0.16	4.72E-01	NS	-0.16	2.20E-02	NS	-0.44	6.29E-02	NS	-0.40	2.31E-01	NS	-0.27
B4DRY0	4.40E-02	NS	0.57	3.35E-02	NS	0.88	5.56E-02	NS	0.67	7.54E-01	NS	-0.09	1.82E-01	NS	0.56	8.84E-02	NS	0.62	3.65E-03	NS	2.01
Q53H26	6.22E-02	NS	-0.84	4.95E-01	NS	-0.29	3.06E-01	NS	-0.42	1.76E-01	NS	-0.88	9.68E-01	NS	0.33	4.17E-01	NS	0.02	9.99E-02	NS	-1.03
RT-Cal protein	5.47E-01	NS	0.08	7.06E-02	NS	-0.42	8.31E-01	NS	-0.02	9.53E-01	NS	-0.01	6.36E-01	NS	-0.05	9.39E-01	NS	0.01	9.70E-01	NS	0.01
A0A0S2Z3D5	1.11E-01	NS	-0.86	3.23E-01	NS	-0.28	9.00E-01	NS	0.16	5.38E-01	NS	-0.03	8.52E-01	NS	0.07	3.83E-01	NS	0.32	5.42E-01	NS	-0.15
A0A024RAG6	2.31E-01	NS	-0.66	7.98E-01	NS	-0.01	8.41E-01	NS	-0.04	3.86E-01	NS	-0.30	3.98E-01	NS	0.38	1.27E-01	NS	-0.49	4.13E-01	NS	-0.23
A0A087WW43	5.72E-01	NS	0.32	4.58E-01	NS	0.19	3.18E-01	NS	0.22	1.94E-01	NS	0.20	5.12E-02	NS	0.26	6.81E-01	NS	0.19	2.35E-01	NS	0.31
B1AP58	7.41E-01	NS	0.23	2.51E-01	NS	0.74	1.41E-01	NS	0.52	3.98E-01	NS	0.52	3.13E-02	NS	0.60	6.85E-01	NS	0.35	5.31E-01	NS	0.38
B2R888	6.13E-01	NS	0.22	1.71E-03	NS	0.68	1.88E-02	NS	0.65	3.24E-02	NS	0.48	1.50E-01	NS	0.42	1.09E-01	NS	0.23	2.43E-01	NS	0.26
A0A024R962	7.38E-01	NS	0.00	4.32E-02	NS	-1.57	6.70E-01	NS	-0.17	9.54E-01	NS	-0.09	1.29E-01	NS	-1.06	6.79E-01	NS	0.01	3.78E-01	NS	-0.58
D9ZGG2	8.34E-01	NS	-0.04	2.55E-02	NS	-0.45	6.04E-02	NS	-0.35	1.40E-01	NS	-0.25	3.52E-02	NS	-0.40	4.10E-01	NS	-0.13	3.54E-01	NS	-0.17
B7Z549	9.39E-01	NS	0.03	8.71E-01	NS	0.11	5.45E-01	NS	0.33	1.08E-01	NS	0.66	3.54E-02	NS	0.57	4.95E-01	NS	0.49	5.91E-01	NS	0.43
A0A024RAA7	4.45E-01	NS	-0.20	6.01E-01	NS	0.39	9.06E-01	NS	0.07	6.82E-01	NS	-0.09	6.61E-01	NS	0.26	5.74E-01	NS	0.13	9.97E-01	NS	0.13
D6R934	5.02E-01	NS	-0.20	8.08E-01	NS	0.26	9.21E-01	NS	-0.03	5.46E-01	NS	-0.18	7.82E-01	NS	0.12	6.99E-01	NS	-0.11	5.44E-01	NS	-0.15
Q8IVC0	3.81E-01	NS	-0.31	7.84E-01	NS	-0.07	6.70E-01	NS	-0.10	6.49E-01	NS	0.17	3.84E-01	NS	0.22	8.00E-01	NS	-0.04	9.00E-01	NS	-0.03
A0A140VKF3	8.98E-01	NS	0.05	7.73E-01	NS	0.27	9.96E-01	NS	0.01	2.04E-01	NS	0.27	8.44E-02	NS	0.41	7.81E-01	NS	0.11	5.57E-01	NS	0.28
B3KT06	4.46E-01	NS	1.74	5.78E-01	NS	4.28	1.25E-01	NS	3.11	6.69E-01	NS	-0.54	3.18E-01	NS	3.13	2.22E-01	NS	2.94	5.05E-01	NS	2.53
E9PIT3	8.65E-01	NS	-0.17	3.14E-01	NS	-0.71	8.50E-01	NS	0.13	7.43E-01	NS	0.04	2.95E-01	NS	-0.96	4.86E-01	NS	0.37	9.00E-01	NS	-0.24
V9HWA9	9.83E-01	NS	0.05	4.46E-01	NS	0.30	2.12E-01	NS	0.37	1.45E-01	NS	0.40	2.90E-02	NS	0.73	4.80E-01	NS	0.28	3.19E-01	NS	0.49
D3DPG0	8.30E-01	NS	-0.09	3.91E-01	NS	0.40	5.97E-01	NS	0.11	4.94E-01	NS	0.26	5.76E-01	NS	0.19	8.79E-01	NS	0.14	9.52E-01	NS	0.20
Q5VY30	7.82E-01	NS	0.27	2.69E-01	NS	-0.45	8.07E-01	NS	0.24	5.97E-01	NS	0.34	3.36E-01	NS	-0.34	1.72E-01	NS	0.40	7.83E-01	NS	0.07
V9HW98	3.83E-01	NS	-0.35	9.73E-01	NS	0.07	4.18E-01	NS	-0.30	8.62E-01	NS	0.07	3.86E-01	NS	-0.30	7.02E-01	NS	0.36	2.44E-01	NS	-0.50
V9HWE9	6.71E-01	NS	0.13	3.29E-02	NS	2.04	1.69E-01	NS	0.97	9.85E-01	NS	-0.30	7.38E-02	NS	1.18	6.35E-01	NS	0.12	3.26E-01	NS	0.30
A5PL27	5.26E-01	NS	0.10	8.10E-01	NS	0.09	9.02E-01	NS	0.01	5.72E-01	NS	0.45	6.57E-01	NS	0.36	6.43E-01	NS	0.35	7.62E-01	NS	0.23
G3V2W1	4.30E-01	NS	-0.32	6.76E-01	NS	-0.16	9.45E-01	NS	-0.03	7.22E-01	NS	0.07	6.55E-01	NS	0.08	5.17E-01	NS	-0.25	8.13E-01	NS	0.03
M0R2W8	7.11E-01	NS	0.09	3.23E-01	NS	0.58	5.38E-01	NS	0.36	5.22E-01	NS	-0.30	4.88E-01	NS	0.64	3.37E-01	NS	-0.51	4.16E-01	NS	-0.42
B2R815	5.16E-01	NS	-0.21	7.72E-01	NS	0.20	8.54E-01	NS	0.00	8.69E-01	NS	0.09	3.47E-01	NS	0.42	6.42E-01	NS	-0.15	9.05E-01	NS	0.19
A0A0G2JPR0	7.66E-01	NS	0.10	6.88E-01	NS	0.20	3.43E-01	NS	0.40	3.16E-02	NS	0.65	1.11E-01	NS	0.58	1.11E-01	NS	0.56	6.74E-01	NS	0.12
B4DDT3	4.73E-01	NS	-0.23	5.52E-01	NS	0.47	2.40E-01	NS	-0.37	3.36E-01	NS	-0.35	9.42E-01	NS	-0.01	9.00E-01	NS	0.03	7.70E-01	NS	-0.09
B7Z555	9.55E-01	NS	0.01	5.83E-01	NS	0.31	3.57E-01	NS	0.13	7.61E-01	NS	0.14	2.17E-02	NS	0.35	9.39E-01	NS	0.14	5.66E-01	NS	0.32
B4DPQ0	4.24E-01	NS	0.19	4.80E-01	NS	0.26	9.83E-01	NS	-0.01	8.09E-01	NS	0.04	3.72E-01	NS	0.25	7.10E-01	NS	0.06	8.63E-01	NS	0.03
Q59GS8	9.98E-01	NS	0.02	5.42E-01	NS	0.18	4.59E-01	NS	0.42	1.19E-01	NS	0.49	1.79E-01	NS	0.45	4.10E-01	NS	0.39	5.22E-01	NS	0.27
F8W876	5.95E-01	NS	0.17	8.68E-01	NS	0.01	6.83E-01	NS	-0.15	9.67E-01	NS	-0.05	7.12E-01	NS	0.05	5.63E-01	NS	0.13	9.65E-01	NS	0.20
F5GXS0	7.15E-01	NS	0.16	5.07E-01	NS	0.25	2.71E-01	NS	0.50	9.81E-02	NS	0.62	4.43E-02	NS	0.70	1.87E-01	NS	0.58	5.08E-01	NS	0.37
A2RTY6	9.75E-01	NS	0.00	8.90E-01	NS	0.10	4.12E-01	NS	0.25	4.27E-01	NS	0.32	4.50E-02	NS	0.56	6.01E-01	NS	0.29	6.82E-01	NS	0.34
A0A024R3E3	6.14E-02	NS	-2.97	6.02E-01	NS	-1.14	6.33E-01	NS	-0.35	1.41E-01	NS	-2.12	9.67E-01	NS	0.66	2.58E-01	NS	-1.80	4.41E-02	NS	-3.21
B2R4M6	1.09E-01	NS	1.34	1.54E-01	NS	2.32	1.31E-01	NS	1.33	8.42E-01	NS	0.21	2.61E-01	NS	0.34	7.66E-01	NS	0.30	3.86E-01	NS	0.80
B3KNX0	4.16E-01	NS	-0.35	7.46E-01	NS	-0.10	9.89E-01	NS	-0.02	8.16E-01	NS	0.07	8.19E-01	NS	0.06	9.56E-01	NS	0.04	4.34E-01	NS	0.36
L8E853	8.07E-01	NS	-0.07	1.13E-01	NS	-0.75	8.40E-01	NS	0.05	6.17E-01	NS	0.20	7.32E-01	NS	-0.05	2.88E-01	NS	0.53	4.73E-01	NS	0.39
Q59E93	2.32E-01	NS	0.46	1.12E-01	NS	1.42	7.78E-02	NS	0.69	1.07E-01	NS	0.48	2.09E-01	NS	0.70	4.16E-01	NS	0.42	2.15E-01	NS	0.45
B3KS79	7.25E-01	NS	0.19	4.41E-01	NS	0.35	8.13E-01	NS	0.20	7.45E-01	NS	0.10	7.24E-01	NS	0.07	4.90E-01	NS	-0.25	8.63E-01	NS	-0.09
A0A384MDV8	8.42E-01	NS	0.12	6.37E-01	NS	0.14	2.95E-01	NS	0.40	6.68E-01	NS	-0.09	1.71E-01	NS	0.37	6.04E-01	NS	-0.10	5.93E-01	NS	-0.15
B2RA39	6.15E-01	NS	-0.15	2.54E-02	NS	2.33	5.74E-01	NS	1.56	1.65E-01	NS	-0.57	2.93E-01	NS	1.08	1.12E-01	NS	-0.65	3.06E-01	NS	2.08
B2CIS9	2.07E-01	NS	-1.01	6.96E-01	NS	0.87	9.50E-01	NS	-0.15	4.52E-01	NS	0.24	5.42E-01	NS	-0.35	9.82E-01	NS	-0.20	7.45E-01	NS	0.38
H0YAC1	3.82E-01	NS	0.41	9.44E-01	NS	0.00	9.35E-01	NS	0.08	8.77E-01	NS	-0.04	8.24E-01	NS	-0.05	9.92E-01	NS	0.01	9.90E-01	NS	-0.01
Q8TAY0	6.64E-01	NS	-0.26	9.39E-01	NS	0.05	9.49E-01	NS	0.05	5.89E-01	NS	0.20	3.01E-01	NS	0.37	7.00E-01	NS	0.18	7.49E-01	NS	0.31
H6VRG1	8.27E-01	NS	0.12	5.93E-01	NS	0.24	5.47E-01	NS	0.22	2.31E-01	NS	-0.67	9.99E-01	NS	-0.07	6.95E-01	NS	-0.24	1.70E-01	NS	1.18
B7ZLE5	1.11E-02	NS	0.59	6.71E-02	NS	0.78	1.23E-01	NS	0.46	8.39E-03	NS	0.91	1.62E-01	NS	1.01	9.50E-02	NS	0.50	4.21E-02	NS	1.44
A6XND1	5.45E-01	NS	-0.16	2.16E-01	NS	-0.35	3.55E-01	NS	-0.23	8.09E-02	NS	-0.50	7.35E-02	NS	-0.66	4.72E-01	NS	-0.19	8.78E-02	NS	-0.50
F8W0J6	4.50E-01	NS	1.17	5.12E-02	NS	1.23	6.58E-01	NS	0.20	2.03E-01	NS	0.79	2.29E-01	NS	0.48	1.15E-01	NS	0.86	9.22E-01	NS	0.02
A0A140VJI7	7.95E-01	NS	-0.07	3.92E-01	NS	-0.38	3.25E-01	NS	-0.46	2.48E-01	NS	-0.48	1.26E-01	NS	-0.71	8.27E-01	NS	-0.09	3.99E-01	NS	-0.37
A0A087WSY5	8.17E-01	NS	-0.05	5.23E-01	NS	-0.16	2.29E-01	NS	-0.43	8.37E-01	NS	0.03	5.38E-01	NS	0.16	4.09E-01	NS	-0.30	8.90E-01	NS	0.03
A0N071	9.28E-02	NS	2.87	5.48E-02	NS	2.86	4.75E-02	NS	1.93	9.18E-02	NS	1.63	6.36E-01	NS	0.27	4.68E-01	NS	0.44	1.16E-01	NS	0.72
Q53H37	3.87E-01	NS	-1.21	7.36E-01	NS	0.51	5.22E-01	NS	-1.08	2.54E-01	NS	-1.69	6.17E-01	NS	-0.68	9.54E-03	NS	-4.43	6.75E-01	NS	0.40
J3QR68	5.36E-01	NS	-0.63	9.21E-01	NS	0.46	5.77E-01	NS	-0.59	6.04E-01	NS	-0.36	6.25E-01	NS	-0.75	3.19E-01	NS	-1.12	4.26E-01	NS	-0.21
A8K1K1	7.51E-01	NS	-0.24	9.46E-01	NS	0.14	8.80E-01	NS	-0.12	8.94E-01	NS	0.27	8.58E-01	NS	0.25	5.70E-01	NS	0.46	9.90E-01	NS	0.57
A0A024R9Q1	4.16E-01	NS	-0.48	8.64E-01	NS	0.08	8.97E-01	NS	-0.03	9.55E-01	NS	0.04	6.64E-01	NS	-0.31	9.34E-01	NS	-0.06	6.02E-01	NS	0.34
E7END6	5.60E-01	NS	-0.15	4.14E-01	NS	-0.19	8.07E-01	NS	-0.05	5.58E-01	NS	-0.14	1.18E-01	NS	-0.39	5.24E-01	NS	0.18	2.54E-01	NS	-0.32
B2R950	6.78E-01	NS	-0.11	5.82E-01	NS	0.44	9.54E-01	NS	0.01	8.19E-01	NS	0.25	4.29E-01	NS	0.58	9.59E-01	NS	0.16	7.72E-01	NS	0.24
A0A024R1X6	6.95E-01	NS	0.11	6.42E-01	NS	0.12	2.52E-01	NS	0.91	7.27E-01	NS	0.27	5.23E-01	NS	0.34	1.37E-01	NS	0.80	6.21E-02	NS	1.49
I3L145	7.70E-01	NS	0.03	8.89E-01	NS	0.11	7.66E-01	NS	0.09	6.30E-01	NS	0.20	2.10E-01	NS	0.39	6.03E-01	NS	-0.08	3.11E-01	NS	0.56
Q4LE79	6.22E-01	NS	-0.16	4.85E-01	NS	-0.18	1.72E-01	NS	0.48	9.00E-01	NS	0.36	8.05E-01	NS	-0.10	7.68E-01	NS	-0.04	3.07E-01	NS	0.22
Q499G5	8.85E-01	NS	0.09	3.75E-01	NS	-0.33	6.69E-01	NS	0.24	5.40E-03	NS	0.64	5.63E-01	NS	-0.01	4.16E-01	NS	0.48	6.78E-01	NS	0.27
S4R471	3.24E-01	NS	0.37	2.28E-01	NS	0.55	2.51E-01	NS	0.51	6.50E-01	NS	0.14	2.98E-01	NS	0.30	4.31E-01	NS	0.19	9.42E-01	NS	-0.02
V9HWF6	9.81E-01	NS	-0.39	9.19E-01	NS	0.50	8.94E-01	NS	1.21	7.08E-01	NS	0.86	7.80E-01	NS	-0.61	3.27E-01	NS	-0.15	3.88E-01	NS	0.35
B7Z8Q5	6.84E-01	NS	0.15	4.56E-01	NS	0.46	8.52E-01	NS	0.14	9.26E-01	NS	0.22	4.36E-01	NS	0.30	9.41E-01	NS	-0.05	9.24E-01	NS	0.09
P78492	3.63E-01	NS	0.22	3.92E-01	NS	0.21	4.57E-01	NS	0.21	1.04E-01	NS	0.47	6.75E-01	NS	0.08	1.73E-01	NS	0.45	7.85E-02	NS	0.45
V9HWP0	9.31E-01	NS	-0.06	5.43E-01	NS	0.60	5.04E-01	NS	0.41	9.42E-01	NS	-0.10	3.84E-01	NS	0.32	6.23E-01	NS	-0.30	2.87E-01	NS	-0.59
V9HW68	1.52E-01	NS	-1.19	8.64E-01	NS	0.19	4.96E-01	NS	-0.42	1.02E-01	NS	-1.37	5.71E-01	NS	0.37	7.19E-02	NS	-1.77	5.43E-02	NS	-1.75
V9HWE3	1.24E-01	NS	0.69	7.08E-01	NS	0.58	9.37E-01	NS	0.10	8.52E-01	NS	0.04	4.16E-01	NS	-0.50	4.09E-01	NS	-0.53	9.88E-01	NS	0.07
C9JEU5	1.38E-01	NS	1.12	4.04E-03	NS	1.97	1.12E-03	NS	2.25	6.90E-02	NS	2.09	1.73E-01	NS	0.96	1.17E-01	NS	1.63	1.12E-01	NS	1.48
K7ERE3	7.73E-01	NS	-0.47	4.94E-01	NS	-0.10	2.73E-01	NS	0.39	8.33E-01	NS	-0.83	4.44E-01	NS	0.30	6.60E-01	NS	0.20	3.20E-01	NS	1.28
A0A0U1RR20	9.92E-01	NS	0.11	9.68E-01	NS	-0.01	9.42E-01	NS	0.10	2.01E-01	NS	-0.50	4.19E-01	NS	0.19	5.07E-02	NS	-1.67	2.82E-01	NS	-0.69
E3UN46	6.53E-01	NS	0.10	4.34E-02	NS	0.83	3.07E-01	NS	0.44	8.88E-01	NS	-0.09	3.49E-01	NS	0.23	5.15E-01	NS	-0.23	7.91E-01	NS	0.31
Q5JVE7	3.98E-01	NS	-0.07	2.14E-01	NS	-0.37	7.02E-02	NS	-0.34	2.13E-01	NS	-0.18	3.35E-02	NS	-0.44	6.39E-02	NS	-0.21	4.02E-03	NS	-0.39
J3K000	6.79E-01	NS	0.03	5.19E-01	NS	0.69	6.95E-01	NS	0.28	2.86E-01	NS	0.52	3.40E-01	NS	0.40	5.09E-01	NS	-0.20	3.76E-01	NS	0.53
Q59EP2	4.36E-01	NS	0.31	4.11E-02	NS	0.76	1.44E-01	NS	0.58	2.98E-02	NS	0.61	1.83E-01	NS	0.44	9.27E-01	NS	0.02	8.90E-01	NS	-0.05
K7EQQ3	6.65E-01	NS	-0.20	6.50E-01	NS	-0.22	8.14E-01	NS	0.03	2.96E-01	NS	-0.50	4.72E-01	NS	-0.34	7.93E-01	NS	-0.13	2.84E-01	NS	1.02
H3BRJ9	6.69E-01	NS	-0.39	3.88E-01	NS	-0.44	3.45E-01	NS	-0.64	4.69E-01	NS	-0.40	9.05E-01	NS	0.01	4.23E-01	NS	-0.59	5.88E-01	NS	-0.19
Q5SQ08	8.92E-01	NS	0.03	5.57E-01	NS	-0.09	2.69E-01	NS	0.20	2.52E-01	NS	0.37	6.74E-01	NS	0.13	7.22E-01	NS	0.32	8.21E-01	NS	0.20
Q6MZL2	7.26E-01	NS	0.09	5.56E-02	NS	0.32	7.11E-01	NS	-0.04	5.78E-01	NS	-0.09	3.78E-01	NS	-0.24	4.93E-01	NS	0.18	3.87E-01	NS	-0.26
Q13784	8.47E-01	NS	0.22	8.12E-01	NS	0.14	8.93E-01	NS	-0.05	9.64E-01	NS	0.05	9.16E-01	NS	-0.09	7.72E-01	NS	-0.13	2.95E-01	NS	-0.67
C0JYY2	5.42E-01	NS	-0.17	9.42E-01	NS	-0.02	3.01E-01	NS	-0.31	3.47E-01	NS	-0.29	1.73E-01	NS	0.29	7.17E-01	NS	-0.11	8.32E-01	NS	-0.06
A0A0S2Z4I5	9.01E-01	NS	-0.02	9.65E-01	NS	0.27	9.71E-01	NS	-0.04	5.27E-01	NS	-0.28	6.86E-01	NS	-0.22	1.57E-01	NS	-0.63	1.79E-01	NS	-0.66
D9IWP9	8.14E-01	NS	0.21	9.17E-01	NS	-0.05	4.22E-01	NS	0.18	8.47E-01	NS	-0.10	2.81E-01	NS	-0.40	5.72E-01	NS	-0.20	4.19E-01	NS	-0.32
B2R773	6.76E-01	NS	0.17	7.19E-01	NS	0.67	8.63E-01	NS	-0.02	8.41E-01	NS	0.31	7.63E-01	NS	0.28	6.81E-01	NS	-0.39	9.70E-01	NS	0.17
E9PHK0	9.28E-01	NS	0.03	7.20E-01	NS	0.17	9.89E-01	NS	0.06	3.27E-01	NS	-0.34	5.41E-01	NS	-0.19	5.41E-01	NS	-0.17	4.77E-01	NS	-0.22
B7ZKJ8	9.83E-01	NS	0.08	2.55E-01	NS	-0.19	8.44E-01	NS	0.07	3.11E-01	NS	0.23	6.61E-01	NS	-0.04	4.59E-01	NS	0.22	9.64E-01	NS	0.00
D3JV41	1.94E-01	NS	-0.35	1.57E-02	NS	-0.68	5.91E-01	NS	0.34	8.27E-02	NS	-0.44	7.65E-01	NS	0.16	2.51E-01	NS	-0.32	9.05E-01	NS	-0.01
A0A140VK00	5.50E-01	NS	-0.16	5.48E-01	NS	-0.16	5.24E-01	NS	-0.18	1.03E-01	NS	-0.56	1.26E-01	NS	-0.52	4.20E-01	NS	-0.18	3.49E-01	NS	-0.32
F8VV57	3.49E-01	NS	0.38	3.27E-01	NS	0.29	8.59E-01	NS	0.19	4.40E-01	NS	-0.18	5.08E-01	NS	-0.19	3.24E-02	NS	-1.05	9.32E-02	NS	0.87
V9HWN8	5.86E-01	NS	-0.38	4.00E-01	NS	-0.50	3.79E-01	NS	-0.49	7.16E-01	NS	-0.30	9.51E-01	NS	-0.16	7.36E-01	NS	-0.27	9.16E-01	NS	0.00
A0A0G2JPA8	7.33E-01	NS	0.10	5.69E-01	NS	0.17	7.43E-01	NS	0.13	4.48E-01	NS	0.19	4.40E-01	NS	0.23	9.59E-01	NS	-0.03	7.45E-01	NS	0.18
A0A182DWH7	2.70E-01	NS	-0.40	2.87E-01	NS	-0.32	2.99E-01	NS	-0.36	2.37E-02	NS	-0.89	9.59E-02	NS	-0.71	2.25E-01	NS	-0.46	8.72E-02	NS	-0.70
S4R3Y4	4.71E-01	NS	0.30	8.43E-01	NS	-0.02	4.18E-01	NS	0.28	9.00E-01	NS	0.02	1.20E-01	NS	-1.02	8.24E-01	NS	-0.04	5.83E-01	NS	-0.13
E7EQ64	2.91E-01	NS	-0.50	1.87E-01	NS	-0.62	3.82E-02	NS	-1.15	7.47E-02	NS	-0.88	3.27E-02	NS	-1.11	4.59E-02	NS	-0.99	2.56E-02	NS	-1.21
Q59EA3	7.60E-01	NS	-0.50	9.14E-01	NS	-0.33	8.08E-01	NS	0.00	6.63E-01	NS	-0.65	6.22E-01	NS	-0.10	9.09E-01	NS	-0.13	9.03E-01	NS	-0.18
A0A3B3IS66	8.06E-01	NS	-0.04	8.33E-01	NS	-0.03	5.83E-01	NS	0.23	7.22E-01	NS	-0.05	1.59E-01	NS	-0.32	8.64E-01	NS	0.01	3.94E-01	NS	-0.21
A0A384MEF1	8.03E-01	NS	-0.15	9.67E-01	NS	0.08	6.94E-01	NS	0.14	6.45E-01	NS	0.27	3.01E-01	NS	0.39	8.23E-01	NS	0.19	6.52E-01	NS	0.42
V9GYM3	6.60E-01	NS	-2.09	9.90E-01	NS	-1.24	7.93E-01	NS	0.01	5.41E-01	NS	-2.36	5.90E-01	NS	0.87	2.22E-01	NS	-3.48	9.83E-01	NS	-1.55
Q68CK4	4.80E-01	NS	-0.08	9.10E-01	NS	0.00	2.12E-01	NS	-0.19	7.84E-03	NS	-0.65	2.44E-02	NS	-0.66	7.63E-02	NS	-0.40	7.45E-02	NS	-0.52
Q53F26	4.96E-01	NS	1.24	9.33E-02	NS	0.71	8.65E-01	NS	0.41	1.13E-01	NS	1.04	7.65E-01	NS	0.07	2.26E-02	NS	1.30	7.57E-01	NS	0.41
V9HWD8	9.37E-01	NS	0.02	5.90E-01	NS	-0.30	7.09E-01	NS	-0.24	7.03E-01	NS	-0.24	4.01E-01	NS	-0.52	8.05E-01	NS	-0.14	5.02E-01	NS	-0.46
B2R6W1	9.47E-01	NS	0.01	9.18E-01	NS	-0.01	7.86E-01	NS	-0.09	7.48E-01	NS	-0.10	8.74E-01	NS	0.07	2.88E-01	NS	-0.30	7.02E-01	NS	-0.11
B4E1Z4	8.70E-01	NS	-0.01	5.48E-01	NS	0.15	5.90E-01	NS	-0.18	9.67E-01	NS	-0.05	9.46E-01	NS	-0.02	6.97E-01	NS	0.09	6.53E-01	NS	0.10
A0A3G1HEF1	4.79E-01	NS	0.13	9.08E-01	NS	-0.10	6.02E-01	NS	0.10	8.35E-01	NS	0.02	3.86E-01	NS	0.23	7.74E-01	NS	-0.09	4.07E-01	NS	0.33
E9KL23	4.30E-01	NS	-0.53	8.14E-01	NS	0.17	5.41E-01	NS	-0.39	8.05E-01	NS	0.50	9.00E-01	NS	-0.09	2.70E-01	NS	-0.43	6.39E-02	NS	-1.41
A0A0J9YY65	9.96E-01	NS	0.03	2.00E-01	NS	0.47	7.92E-01	NS	-0.05	4.98E-01	NS	-0.19	3.16E-01	NS	0.23	1.96E-01	NS	-0.32	6.08E-01	NS	0.31
Q59EZ3	8.61E-01	NS	0.32	1.61E-01	NS	1.14	3.26E-01	NS	0.69	1.08E-01	NS	-0.69	5.39E-01	NS	0.26	3.96E-02	NS	-1.45	1.42E-01	NS	-0.86
V9HVY1	9.10E-02	NS	1.11	3.64E-01	NS	1.09	5.27E-01	NS	0.61	3.87E-01	NS	1.25	4.96E-02	NS	1.25	5.32E-02	NS	1.95	9.63E-01	NS	0.75
B4E1C2	4.99E-01	NS	-0.21	9.90E-01	NS	0.04	4.30E-01	NS	-0.28	9.23E-02	NS	-0.62	2.44E-01	NS	-0.45	4.40E-01	NS	-0.26	1.60E-01	NS	-0.46
D3DNN4	8.13E-01	NS	0.26	9.98E-01	NS	-0.03	7.01E-01	NS	-0.20	8.77E-01	NS	0.66	9.66E-01	NS	-0.17	8.12E-01	NS	0.06	6.33E-01	NS	0.60
Q96K68	8.34E-01	NS	-0.15	4.94E-01	NS	1.67	3.29E-01	NS	-0.60	2.95E-01	NS	-0.66	7.31E-01	NS	-0.09	1.60E-01	NS	-0.88	1.52E-01	NS	-0.99
A0A3B3ISJ1	7.33E-01	NS	-0.05	5.59E-01	NS	-0.11	6.88E-01	NS	0.05	2.45E-01	NS	0.16	3.75E-01	NS	0.14	5.35E-01	NS	0.09	4.00E-01	NS	0.13
J3KPA1	7.61E-01	NS	0.08	9.56E-01	NS	0.09	7.51E-01	NS	-0.18	3.18E-01	NS	-0.51	4.19E-01	NS	-0.42	3.99E-01	NS	-0.40	4.12E-01	NS	-0.40
F8W7L3	5.41E-01	NS	-0.76	2.07E-01	NS	-1.20	9.55E-01	NS	0.03	7.00E-01	NS	-0.59	9.85E-01	NS	0.11	2.97E-01	NS	-1.06	8.00E-01	NS	-0.43
A8K8Z4	7.69E-01	NS	-0.04	7.17E-01	NS	-0.05	7.29E-01	NS	0.06	3.79E-01	NS	-0.17	3.47E-01	NS	-0.13	4.23E-01	NS	-0.14	4.74E-01	NS	-0.13
A0A024R6N9	3.46E-01	NS	-0.25	2.92E-01	NS	-0.29	3.12E-01	NS	-0.24	1.62E-01	NS	-0.47	5.35E-01	NS	-0.14	1.18E-01	NS	-0.46	2.23E-01	NS	-0.26
Q9BS19	9.69E-01	NS	0.04	2.94E-01	NS	-0.27	7.40E-02	NS	-0.37	1.90E-01	NS	-0.38	6.91E-01	NS	-0.07	2.88E-01	NS	-0.29	5.52E-01	NS	-0.13
D6RAR4	8.45E-01	NS	-0.13	4.87E-01	NS	-0.41	8.61E-01	NS	-0.09	5.54E-01	NS	-0.38	4.99E-01	NS	-0.42	9.34E-01	NS	0.00	5.48E-01	NS	-0.40
A0A2Z4LCH4	8.84E-01	NS	-0.23	4.85E-01	NS	-0.55	4.79E-01	NS	-0.51	8.65E-01	NS	-0.17	9.36E-01	NS	-0.09	9.53E-01	NS	-0.18	8.78E-01	NS	-0.04
A0A0A0MRJ7	9.38E-01	NS	0.04	5.96E-01	NS	-0.14	7.89E-01	NS	-0.05	1.27E-01	NS	-0.44	2.02E-01	NS	-0.41	3.63E-01	NS	-0.29	2.60E-01	NS	-0.38
A0A024R035	9.02E-01	NS	-0.01	8.01E-01	NS	0.01	7.03E-01	NS	0.12	8.26E-01	NS	0.00	7.02E-01	NS	0.14	9.25E-01	NS	-0.08	9.91E-01	NS	-0.07
B4DRT3	1.85E-02	NS	1.47	3.71E-02	NS	1.60	1.04E-01	NS	1.43	3.97E-01	NS	0.24	2.99E-01	NS	0.27	3.99E-01	NS	0.26	5.70E-02	NS	0.83
Q9UEU7	8.51E-01	NS	0.41	5.46E-01	NS	-0.23	9.32E-01	NS	0.70	1.56E-01	NS	0.62	8.26E-01	NS	0.18	1.93E-01	NS	0.63	5.41E-01	NS	0.48
Q05CV2	9.13E-01	NS	-0.04	6.48E-01	NS	0.27	9.71E-01	NS	-0.02	6.76E-01	NS	0.18	2.59E-01	NS	0.29	3.59E-01	NS	-0.27	5.72E-01	NS	0.28
A0A384N669	7.86E-01	NS	-0.22	9.66E-01	NS	-0.18	8.10E-01	NS	-0.17	4.04E-01	NS	-0.67	5.16E-01	NS	-0.52	7.47E-01	NS	-0.31	6.02E-01	NS	-0.48
F5H1C6	9.79E-01	NS	-0.34	2.39E-01	NS	0.65	7.15E-01	NS	-0.80	6.83E-01	NS	-0.12	5.92E-01	NS	0.07	7.09E-01	NS	-0.07	9.32E-01	NS	-0.55
Q53GN8	9.79E-01	NS	0.02	4.90E-01	NS	-0.34	9.22E-01	NS	-0.01	7.85E-01	NS	0.28	7.02E-01	NS	-0.24	4.47E-01	NS	0.29	8.81E-01	NS	-0.12
A0A2S0BDD1	4.58E-01	NS	0.30	6.84E-01	NS	-0.19	5.58E-01	NS	-0.30	9.79E-01	NS	-0.05	7.16E-01	NS	-0.21	6.92E-01	NS	0.13	9.21E-01	NS	0.11
B2RAN2	3.80E-01	NS	0.16	4.28E-01	NS	-0.30	9.69E-01	NS	0.02	5.80E-01	NS	-0.18	4.27E-01	NS	-0.26	4.37E-01	NS	-0.25	8.02E-01	NS	-0.08
Q6LAN8	9.04E-01	NS	-0.16	8.29E-01	NS	-0.51	7.61E-01	NS	-0.20	7.08E-01	NS	-0.61	5.14E-01	NS	-0.75	9.43E-01	NS	-0.36	6.79E-01	NS	-0.84
C9JV77	9.89E-01	NS	-0.04	7.14E-01	NS	-0.22	5.92E-01	NS	-0.37	9.43E-01	NS	0.05	3.71E-01	NS	-0.55	5.26E-01	NS	0.28	7.98E-01	NS	-0.16
B2R7F8	9.11E-01	NS	0.00	8.46E-01	NS	0.10	9.28E-01	NS	0.00	8.26E-01	NS	-0.04	8.15E-01	NS	-0.05	9.76E-01	NS	0.01	5.19E-01	NS	-0.14
Q5JP69	2.74E-01	NS	0.40	5.38E-01	NS	-0.27	1.64E-01	NS	0.48	1.61E-01	NS	0.54	2.97E-01	NS	0.40	3.75E-01	NS	0.39	2.26E-01	NS	0.42
B0YJC6	6.52E-01	NS	0.14	8.21E-01	NS	-0.05	6.39E-01	NS	-0.31	4.76E-01	NS	0.33	8.38E-01	NS	-0.09	4.92E-01	NS	0.18	5.67E-01	NS	0.18
H0YID6	8.71E-01	NS	-0.18	9.92E-01	NS	0.12	3.97E-01	NS	-0.51	8.38E-02	NS	-1.96	8.03E-01	NS	-0.49	9.51E-01	NS	0.10	2.57E-01	NS	1.16
B4DPH4	8.05E-01	NS	-0.06	5.25E-01	NS	-0.16	7.60E-01	NS	-0.06	8.10E-01	NS	-0.06	5.12E-01	NS	-0.15	9.77E-01	NS	0.02	2.48E-01	NS	-0.31
A0A087X1J7	6.60E-01	NS	-0.16	5.30E-01	NS	0.31	8.95E-01	NS	-0.03	9.11E-01	NS	0.02	4.42E-01	NS	0.27	9.62E-01	NS	-0.05	8.73E-01	NS	0.03
B4DVE1	9.90E-01	NS	-0.09	8.65E-01	NS	0.76	6.54E-01	NS	-0.23	6.03E-01	NS	-0.33	9.17E-01	NS	-0.11	5.47E-01	NS	0.28	9.90E-01	NS	-0.05
Q9H2E1	7.32E-01	NS	-0.06	9.37E-01	NS	-0.12	8.75E-01	NS	-0.28	5.27E-01	NS	0.41	3.57E-01	NS	1.74	9.10E-01	NS	0.36	6.89E-01	NS	-0.70
A0A384NKS6	2.61E-01	NS	-0.36	8.15E-01	NS	-0.04	4.19E-01	NS	-0.21	4.51E-01	NS	-0.23	6.04E-01	NS	0.15	3.80E-01	NS	-0.23	3.26E-01	NS	-0.29
Q8NBH6	6.88E-01	NS	-0.13	7.30E-01	NS	0.05	7.22E-01	NS	-0.11	9.92E-01	NS	0.07	3.93E-01	NS	0.36	3.99E-01	NS	0.46	8.02E-01	NS	0.15
Q9UNL6	9.87E-01	NS	0.52	4.04E-01	NS	1.22	2.29E-01	NS	0.47	3.63E-01	NS	0.21	9.99E-01	NS	0.06	2.40E-01	NS	-0.38	7.88E-02	NS	-0.82
A0A024R104	9.91E-01	NS	0.06	7.13E-01	NS	-0.17	2.23E-01	NS	-0.56	6.77E-01	NS	-0.20	6.35E-01	NS	-0.19	9.32E-01	NS	0.03	7.66E-01	NS	-0.08
A0A0F7G8J1	9.76E-01	NS	-0.04	4.24E-01	NS	-0.30	7.79E-01	NS	0.12	7.07E-01	NS	0.13	6.73E-01	NS	0.10	9.09E-01	NS	0.02	7.49E-01	NS	-0.11
A0A0S2Z3E8	4.58E-01	NS	-1.33	8.31E-01	NS	-0.32	8.98E-01	NS	-0.14	7.33E-01	NS	0.45	8.54E-01	NS	0.32	8.76E-01	NS	-0.31	9.95E-01	NS	0.76
B0YIW2	3.96E-01	NS	-0.22	8.55E-02	NS	-1.26	6.94E-01	NS	0.34	7.13E-01	NS	0.10	5.25E-01	NS	0.54	9.10E-01	NS	0.27	7.71E-01	NS	0.03
A0A1B0GUU9	6.71E-01	NS	0.25	1.53E-01	NS	1.01	6.65E-01	NS	0.18	9.49E-01	NS	-0.12	1.50E-01	NS	0.99	4.95E-01	NS	-0.68	9.83E-01	NS	-0.37
B4DZ36	7.62E-01	NS	0.12	6.87E-01	NS	0.27	9.64E-01	NS	-0.12	7.76E-01	NS	0.14	7.15E-01	NS	0.08	8.52E-01	NS	0.18	4.79E-01	NS	0.47
A0A1K0GXZ1	3.12E-01	NS	1.14	2.80E-01	NS	2.14	9.74E-01	NS	1.23	8.36E-01	NS	0.20	2.38E-02	NS	-0.72	6.62E-02	NS	-0.99	1.81E-02	NS	-0.97
Q5J875	8.33E-01	NS	0.08	6.90E-01	NS	-0.16	9.54E-01	NS	-0.02	2.11E-01	NS	-0.57	5.57E-01	NS	-0.27	8.65E-01	NS	0.05	5.88E-01	NS	0.23
D9YZU5	4.66E-01	NS	1.20	2.02E-01	NS	2.53	6.36E-01	NS	1.20	6.21E-01	NS	0.23	3.37E-03	NS	-0.79	4.24E-02	NS	-1.11	3.37E-02	NS	-0.74

Supplementary Table 5.

	0.25 h/Pre			0.5 h/Pre			1 h/Pre			2 h/Pre			4 h/Pre			8 h/Pre			16 h/Pre			1 d/Pre			2 d/Pre			3 d/Pre			7 d/Pre			14 d/Pre			21 d/Pre		
**Uniprot Symbol**	*p-value*	*FDR*	*log2(FC)*	*p-value*	*FDR*	*log2(FC)*	*p-value*	*FDR*	*log2(FC)*	*p-value*	*FDR*	*log2(FC)*	*p-value*	*FDR*	*log2(FC)*	*p-value*	*FDR*	*log2(FC)*	*p-value*	*FDR*	*log2(FC)*	*p-value*	*FDR*	*log2(FC)*	*p-value*	*FDR*	*log2(FC)*	*p-value*	*FDR*	*log2(FC)*	*p-value*	*FDR*	*log2(FC)*	*p-value*	*FDR*	*log2(FC)*	*p-value*	*FDR*	*log2(FC)*
F7H312	7.87E-01	NS	0.38	6.33E-02	NS	3.43	8.98E-01	NS	0.24	9.22E-01	NS	0.32	1.24E-01	NS	0.99	8.71E-01	NS	0.50	5.12E-01	NS	-0.08	4.43E-01	NS	-0.21	5.98E-01	NS	0.07	9.08E-01	NS	0.07	5.27E-01	NS	-0.07	8.43E-02	NS	0.45	2.03E-01	NS	1.28
F6SYK0	1.02E-02	NS	1.57	2.47E-02	NS	1.29	9.35E-02	NS	2.26	7.27E-03	NS	1.69	6.80E-03	NS	2.15	8.36E-03	NS	2.19	2.76E-02	NS	1.15	1.25E-02	NS	1.55	9.88E-02	NS	1.65	7.03E-02	NS	1.41	3.22E-01	NS	1.32	1.02E-01	NS	1.40	6.65E-02	NS	0.83
F6RKI4	5.06E-01	NS	1.83	4.38E-02	NS	1.99	1.11E-02	NS	2.09	9.69E-01	NS	0.53	1.22E-01	NS	0.70	5.04E-01	NS	0.56	1.72E-01	NS	-0.79	4.51E-02	NS	-1.58	8.72E-01	NS	-0.14	5.12E-02	NS	-1.05	8.28E-01	NS	0.27	7.50E-01	NS	-0.18	8.54E-01	NS	0.07
F6TIK4	1.30E-02	NS	2.64	3.65E-02	NS	2.23	1.47E-02	NS	2.35	2.86E-02	NS	2.30	3.20E-02	NS	1.15	2.27E-03	NS	1.81	1.72E-01	NS	0.59	4.81E-02	NS	1.06	5.99E-03	NS	0.79	2.17E-01	NS	0.38	1.78E-02	NS	0.83	3.57E-02	NS	1.34	1.29E-01	NS	0.64
F7EI47	2.95E-03	NS	0.45	4.58E-03	NS	0.37	2.85E-02	NS	0.64	6.91E-02	NS	0.33	1.17E-03	NS	0.37	8.98E-02	NS	0.37	5.54E-02	NS	0.30	9.19E-02	NS	0.65	9.70E-01	NS	0.03	9.78E-02	NS	0.37	6.63E-03	NS	0.25	1.54E-01	NS	0.21	1.13E-02	NS	0.46
F7GAT4	8.93E-03	NS	0.51	1.97E-02	NS	0.22	4.40E-02	NS	0.77	9.30E-02	NS	0.44	3.45E-01	NS	0.35	1.55E-02	NS	0.60	2.52E-01	NS	0.44	1.69E-01	NS	0.59	8.54E-03	NS	0.50	4.84E-02	NS	0.39	1.01E-01	NS	0.69	4.55E-01	NS	0.49	2.87E-02	NS	0.81
A0A1D5Q2R8	1.00E-01	NS	0.68	2.43E-01	NS	0.27	4.03E-01	NS	0.28	5.09E-01	NS	0.29	7.40E-01	NS	0.25	3.45E-01	NS	0.45	5.43E-01	NS	0.29	8.20E-01	NS	0.14	3.54E-01	NS	0.51	4.60E-02	NS	0.75	1.76E-01	NS	0.60	1.66E-01	NS	0.66	5.69E-01	NS	0.28
F7HEL0	4.86E-01	NS	0.14	5.39E-01	NS	-0.16	8.36E-01	NS	0.11	9.44E-01	NS	0.00	6.47E-01	NS	0.13	7.74E-01	NS	0.19	5.72E-01	NS	-0.15	5.79E-01	NS	0.46	8.93E-01	NS	0.11	9.52E-01	NS	0.02	4.66E-01	NS	0.35	5.62E-01	NS	0.22	8.57E-01	NS	0.20
A0A1D5QS06	9.73E-02	NS	0.29	1.28E-02	NS	0.27	8.19E-02	NS	0.52	1.50E-02	NS	0.49	1.45E-01	NS	0.25	8.19E-03	NS	0.61	2.82E-01	NS	0.43	2.58E-01	NS	0.63	1.66E-01	NS	0.25	1.03E-01	NS	0.48	1.53E-02	NS	0.37	6.09E-01	NS	0.34	6.89E-02	NS	0.44
F6YK65	3.71E-01	NS	0.18	3.60E-01	NS	-0.24	1.39E-01	NS	0.34	5.38E-01	NS	0.23	9.51E-01	NS	0.00	7.76E-01	NS	0.13	7.35E-01	NS	0.04	8.10E-01	NS	-0.04	7.03E-01	NS	-0.05	3.40E-01	NS	0.29	4.99E-01	NS	0.16	7.12E-01	NS	0.25	5.29E-01	NS	0.24
F6SSW9	9.63E-01	NS	0.00	9.26E-01	NS	0.02	1.95E-01	NS	-0.28	4.67E-01	NS	-0.17	8.20E-01	NS	-0.02	1.61E-01	NS	-0.34	9.79E-01	NS	0.07	2.75E-02	NS	-0.55	2.09E-01	NS	-0.34	5.25E-01	NS	0.11	1.39E-01	NS	-0.34	5.36E-01	NS	0.13	9.07E-01	NS	-0.02
F7F6Y0	3.77E-01	NS	0.17	1.55E-01	NS	0.19	1.17E-01	NS	0.35	6.60E-02	NS	0.33	4.33E-01	NS	0.20	4.44E-03	NS	0.38	6.71E-01	NS	0.13	6.99E-01	NS	0.10	8.93E-01	NS	0.04	5.29E-01	NS	0.14	1.11E-01	NS	0.33	5.95E-01	NS	0.09	1.76E-01	NS	0.18
F7HL22	1.70E-02	NS	1.55	5.42E-02	NS	2.93	8.11E-02	NS	4.18	7.50E-02	NS	2.78	8.67E-02	NS	1.28	4.42E-02	NS	1.53	1.01E-02	NS	1.02	1.76E-01	NS	3.07	5.42E-02	NS	1.39	2.29E-01	NS	0.86	3.86E-02	NS	1.73	6.10E-03	NS	1.75	1.96E-01	NS	1.51
F7H2A5	1.53E-01	NS	1.15	3.41E-01	NS	0.81	2.04E-01	NS	1.01	6.70E-02	NS	1.33	4.42E-01	NS	0.60	4.86E-02	NS	1.08	6.50E-01	NS	-0.22	5.94E-01	NS	-0.21	5.13E-01	NS	0.14	1.45E-01	NS	-0.78	9.26E-01	NS	0.26	5.15E-01	NS	-0.20	4.33E-01	NS	-0.32
H9H3P6	2.59E-01	NS	-0.20	8.46E-01	NS	-0.04	7.84E-01	NS	-0.04	3.49E-01	NS	-0.20	7.23E-01	NS	-0.06	1.07E-01	NS	-0.23	4.59E-01	NS	-0.15	3.85E-01	NS	-0.18	2.27E-01	NS	-0.22	1.71E-01	NS	-0.29	4.40E-02	NS	-0.41	1.68E-01	NS	-0.33	2.46E-01	NS	-0.28
F7DD08	1.20E-01	NS	0.48	6.25E-01	NS	-0.13	6.94E-02	NS	0.49	1.82E-01	NS	0.52	7.24E-01	NS	0.11	1.26E-01	NS	0.50	9.13E-01	NS	0.07	5.94E-01	NS	0.27	3.63E-01	NS	0.34	2.36E-01	NS	0.51	2.04E-02	NS	0.66	2.69E-01	NS	0.58	4.83E-01	NS	0.48
A0A1D5QCC0	3.22E-01	NS	0.43	7.38E-01	NS	0.26	3.79E-01	NS	0.81	3.13E-01	NS	0.68	6.71E-01	NS	0.10	5.50E-01	NS	0.55	1.52E-01	NS	0.46	5.25E-01	NS	0.51	3.04E-01	NS	0.31	5.35E-01	NS	0.44	1.63E-01	NS	0.53	2.15E-01	NS	0.75	2.83E-01	NS	0.72
F7HCH2	1.50E-01	NS	-3.07	5.80E-01	NS	-0.41	9.18E-02	NS	-3.45	1.68E-01	NS	-2.82	1.85E-01	NS	-2.40	2.29E-01	NS	-2.57	3.52E-01	NS	-2.21	5.90E-01	NS	-1.62	5.86E-01	NS	-0.58	2.17E-01	NS	-2.68	6.29E-01	NS	0.21	7.95E-02	NS	-3.58	6.61E-02	NS	-3.78
F7GFB4	2.16E-01	NS	0.50	9.65E-01	NS	-0.05	4.50E-02	NS	0.57	3.20E-01	NS	0.49	9.04E-01	NS	0.20	2.88E-01	NS	0.32	7.64E-01	NS	0.23	5.03E-01	NS	0.26	5.25E-01	NS	0.33	2.72E-01	NS	0.51	1.09E-01	NS	0.61	6.29E-01	NS	0.43	3.22E-01	NS	0.45
F7DHR8	1.03E-02	NS	-1.60	1.36E-01	NS	-0.64	5.13E-01	NS	-0.21	3.33E-01	NS	-0.37	4.77E-01	NS	-0.19	5.47E-01	NS	-0.23	1.79E-01	NS	-0.51	7.84E-01	NS	-0.12	1.84E-01	NS	-0.63	2.65E-01	NS	-0.56	8.93E-01	NS	0.26	5.03E-01	NS	0.14	1.58E-01	NS	-0.72
F6XIG8	1.91E-01	NS	0.31	5.17E-01	NS	-0.22	1.30E-01	NS	0.43	4.83E-01	NS	0.24	9.93E-01	NS	0.03	4.89E-01	NS	0.26	6.41E-01	NS	-0.13	5.52E-01	NS	0.26	8.04E-01	NS	-0.06	3.19E-01	NS	0.34	2.23E-01	NS	0.47	9.56E-01	NS	0.11	4.59E-01	NS	0.17
A0A1D5Q7U7	5.00E-01	NS	-0.09	8.80E-01	NS	-0.02	6.09E-01	NS	0.16	3.89E-01	NS	-0.12	2.63E-01	NS	-0.16	9.07E-01	NS	0.04	7.74E-01	NS	0.12	9.81E-01	NS	0.03	8.78E-01	NS	-0.01	4.40E-01	NS	-0.12	6.56E-01	NS	-0.06	3.55E-01	NS	-0.14	9.64E-01	NS	0.01
F6TP36	3.92E-01	NS	0.24	2.89E-01	NS	-0.19	2.80E-01	NS	0.40	5.37E-01	NS	0.14	6.29E-01	NS	-0.09	1.69E-01	NS	0.25	6.48E-01	NS	-0.08	8.93E-01	NS	0.01	6.17E-01	NS	0.11	6.45E-02	NS	0.26	8.85E-02	NS	0.32	6.09E-01	NS	0.24	6.59E-01	NS	0.28
F6ZGR2	8.67E-01	NS	0.06	9.98E-02	NS	-0.39	3.07E-01	NS	0.36	8.57E-01	NS	0.05	9.90E-01	NS	0.03	3.54E-01	NS	0.24	7.68E-01	NS	-0.07	4.70E-01	NS	0.30	8.80E-01	NS	-0.03	6.73E-01	NS	0.09	4.12E-02	NS	0.45	9.22E-01	NS	0.03	7.98E-01	NS	0.14
F6WSG1	5.94E-01	NS	0.13	8.44E-01	NS	-0.37	2.46E-01	NS	0.57	2.94E-01	NS	0.30	9.93E-01	NS	-0.21	3.06E-01	NS	0.76	8.47E-01	NS	-0.13	4.70E-01	NS	0.53	5.73E-01	NS	0.09	7.53E-01	NS	0.11	3.37E-01	NS	0.69	9.14E-01	NS	-0.21	6.99E-01	NS	0.50
F7F8G8	9.63E-01	NS	0.28	5.19E-01	NS	0.66	1.79E-01	NS	0.94	1.94E-01	NS	1.02	1.50E-02	NS	1.64	3.73E-04	NS	2.15	2.24E-01	NS	1.34	2.22E-02	NS	1.96	6.34E-02	NS	1.46	7.68E-01	NS	0.33	1.36E-01	NS	0.41	7.13E-01	NS	0.24	3.25E-01	NS	0.92
F7HSL6	2.31E-01	NS	0.97	4.97E-01	NS	0.77	1.09E-01	NS	1.56	2.62E-01	NS	0.96	7.21E-01	NS	0.98	3.68E-01	NS	0.40	4.29E-01	NS	0.34	9.11E-01	NS	0.37	8.90E-01	NS	-0.01	8.80E-01	NS	-0.20	3.99E-01	NS	0.34	3.89E-01	NS	-1.29	7.32E-01	NS	0.22
A0A1D5R0L5	4.76E-01	NS	0.13	7.62E-01	NS	-0.04	2.42E-01	NS	0.75	3.89E-01	NS	0.49	6.12E-01	NS	-0.09	1.88E-01	NS	0.55	3.12E-01	NS	0.27	6.35E-01	NS	0.29	8.78E-02	NS	-0.39	3.03E-01	NS	0.45	4.94E-02	NS	0.58	7.80E-01	NS	0.23	4.66E-01	NS	0.24
F7B8S4	5.94E-01	NS	0.71	2.51E-01	NS	0.92	7.44E-02	NS	1.21	4.87E-01	NS	0.86	2.91E-01	NS	0.96	7.64E-01	NS	1.04	7.32E-01	NS	-0.02	6.92E-01	NS	0.60	3.38E-01	NS	0.82	8.71E-01	NS	0.24	5.05E-01	NS	-0.16	5.70E-01	NS	0.53	2.16E-01	NS	1.32
F6YW79	2.50E-02	NS	0.60	9.74E-01	NS	0.01	4.98E-01	NS	0.38	4.15E-01	NS	0.42	6.91E-01	NS	0.13	8.51E-01	NS	0.03	9.01E-01	NS	0.00	5.02E-01	NS	-0.16	7.73E-01	NS	0.17	1.06E-01	NS	0.58	9.12E-02	NS	0.48	3.63E-01	NS	0.64	3.98E-01	NS	0.54
F7GHR4	4.65E-01	NS	-0.23	6.00E-01	NS	-0.16	1.45E-01	NS	-0.51	2.65E-01	NS	-0.34	6.35E-01	NS	0.07	3.34E-01	NS	-0.29	6.86E-01	NS	0.10	9.68E-01	NS	-0.04	7.28E-01	NS	0.05	9.02E-01	NS	0.04	1.47E-01	NS	-0.45	6.65E-01	NS	-0.15	7.20E-01	NS	-0.11
A0A1D5Q6N1	1.94E-01	NS	0.45	3.71E-01	NS	-0.23	6.35E-02	NS	0.65	3.29E-01	NS	0.44	7.70E-01	NS	-0.07	2.41E-01	NS	0.40	9.18E-01	NS	0.01	7.91E-01	NS	0.12	8.07E-01	NS	0.11	2.68E-01	NS	0.49	4.06E-02	NS	0.55	6.46E-01	NS	0.44	6.05E-01	NS	0.45
F6ZZL9	5.76E-01	NS	-0.08	9.91E-01	NS	0.02	1.79E-01	NS	-0.20	7.71E-01	NS	-0.03	6.47E-01	NS	0.20	6.85E-01	NS	-0.05	8.15E-01	NS	-0.02	5.80E-01	NS	-0.09	7.09E-01	NS	0.11	2.71E-01	NS	-0.20	1.47E-01	NS	-0.21	9.31E-01	NS	0.10	6.91E-01	NS	-0.03
F7GHV1	2.07E-01	NS	0.48	8.42E-01	NS	-0.02	2.02E-01	NS	0.39	3.55E-01	NS	0.30	8.46E-01	NS	0.11	2.89E-01	NS	0.35	9.94E-01	NS	0.05	4.40E-01	NS	0.27	3.85E-01	NS	0.50	5.92E-02	NS	0.63	9.99E-02	NS	0.61	5.41E-01	NS	0.40	4.60E-01	NS	0.36
H9FY19	5.81E-01	NS	0.15	4.71E-02	NS	0.54	8.61E-01	NS	0.01	7.44E-01	NS	0.10	5.47E-01	NS	0.31	9.58E-01	NS	0.01	5.72E-01	NS	0.38	6.55E-01	NS	-0.09	5.45E-01	NS	0.35	2.97E-01	NS	0.56	6.86E-01	NS	-0.11	8.98E-02	NS	0.48	1.76E-01	NS	0.31
F7HF51	1.72E-01	NS	0.37	5.59E-01	NS	-0.17	1.85E-01	NS	0.71	3.00E-01	NS	0.31	8.06E-01	NS	0.10	2.64E-01	NS	0.44	5.91E-01	NS	-0.15	4.18E-01	NS	0.38	7.02E-01	NS	-0.06	6.04E-02	NS	0.46	1.26E-01	NS	0.42	6.64E-01	NS	0.24	8.00E-01	NS	0.19
F7DLB8	1.88E-01	NS	0.70	5.09E-01	NS	0.24	2.26E-01	NS	0.69	9.90E-01	NS	0.35	6.38E-01	NS	0.08	2.35E-01	NS	0.83	5.65E-01	NS	0.55	8.31E-01	NS	0.80	5.36E-01	NS	0.49	4.61E-01	NS	0.60	1.17E-01	NS	1.05	8.88E-01	NS	0.65	4.53E-01	NS	0.87
B3Y6B8	5.82E-01	NS	0.15	7.46E-01	NS	-0.05	4.46E-02	NS	0.74	9.26E-02	NS	0.57	1.35E-01	NS	0.48	1.09E-02	NS	0.82	2.37E-01	NS	0.53	1.87E-02	NS	0.81	1.42E-02	NS	0.64	2.06E-02	NS	0.65	7.43E-02	NS	0.60	2.04E-01	NS	0.35	2.90E-01	NS	0.32
F7B786	3.48E-01	NS	0.37	2.12E-01	NS	2.01	8.38E-02	NS	0.63	7.95E-01	NS	-0.03	4.46E-01	NS	0.76	1.14E-01	NS	1.09	9.99E-01	NS	0.01	5.99E-01	NS	0.11	6.11E-01	NS	0.21	2.73E-01	NS	-0.50	9.07E-01	NS	0.06	4.62E-01	NS	-0.32	1.16E-01	NS	1.07
F7F4G6	7.36E-01	NS	0.10	5.63E-01	NS	0.09	5.15E-01	NS	-0.10	6.89E-01	NS	-0.05	7.04E-01	NS	0.07	9.53E-01	NS	0.04	8.21E-01	NS	-0.02	8.06E-01	NS	-0.03	2.41E-01	NS	0.24	2.70E-01	NS	-0.27	1.98E-01	NS	0.21	7.68E-01	NS	-0.01	8.64E-02	NS	-0.27
F7DNJ5	3.49E-01	NS	0.19	5.70E-01	NS	0.08	1.50E-01	NS	0.78	4.05E-01	NS	0.19	5.08E-01	NS	0.17	1.31E-01	NS	0.68	5.39E-01	NS	0.20	7.50E-03	NS	0.78	8.04E-01	NS	-0.06	4.19E-01	NS	0.16	4.93E-02	NS	0.48	2.90E-01	NS	0.22	1.21E-01	NS	0.34
F6YYK7	4.57E-01	NS	0.31	2.43E-01	NS	-0.21	2.13E-01	NS	0.49	2.64E-01	NS	0.40	6.64E-01	NS	0.08	1.48E-01	NS	0.47	2.86E-01	NS	0.22	4.92E-01	NS	0.40	3.92E-01	NS	0.29	2.04E-01	NS	0.58	4.82E-02	NS	0.55	4.15E-01	NS	0.32	5.29E-01	NS	0.43
O19092	3.71E-01	NS	0.31	5.53E-02	NS	0.78	1.44E-01	NS	-0.86	3.93E-01	NS	-0.36	5.79E-01	NS	0.64	2.42E-01	NS	-0.63	8.67E-01	NS	0.29	8.69E-01	NS	0.07	5.44E-01	NS	-0.18	7.22E-01	NS	0.26	5.33E-01	NS	-0.28	1.03E-01	NS	0.58	5.40E-01	NS	-0.14
F7CC91	8.81E-01	NS	0.11	8.42E-01	NS	0.08	8.37E-02	NS	0.70	4.33E-01	NS	0.17	9.55E-01	NS	-0.02	3.75E-01	NS	0.40	9.50E-01	NS	0.05	1.65E-01	NS	0.65	9.82E-01	NS	0.02	5.26E-01	NS	-0.17	3.96E-02	NS	0.62	3.69E-01	NS	-0.23	7.97E-01	NS	0.04
F7B749	5.52E-01	NS	0.22	9.48E-01	NS	-0.01	5.83E-02	NS	0.35	7.82E-01	NS	0.14	3.88E-01	NS	0.19	2.16E-01	NS	0.43	7.77E-01	NS	0.05	8.50E-01	NS	0.01	7.86E-01	NS	0.06	4.40E-01	NS	0.23	1.09E-01	NS	0.28	1.92E-01	NS	0.29	8.56E-01	NS	0.09
F6SL92	5.40E-01	NS	0.14	6.81E-01	NS	-0.17	3.83E-01	NS	0.19	4.26E-01	NS	0.19	8.85E-01	NS	0.09	3.01E-01	NS	0.27	5.67E-01	NS	0.33	2.42E-01	NS	0.51	4.08E-01	NS	0.49	2.32E-01	NS	0.45	2.43E-01	NS	0.41	9.40E-01	NS	0.00	6.96E-01	NS	0.07
F7H532	1.46E-01	NS	-0.42	8.85E-01	NS	0.17	5.04E-01	NS	-0.19	9.10E-02	NS	-0.61	8.25E-01	NS	-0.07	3.50E-01	NS	-0.29	8.47E-01	NS	-0.01	4.10E-01	NS	-0.23	3.79E-01	NS	-0.25	2.69E-01	NS	-0.28	8.09E-02	NS	-0.53	8.57E-01	NS	-0.04	6.00E-01	NS	-0.15
F6Z7P7	5.98E-01	NS	0.18	8.55E-01	NS	-0.01	2.30E-01	NS	0.54	2.80E-01	NS	0.50	6.82E-01	NS	0.19	1.56E-01	NS	0.51	6.74E-01	NS	0.25	7.21E-01	NS	0.36	5.34E-01	NS	0.12	3.36E-01	NS	0.40	3.01E-01	NS	0.67	7.15E-01	NS	0.34	5.24E-01	NS	0.64
F7H5Q6	6.32E-01	NS	0.20	1.06E-01	NS	-0.40	3.01E-01	NS	0.37	6.56E-01	NS	-0.11	6.97E-01	NS	-0.06	6.47E-01	NS	0.10	3.73E-01	NS	-0.33	5.34E-01	NS	-0.17	2.85E-01	NS	-0.28	5.68E-01	NS	-0.14	2.69E-02	NS	0.53	9.50E-01	NS	0.02	6.75E-01	NS	0.24
A0A1D5QKX6	6.66E-01	NS	0.20	4.69E-01	NS	-0.30	3.89E-01	NS	0.24	8.18E-01	NS	-0.08	9.51E-01	NS	0.11	3.77E-01	NS	0.22	5.59E-01	NS	-0.16	8.87E-01	NS	-0.03	7.41E-01	NS	-0.13	8.34E-01	NS	-0.08	7.31E-02	NS	0.63	7.73E-01	NS	0.17	6.31E-01	NS	0.18
F6TLR3	4.18E-01	NS	0.29	8.08E-01	NS	-0.13	1.35E-01	NS	0.43	3.43E-01	NS	0.24	9.43E-01	NS	-0.05	1.25E-01	NS	0.48	3.88E-01	NS	0.33	7.07E-02	NS	0.68	2.46E-01	NS	0.49	6.87E-02	NS	0.56	2.22E-01	NS	0.42	8.88E-01	NS	0.05	8.68E-01	NS	0.04
F6UQI4	5.70E-02	NS	0.28	1.78E-01	NS	0.11	4.19E-01	NS	0.17	1.33E-01	NS	0.29	3.90E-01	NS	0.13	4.12E-01	NS	0.15	1.92E-01	NS	0.14	5.72E-01	NS	0.12	2.34E-01	NS	0.15	1.60E-01	NS	0.29	5.72E-01	NS	0.07	4.18E-01	NS	0.26	4.51E-01	NS	0.19
F6WR34	1.06E-01	NS	-0.25	9.47E-01	NS	-0.01	1.41E-01	NS	-0.43	4.67E-01	NS	-0.14	9.30E-01	NS	0.04	1.19E-01	NS	-0.40	8.19E-01	NS	-0.02	4.27E-01	NS	-0.15	3.44E-01	NS	-0.15	1.07E-01	NS	-0.33	7.15E-02	NS	-0.37	3.88E-01	NS	-0.20	3.12E-01	NS	-0.24
F6UR30	5.29E-01	NS	-0.24	8.70E-01	NS	-0.08	9.48E-01	NS	0.26	9.45E-01	NS	0.06	8.94E-01	NS	0.28	3.73E-01	NS	0.50	1.63E-01	NS	-0.79	6.15E-01	NS	-0.20	2.78E-01	NS	-0.52	2.03E-01	NS	-0.66	7.28E-01	NS	-0.20	1.10E-01	NS	-0.83	4.41E-01	NS	0.44
F6UM69	1.81E-01	NS	-0.27	3.98E-01	NS	0.15	2.22E-01	NS	-0.40	8.04E-01	NS	-0.03	9.66E-01	NS	0.03	3.92E-01	NS	-0.17	7.70E-01	NS	0.13	9.15E-01	NS	0.00	3.13E-01	NS	0.13	8.15E-01	NS	-0.03	2.39E-01	NS	-0.27	3.27E-01	NS	-0.24	2.95E-01	NS	-0.26
F7AEK9	5.95E-01	NS	0.35	8.33E-01	NS	0.04	1.22E-01	NS	0.79	4.43E-01	NS	-0.36	4.62E-01	NS	0.72	8.91E-02	NS	1.32	3.87E-01	NS	0.44	9.55E-01	NS	0.14	4.81E-01	NS	0.19	2.49E-01	NS	-0.54	2.41E-01	NS	0.43	8.01E-01	NS	0.05	7.75E-02	NS	0.99
F6RNZ2	2.39E-01	NS	-0.45	4.47E-02	NS	-0.72	8.72E-01	NS	0.08	5.49E-01	NS	-0.20	2.83E-01	NS	-0.38	1.22E-01	NS	-0.57	1.22E-01	NS	-0.55	1.18E-01	NS	-0.69	5.95E-02	NS	-0.67	1.69E-01	NS	-0.52	2.59E-01	NS	-0.36	5.55E-01	NS	-0.21	8.45E-01	NS	0.02
F6YJW2	9.48E-01	NS	0.03	8.01E-01	NS	-0.04	4.33E-02	NS	0.35	5.33E-01	NS	0.10	9.17E-01	NS	0.06	4.73E-01	NS	0.09	5.75E-01	NS	-0.10	5.91E-01	NS	0.08	8.79E-01	NS	0.00	7.69E-01	NS	0.03	2.03E-01	NS	0.28	7.52E-01	NS	-0.05	7.20E-01	NS	0.12
F6TNV9	8.69E-01	NS	0.02	5.57E-01	NS	-0.10	7.01E-01	NS	0.20	9.26E-01	NS	-0.01	5.12E-01	NS	-0.09	5.39E-01	NS	0.14	2.44E-01	NS	-0.21	9.72E-01	NS	0.05	1.30E-01	NS	-0.33	6.64E-01	NS	-0.06	5.74E-01	NS	0.21	5.28E-01	NS	-0.11	5.51E-01	NS	-0.13
F6U2J6	3.81E-01	NS	-0.17	6.86E-01	NS	0.05	2.06E-01	NS	-0.26	4.52E-01	NS	-0.15	7.47E-01	NS	-0.05	2.88E-01	NS	-0.25	5.15E-01	NS	-0.15	1.88E-01	NS	-0.29	4.59E-01	NS	-0.15	1.60E-01	NS	-0.31	9.66E-02	NS	-0.47	5.50E-01	NS	-0.13	1.26E-01	NS	-0.34
F7FQN1	3.62E-01	NS	0.87	1.06E-01	NS	1.47	3.17E-01	NS	0.29	4.12E-01	NS	0.34	7.33E-01	NS	0.08	4.36E-01	NS	1.01	7.60E-01	NS	0.39	8.48E-01	NS	0.03	3.85E-01	NS	0.40	2.07E-01	NS	0.64	1.92E-01	NS	1.12	3.25E-01	NS	0.73	2.37E-01	NS	0.53
F7B5G7	2.27E-01	NS	0.76	2.85E-01	NS	0.77	5.32E-02	NS	1.08	7.81E-01	NS	0.24	9.34E-02	NS	0.83	3.28E-02	NS	1.34	5.88E-02	NS	0.78	2.01E-01	NS	0.48	2.70E-01	NS	0.53	7.81E-01	NS	-0.14	2.57E-01	NS	0.44	2.21E-01	NS	0.74	6.19E-02	NS	1.95
F7HQV3	4.73E-01	NS	-0.13	8.25E-02	NS	-0.30	7.09E-01	NS	0.09	2.53E-01	NS	0.18	5.32E-01	NS	-0.08	8.62E-01	NS	0.10	2.51E-01	NS	-0.68	5.69E-01	NS	-0.10	1.23E-01	NS	-0.31	4.97E-01	NS	-0.12	7.39E-01	NS	0.12	5.76E-01	NS	-0.10	8.78E-01	NS	0.02
F6WG91	8.15E-01	NS	-0.04	6.91E-01	NS	-0.07	4.38E-01	NS	0.20	1.89E-01	NS	-0.34	8.28E-01	NS	0.03	4.61E-01	NS	0.10	8.53E-01	NS	-0.03	4.23E-01	NS	-0.20	4.33E-01	NS	-0.17	2.86E-01	NS	-0.26	8.87E-01	NS	0.06	1.76E-01	NS	-0.36	1.03E-01	NS	-0.35
A0A1D5QXZ0	1.48E-01	NS	1.01	5.18E-02	NS	1.08	2.97E-01	NS	1.14	2.59E-01	NS	0.69	1.47E-02	NS	1.41	6.02E-01	NS	0.56	1.28E-01	NS	1.03	1.10E-01	NS	0.82	6.31E-02	NS	1.09	1.08E-01	NS	1.07	5.35E-01	NS	0.38	4.60E-01	NS	1.02	1.65E-01	NS	0.85
F6WIY2	3.60E-01	NS	0.37	6.02E-01	NS	0.19	5.37E-01	NS	0.35	5.01E-01	NS	0.37	8.76E-01	NS	0.05	8.20E-01	NS	0.16	3.92E-01	NS	-0.34	7.31E-01	NS	0.10	6.92E-01	NS	-0.13	8.28E-01	NS	-0.07	6.59E-01	NS	-0.18	5.39E-01	NS	-0.24	6.25E-01	NS	0.16
F7FJQ0	4.43E-01	NS	-0.23	7.82E-01	NS	0.04	1.68E-01	NS	-0.40	4.93E-01	NS	-0.21	6.89E-01	NS	-0.12	4.25E-01	NS	-0.24	7.96E-01	NS	-0.09	5.52E-01	NS	-0.17	6.04E-01	NS	-0.15	2.81E-01	NS	-0.36	2.31E-01	NS	-0.34	2.14E-01	NS	-0.39	3.98E-01	NS	-0.26
A0A1D5QF45	7.50E-01	NS	0.14	9.08E-01	NS	0.16	3.44E-01	NS	0.82	5.79E-01	NS	0.54	9.38E-01	NS	-0.01	7.06E-01	NS	0.48	5.51E-01	NS	-0.14	5.03E-01	NS	0.63	3.59E-01	NS	0.26	4.63E-01	NS	0.55	3.37E-01	NS	0.66	6.88E-01	NS	0.52	4.68E-01	NS	0.64
F7H1V9	1.08E-01	NS	0.44	6.26E-01	NS	0.09	4.70E-01	NS	0.40	4.48E-01	NS	0.39	8.99E-01	NS	0.03	4.93E-01	NS	-0.17	7.21E-01	NS	-0.08	9.93E-01	NS	0.05	4.30E-01	NS	0.26	2.70E-01	NS	0.54	1.42E-01	NS	0.44	6.72E-01	NS	0.36	5.41E-01	NS	0.22
A0A1D5QYD0	2.64E-01	NS	0.23	1.65E-01	NS	0.38	1.20E-01	NS	0.33	5.04E-01	NS	0.22	4.06E-01	NS	0.20	5.91E-01	NS	0.34	7.80E-01	NS	0.19	6.30E-01	NS	-0.09	1.82E-01	NS	0.30	7.89E-02	NS	0.35	6.89E-01	NS	0.17	4.79E-01	NS	0.21	6.09E-01	NS	0.13
F7HP21	6.21E-01	NS	0.23	6.12E-01	NS	-0.24	2.74E-01	NS	0.51	5.17E-01	NS	0.23	8.75E-01	NS	-0.03	4.00E-01	NS	0.48	9.38E-01	NS	-0.05	8.45E-01	NS	0.17	7.74E-01	NS	0.15	4.59E-01	NS	0.42	2.32E-01	NS	0.40	6.58E-01	NS	0.40	5.49E-01	NS	0.54
F7FQN6	6.54E-01	NS	-0.47	9.59E-01	NS	-0.05	4.29E-01	NS	0.23	5.40E-01	NS	-0.64	4.11E-01	NS	-0.79	9.33E-01	NS	-0.20	8.34E-01	NS	0.25	4.08E-01	NS	0.60	7.36E-01	NS	0.06	8.10E-01	NS	0.25	3.30E-01	NS	0.45	7.91E-01	NS	-0.34	6.15E-01	NS	0.32
F7EL64	5.13E-01	NS	0.56	8.80E-02	NS	1.81	1.80E-01	NS	0.99	5.63E-01	NS	0.42	1.74E-01	NS	2.11	3.00E-01	NS	1.29	4.85E-01	NS	0.82	3.07E-01	NS	0.82	6.84E-02	NS	1.44	6.16E-02	NS	1.01	1.55E-01	NS	1.02	5.76E-01	NS	0.74	2.40E-01	NS	0.44
F7ALN6	7.56E-01	NS	0.09	7.25E-01	NS	-0.04	2.23E-01	NS	0.38	2.21E-01	NS	0.22	8.06E-01	NS	-0.03	4.74E-01	NS	0.22	6.88E-01	NS	0.07	6.77E-01	NS	0.16	8.17E-01	NS	-0.02	3.17E-01	NS	0.18	7.70E-01	NS	0.06	9.02E-01	NS	0.07	8.58E-01	NS	0.07
F7DIN9	8.70E-01	NS	0.12	1.89E-01	NS	-0.32	3.03E-01	NS	0.41	9.21E-01	NS	-0.01	6.71E-01	NS	0.10	4.39E-01	NS	0.52	9.31E-01	NS	0.00	2.01E-01	NS	0.67	9.48E-01	NS	0.00	3.83E-01	NS	0.24	4.23E-01	NS	-0.22	6.73E-01	NS	0.18	6.69E-01	NS	-0.10
F7HT52	1.07E-01	NS	-1.45	6.32E-01	NS	-0.51	3.24E-01	NS	-0.92	5.83E-01	NS	-0.55	4.13E-01	NS	-0.66	4.06E-01	NS	-0.78	7.00E-01	NS	-0.29	8.05E-01	NS	-0.28	8.90E-01	NS	-0.30	3.71E-01	NS	-0.85	4.50E-01	NS	-0.66	7.09E-01	NS	-0.41	5.80E-02	NS	-1.73
F7H7G9	4.33E-01	NS	-0.93	9.63E-01	NS	-0.32	4.53E-01	NS	0.15	4.69E-01	NS	-0.10	4.05E-01	NS	-1.46	6.35E-01	NS	-0.44	8.56E-01	NS	-0.53	8.67E-01	NS	0.58	8.86E-01	NS	1.42	7.26E-01	NS	0.97	7.96E-01	NS	-0.63	3.46E-01	NS	-0.02	3.79E-01	NS	0.21
A0A1D5QJP4	5.27E-01	NS	-0.16	9.24E-01	NS	-0.04	7.31E-01	NS	0.22	3.77E-01	NS	-0.19	9.46E-01	NS	0.01	3.04E-01	NS	-0.23	7.27E-01	NS	-0.08	9.11E-01	NS	-0.04	7.32E-01	NS	-0.08	2.08E-01	NS	-0.28	2.79E-01	NS	-0.26	2.25E-01	NS	-0.35	1.21E-01	NS	-0.43
F7HR41	5.24E-01	NS	0.34	3.33E-01	NS	0.30	2.89E-01	NS	0.62	2.22E-01	NS	0.25	3.96E-01	NS	0.13	4.28E-01	NS	0.51	5.53E-01	NS	0.08	8.56E-02	NS	0.45	6.16E-01	NS	0.15	8.55E-02	NS	0.40	2.90E-01	NS	0.16	3.39E-01	NS	0.36	1.86E-01	NS	0.34
F6T094	7.92E-01	NS	-0.06	9.78E-01	NS	-0.04	3.95E-01	NS	0.44	5.98E-01	NS	0.47	9.84E-01	NS	0.61	1.54E-01	NS	0.91	1.59E-01	NS	1.01	6.82E-02	NS	1.65	3.76E-01	NS	1.26	3.87E-01	NS	0.68	4.40E-01	NS	0.77	8.95E-01	NS	0.09	9.32E-01	NS	-0.16
G7NLH4	6.76E-01	NS	0.54	3.15E-01	NS	0.46	4.39E-01	NS	0.36	3.99E-02	NS	1.21	1.38E-01	NS	0.72	5.66E-02	NS	1.45	8.91E-02	NS	1.16	1.23E-01	NS	1.26	2.88E-01	NS	0.49	9.18E-01	NS	0.02	8.69E-01	NS	-0.06	9.70E-01	NS	0.12	4.28E-01	NS	0.39
F7FY72	7.77E-01	NS	-0.11	2.81E-01	NS	0.31	8.10E-01	NS	-0.10	9.01E-01	NS	-0.02	7.55E-01	NS	0.09	6.57E-01	NS	0.15	3.62E-01	NS	0.29	9.46E-01	NS	0.00	6.74E-01	NS	0.13	7.16E-01	NS	0.10	7.10E-01	NS	0.07	7.43E-01	NS	0.03	7.21E-01	NS	-0.14
F6Z0T5	6.52E-01	NS	0.49	7.68E-01	NS	-0.03	3.60E-01	NS	1.04	6.50E-01	NS	1.06	3.09E-01	NS	0.84	5.84E-01	NS	0.27	6.07E-01	NS	0.43	9.02E-01	NS	1.54	5.11E-01	NS	-0.67	4.47E-01	NS	1.10	9.75E-01	NS	-0.03	3.92E-01	NS	-1.41	9.22E-01	NS	-0.44
F7CNK6	8.65E-01	NS	0.07	8.38E-01	NS	0.23	4.25E-01	NS	0.90	6.54E-01	NS	0.27	7.10E-01	NS	-0.12	5.93E-01	NS	1.04	4.21E-01	NS	0.21	7.47E-01	NS	0.99	6.35E-01	NS	-0.20	8.68E-01	NS	0.13	4.22E-01	NS	0.36	3.48E-01	NS	0.64	5.50E-01	NS	0.30
F6Y7Q4	5.44E-01	NS	0.34	4.71E-01	NS	-0.21	1.61E-01	NS	0.52	3.45E-01	NS	0.26	5.63E-01	NS	-0.18	3.58E-01	NS	0.40	4.97E-01	NS	-0.20	8.46E-01	NS	0.00	9.50E-01	NS	0.06	4.24E-01	NS	0.38	3.04E-02	NS	0.57	7.38E-01	NS	0.20	6.33E-01	NS	0.39
A0A1D5Q3Z5	6.39E-01	NS	0.27	2.70E-01	NS	0.55	7.07E-01	NS	-0.12	6.41E-01	NS	0.22	7.92E-01	NS	0.17	8.84E-01	NS	-0.11	4.11E-01	NS	0.42	8.98E-01	NS	-0.26	6.92E-01	NS	0.14	4.53E-01	NS	0.28	9.64E-01	NS	-0.02	5.59E-01	NS	0.23	6.62E-01	NS	0.15
A0A1D5R9B8	3.22E-01	NS	0.80	6.58E-01	NS	0.20	1.74E-01	NS	1.31	3.02E-01	NS	0.72	6.09E-01	NS	0.08	9.25E-01	NS	-0.79	3.64E-01	NS	0.05	1.70E-01	NS	0.76	7.69E-01	NS	-0.14	5.64E-01	NS	0.72	8.25E-01	NS	-1.05	1.68E-01	NS	1.06	2.01E-01	NS	0.71
F7DNI9	5.00E-01	NS	0.17	8.10E-01	NS	-0.05	3.57E-01	NS	0.17	4.45E-01	NS	-0.21	7.19E-01	NS	0.18	9.45E-01	NS	0.51	6.95E-01	NS	-0.08	2.42E-01	NS	-0.35	2.33E-01	NS	-0.54	9.93E-01	NS	0.14	7.86E-02	NS	-0.73	4.48E-01	NS	-0.21	9.00E-01	NS	-0.03
F7BTD3	8.87E-01	NS	-0.01	2.28E-01	NS	-0.32	6.78E-01	NS	0.13	7.47E-01	NS	-0.09	8.38E-01	NS	-0.06	2.13E-01	NS	0.35	9.06E-01	NS	-0.05	6.16E-01	NS	0.21	8.42E-01	NS	0.01	7.82E-01	NS	0.06	9.56E-02	NS	0.34	7.49E-01	NS	0.06	9.77E-01	NS	0.01
F6PJA1	1.61E-02	NS	2.00	7.01E-01	NS	0.31	1.40E-01	NS	1.41	1.71E-01	NS	0.65	1.73E-01	NS	0.68	7.47E-01	NS	0.84	3.63E-01	NS	1.30	7.42E-02	NS	1.16	1.02E-01	NS	1.99	1.07E-01	NS	0.85	9.60E-02	NS	0.91	1.07E-01	NS	1.56	6.04E-01	NS	0.99
F6SW75	4.23E-01	NS	0.36	9.92E-01	NS	0.04	3.56E-01	NS	0.33	5.13E-01	NS	0.26	9.33E-01	NS	0.03	8.67E-01	NS	0.06	7.32E-01	NS	0.12	7.00E-01	NS	-0.10	9.77E-01	NS	0.12	4.82E-01	NS	0.31	6.46E-01	NS	0.08	4.91E-01	NS	0.39	4.79E-01	NS	0.40
A0A1D5QLP0	5.55E-01	NS	0.63	9.92E-01	NS	-0.01	9.23E-01	NS	0.14	5.70E-01	NS	0.24	8.24E-01	NS	-0.06	7.35E-01	NS	0.19	8.97E-01	NS	-0.18	9.82E-01	NS	-0.06	8.45E-01	NS	0.29	4.65E-01	NS	0.38	6.43E-01	NS	0.22	5.51E-01	NS	0.20	6.39E-01	NS	0.43
F7FPV8	5.25E-01	NS	0.11	2.13E-01	NS	-0.92	7.99E-01	NS	-0.05	4.33E-01	NS	-0.61	9.07E-01	NS	-0.05	8.12E-01	NS	0.27	7.67E-01	NS	-0.13	4.78E-01	NS	0.46	6.00E-01	NS	-0.47	6.20E-01	NS	-0.15	7.19E-01	NS	0.20	7.20E-01	NS	-0.36	8.71E-01	NS	-0.01
H9FXZ3	6.91E-01	NS	0.47	4.30E-01	NS	0.57	2.04E-01	NS	0.59	5.82E-01	NS	0.19	4.56E-01	NS	0.28	1.11E-01	NS	0.71	8.70E-01	NS	0.14	9.90E-01	NS	0.20	7.87E-01	NS	0.08	6.02E-01	NS	0.32	2.41E-01	NS	0.56	4.04E-01	NS	0.31	5.58E-01	NS	0.55
F7HQ03	4.91E-01	NS	0.61	4.68E-01	NS	0.54	1.35E-01	NS	0.87	2.77E-01	NS	0.73	7.66E-01	NS	0.31	3.95E-01	NS	0.71	6.58E-01	NS	0.03	3.51E-01	NS	0.65	3.91E-01	NS	0.30	2.10E-01	NS	0.70	6.21E-01	NS	0.16	1.00E+00	NS	-0.03	6.52E-01	NS	0.51
A0A1D5QUE8	3.64E-01	NS	0.12	4.18E-01	NS	0.12	1.69E-01	NS	0.62	7.20E-01	NS	0.05	5.99E-01	NS	-0.10	1.66E-01	NS	0.39	6.42E-02	NS	0.38	2.76E-02	NS	0.84	7.45E-02	NS	0.42	5.43E-01	NS	0.09	2.96E-02	NS	0.50	8.50E-01	NS	0.03	1.29E-01	NS	0.38
A0A1D5QD27	7.25E-01	NS	-0.24	8.34E-01	NS	0.05	6.35E-01	NS	-0.31	5.85E-01	NS	-0.34	7.81E-01	NS	-0.17	4.71E-01	NS	-0.47	8.75E-01	NS	-0.05	5.87E-01	NS	-0.35	9.76E-01	NS	-0.09	7.47E-01	NS	-0.20	4.97E-01	NS	-0.44	6.85E-01	NS	-0.23	8.00E-01	NS	-0.20
F6Z0R5	5.03E-01	NS	-0.37	6.51E-01	NS	-0.47	6.52E-01	NS	-0.55	9.15E-01	NS	-0.16	9.90E-01	NS	-0.49	6.40E-01	NS	-0.31	6.07E-01	NS	-0.65	6.49E-01	NS	-0.09	6.16E-01	NS	-0.63	7.87E-01	NS	-0.18	3.68E-01	NS	-1.11	4.62E-01	NS	-0.58	4.15E-01	NS	-0.63
F7F6V9	8.48E-01	NS	0.11	7.82E-01	NS	-0.23	6.21E-01	NS	0.22	9.79E-01	NS	-0.14	9.77E-01	NS	-0.18	8.83E-01	NS	0.05	9.82E-01	NS	-0.10	7.14E-01	NS	-0.29	6.29E-01	NS	-0.33	8.89E-01	NS	0.02	7.29E-01	NS	0.13	8.46E-01	NS	-0.01	9.76E-01	NS	0.11
F6Y0N9	6.37E-01	NS	0.19	4.48E-01	NS	-0.26	6.13E-01	NS	0.09	8.04E-01	NS	-0.11	9.27E-01	NS	-0.08	4.01E-01	NS	0.38	1.84E-01	NS	0.37	8.82E-01	NS	0.06	8.06E-01	NS	-0.10	8.52E-01	NS	0.08	9.42E-01	NS	0.00	7.54E-01	NS	0.17	6.61E-01	NS	0.37
A0A1D5QSH6	5.59E-01	NS	-0.13	8.33E-01	NS	0.07	6.92E-01	NS	-0.09	9.41E-01	NS	0.01	7.91E-01	NS	0.05	8.54E-01	NS	-0.05	7.29E-01	NS	-0.08	8.98E-01	NS	0.01	8.71E-01	NS	-0.05	7.97E-01	NS	-0.05	3.00E-01	NS	-0.23	4.82E-01	NS	-0.16	3.52E-01	NS	-0.23
F7H9D9	4.99E-01	NS	-0.24	8.85E-01	NS	0.05	8.57E-01	NS	0.10	8.61E-01	NS	0.02	4.24E-01	NS	-0.31	6.82E-01	NS	0.24	7.95E-01	NS	0.13	9.52E-01	NS	0.06	8.90E-01	NS	0.07	9.71E-01	NS	0.05	6.46E-01	NS	-0.10	3.09E-01	NS	-0.42	9.88E-01	NS	-0.01
F6VKN1	9.25E-01	NS	-0.15	6.62E-02	NS	0.97	5.69E-01	NS	0.56	7.88E-01	NS	0.14	1.80E-01	NS	1.11	2.82E-02	NS	1.55	2.65E-01	NS	0.89	4.80E-01	NS	0.62	2.11E-01	NS	0.80	9.19E-01	NS	0.10	4.37E-01	NS	0.42	3.23E-01	NS	1.00	6.06E-01	NS	0.46
Q5NKV6	2.52E-01	NS	-0.61	9.50E-01	NS	-0.03	7.48E-01	NS	-0.12	6.54E-01	NS	-0.17	9.94E-01	NS	0.02	1.87E-01	NS	-0.80	7.26E-01	NS	-0.12	5.23E-01	NS	-0.25	6.32E-01	NS	-0.18	1.33E-01	NS	-0.74	2.09E-01	NS	-0.54	6.52E-01	NS	-0.15	6.31E-01	NS	-0.18
F7GRY2	9.04E-01	NS	-0.02	9.38E-01	NS	-0.08	3.50E-01	NS	0.28	6.32E-01	NS	0.13	8.17E-01	NS	0.10	6.20E-01	NS	0.10	7.91E-01	NS	0.09	8.03E-01	NS	0.01	6.72E-01	NS	0.12	5.69E-01	NS	0.16	6.71E-01	NS	0.19	9.99E-01	NS	-0.04	9.26E-01	NS	-0.07
F6XAC5	4.00E-01	NS	-0.25	8.04E-01	NS	0.02	3.72E-01	NS	-0.31	3.20E-01	NS	-0.27	8.81E-01	NS	-0.05	5.75E-01	NS	-0.17	6.70E-01	NS	-0.13	3.34E-01	NS	-0.27	5.30E-01	NS	-0.19	2.40E-01	NS	-0.31	1.89E-01	NS	-0.43	5.69E-01	NS	-0.17	2.90E-01	NS	-0.34
F7HAH7	4.65E-01	NS	-0.45	9.43E-01	NS	-0.03	3.74E-01	NS	-0.53	6.18E-01	NS	-0.28	8.48E-01	NS	-0.10	6.35E-01	NS	-0.30	6.99E-01	NS	-0.24	6.93E-01	NS	-0.20	5.99E-01	NS	-0.30	2.48E-01	NS	-0.70	4.78E-01	NS	-0.42	5.33E-01	NS	-0.30	3.51E-01	NS	-0.55
A0A1D5QW71	3.72E-01	NS	-0.21	9.73E-01	NS	-0.01	7.36E-01	NS	-0.08	6.59E-01	NS	-0.11	9.63E-01	NS	0.01	8.43E-01	NS	-0.05	9.38E-01	NS	0.00	5.51E-01	NS	-0.16	3.21E-01	NS	-0.29	1.21E-01	NS	-0.49	1.78E-01	NS	-0.39	8.88E-01	NS	0.00	5.79E-01	NS	0.19
F7DHQ1	5.22E-01	NS	-0.38	8.99E-01	NS	0.04	9.62E-01	NS	1.52	7.62E-01	NS	-0.36	4.71E-01	NS	-0.24	2.80E-01	NS	0.93	8.08E-01	NS	1.77	4.53E-01	NS	1.66	6.06E-01	NS	0.91	6.61E-01	NS	0.26	4.10E-01	NS	0.49	6.20E-01	NS	-0.23	8.71E-01	NS	0.11
F6T0W5	1.51E-01	NS	-0.39	1.61E-01	NS	0.33	7.62E-01	NS	-0.06	9.26E-01	NS	0.14	7.91E-01	NS	0.17	2.79E-01	NS	0.21	5.71E-01	NS	0.24	8.71E-01	NS	0.10	4.16E-01	NS	0.48	5.92E-01	NS	0.28	8.23E-01	NS	0.09	8.74E-01	NS	0.09	8.98E-01	NS	0.02
F6PLV7	4.04E-01	NS	0.32	3.30E-01	NS	-1.55	6.40E-01	NS	0.54	8.27E-01	NS	0.02	3.76E-01	NS	0.27	7.37E-01	NS	0.02	4.50E-01	NS	0.17	6.58E-01	NS	0.52	8.10E-01	NS	-0.52	4.95E-01	NS	-1.05	8.94E-01	NS	0.03	4.03E-01	NS	0.55	9.19E-01	NS	0.36
F7HCV8	6.23E-01	NS	-0.17	6.28E-01	NS	-0.20	9.13E-01	NS	0.12	9.82E-01	NS	0.02	5.95E-01	NS	-0.20	9.52E-01	NS	0.04	4.58E-01	NS	0.30	7.35E-01	NS	0.16	4.96E-01	NS	0.21	7.15E-01	NS	-0.16	9.86E-01	NS	-0.03	4.79E-01	NS	-0.31	6.15E-01	NS	-0.21
F6SCP6	1.40E-01	NS	-0.41	8.71E-01	NS	0.02	7.10E-01	NS	0.29	7.29E-01	NS	0.25	1.43E-01	NS	-0.44	3.44E-01	NS	0.33	1.07E-01	NS	-0.39	8.16E-02	NS	-0.44	5.44E-01	NS	0.34	1.62E-01	NS	-0.35	6.08E-01	NS	0.29	1.02E-01	NS	-0.38	7.19E-01	NS	-0.06
F6S885	1.79E-01	NS	-0.40	6.77E-02	NS	-0.44	8.38E-01	NS	0.06	9.22E-01	NS	-0.05	3.41E-01	NS	-0.23	6.84E-01	NS	-0.11	3.22E-01	NS	-0.29	3.10E-01	NS	-0.28	3.07E-01	NS	-0.26	5.41E-01	NS	0.12	6.99E-01	NS	0.07	8.97E-01	NS	0.01	3.19E-01	NS	-0.31
H9H2V9	4.47E-01	NS	-0.42	7.31E-01	NS	-0.27	5.25E-01	NS	0.20	9.97E-01	NS	0.04	9.77E-01	NS	-0.03	9.55E-01	NS	-0.20	8.06E-01	NS	0.26	9.39E-01	NS	-0.06	7.62E-01	NS	-0.28	7.62E-01	NS	-0.18	6.59E-01	NS	-0.30	4.06E-01	NS	-0.58	3.63E-01	NS	-0.65
G7MVP0	5.15E-01	NS	1.41	2.34E-01	NS	-0.75	7.59E-01	NS	0.64	5.22E-01	NS	-0.35	8.46E-01	NS	-0.14	3.39E-01	NS	-0.50	5.82E-01	NS	-0.36	5.24E-01	NS	-0.23	7.77E-01	NS	0.06	1.31E-01	NS	-1.05	5.23E-01	NS	1.24	9.47E-01	NS	0.40	8.18E-01	NS	-0.03
F7DX07	6.20E-01	NS	-0.48	6.66E-01	NS	0.37	5.46E-01	NS	0.32	2.40E-01	NS	-0.80	9.75E-01	NS	0.03	6.94E-01	NS	-0.32	5.54E-01	NS	-0.45	4.27E-01	NS	-0.35	7.24E-01	NS	0.32	7.80E-01	NS	-0.06	9.51E-01	NS	-0.22	6.25E-01	NS	0.20	9.47E-01	NS	-0.08
F6SEN6	2.29E-01	NS	-0.99	1.53E-01	NS	-1.16	9.55E-01	NS	-0.12	4.70E-01	NS	-0.46	6.19E-01	NS	-0.50	9.42E-01	NS	-0.02	7.45E-01	NS	-0.44	2.88E-01	NS	0.51	9.92E-01	NS	0.08	9.46E-01	NS	0.54	5.55E-01	NS	0.06	3.87E-01	NS	-0.40	2.24E-01	NS	-0.93
F6ZH99	3.75E-01	NS	0.51	6.61E-01	NS	0.15	6.86E-01	NS	0.29	5.61E-01	NS	0.07	7.30E-01	NS	-0.42	4.74E-01	NS	0.42	7.79E-01	NS	0.01	1.08E-01	NS	0.96	4.40E-01	NS	0.14	2.01E-01	NS	0.83	9.87E-01	NS	-0.21	1.73E-01	NS	0.75	8.51E-01	NS	-0.17
A0A1D5QUU6	7.39E-01	NS	-0.14	2.68E-01	NS	-0.44	6.59E-01	NS	0.19	9.68E-01	NS	-0.04	9.32E-01	NS	-0.02	8.57E-01	NS	0.08	7.62E-01	NS	-0.15	9.18E-01	NS	-0.03	5.22E-01	NS	-0.18	7.11E-01	NS	0.05	5.72E-01	NS	0.15	8.81E-01	NS	0.06	9.34E-01	NS	0.08
F7BCK9	7.67E-01	NS	-0.15	7.21E-01	NS	0.16	6.75E-01	NS	-0.28	8.45E-01	NS	-0.06	8.73E-01	NS	0.15	9.31E-01	NS	-0.11	9.53E-01	NS	0.05	8.05E-01	NS	-0.09	7.60E-01	NS	-0.19	3.81E-01	NS	-0.58	6.83E-01	NS	-0.27	6.95E-01	NS	-0.19	7.04E-01	NS	-0.31
F6PRP0	4.40E-01	NS	-0.60	7.16E-01	NS	-0.33	6.39E-01	NS	-0.42	6.27E-01	NS	-0.41	6.16E-01	NS	-0.43	8.72E-01	NS	-0.22	7.74E-01	NS	-0.25	9.83E-01	NS	-0.15	8.57E-01	NS	-0.13	4.14E-01	NS	-0.62	6.50E-01	NS	-0.36	6.79E-01	NS	-0.37	6.40E-01	NS	-0.41
F7FY55	7.42E-01	NS	0.03	4.85E-01	NS	0.19	9.54E-01	NS	0.04	7.69E-01	NS	0.12	5.45E-01	NS	0.35	6.16E-01	NS	0.16	6.47E-01	NS	0.21	7.73E-01	NS	0.14	9.77E-01	NS	-0.04	8.85E-01	NS	-0.08	8.82E-01	NS	-0.14	7.91E-01	NS	-0.16	7.70E-01	NS	-0.21
F6SG43	3.31E-01	NS	-0.32	6.16E-01	NS	-0.16	7.17E-01	NS	-0.11	4.90E-01	NS	-0.22	7.97E-01	NS	0.04	8.17E-01	NS	0.06	5.36E-01	NS	-0.19	9.05E-01	NS	0.06	3.88E-01	NS	-0.30	1.43E-01	NS	-0.51	3.04E-01	NS	-0.36	3.94E-01	NS	-0.27	2.02E-01	NS	-0.38
F7FHW4	2.52E-01	NS	0.42	4.99E-01	NS	0.30	8.21E-01	NS	-0.12	4.60E-01	NS	0.31	8.40E-01	NS	0.23	7.02E-01	NS	0.10	5.02E-01	NS	0.31	8.52E-01	NS	-0.06	8.68E-01	NS	0.17	6.30E-02	NS	0.91	9.74E-01	NS	-0.08	2.23E-01	NS	0.71	2.35E-01	NS	0.45
F7DXU0	8.47E-01	NS	0.28	7.28E-01	NS	0.69	5.09E-01	NS	-0.39	4.45E-01	NS	-0.59	1.67E-01	NS	-1.44	3.77E-01	NS	-0.31	1.01E-01	NS	-1.86	4.74E-01	NS	0.72	3.86E-03	NS	-3.97	2.01E-01	NS	-0.52	7.86E-01	NS	1.09	6.62E-01	NS	0.59	5.99E-01	NS	0.02
A0N064	9.88E-01	NS	0.07	5.22E-01	NS	0.08	4.90E-01	NS	-0.15	6.38E-01	NS	-0.10	4.92E-01	NS	0.24	3.54E-01	NS	-0.20	9.73E-01	NS	0.05	6.57E-01	NS	-0.08	8.07E-01	NS	-0.02	4.44E-01	NS	-0.18	1.84E-01	NS	-0.31	7.37E-01	NS	-0.06	1.26E-01	NS	-0.27
F6SB17	9.14E-01	NS	-0.04	7.18E-01	NS	0.10	9.10E-01	NS	0.07	9.91E-01	NS	-0.03	7.16E-01	NS	0.07	5.37E-01	NS	-0.22	9.63E-01	NS	0.00	5.61E-01	NS	-0.19	6.31E-01	NS	-0.16	9.04E-01	NS	0.06	6.81E-01	NS	-0.14	7.74E-01	NS	0.05	8.83E-01	NS	-0.07
I2CYL7	3.08E-01	NS	-0.27	6.39E-01	NS	-0.08	9.49E-02	NS	0.75	6.67E-01	NS	0.12	8.48E-01	NS	-0.03	6.20E-02	NS	0.55	7.74E-01	NS	-0.03	7.06E-01	NS	0.30	4.02E-01	NS	0.35	2.73E-01	NS	0.22	2.30E-01	NS	0.36	8.35E-01	NS	-0.03	7.78E-01	NS	0.07
F7DH19	8.66E-01	NS	-0.16	8.97E-01	NS	0.00	8.47E-01	NS	-0.20	9.00E-01	NS	-0.11	8.79E-01	NS	-0.08	9.54E-01	NS	-0.02	8.16E-01	NS	-0.19	4.24E-01	NS	-0.52	8.50E-01	NS	-0.13	6.90E-01	NS	-0.30	5.78E-01	NS	-0.38	9.27E-01	NS	-0.09	6.17E-01	NS	-0.37
F6U5Q9	4.83E-01	NS	0.83	5.07E-01	NS	-0.40	6.85E-01	NS	0.18	6.28E-01	NS	0.26	7.44E-01	NS	0.22	5.57E-01	NS	0.19	8.29E-01	NS	-0.20	6.05E-01	NS	-0.30	8.69E-01	NS	-0.05	9.99E-01	NS	-0.12	8.80E-01	NS	-0.06	7.77E-01	NS	-0.14	9.93E-01	NS	-0.02
F6W3M2	9.79E-01	NS	-0.03	5.50E-01	NS	-0.25	4.37E-01	NS	0.25	7.68E-01	NS	-0.15	7.31E-01	NS	-0.17	3.54E-01	NS	0.60	9.15E-01	NS	0.07	2.39E-01	NS	0.54	8.22E-01	NS	-0.11	8.40E-01	NS	-0.01	4.55E-01	NS	0.34	4.48E-01	NS	-0.33	2.43E-01	NS	-0.47
F7HM21	9.80E-01	NS	0.03	5.19E-03	NS	-0.54	9.66E-01	NS	0.02	2.97E-01	NS	-0.28	5.65E-01	NS	-0.08	3.52E-01	NS	-0.21	7.81E-01	NS	0.04	1.10E-01	NS	-0.33	5.86E-02	NS	-0.35	5.93E-01	NS	-0.06	1.44E-01	NS	-0.29	9.89E-02	NS	-0.13	7.50E-03	NS	-0.33
F7HFD0	5.31E-01	NS	0.83	7.01E-01	NS	1.11	5.80E-01	NS	0.66	8.15E-01	NS	0.75	8.11E-01	NS	0.39	6.42E-01	NS	0.51	5.93E-01	NS	0.69	8.23E-01	NS	0.31	7.55E-01	NS	0.49	7.75E-01	NS	0.67	8.57E-01	NS	0.28	5.96E-01	NS	0.60	7.56E-01	NS	0.61
F7H263	6.48E-01	NS	0.24	3.36E-01	NS	0.33	8.20E-01	NS	0.13	7.86E-01	NS	0.08	8.34E-01	NS	0.21	6.91E-01	NS	-0.16	7.13E-01	NS	0.34	1.29E-01	NS	-0.64	5.78E-01	NS	-0.21	7.10E-01	NS	0.15	7.56E-01	NS	-0.13	5.12E-01	NS	0.29	7.38E-01	NS	0.18
F7AV81	6.92E-01	NS	1.28	1.79E-01	NS	-0.60	1.94E-01	NS	-0.35	3.42E-01	NS	1.45	8.19E-01	NS	0.33	1.02E-01	NS	2.39	3.29E-01	NS	1.56	1.25E-01	NS	2.47	5.06E-01	NS	1.49	2.83E-01	NS	0.58	1.50E-01	NS	-0.37	8.75E-02	NS	-0.86	4.62E-02	NS	-0.62
F7EVX1	7.42E-01	NS	0.23	6.30E-01	NS	-0.28	6.01E-01	NS	0.55	7.68E-01	NS	0.18	5.45E-01	NS	-0.43	7.58E-01	NS	0.63	6.06E-01	NS	-0.28	8.74E-01	NS	0.20	9.92E-01	NS	-0.05	8.06E-01	NS	0.29	8.50E-01	NS	0.34	9.70E-01	NS	0.37	7.30E-01	NS	0.73
H9YUD6	7.34E-01	NS	-0.20	6.50E-01	NS	0.10	5.60E-01	NS	-0.08	6.09E-01	NS	-0.17	9.65E-01	NS	0.06	7.55E-01	NS	0.05	8.66E-01	NS	0.08	9.64E-01	NS	-0.01	5.73E-01	NS	-0.17	3.18E-01	NS	-0.48	3.29E-01	NS	-0.53	4.96E-01	NS	-0.26	7.52E-01	NS	-0.17
F6W003	7.82E-01	NS	-0.15	7.27E-01	NS	-0.17	6.20E-01	NS	0.47	7.83E-01	NS	-0.15	5.26E-01	NS	-0.30	5.83E-01	NS	0.21	6.61E-01	NS	-0.18	8.70E-01	NS	0.01	4.14E-01	NS	-0.37	2.19E-01	NS	-0.55	4.57E-01	NS	-0.35	3.55E-01	NS	-0.43	5.11E-01	NS	-0.31
F7BQW1	7.24E-01	NS	0.60	9.79E-01	NS	-0.12	5.59E-01	NS	0.22	8.33E-01	NS	-0.10	9.72E-01	NS	-0.22	7.97E-01	NS	0.31	9.40E-01	NS	-0.26	6.05E-01	NS	0.62	9.84E-01	NS	0.34	8.00E-01	NS	0.16	5.60E-01	NS	0.27	7.60E-01	NS	-0.17	6.99E-01	NS	0.09
F6YWS9	3.58E-01	NS	-0.21	5.37E-01	NS	-0.15	7.39E-01	NS	0.10	3.17E-01	NS	-0.26	6.29E-01	NS	-0.12	9.57E-01	NS	-0.02	3.23E-01	NS	-0.28	7.31E-01	NS	0.04	9.45E-01	NS	-0.02	2.51E-01	NS	-0.31	3.63E-01	NS	-0.23	4.49E-01	NS	-0.21	6.59E-01	NS	-0.11
A0A1D5R122	8.41E-01	NS	0.15	5.19E-01	NS	0.31	7.92E-01	NS	0.12	7.90E-01	NS	0.14	8.43E-01	NS	0.26	5.70E-01	NS	-0.60	6.37E-01	NS	0.72	7.13E-01	NS	-0.41	9.34E-01	NS	0.17	6.27E-01	NS	0.20	7.64E-01	NS	-0.43	6.89E-01	NS	0.27	9.48E-01	NS	0.07
F7HG52	9.70E-01	NS	-0.02	7.61E-01	NS	0.06	4.44E-01	NS	0.15	4.77E-01	NS	0.15	9.77E-01	NS	-0.03	4.12E-01	NS	0.18	5.35E-01	NS	0.10	7.70E-01	NS	-0.11	9.06E-01	NS	0.02	7.83E-01	NS	0.04	8.31E-01	NS	0.01	9.37E-01	NS	-0.05	7.62E-01	NS	0.05
F6WYB6	9.48E-01	NS	-0.07	8.41E-01	NS	0.32	9.74E-01	NS	0.29	9.77E-01	NS	0.25	9.70E-01	NS	0.17	8.35E-01	NS	0.25	9.82E-01	NS	0.17	9.10E-01	NS	0.19	9.85E-01	NS	0.09	9.76E-01	NS	0.23	9.77E-01	NS	0.06	9.12E-01	NS	-0.34	9.86E-01	NS	-0.18
F6VZ47	9.94E-01	NS	-0.07	4.73E-01	NS	0.21	5.20E-01	NS	-0.38	9.95E-01	NS	-0.03	7.60E-01	NS	0.09	9.83E-01	NS	-0.03	9.79E-01	NS	-0.03	8.37E-01	NS	-0.10	6.57E-01	NS	-0.29	9.13E-01	NS	0.02	5.13E-01	NS	-0.35	6.07E-01	NS	0.22	9.28E-01	NS	-0.07
G7NQC3	5.57E-01	NS	0.92	5.60E-02	NS	-1.08	8.36E-02	NS	-0.54	8.45E-01	NS	1.05	9.90E-01	NS	0.38	1.69E-01	NS	1.55	2.96E-01	NS	1.18	3.12E-01	NS	2.19	9.73E-01	NS	1.23	6.29E-01	NS	0.28	5.10E-02	NS	-0.60	1.10E-01	NS	-0.86	8.20E-03	NS	-0.96
F7DAC5	6.07E-01	NS	-0.18	6.59E-01	NS	-0.16	4.95E-01	NS	-0.24	7.31E-01	NS	0.11	8.08E-01	NS	0.08	5.91E-01	NS	0.19	5.97E-01	NS	-0.17	1.37E-01	NS	0.49	8.41E-01	NS	-0.10	3.67E-01	NS	-0.33	7.80E-01	NS	-0.09	2.74E-01	NS	-0.38	2.87E-01	NS	-0.37
F6QJD8	9.74E-01	NS	0.12	8.49E-01	NS	0.37	7.56E-01	NS	-0.04	4.22E-01	NS	0.62	7.31E-02	NS	1.32	1.63E-02	NS	2.05	1.75E-01	NS	1.36	1.37E-01	NS	1.27	3.52E-01	NS	0.81	7.30E-01	NS	0.10	7.60E-01	NS	0.01	9.28E-01	NS	-0.01	7.34E-01	NS	0.09
F7C9A4	5.85E-01	NS	-0.10	6.40E-01	NS	-0.09	9.26E-01	NS	0.08	5.39E-01	NS	-0.12	5.89E-01	NS	-0.10	7.68E-01	NS	-0.03	6.73E-01	NS	0.15	5.15E-01	NS	-0.14	3.68E-01	NS	-0.20	5.63E-01	NS	-0.12	8.83E-01	NS	-0.01	4.01E-01	NS	-0.20	6.65E-01	NS	-0.07
F6X848	8.61E-01	NS	0.10	8.11E-01	NS	0.12	6.58E-01	NS	0.48	6.88E-01	NS	0.12	7.75E-01	NS	0.06	9.09E-01	NS	-0.04	9.48E-01	NS	0.00	3.35E-01	NS	0.27	8.58E-01	NS	-0.15	9.92E-01	NS	0.07	9.59E-01	NS	-0.02	7.86E-01	NS	0.22	3.60E-01	NS	0.44
F6UZ60	9.63E-01	NS	-0.29	4.66E-01	NS	0.29	9.73E-01	NS	-0.80	7.38E-01	NS	-1.02	1.64E-01	NS	-2.53	4.06E-01	NS	-1.72	3.81E-01	NS	-1.87	6.88E-01	NS	0.15	9.32E-01	NS	-0.28	5.40E-01	NS	0.63	7.62E-01	NS	0.41	9.95E-01	NS	-0.40	7.17E-01	NS	0.91
A0A1D5Q4R2	6.96E-01	NS	0.28	2.36E-01	NS	0.60	4.79E-01	NS	0.17	4.99E-01	NS	0.19	5.75E-01	NS	0.11	4.86E-01	NS	0.34	2.18E-01	NS	-0.54	7.69E-01	NS	-0.14	7.02E-01	NS	-0.21	4.39E-01	NS	0.20	8.83E-01	NS	-0.13	3.06E-01	NS	0.73	3.77E-01	NS	0.42

Supplementary table 6

name	0.25 h/Pre p-value	FDR	log2(FC)	0.5 h/Pre p-value	FDR	log2(FC)	1 h/Pre p-value	FDR	log2(FC)	2 h/Pre p-value	FDR	log2(FC)	4 h/Pre p-value	FDR	log2(FC)	8 h/Pre p-value	FDR	log2(FC)	16 h/Pre p-value	FDR	log2(FC)
X6RJP6	3.43E-03	NS	↑ 3.24	2.86E-05	NS	↑ 3.58	4.84E-06	NS	↑ 3.57	8.81E-03	NS	↑ 2.52	2.41E-02	NS	↑ 1.29	1.73E-01	NS	↑ 0.97	6.90E-01	NS	↑ 0.52
I3L3D5	6.24E-04	NS	↑ 2.91	3.04E-03	NS	↑ 2.93	3.97E-05	NS	↑ 2.97	7.11E-04	NS	↑ 2.57	4.43E-03	NS	↑ 1.03	1.58E-02	NS	↑ 1.05	6.25E-01	NS	↑ 0.52
Q53GK6	1.72E-04	NS	↑ 2.48	2.21E-03	NS	↑ 2.98	2.92E-04	NS	↑ 3.17	1.22E-03	NS	↑ 1.77	1.77E-02	NS	↑ 0.81	5.14E-04	NS	↑ 1.43	2.01E-01	NS	↑ 0.88
B4E3P1	2.98E-04	NS	↑ 2.90	1.67E-04	NS	↑ 2.57	2.26E-03	NS	↑ 2.32	4.67E-03	NS	↑ 2.58	6.80E-03	NS	↑ 0.94	2.11E-02	NS	↑ 0.90	1.22E-01	NS	↑ 0.65
F2RM35	5.49E-02	NS	↓ -0.40	7.15E-02	NS	↓ -0.36	1.62E-02	NS	↓ -0.46	3.52E-01	NS	↓ -0.16	2.87E-01	NS	↓ -0.17	7.49E-02	NS	↓ -0.37	8.54E-01	NS	↑ 0.09
B3KT06	8.46E-05	NS	↑ 2.69	1.28E-04	NS	↑ 3.86	4.77E-04	NS	↑ 3.12	5.95E-03	NS	↑ 2.80	2.34E-01	NS	↑ 0.65	1.02E-01	NS	↑ 2.25	1.41E-01	NS	↑ 2.02
A5PL27	3.27E-02	NS	↑ 0.73	5.68E-02	NS	↑ 0.79	1.11E-03	NS	↑ 1.17	7.81E-01	NS	↑ 0.35	2.87E-01	NS	↑ 0.73	1.52E-01	NS	↑ 0.75	9.22E-02	NS	↑ 0.67
D0PNI1	3.00E-01	NS	↑ 1.58	2.60E-03	NS	↑ 1.89	3.89E-02	NS	↑ 1.11	6.29E-01	NS	↑ 1.22	3.97E-01	NS	↑ 0.52	1.08E-01	NS	↑ 0.52	4.95E-01	NS	↓ -0.37
F8W876	1.73E-01	NS	↑ 0.43	4.55E-02	NS	↑ 0.42	7.86E-03	NS	↑ 0.73	3.28E-01	NS	↑ 0.24	3.24E-02	NS	↑ 0.38	1.64E-01	NS	↑ 0.27	7.19E-02	NS	↑ 0.38
Q53H26	5.93E-02	NS	↓ -0.74	7.68E-02	NS	↓ -0.71	5.96E-02	NS	↓ -0.76	1.26E-01	NS	↓ -0.53	5.12E-01	NS	↑ 0.04	1.91E-01	NS	↓ -0.51	1.98E-01	NS	↓ -0.52
V9HWE9	1.20E-02	NS	↑ 1.21	9.29E-03	NS	↑ 1.45	2.66E-02	NS	↑ 1.98	4.13E-01	NS	↑ 0.76	1.40E-02	NS	↑ 0.99	2.10E-01	NS	↑ 1.29	6.95E-01	NS	↑ 0.37
B4DDT3	8.62E-01	NS	↓ -0.01	6.26E-01	NS	↓ -0.07	1.06E-01	NS	↑ 0.72	7.70E-01	NS	↓ -0.05	2.39E-01	NS	↓ -0.26	1.47E-01	NS	↑ 0.72	9.11E-01	NS	↑ 0.05
B7Z555	8.44E-01	NS	↑ 0.06	8.41E-01	NS	↑ 0.08	2.17E-02	NS	↑ 0.54	9.40E-01	NS	↑ 0.06	8.39E-01	NS	↓ -0.03	9.99E-02	NS	↑ 0.26	5.28E-01	NS	↑ 0.20
F8W0J6	2.58E-01	NS	↑ 0.49	4.43E-01	NS	↑ 0.62	2.44E-01	NS	↑ 0.54	9.44E-02	NS	↑ 0.91	2.94E-01	NS	↑ 0.83	4.42E-01	NS	↑ 0.67	2.33E-01	NS	↑ 0.81
B1AP58	3.08E-01	NS	↑ 0.25	7.01E-01	NS	↑ 0.18	3.67E-02	NS	↑ 0.47	8.29E-01	NS	↑ 0.13	9.08E-01	NS	↑ 0.02	1.05E-01	NS	↑ 0.45	2.26E-01	NS	↑ 0.39
Q53H37	1.02E-01	NS	↓ -0.68	4.56E-01	NS	↓ -0.30	3.26E-01	NS	↓ -0.53	2.38E-01	NS	↓ -0.11	3.25E-01	NS	↓ -0.38	8.16E-01	NS	↑ 0.56	3.59E-01	NS	↓ -0.25
V9HWN8	6.32E-01	NS	↑ 0.03	3.74E-01	NS	↑ 0.16	4.90E-02	NS	↑ 0.52	8.65E-01	NS	↓ -0.02	2.57E-01	NS	↑ 0.22	3.44E-01	NS	↑ 0.27	2.86E-01	NS	↑ 0.15
H3BRJ9	6.77E-01	NS	↓ -0.40	8.59E-01	NS	↑ 0.14	1.30E-01	NS	↑ 1.26	9.29E-01	NS	↓ -0.04	3.69E-01	NS	↑ 0.16	5.62E-01	NS	↑ 0.30	1.63E-01	NS	↑ 0.42
D6R934	8.87E-01	NS	↓ -0.04	8.02E-01	NS	↓ -0.09	7.98E-02	NS	↑ 0.46	2.45E-01	NS	↓ -0.36	8.80E-01	NS	↓ -0.05	5.14E-01	NS	↑ 0.14	8.11E-01	NS	↓ -0.03
B2RA39	2.30E-01	NS	↓ -1.51	3.50E-01	NS	↓ -1.29	6.63E-01	NS	↓ -0.76	9.78E-01	NS	↓ -0.21	2.00E-01	NS	↓ -1.58	3.68E-01	NS	↑ 0.07	6.77E-01	NS	↓ -0.95
L8E853	7.06E-01	NS	↑ 0.14	2.79E-01	NS	↑ 0.29	1.39E-01	NS	↑ 0.86	2.41E-01	NS	↑ 0.51	1.10E-01	NS	↑ 0.51	3.53E-01	NS	↑ 0.69	3.30E-01	NS	↑ 0.28
E3UN46	4.14E-01	NS	↓ -0.39	3.18E-01	NS	↓ -0.45	6.37E-01	NS	↓ -0.26	9.80E-01	NS	↓ -0.09	4.03E-01	NS	↓ -0.39	9.77E-01	NS	↓ -0.15	4.75E-01	NS	↓ -0.37
A0A182DWH7	1.78E-02	NS	↓ -0.41	3.49E-02	NS	↓ -0.45	1.09E-01	NS	↓ -0.46	8.65E-02	NS	↓ -0.33	9.60E-02	NS	↓ -0.31	8.90E-02	NS	↓ -0.35	3.92E-02	NS	↓ -0.44
B7Z549	7.82E-01	NS	↑ 0.04	7.44E-01	NS	↑ 0.09	3.19E-02	NS	↑ 0.45	4.84E-01	NS	↑ 0.25	6.26E-01	NS	↓ -0.09	1.62E-01	NS	↑ 0.37	9.91E-01	NS	↑ 0.05
A0A024R9Q1	7.04E-02	NS	↑ 0.52	4.52E-01	NS	↑ 0.32	2.67E-01	NS	↑ 0.43	4.96E-01	NS	↑ 0.35	9.78E-01	NS	↓ -0.07	6.47E-01	NS	↓ -0.12	3.01E-01	NS	↓ -0.38
A0A087WSY5	5.37E-01	NS	↑ 0.14	9.96E-01	NS	↑ 0.01	5.69E-01	NS	↑ 0.14	2.86E-01	NS	↑ 0.22	3.56E-01	NS	↑ 0.12	2.90E-01	NS	↑ 0.24	6.72E-01	NS	↑ 0.05
E7END6	7.19E-01	NS	↓ -0.05	6.89E-01	NS	↓ -0.05	8.71E-02	NS	↓ -0.30	6.32E-01	NS	↓ -0.08	9.23E-01	NS	↑ 0.01	1.26E-02	NS	↓ -0.40	1.99E-01	NS	↓ -0.22
B7ZKJ8	1.05E-01	NS	↑ 0.35	6.09E-02	NS	↑ 0.42	5.23E-02	NS	↑ 0.39	3.67E-01	NS	↑ 0.24	1.25E-01	NS	↑ 0.34	4.72E-01	NS	↑ 0.30	3.29E-01	NS	↑ 0.23
D9ZGG2	3.78E-01	NS	↓ -0.13	2.56E-01	NS	↓ -0.17	4.66E-02	NS	↓ -0.26	9.40E-01	NS	↓ -0.02	3.44E-01	NS	↓ -0.14	2.89E-02	NS	↓ -0.31	4.62E-01	NS	↓ -0.11
G3V2W1	9.76E-01	NS	↑ 0.05	8.61E-01	NS	↓ -0.07	2.06E-01	NS	↓ -0.35	2.67E-01	NS	↓ -0.22	8.66E-01	NS	↓ -0.07	2.19E-01	NS	↓ -0.30	3.34E-01	NS	↓ -0.22
J3QR68	3.58E-01	NS	↑ 0.02	3.60E-01	NS	↓ -0.28	1.88E-01	NS	↓ -0.76	2.66E-01	NS	↓ -0.58	3.41E-01	NS	↓ -0.66	1.07E-01	NS	↓ -0.68	9.39E-02	NS	↓ -0.81
V9HWE3	6.22E-01	NS	↑ 0.24	4.44E-01	NS	↑ 0.89	9.50E-01	NS	↑ 0.25	2.46E-01	NS	↑ 0.45	1.78E-02	NS	↑ 0.83	8.59E-02	NS	↑ 0.53	4.06E-01	NS	↑ 0.55
B4E1C2	3.34E-01	NS	↓ -0.17	1.03E-01	NS	↓ -0.30	1.68E-01	NS	↓ -0.25	3.91E-01	NS	↓ -0.14	8.14E-01	NS	↓ -0.01	2.76E-01	NS	↓ -0.19	1.78E-01	NS	↓ -0.25
A0A087WW43	5.44E-01	NS	↑ 0.14	3.96E-01	NS	↑ 0.12	5.64E-03	NS	↑ 0.51	3.58E-01	NS	↑ 0.19	8.26E-01	NS	↑ 0.10	1.40E-01	NS	↑ 0.30	4.82E-01	NS	↑ 0.30
A2RTY6	4.34E-01	NS	↑ 0.15	5.81E-01	NS	↑ 0.10	4.75E-02	NS	↑ 0.40	8.80E-01	NS	↑ 0.04	9.75E-01	NS	↓ -0.06	2.02E-01	NS	↑ 0.24	7.70E-01	NS	↑ 0.07
A0A0U1RR20	1.75E-01	NS	↓ -0.29	4.59E-01	NS	↓ -0.10	2.26E-01	NS	↓ -0.23	1.86E-01	NS	↓ -0.29	1.16E-01	NS	↓ -0.47	6.70E-01	NS	↓ -0.01	5.71E-01	NS	↑ 0.18
E9PHK0	4.02E-01	NS	↓ -0.16	5.03E-01	NS	↓ -0.14	3.15E-01	NS	↓ -0.21	5.23E-01	NS	↓ -0.13	4.50E-01	NS	↑ 0.27	7.55E-01	NS	↑ 0.01	9.23E-01	NS	↑ 0.08
D3DNN4	2.97E-01	NS	↑ 0.47	1.68E-01	NS	↑ 0.86	1.88E-02	NS	↑ 1.03	9.25E-01	NS	↑ 0.43	7.57E-01	NS	↓ -0.37	2.61E-01	NS	↑ 0.70	4.00E-01	NS	↑ 0.71
Q499G5	6.62E-01	NS	↑ 0.06	4.45E-01	NS	↑ 0.28	4.38E-01	NS	↓ -0.09	9.14E-01	NS	↑ 0.14	8.12E-01	NS	↑ 0.08	3.40E-01	NS	↑ 0.17	5.46E-01	NS	↓ -0.08
A0A0S2Z3D5	2.93E-01	NS	↑ 0.33	4.02E-01	NS	↑ 0.30	3.59E-01	NS	↑ 0.39	9.27E-01	NS	↑ 0.12	5.22E-01	NS	↓ -0.17	2.97E-01	NS	↑ 0.58	8.50E-01	NS	↓ -0.01
RT-Cal protein	7.33E-01	NS	↓ -0.03	8.10E-01	NS	↓ -0.02	3.28E-01	NS	↓ -0.08	3.17E-01	NS	↑ 0.08	6.98E-01	NS	↓ -0.03	3.79E-01	NS	↓ -0.07	7.05E-01	NS	↓ -0.03
B4DRT3	7.37E-01	NS	↑ 0.29	2.68E-02	NS	↑ 0.91	2.20E-01	NS	↑ 0.63	7.68E-02	NS	↑ 0.73	2.10E-02	NS	↑ 0.87	8.30E-06	NS	↑ 1.91	5.08E-03	NS	↑ 1.61
A8K1K1	7.30E-01	NS	↑ 0.05	6.43E-01	NS	↑ 0.32	2.54E-01	NS	↑ 0.42	8.52E-01	NS	↑ 0.03	7.89E-01	NS	↓ -0.37	3.72E-01	NS	↑ 0.35	9.00E-01	NS	↓ -0.18
A0A140VK00	5.84E-01	NS	↓ -0.10	4.24E-01	NS	↓ -0.14	2.42E-01	NS	↓ -0.25	1.80E-01	NS	↓ -0.29	2.50E-01	NS	↓ -0.23	3.51E-01	NS	↓ -0.19	5.07E-01	NS	↓ -0.08
A0A140VJI7	2.67E-01	NS	↓ -0.32	3.59E-01	NS	↓ -0.28	2.40E-01	NS	↓ -0.33	2.18E-01	NS	↓ -0.35	7.00E-01	NS	↑ 0.04	1.79E-01	NS	↓ -0.38	9.55E-01	NS	↑ 0.03
A0A024RAA7	3.68E-01	NS	↓ -0.23	3.95E-01	NS	↓ -0.22	8.53E-01	NS	↑ 0.05	3.13E-01	NS	↓ -0.25	7.27E-01	NS	↓ -0.10	8.08E-01	NS	↓ -0.05	2.52E-01	NS	↓ -0.28
B3KS79	9.84E-01	NS	↓ -0.02	7.31E-01	NS	↓ -0.07	1.53E-01	NS	↑ 0.27	7.85E-01	NS	↓ -0.05	9.74E-01	NS	↑ 0.03	1.01E-01	NS	↑ 0.27	1.96E-01	NS	↑ 0.34
Q8TAY0	7.06E-01	NS	↑ 0.13	6.39E-01	NS	↑ 0.10	1.46E-01	NS	↑ 0.35	9.83E-01	NS	↑ 0.03	7.18E-01	NS	↓ -0.16	4.75E-01	NS	↑ 0.13	9.05E-01	NS	↓ -0.10
Q9H2E1	7.54E-01	NS	↑ 0.83	3.05E-01	NS	↑ 0.27	9.47E-01	NS	↓ -0.65	2.97E-01	NS	↑ 0.11	6.98E-01	NS	↑ 0.94	6.09E-01	NS	↓ -0.09	8.11E-01	NS	↓ -0.20
V9HWA9	4.38E-01	NS	↑ 0.16	5.97E-01	NS	↑ 0.10	1.56E-01	NS	↑ 0.34	8.01E-01	NS	↑ 0.08	6.55E-01	NS	↓ -0.15	1.15E-01	NS	↑ 0.29	9.19E-01	NS	↑ 0.02
F5GXS0	5.18E-01	NS	↑ 0.11	5.93E-01	NS	↑ 0.07	1.52E-01	NS	↑ 0.31	9.60E-01	NS	↑ 0.03	8.27E-01	NS	↓ -0.01	2.39E-01	NS	↑ 0.21	7.63E-01	NS	↑ 0.06
Q59GS8	9.15E-01	NS	↑ 0.01	6.44E-01	NS	↑ 0.04	2.90E-01	NS	↑ 0.19	9.80E-01	NS	↓ -0.03	9.99E-01	NS	↑ 0.04	4.88E-01	NS	↑ 0.12	7.97E-01	NS	↓ -0.03
A0A3G1HEF1	9.44E-02	NS	↑ 0.46	2.42E-01	NS	↑ 0.24	8.85E-02	NS	↑ 0.48	5.61E-01	NS	↑ 0.11	6.18E-01	NS	↑ 0.09	4.43E-02	NS	↑ 0.56	4.67E-01	NS	↑ 0.15
A0A3B3IS66	7.25E-01	NS	↓ -0.07	7.17E-01	NS	↑ 0.02	5.40E-01	NS	↓ -0.16	9.59E-01	NS	↓ 0.00	7.75E-01	NS	↑ 0.04	2.19E-01	NS	↓ -0.29	2.30E-01	NS	↓ -0.30
A0A024R3E3	5.11E-01	NS	↓ -0.66	1.18E-01	NS	↓ -0.61	9.58E-02	NS	↓ -1.35	6.93E-02	NS	↓ -1.66	3.12E-01	NS	↑ 0.03	1.59E-01	NS	↓ -1.25	1.15E-01	NS	↓ -1.28
Q5VY30	5.74E-01	NS	↑ 0.35	3.98E-01	NS	↑ 0.20	3.20E-01	NS	↓ -0.27	8.25E-01	NS	↑ 0.06	6.72E-01	NS	↑ 0.22	1.50E-01	NS	↓ -0.39	6.97E-01	NS	↓ -0.05
E9PIT3	7.93E-01	NS	↑ 0.17	9.48E-01	NS	↓ -0.08	1.10E-01	NS	↓ -0.92	6.45E-01	NS	↑ 0.38	4.84E-01	NS	↑ 0.43	2.23E-01	NS	↓ -0.75	4.17E-01	NS	↓ -0.40
A6XND1	6.41E-01	NS	↓ -0.13	5.69E-01	NS	↓ -0.15	1.31E-01	NS	↓ -0.38	8.49E-01	NS	↓ -0.06	9.25E-01	NS	↓ -0.03	6.44E-02	NS	↓ -0.48	3.19E-01	NS	↓ -0.25
A0A2S0BDD1	8.46E-01	NS	↑ 0.04	9.56E-01	NS	↑ 0.03	4.16E-01	NS	↑ 0.16	7.66E-01	NS	↓ -0.12	4.61E-01	NS	↑ 0.08	2.06E-01	NS	↑ 0.30	1.40E-01	NS	↑ 0.36
V9HVY1	1.24E-01	NS	↑ 0.87	3.48E-01	NS	↑ 0.78	2.27E-01	NS	↑ 0.51	1.02E-02	NS	↑ 1.28	6.90E-02	NS	↑ 0.87	5.61E-01	NS	↑ 0.56	1.30E-01	NS	↑ 0.59
A0A2Z4LCH4	7.16E-01	NS	↑ 0.06	6.48E-01	NS	↑ 0.13	3.02E-01	NS	↑ 0.28	9.52E-01	NS	↓ -0.14	6.91E-01	NS	↓ -0.03	5.85E-01	NS	↑ 0.07	6.34E-01	NS	↑ 0.04
J3K000	9.38E-01	NS	↑ 0.08	8.78E-01	NS	↑ 0.05	4.77E-01	NS	↑ 0.26	5.22E-01	NS	↑ 0.22	8.98E-01	NS	↓ -0.08	2.45E-01	NS	↑ 0.35	5.67E-01	NS	↑ 0.13
B4DVE1	9.32E-01	NS	↓ -0.01	5.02E-01	NS	↑ 0.15	1.79E-01	NS	↑ 0.78	4.48E-01	NS	↑ 0.14	6.34E-01	NS	↑ 0.18	2.01E-01	NS	↑ 1.00	6.30E-01	NS	↑ 0.22
D3DPG0	6.95E-01	NS	↓ -0.18	8.61E-01	NS	↓ -0.03	1.72E-01	NS	↑ 0.41	6.72E-01	NS	↓ -0.19	5.45E-01	NS	↓ -0.30	3.50E-01	NS	↑ 0.16	9.30E-01	NS	↓ -0.03
D9IWP9	9.21E-01	NS	↓ -0.08	6.03E-01	NS	↓ -0.20	1.03E-01	NS	↓ -0.55	7.33E-01	NS	↓ -0.16	9.44E-01	NS	↓ -0.02	1.52E-01	NS	↓ -0.46	7.91E-01	NS	↓ 0.00
M0R2W8	8.61E-01	NS	↓ -0.04	3.81E-01	NS	↓ -0.53	9.63E-01	NS	↑ 0.01	3.49E-01	NS	↓ -0.49	5.02E-01	NS	↓ -0.45	4.61E-01	NS	↑ 0.17	9.36E-01	NS	↓ -0.01
Q59E93	6.23E-01	NS	↓ -0.06	3.96E-01	NS	↓ -0.39	3.92E-01	NS	↑ 0.82	4.33E-01	NS	↑ 0.08	5.55E-01	NS	↑ 0.17	1.44E-01	NS	↑ 0.81	6.94E-01	NS	↑ 0.34
B2R815	9.13E-01	NS	↑ 0.01	9.92E-01	NS	↓ -0.03	1.76E-01	NS	↑ 0.29	7.80E-01	NS	↓ -0.11	5.09E-01	NS	↓ -0.24	4.19E-01	NS	↑ 0.17	8.48E-01	NS	↓ -0.09
B2R4M6	3.75E-01	NS	↑ 0.57	4.47E-01	NS	↑ 0.27	1.75E-01	NS	↑ 0.47	3.86E-02	NS	↑ 1.13	2.92E-03	NS	↑ 1.82	2.04E-04	NS	↑ 2.38	9.26E-03	NS	↑ 1.74
V9GYM3	9.66E-01	NS	↓ -0.80	7.38E-01	NS	↓ -0.49	5.15E-01	NS	↓ -1.77	5.59E-01	NS	↓ -1.81	9.09E-01	NS	↓ -0.35	9.09E-01	NS	↓ -0.08	6.76E-01	NS	↓ -1.69
B4DPQ0	9.08E-01	NS	↓ -0.02	6.32E-01	NS	↑ 0.03	1.71E-01	NS	↑ 0.24	5.93E-01	NS	↑ 0.06	6.59E-01	NS	↓ -0.15	2.06E-01	NS	↑ 0.20	5.02E-01	NS	↑ 0.13
Q5SQ08	8.27E-01	NS	↑ 0.02	5.35E-01	NS	↑ 0.12	1.01E-01	NS	↑ 0.30	2.62E-01	NS	↑ 0.21	5.52E-01	NS	↓ -0.15	3.86E-01	NS	↑ 0.13	8.48E-01	NS	↑ 0.08
B4DRY0	5.69E-01	NS	↑ 0.90	1.77E-01	NS	↑ 2.95	4.71E-01	NS	↑ 0.27	4.31E-01	NS	↑ 0.69	2.81E-01	NS	↑ 0.51	5.31E-02	NS	↑ 1.33	2.15E-01	NS	↑ 0.43
S4R3Y4	1.08E-01	NS	↑ 0.32	2.38E-01	NS	↑ 0.24	3.31E-01	NS	↓ -0.16	8.00E-01	NS	↑ 0.02	2.59E-01	NS	↑ 0.18	7.89E-01	NS	↓ -0.08	6.94E-01	NS	↑ 0.12
A0A0S2Z4I5	8.56E-01	NS	↑ 0.03	5.43E-01	NS	↓ -0.12	8.15E-01	NS	↑ 0.02	1.47E-01	NS	↓ -0.32	1.25E-01	NS	↑ 0.30	4.68E-01	NS	↑ 0.24	5.19E-01	NS	↑ 0.10
A0A0G2JPR0	6.88E-01	NS	↑ 0.05	4.35E-01	NS	↑ 0.15	4.36E-01	NS	↑ 0.08	8.43E-01	NS	↓ -0.05	9.13E-01	NS	↓ -0.05	3.57E-01	NS	↑ 0.19	9.27E-01	NS	↓ -0.01
H0YID6	8.70E-02	NS	↑ 1.38	5.69E-01	NS	↑ 0.61	9.31E-02	NS	↑ 1.97	8.88E-01	NS	↑ 0.55	6.93E-01	NS	↑ 0.93	5.78E-02	NS	↑ 1.37	2.38E-01	NS	↑ 1.06
V9HWD8	8.20E-01	NS	↓ -0.07	6.79E-01	NS	↓ -0.15	9.84E-02	NS	↓ -0.55	8.60E-01	NS	↑ 0.01	9.97E-01	NS	↑ 0.08	9.08E-02	NS	↓ -0.55	4.95E-01	NS	↓ -0.20
H6VRG1	9.60E-01	NS	↑ 0.04	7.49E-01	NS	↑ 0.13	4.99E-01	NS	↑ 0.23	6.58E-01	NS	↓ -0.09	4.23E-01	NS	↑ 0.52	2.40E-01	NS	↑ 0.59	5.03E-01	NS	↑ 0.22
P78492	2.21E-01	NS	↑ 0.18	5.52E-01	NS	↑ 0.11	3.67E-01	NS	↑ 0.15	1.60E-01	NS	↑ 0.22	1.52E-01	NS	↑ 0.21	3.42E-01	NS	↑ 0.14	2.94E-01	NS	↑ 0.15
D3JV41	7.44E-01	NS	↑ 0.00	6.29E-01	NS	↓ -0.08	6.08E-01	NS	↑ 0.15	9.23E-01	NS	↑ 0.01	1.11E-01	NS	↓ -0.35	6.72E-01	NS	↑ 0.03	2.26E-01	NS	↓ -0.27
Q59EP2	5.68E-01	NS	↑ 0.11	4.71E-01	NS	↑ 0.14	9.34E-02	NS	↑ 0.25	9.51E-01	NS	↑ 0.07	9.75E-01	NS	↑ 0.01	1.34E-02	NS	↑ 0.40	1.38E-01	NS	↑ 0.41
C0JYY2	6.77E-01	NS	↓ -0.08	6.13E-01	NS	↓ -0.12	3.97E-01	NS	↑ 0.20	4.59E-01	NS	↓ -0.16	6.26E-01	NS	↓ -0.11	1.93E-01	NS	↑ 0.20	7.09E-01	NS	↓ -0.06
A0A024R1X6	9.35E-01	NS	↑ 0.05	1.23E-01	NS	↑ 2.64	5.25E-01	NS	↑ 0.04	6.19E-01	NS	↑ 0.43	4.86E-01	NS	↑ 0.25	6.31E-01	NS	↑ 0.60	6.55E-01	NS	↑ 0.12
S4R471	3.87E-01	NS	↑ 0.29	9.43E-02	NS	↑ 0.38	4.19E-01	NS	↑ 0.31	8.64E-01	NS	↑ 0.05	7.27E-01	NS	↑ 0.07	4.50E-01	NS	↑ 0.17	6.97E-01	NS	↑ 0.14
A0A024R104	6.01E-01	NS	↑ 0.41	6.97E-01	NS	↑ 0.13	1.47E-01	NS	↓ -0.40	4.48E-01	NS	↑ 0.20	3.39E-01	NS	↓ -0.23	2.87E-01	NS	↓ -0.24	3.73E-01	NS	↓ -0.19
V9HW68	2.49E-01	NS	↓ -0.63	2.78E-01	NS	↓ -0.40	6.34E-01	NS	↓ -0.29	1.40E-01	NS	↓ -0.83	4.98E-01	NS	↑ 0.00	6.93E-01	NS	↓ -0.08	7.70E-01	NS	↓ -0.32
B7Z8Q5	7.92E-01	NS	↓ -0.17	5.48E-01	NS	↓ -0.09	9.24E-01	NS	↑ 0.06	7.52E-02	NS	↑ 0.57	2.75E-01	NS	↑ 0.49	9.12E-01	NS	↓ -0.09	5.51E-01	NS	↑ 0.16
A0A140VKF3	4.79E-01	NS	↓ -0.20	9.07E-01	NS	↓ -0.04	4.76E-01	NS	↑ 0.18	7.97E-01	NS	↓ -0.08	7.61E-01	NS	↓ -0.13	1.21E-01	NS	↑ 0.29	8.88E-01	NS	↑ 0.06
A0A0S2Z3E8	6.32E-01	NS	↓ -0.63	8.97E-01	NS	↑ 0.05	4.42E-01	NS	↓ -1.04	4.72E-01	NS	↓ -0.99	9.83E-01	NS	↓ -0.60	2.69E-01	NS	↓ -1.30	4.77E-01	NS	↓ -1.03
Q59EA3	5.45E-01	NS	↓ -0.54	8.80E-01	NS	↓ -0.15	4.39E-01	NS	↓ -0.03	8.36E-01	NS	↑ 0.27	9.89E-01	NS	↓ -0.05	7.70E-01	NS	↓ -0.20	8.91E-01	NS	↓ -0.02
K7ERE3	9.33E-01	NS	↓ -0.09	8.79E-01	NS	↑ 1.62	5.77E-01	NS	↑ 0.05	7.52E-01	NS	↓ -0.08	3.40E-01	NS	↑ 0.50	6.52E-01	NS	↑ 0.42	8.09E-01	NS	↑ 0.10
V9HWF6	7.88E-01	NS	↓ -0.42	8.39E-01	NS	↓ -0.46	3.53E-01	NS	↓ -0.18	4.31E-01	NS	↓ -0.39	6.91E-01	NS	↓ -1.21	5.29E-01	NS	↓ -0.47	9.80E-01	NS	↓ -0.47
B4DPH4	5.38E-01	NS	↑ 0.08	9.44E-01	NS	↑ 0.02	2.39E-01	NS	↑ 0.23	9.97E-02	NS	↑ 0.18	7.22E-01	NS	↓ -0.04	8.55E-01	NS	↑ 0.06	9.52E-01	NS	↑ 0.03
C9JV77	8.56E-01	NS	↓ -0.07	8.24E-01	NS	↑ 0.06	3.28E-01	NS	↓ -0.34	8.48E-01	NS	↓ -0.05	5.55E-01	NS	↑ 0.22	1.66E-01	NS	↓ -0.46	4.91E-01	NS	↓ -0.21
A0A384MEF1	9.53E-01	NS	↑ 0.00	9.26E-01	NS	↓ 0.00	2.92E-01	NS	↑ 0.29	6.16E-01	NS	↑ 0.17	8.00E-01	NS	↓ -0.01	1.10E-01	NS	↑ 0.41	6.84E-01	NS	↑ 0.08
F8VV57	6.30E-01	NS	↓ -0.15	1.71E-01	NS	↑ 1.70	9.42E-01	NS	↓ -0.02	4.70E-01	NS	↓ -0.26	6.28E-01	NS	↑ 0.05	8.67E-01	NS	↑ 0.00	4.12E-01	NS	↑ 0.15
A0A384N669	4.66E-01	NS	↓ -0.34	5.42E-01	NS	↓ -0.29	3.97E-01	NS	↓ -0.39	5.87E-01	NS	↓ -0.27	6.73E-01	NS	↓ -0.21	4.77E-01	NS	↓ -0.34	7.24E-01	NS	↓ -0.17
Q4LE79	9.81E-01	NS	↓ -0.01	6.12E-01	NS	↑ 0.88	6.51E-01	NS	↑ 0.09	7.82E-01	NS	↑ 0.19	3.64E-01	NS	↓ -0.34	9.15E-01	NS	↓ -0.09	3.65E-01	NS	↓ -0.54
Q6MZL2	6.80E-01	NS	↑ 0.08	9.03E-01	NS	↑ 0.04	4.58E-01	NS	↑ 0.53	2.94E-01	NS	↑ 0.17	8.02E-02	NS	↑ 0.20	8.44E-01	NS	↑ 0.18	4.46E-01	NS	↑ 0.11
Q5J875	8.13E-01	NS	↑ 0.03	7.38E-01	NS	↓ -0.09	7.47E-01	NS	↓ -0.13	6.88E-01	NS	↓ -0.11	7.82E-01	NS	↓ -0.05	6.04E-01	NS	↓ -0.18	7.30E-01	NS	↓ -0.06
Q96K68	2.51E-01	NS	↓ -0.45	3.73E-01	NS	↓ -0.32	9.14E-01	NS	↑ 0.30	3.38E-01	NS	↓ -0.38	5.27E-01	NS	↓ -0.20	3.16E-01	NS	↓ -0.34	7.22E-01	NS	↓ -0.10
A0A0A0MRJ7	6.91E-01	NS	↓ -0.05	3.94E-01	NS	↓ -0.14	2.04E-01	NS	↓ -0.25	6.36E-01	NS	↓ -0.06	8.61E-01	NS	↓ -0.01	2.95E-02	NS	↓ -0.39	4.35E-01	NS	↓ -0.11
Q68CK4	6.23E-02	NS	↓ -0.35	8.37E-02	NS	↓ -0.28	1.94E-01	NS	↓ -0.22	7.24E-02	NS	↓ -0.27	1.99E-01	NS	↓ -0.18	3.31E-01	NS	↓ -0.13	9.37E-01	NS	↑ 0.03
B7ZLE5	8.79E-01	NS	↓ -0.19	6.35E-01	NS	↓ -0.36	5.26E-01	NS	↑ 0.14	8.32E-01	NS	↓ -0.18	6.80E-01	NS	↓ -0.32	1.97E-01	NS	↑ 0.34	8.21E-01	NS	↓ -0.27
D6RAR4	7.36E-01	NS	↓ -0.13	8.12E-01	NS	↓ -0.11	5.78E-01	NS	↓ -0.22	9.28E-01	NS	↓ -0.03	9.63E-01	NS	↑ 0.03	5.20E-01	NS	↓ -0.24	5.48E-01	NS	↓ -0.24
B2R773	9.51E-01	NS	↑ 0.03	7.10E-01	NS	↑ 0.11	4.90E-01	NS	↑ 0.17	6.96E-01	NS	↑ 0.21	3.60E-01	NS	↓ -0.40	6.49E-01	NS	↑ 0.40	6.21E-01	NS	↑ 0.15
A8K8Z4	5.31E-01	NS	↓ -0.08	2.72E-01	NS	↓ -0.15	6.72E-01	NS	↓ -0.05	9.24E-01	NS	↓ -0.01	2.37E-01	NS	↓ -0.16	8.55E-01	NS	↓ -0.01	6.31E-01	NS	↓ -0.07
F5H1C6	8.35E-01	NS	↓ -0.43	3.90E-01	NS	↑ 0.12	4.33E-01	NS	↑ 0.72	9.97E-01	NS	↓ -0.30	8.29E-01	NS	↓ -0.24	3.91E-01	NS	↑ 0.06	5.55E-01	NS	↓ -0.35
A0A024R6N9	2.74E-01	NS	↓ -0.19	2.92E-02	NS	↓ -0.40	1.74E-01	NS	↓ -0.27	4.43E-02	NS	↓ -0.38	2.45E-01	NS	↓ -0.19	5.97E-01	NS	↓ -0.08	2.85E-01	NS	↓ -0.18
Q53F26	5.58E-01	NS	↑ 0.17	7.48E-01	NS	↑ 0.08	8.67E-01	NS	↑ 0.43	7.17E-01	NS	↑ 0.28	4.73E-03	NS	↑ 0.82	3.43E-01	NS	↑ 0.67	4.11E-01	NS	↑ 0.70
B3KNX0	2.99E-01	NS	↓ -0.30	7.78E-01	NS	↑ 0.15	8.79E-01	NS	↑ 0.24	9.44E-01	NS	↓ -0.07	6.44E-01	NS	↑ 0.20	4.37E-01	NS	↓ -0.17	9.72E-01	NS	↑ 0.07
J3KPA1	5.64E-01	NS	↑ 0.32	7.33E-01	NS	↓ -0.02	6.75E-01	NS	↓ -0.03	5.25E-01	NS	↓ -0.01	7.74E-01	NS	↑ 0.22	8.54E-01	NS	↓ -0.01	9.98E-01	NS	↑ 0.09
Q53GN8	5.73E-01	NS	↑ 0.22	5.83E-01	NS	↑ 0.17	6.60E-01	NS	↓ -0.11	8.87E-01	NS	↑ 0.21	2.85E-01	NS	↑ 0.38	2.80E-01	NS	↓ -0.32	5.72E-01	NS	↓ -0.16
A0A1K0GXZ1	2.80E-01	NS	↓ -0.37	9.55E-01	NS	↑ 0.62	9.37E-01	NS	↓ -0.22	7.58E-01	NS	↑ 0.53	2.74E-01	NS	↑ 1.16	6.10E-02	NS	↑ 1.17	8.41E-02	NS	↑ 1.41
V9HWP0	8.44E-01	NS	↑ 0.03	4.50E-01	NS	↑ 0.27	5.42E-01	NS	↑ 0.28	6.76E-01	NS	↓ -0.23	4.08E-01	NS	↓ -0.33	9.28E-01	NS	↓ -0.09	7.77E-01	NS	↓ -0.12
A0A3B3ISJ1	6.55E-01	NS	↑ 0.08	4.26E-01	NS	↑ 0.14	1.86E-01	NS	↑ 0.21	2.26E-01	NS	↑ 0.18	7.45E-01	NS	↓ -0.02	9.12E-01	NS	↑ 0.02	9.45E-01	NS	↑ 0.02
Q8IVC0	9.86E-01	NS	↑ 0.01	5.86E-01	NS	↓ -0.10	3.16E-01	NS	↑ 0.18	5.53E-01	NS	↓ -0.09	6.84E-01	NS	↓ -0.03	7.18E-01	NS	↑ 0.07	2.50E-01	NS	↓ -0.24
E7EQ64	9.79E-01	NS	↓ -0.04	6.09E-01	NS	↓ -0.17	6.10E-01	NS	↓ -0.08	1.22E-01	NS	↓ -0.59	6.18E-01	NS	↑ 0.20	8.34E-01	NS	↓ -0.11	7.19E-01	NS	↓ -0.06
K7EQQ3	7.80E-01	NS	↑ 0.16	6.18E-01	NS	↑ 0.32	7.47E-01	NS	↑ 0.10	7.28E-01	NS	↓ -0.05	4.09E-01	NS	↑ 0.68	6.70E-01	NS	↑ 0.32	9.39E-01	NS	↓ -0.05
Q9BS19	8.70E-01	NS	↓ -0.07	9.61E-01	NS	↓ -0.03	8.40E-01	NS	↓ -0.03	9.67E-01	NS	↓ -0.03	6.06E-01	NS	↓ -0.13	6.91E-01	NS	↓ -0.08	9.99E-01	NS	↑ 0.00
B0YIW2	7.52E-01	NS	↑ 0.41	7.30E-01	NS	↑ 0.58	5.75E-01	NS	↓ -0.11	3.77E-01	NS	↓ -0.12	7.09E-01	NS	↑ 0.12	3.86E-01	NS	↓ -0.03	3.65E-01	NS	↓ -0.09
B2CIS9	3.04E-01	NS	↓ -0.40	6.00E-01	NS	↓ -0.28	8.47E-01	NS	↑ 0.23	5.12E-01	NS	↑ 0.37	9.71E-01	NS	↓ -0.12	4.71E-01	NS	↑ 0.08	4.98E-01	NS	↓ 0.00
Q5JP69	2.25E-01	NS	↑ 0.24	5.39E-01	NS	↑ 0.21	7.75E-01	NS	↑ 0.15	4.21E-01	NS	↑ 0.29	9.72E-01	NS	↓ 0.00	2.71E-01	NS	↑ 0.24	5.99E-01	NS	↓ -0.09
A0A1B0GUU9	7.24E-01	NS	↓ -0.09	7.12E-01	NS	↓ -0.42	7.02E-01	NS	↓ -0.09	5.57E-01	NS	↓ -0.52	7.79E-01	NS	↓ -0.40	5.23E-01	NS	↑ 0.24	9.20E-01	NS	↓ -0.24
Q5JVE7	6.20E-01	NS	↑ 0.04	9.35E-01	NS	↓ -0.03	4.01E-01	NS	↑ 0.15	8.45E-01	NS	↓ -0.07	4.55E-01	NS	↑ 0.09	9.38E-01	NS	↓ -0.05	2.13E-01	NS	↑ 0.21
I3L145	4.55E-01	NS	↓ -0.29	6.02E-01	NS	↓ -0.23	6.48E-01	NS	↑ 0.05	2.87E-01	NS	↓ -0.42	2.46E-01	NS	↓ -0.45	9.90E-01	NS	↓ -0.06	5.45E-01	NS	↓ -0.21
C9JEU5	6.51E-01	NS	↑ 0.49	3.97E-02	NS	↑ 0.91	8.50E-01	NS	↑ 0.80	8.99E-01	NS	↑ 0.26	5.39E-01	NS	↑ 0.36	1.58E-01	NS	↑ 0.81	5.57E-01	NS	↑ 0.20
E9KL23	2.67E-01	NS	↓ -0.44	2.73E-01	NS	↓ -0.36	4.29E-01	NS	↓ -0.09	2.12E-01	NS	↓ -0.42	5.25E-01	NS	↓ -0.45	5.51E-01	NS	↓ -0.03	8.96E-01	NS	↑ 0.11
A0A0G2JPA8	1.72E-01	NS	↓ -0.24	3.23E-01	NS	↓ -0.18	1.18E-01	NS	↓ -0.32	2.59E-01	NS	↓ -0.21	4.93E-01	NS	↓ -0.13	8.05E-01	NS	↑ 0.03	9.98E-01	NS	↑ 0.01
B2R950	7.40E-01	NS	↓ -0.17	9.88E-01	NS	↓ -0.02	5.19E-01	NS	↑ 0.15	7.28E-01	NS	↓ -0.16	6.26E-01	NS	↓ -0.24	8.67E-01	NS	↑ 0.02	7.02E-01	NS	↓ -0.16
A0A024R035	6.88E-01	NS	↓ -0.12	8.07E-01	NS	↓ -0.06	7.06E-01	NS	↑ 0.09	7.94E-01	NS	↑ 0.06	9.28E-01	NS	↑ 0.07	5.85E-01	NS	↑ 0.13	6.16E-01	NS	↑ 0.11
Q6LAN8	9.72E-01	NS	↑ 0.09	9.58E-01	NS	↓ -0.08	5.34E-01	NS	↓ -0.37	8.86E-01	NS	↑ 0.18	9.00E-01	NS	↑ 0.19	3.48E-01	NS	↓ -0.66	8.23E-01	NS	↓ -0.09
Q05CV2	9.84E-01	NS	↑ 0.01	5.79E-01	NS	↓ -0.12	3.96E-01	NS	↑ 0.34	3.87E-01	NS	↑ 0.17	5.39E-01	NS	↓ -0.15	4.71E-01	NS	↑ 0.20	7.17E-01	NS	↓ -0.08
Q13784	5.69E-01	NS	↓ -0.29	8.19E-01	NS	↓ -0.11	3.65E-01	NS	↓ -0.43	7.05E-01	NS	↓ -0.21	8.85E-01	NS	↓ -0.01	7.35E-01	NS	↓ -0.12	3.22E-01	NS	↑ 0.63
A0N071	9.27E-01	NS	↓ -0.27	4.33E-01	NS	↑ 1.08	1.12E-01	NS	↑ 1.52	3.12E-01	NS	↑ 0.20	1.03E-01	NS	↑ 1.32	3.23E-02	NS	↑ 2.22	3.62E-02	NS	↑ 2.44
A0A0J9YY65	2.28E-01	NS	↓ -0.22	8.77E-02	NS	↓ -0.30	9.84E-02	NS	↑ 0.45	9.31E-01	NS	↑ 0.02	7.92E-01	NS	↓ -0.04	3.74E-02	NS	↑ 0.46	2.58E-01	NS	↑ 0.31
B0YJC6	4.81E-01	NS	↑ 0.19	7.77E-01	NS	↓ -0.07	4.36E-01	NS	↑ 0.22	7.09E-01	NS	↑ 0.05	2.17E-01	NS	↑ 0.34	5.00E-01	NS	↓ -0.18	8.99E-01	NS	↓ -0.03
Q8NBH6	5.49E-01	NS	↓ -0.11	7.13E-01	NS	↓ -0.05	4.19E-01	NS	↑ 0.24	7.52E-01	NS	↓ -0.02	7.74E-01	NS	↓ -0.07	5.51E-01	NS	↑ 0.16	8.34E-01	NS	↓ -0.01
B2R7F8	2.30E-01	NS	↑ 0.18	9.32E-01	NS	↓ -0.03	4.98E-01	NS	↑ 0.14	2.09E-01	NS	↑ 0.20	9.92E-01	NS	↓ -0.03	7.82E-01	NS	↑ 0.07	9.51E-01	NS	↓ -0.02
B2RAN2	4.88E-01	NS	↑ 0.27	1.97E-01	NS	↑ 0.25	4.04E-01	NS	↓ -0.17	8.60E-01	NS	↑ 0.07	6.19E-01	NS	↑ 0.18	3.39E-01	NS	↓ -0.19	5.28E-01	NS	↓ -0.13
A0A0F7G8J1	7.69E-01	NS	↑ 0.04	7.27E-01	NS	↓ -0.05	1.19E-01	NS	↑ 0.35	2.58E-01	NS	↑ 0.31	1.81E-01	NS	↓ -0.26	6.12E-01	NS	↑ 0.21	5.01E-01	NS	↑ 0.15
A0A087X1J7	6.09E-01	NS	↓ -0.16	4.87E-01	NS	↓ -0.20	6.32E-01	NS	↑ 0.11	9.21E-01	NS	↓ -0.03	8.41E-01	NS	↓ -0.12	1.56E-01	NS	↑ 0.31	9.40E-01	NS	↓ -0.06
H0YAC1	5.72E-01	NS	↑ 0.09	4.52E-01	NS	↑ 0.09	5.97E-01	NS	↑ 0.08	5.25E-01	NS	↑ 0.12	8.27E-01	NS	↑ 0.02	8.60E-01	NS	↓ -0.03	2.98E-01	NS	↑ 0.26
A0A024R962	3.69E-01	NS	↑ 0.36	2.88E-01	NS	↑ 0.28	8.44E-01	NS	↓ -0.25	7.27E-01	NS	↑ 0.07	2.57E-01	NS	↑ 0.39	5.21E-01	NS	↓ -0.50	9.55E-01	NS	↑ 0.07
B2R6W1	2.62E-01	NS	↓ -0.20	2.29E-01	NS	↓ -0.21	3.48E-01	NS	↑ 0.13	7.59E-01	NS	↓ -0.06	3.26E-02	NS	↓ -0.32	7.93E-01	NS	↑ 0.06	9.26E-01	NS	↑ 0.02
A0A384MDV8	5.67E-01	NS	↓ -0.08	4.78E-01	NS	↓ -0.11	1.36E-01	NS	↓ -0.33	3.28E-01	NS	↓ -0.16	1.80E-01	NS	↓ -0.20	9.10E-01	NS	↑ 0.09	3.46E-01	NS	↓ -0.14
V9HW98	4.37E-01	NS	↑ 0.34	1.37E-01	NS	↑ 0.52	5.52E-01	NS	↑ 0.30	6.79E-01	NS	↑ 0.17	7.15E-01	NS	↑ 0.33	6.84E-01	NS	↓ -0.19	9.95E-01	NS	↑ 0.15
B4E1Z4	6.20E-01	NS	↓ -0.09	5.25E-01	NS	↓ -0.11	9.76E-01	NS	↑ 0.00	5.33E-01	NS	↑ 0.07	9.91E-01	NS	↑ 0.02	9.16E-01	NS	↓ -0.03	8.12E-01	NS	↓ -0.05
D9YZU5	2.68E-01	NS	↓ -0.59	8.32E-01	NS	↑ 0.15	8.51E-01	NS	↓ -0.07	9.19E-01	NS	↓ -0.01	4.89E-01	NS	↑ 0.72	7.14E-02	NS	↑ 1.17	1.56E-01	NS	↑ 1.38
B2R888	7.28E-01	NS	↓ -0.05	5.18E-01	NS	↓ -0.11	1.22E-01	NS	↑ 0.54	6.99E-01	NS	↓ -0.05	6.66E-01	NS	↑ 0.05	3.80E-02	NS	↑ 0.43	5.87E-01	NS	↑ 0.20
F8W7L3	9.65E-01	NS	↓ -0.08	5.24E-01	NS	↑ 0.34	6.80E-01	NS	↓ -0.10	7.48E-01	NS	↑ 0.06	2.66E-01	NS	↑ 0.33	7.96E-01	NS	↓ -0.23	8.88E-01	NS	↓ -0.36
Q59EZ3	4.32E-02	NS	↑ 1.17	4.40E-01	NS	↓ -0.16	1.64E-01	NS	↑ 1.17	2.53E-01	NS	↑ 0.79	8.51E-01	NS	↓ -0.04	6.17E-01	NS	↑ 0.59	2.34E-01	NS	↑ 0.57
Q9UNL6	2.87E-01	NS	↓ -0.55	7.02E-01	NS	↓ -0.34	7.14E-01	NS	↓ -0.61	7.27E-01	NS	↓ -0.03	8.99E-01	NS	↑ 0.06	9.52E-02	NS	↑ 1.28	5.50E-01	NS	↑ 0.42
B4DZ36	7.98E-01	NS	↑ 0.14	7.09E-01	NS	↑ 0.15	5.34E-01	NS	↑ 0.40	5.55E-01	NS	↑ 0.23	9.32E-01	NS	↓ -0.02	8.29E-01	NS	↑ 0.09	9.81E-01	NS	↑ 0.05
A0A024RAG6	9.92E-01	NS	↑ 0.04	2.00E-01	NS	↓ -0.35	8.94E-01	NS	↓ -0.04	1.08E-01	NS	↓ -0.54	2.23E-01	NS	↓ -0.33	4.54E-01	NS	↓ -0.16	1.23E-01	NS	↓ -0.63
Q9UEU7	5.24E-01	NS	↑ 0.65	3.79E-01	NS	↑ 0.42	9.58E-01	NS	↓ -0.07	4.99E-01	NS	↑ 0.60	6.61E-01	NS	↑ 0.19	7.86E-01	NS	↑ 0.09	8.25E-01	NS	↑ 0.03
A0A384NKS6	1.52E-01	NS	↓ -0.26	5.05E-02	NS	↓ -0.31	1.00E+00	NS	↑ 0.01	2.59E-01	NS	↓ -0.18	1.94E-01	NS	↓ -0.20	3.68E-01	NS	↓ -0.14	8.31E-02	NS	↓ -0.31

name	1 d/Pre p-value	FDR	log2(FC)	2 d/Pre p-value	FDR	log2(FC)	3 d/Pre p-value	FDR	log2(FC)	7 d/Pre p-value	FDR	log2(FC)	14 d/Pre p-value	FDR	log2(FC)	21 d/Pre p-value	FDR	log2(FC)
X6RJP6	9.33E-02	NS	↑ 2.15	1.20E-02	NS	↑ 1.56	8.43E-01	NS	↓ -0.17	4.47E-01	NS	↑ 0.63	1.28E-02	NS	↑ 1.10	5.78E-02	NS	↑ 1.96
I3L3D5	1.80E-01	NS	↑ 1.55	2.45E-01	NS	↑ 0.78	1.94E-01	NS	↓ -0.69	7.62E-01	NS	↑ 0.04	1.57E-01	NS	↓ -0.50	2.43E-01	NS	↑ 1.35
Q53GK6	1.47E-02	NS	↑ 2.03	2.71E-01	NS	↑ 0.74	3.71E-01	NS	↑ 0.23	6.03E-01	NS	↑ 0.04	2.13E-01	NS	↑ 0.25	1.44E-01	NS	↑ 1.11
B4E3P1	2.69E-02	NS	↑ 2.06	1.93E-01	NS	↑ 0.56	8.99E-01	NS	↓ -0.28	7.16E-01	NS	↓ -0.41	9.50E-01	NS	↓ -0.19	1.97E-01	NS	↑ 0.98
F2RM35	7.42E-01	NS	↓ -0.05	6.92E-01	NS	↓ -0.04	3.56E-01	NS	↓ -0.16	4.35E-02	NS	↓ -0.31	5.59E-02	NS	↓ -0.33	1.43E-01	NS	↓ -0.31
B3KT06	1.06E-01	NS	↑ 4.01	6.83E-02	NS	↑ 2.46	2.56E-01	NS	↓ -0.47	1.73E-01	NS	↑ 2.93	2.05E-01	NS	↑ 2.12	5.58E-01	NS	↑ 1.90
A5PL27	7.99E-02	NS	↑ 0.79	5.17E-01	NS	↑ 0.30	8.66E-02	NS	↑ 0.78	6.71E-02	NS	↑ 0.79	6.72E-02	NS	↑ 0.87	2.23E-01	NS	↑ 0.47
D0PNI1	2.40E-02	NS	↑ 1.33	3.14E-01	NS	↓ -0.54	7.45E-02	NS	↓ -1.10	3.74E-01	NS	↑ 0.05	9.92E-01	NS	↓ -0.15	4.82E-01	NS	↑ 0.37
F8W876	6.53E-01	NS	↑ 0.18	9.16E-01	NS	↑ 0.02	3.15E-01	NS	↑ 0.18	1.02E-01	NS	↑ 0.27	2.27E-01	NS	↑ 0.21	5.07E-01	NS	↑ 0.34
Q53H26	7.82E-01	NS	↑ 0.29	6.92E-01	NS	↑ 0.14	4.65E-01	NS	↓ -0.19	7.88E-01	NS	↑ 0.13	6.22E-01	NS	↑ 0.18	1.86E-01	NS	↓ -0.43
V9HWE9	8.32E-03	NS	↑ 1.52	3.55E-01	NS	↑ 0.28	6.51E-01	NS	↓ -0.47	6.49E-01	NS	↑ 0.41	3.93E-01	NS	↑ 0.35	1.73E-01	NS	↑ 0.42
B4DDT3	9.35E-01	NS	↑ 0.16	4.76E-01	NS	↓ -0.16	6.44E-01	NS	↓ -0.07	5.47E-01	NS	↓ -0.14	2.33E-01	NS	↑ 0.22	9.82E-01	NS	↑ 0.03
B7Z555	4.30E-01	NS	↑ 0.23	4.83E-01	NS	↑ 0.15	3.88E-01	NS	↑ 0.24	9.42E-02	NS	↑ 0.31	4.87E-01	NS	↑ 0.27	5.23E-01	NS	↑ 0.21
F8W0J6	5.59E-03	NS	↑ 1.01	9.62E-01	NS	↑ 0.49	4.57E-01	NS	↑ 0.54	1.90E-01	NS	↑ 0.52	5.16E-02	NS	↑ 0.72	2.38E-01	NS	↑ 0.81
B1AP58	1.89E-01	NS	↑ 0.41	3.36E-01	NS	↑ 0.29	4.23E-01	NS	↑ 0.27	7.73E-01	NS	↑ 0.10	2.73E-01	NS	↑ 0.37	7.57E-01	NS	↑ 0.09
Q53H37	3.62E-01	NS	↑ 1.36	5.72E-01	NS	↓ -0.35	4.04E-02	NS	↓ -1.41	1.53E-01	NS	↓ -0.64	5.96E-03	NS	↓ -1.90	1.93E-01	NS	↓ -0.34
V9HWN8	6.93E-01	NS	↑ 0.01	8.81E-01	NS	↓ -0.01	2.26E-01	NS	↑ 0.23	1.29E-01	NS	↑ 0.31	2.20E-01	NS	↑ 0.23	1.09E-01	NS	↑ 0.49
H3BRJ9	9.22E-01	NS	↑ 0.01	6.54E-01	NS	↓ -0.14	7.59E-01	NS	↑ 0.43	3.90E-01	NS	↑ 0.25	4.26E-01	NS	↑ 0.41	4.72E-01	NS	↑ 0.57
D6R934	6.34E-01	NS	↓ -0.08	8.59E-01	NS	↓ -0.07	6.20E-01	NS	↑ 0.24	6.58E-01	NS	↓ -0.13	3.20E-01	NS	↑ 0.30	9.28E-01	NS	↑ 0.07
B2RA39	2.18E-01	NS	↑ 0.35	6.01E-01	NS	↑ 0.34	3.05E-01	NS	↓ -1.22	6.03E-01	NS	↓ -0.23	2.51E-01	NS	↓ -1.20	9.53E-01	NS	↓ -0.08
L8E853	9.15E-01	NS	↑ 0.14	4.74E-01	NS	↑ 0.27	2.73E-01	NS	↑ 0.25	3.68E-01	NS	↑ 0.27	1.98E-01	NS	↑ 0.41	6.36E-02	NS	↑ 0.50
E3UN46	3.73E-01	NS	↑ 0.15	9.41E-01	NS	↑ 0.04	4.89E-01	NS	↓ -0.34	9.12E-01	NS	↓ -0.14	2.64E-01	NS	↓ -0.42	7.81E-01	NS	↓ -0.16
A0A182DWH7	7.16E-02	NS	↓ -0.36	1.55E-01	NS	↓ -0.28	2.63E-02	NS	↓ -0.46	1.23E-02	NS	↓ -0.56	8.93E-02	NS	↓ -0.33	4.78E-02	NS	↓ -0.41
B7Z549	9.18E-01	NS	↓ 0.00	7.71E-01	NS	↑ 0.11	1.16E-01	NS	↑ 0.51	2.84E-01	NS	↑ 0.25	2.37E-01	NS	↑ 0.37	5.74E-01	NS	↑ 0.22
A0A024R9Q1	8.40E-01	NS	↑ 0.23	3.81E-01	NS	↓ -0.32	5.55E-01	NS	↓ -0.25	4.99E-01	NS	↓ -0.29	6.48E-01	NS	↑ 0.10	2.25E-01	NS	↑ 0.58
A0A087WSY5	9.79E-01	NS	↓ 0.00	8.21E-01	NS	↓ -0.07	3.62E-01	NS	↑ 0.16	3.31E-01	NS	↑ 0.19	9.78E-01	NS	↑ 0.04	5.01E-01	NS	↑ 0.18
E7END6	2.97E-01	NS	↓ -0.18	3.47E-01	NS	↓ -0.17	1.87E-01	NS	↓ -0.23	1.04E-01	NS	↓ -0.26	5.56E-01	NS	↓ -0.09	4.52E-01	NS	↓ -0.12
B7ZKJ8	7.20E-01	NS	↑ 0.07	7.51E-01	NS	↑ 0.10	1.55E-01	NS	↑ 0.29	2.88E-01	NS	↑ 0.30	1.33E-01	NS	↑ 0.33	1.97E-01	NS	↑ 0.22
D9ZGG2	1.57E-02	NS	↓ -0.37	3.61E-02	NS	↓ -0.33	8.20E-02	NS	↓ -0.27	1.09E-01	NS	↓ -0.22	5.88E-01	NS	↓ -0.08	7.89E-01	NS	↓ -0.05
G3V2W1	1.97E-01	NS	↓ -0.51	7.64E-01	NS	↓ -0.08	6.24E-01	NS	↑ 0.05	5.34E-01	NS	↓ -0.16	1.35E-01	NS	↓ -0.44	6.81E-01	NS	↑ 0.03
J3QR68	5.99E-01	NS	↑ 0.23	3.57E-01	NS	↓ -0.24	3.33E-01	NS	↓ -0.35	2.50E-01	NS	↓ -0.66	1.04E-01	NS	↓ -0.95	2.55E-01	NS	↓ -0.29
V9HWE3	7.25E-01	NS	↑ 0.17	8.43E-01	NS	↑ 0.47	9.44E-01	NS	↑ 0.19	3.53E-01	NS	↓ -0.25	9.80E-01	NS	↑ 0.24	6.33E-01	NS	↑ 0.39
B4E1C2	4.42E-01	NS	↓ -0.11	2.35E-01	NS	↓ -0.22	2.48E-02	NS	↓ -0.47	5.99E-02	NS	↓ -0.38	9.77E-02	NS	↓ -0.31	1.59E-01	NS	↓ -0.24
A0A087WW43	5.98E-01	NS	↑ 0.09	7.99E-01	NS	↑ 0.10	1.71E-01	NS	↑ 0.20	1.68E-01	NS	↑ 0.21	1.50E-01	NS	↑ 0.32	1.73E-01	NS	↑ 0.22
A2RTY6	9.76E-01	NS	↓ -0.03	8.96E-01	NS	↓ 0.00	4.21E-01	NS	↑ 0.22	4.55E-01	NS	↑ 0.18	2.54E-01	NS	↑ 0.29	6.05E-01	NS	↑ 0.18
A0A0U1RR20	4.70E-01	NS	↓ -0.08	4.11E-01	NS	↓ -0.05	7.44E-02	NS	↓ -0.75	2.43E-01	NS	↓ -0.23	1.92E-02	NS	↓ -1.00	1.70E-01	NS	↓ -0.49
E9PHK0	8.98E-01	NS	↑ 0.04	7.51E-01	NS	↓ -0.05	2.48E-01	NS	↓ -0.26	3.16E-01	NS	↓ -0.22	4.14E-01	NS	↓ -0.17	5.79E-01	NS	↓ -0.12
D3DNN4	6.05E-01	NS	↑ 0.62	8.56E-01	NS	↓ -0.01	2.70E-01	NS	↑ 0.91	2.71E-01	NS	↑ 0.15	3.27E-01	NS	↑ 0.53	2.50E-01	NS	↑ 0.57
Q499G5	3.49E-01	NS	↓ -0.20	3.46E-01	NS	↓ -0.14	7.42E-01	NS	↑ 0.24	6.39E-01	NS	↑ 0.03	7.36E-01	NS	↑ 0.19	5.32E-01	NS	↑ 0.22
A0A0S2Z3D5	1.46E-01	NS	↑ 1.05	9.19E-02	NS	↑ 0.68	2.10E-01	NS	↑ 0.85	3.34E-02	NS	↑ 1.02	1.40E-01	NS	↑ 0.62	5.46E-01	NS	↑ 0.33
RT-Cal protein	4.46E-02	NS	↓ -0.26	2.54E-01	NS	↓ -0.11	3.18E-01	NS	↓ -0.08	9.87E-01	NS	↑ 0.00	7.21E-01	NS	↑ 0.02	9.50E-01	NS	↑ 0.01
B4DRT3	8.38E-04	NS	↑ 2.07	8.18E-03	NS	↑ 1.42	1.18E-01	NS	↑ 0.79	5.64E-01	NS	↑ 0.42	6.77E-01	NS	↓ -0.12	3.25E-01	NS	↑ 0.33
A8K1K1	8.95E-01	NS	↑ 0.05	8.83E-01	NS	↑ 0.11	9.46E-01	NS	↑ 0.12	8.47E-01	NS	↓ -0.10	3.39E-01	NS	↑ 0.51	7.72E-01	NS	↑ 0.41
A0A140VK00	3.73E-01	NS	↓ -0.17	6.14E-01	NS	↓ -0.06	2.35E-01	NS	↓ -0.25	6.00E-02	NS	↓ -0.40	2.81E-01	NS	↓ -0.19	2.74E-01	NS	↓ -0.22
A0A140VJI7	7.82E-01	NS	↓ -0.14	1.62E-01	NS	↓ -0.41	5.88E-01	NS	↓ -0.20	4.34E-01	NS	↓ -0.22	5.96E-01	NS	↓ -0.15	7.92E-01	NS	↓ -0.12
A0A024RAA7	3.27E-01	NS	↓ -0.21	7.05E-01	NS	↓ -0.05	9.37E-01	NS	↑ 0.09	4.48E-01	NS	↓ -0.16	4.91E-01	NS	↑ 0.16	8.48E-01	NS	↑ 0.11
B3KS79	2.12E-01	NS	↑ 0.27	2.92E-01	NS	↑ 0.30	1.48E-01	NS	↑ 0.36	2.54E-01	NS	↑ 0.22	7.96E-01	NS	↑ 0.06	6.62E-01	NS	↑ 0.05
Q8TAY0	9.89E-01	NS	↑ 0.03	8.90E-01	NS	↑ 0.02	5.55E-01	NS	↑ 0.23	4.71E-01	NS	↑ 0.13	4.62E-01	NS	↑ 0.21	6.86E-01	NS	↑ 0.18
Q9H2E1	3.13E-01	NS	↑ 0.29	7.99E-01	NS	↓ -0.48	8.40E-01	NS	↓ -0.26	4.38E-01	NS	↑ 1.01	4.92E-01	NS	↑ 0.18	5.08E-01	NS	↑ 0.25
V9HWA9	7.39E-01	NS	↑ 0.06	5.42E-01	NS	↑ 0.10	2.94E-01	NS	↑ 0.25	1.96E-01	NS	↑ 0.32	2.76E-01	NS	↑ 0.24	3.66E-01	NS	↑ 0.21
F5GXS0	7.45E-01	NS	↑ 0.08	3.03E-01	NS	↑ 0.27	1.61E-01	NS	↑ 0.42	1.40E-01	NS	↑ 0.40	7.08E-02	NS	↑ 0.49	3.16E-01	NS	↑ 0.28
Q59GS8	8.66E-01	NS	↑ 0.02	4.05E-01	NS	↑ 0.22	5.12E-02	NS	↑ 0.42	5.02E-01	NS	↑ 0.13	2.73E-01	NS	↑ 0.25	4.22E-01	NS	↑ 0.18
A0A3G1HEF1	2.96E-01	NS	↑ 0.23	2.95E-01	NS	↑ 0.17	3.53E-01	NS	↑ 0.24	4.68E-02	NS	↑ 0.47	1.97E-01	NS	↑ 0.23	5.33E-01	NS	↑ 0.20
A0A3B3IS66	5.31E-01	NS	↓ -0.15	7.31E-01	NS	↓ -0.08	2.40E-01	NS	↓ -0.29	5.44E-02	NS	↓ -0.55	1.60E-01	NS	↓ -0.33	1.18E-01	NS	↓ -0.37
A0A024R3E3	9.67E-01	NS	↑ 0.56	6.21E-01	NS	↑ 0.77	4.71E-01	NS	↓ -0.73	8.91E-01	NS	↑ 0.49	9.60E-01	NS	↓ -0.20	4.23E-01	NS	↓ -0.58
Q5VY30	2.64E-01	NS	↓ -0.30	4.90E-01	NS	↓ -0.15	6.09E-01	NS	↓ -0.04	8.79E-01	NS	↑ 0.04	8.29E-01	NS	↓ -0.04	9.36E-01	NS	↓ -0.02
E9PIT3	5.81E-01	NS	↓ -0.24	8.20E-01	NS	↓ -0.13	5.25E-01	NS	↓ -0.26	8.78E-01	NS	↓ -0.12	7.98E-01	NS	↓ -0.17	8.98E-01	NS	↓ -0.13
A6XND1	1.76E-01	NS	↓ -0.34	2.47E-01	NS	↓ -0.31	2.34E-01	NS	↓ -0.31	1.40E-01	NS	↓ -0.40	1.54E-01	NS	↓ -0.36	1.13E-01	NS	↓ -0.40
A0A2S0BDD1	7.56E-01	NS	↑ 0.06	6.59E-01	NS	↓ -0.16	2.13E-01	NS	↑ 0.29	1.03E-01	NS	↑ 0.39	2.44E-01	NS	↑ 0.23	3.92E-01	NS	↑ 0.27
V9HVY1	6.14E-01	NS	↑ 0.53	5.50E-01	NS	↑ 0.35	3.28E-01	NS	↑ 0.62	2.89E-02	NS	↑ 0.78	5.44E-02	NS	↑ 1.06	9.24E-01	NS	↑ 0.28
A0A2Z4LCH4	9.62E-01	NS	↓ -0.14	6.24E-01	NS	↓ -0.25	6.47E-01	NS	↑ 0.13	5.47E-01	NS	↑ 0.14	3.51E-01	NS	↑ 0.19	4.78E-01	NS	↑ 0.35
J3K000	4.95E-01	NS	↑ 0.50	8.82E-01	NS	↑ 0.06	3.07E-01	NS	↑ 0.42	9.16E-01	NS	↑ 0.01	5.56E-01	NS	↓ -0.11	4.94E-01	NS	↑ 0.23
B4DVE1	8.56E-01	NS	↑ 0.49	7.23E-01	NS	↓ -0.11	6.65E-01	NS	↑ 0.07	1.16E-01	NS	↑ 0.42	3.22E-01	NS	↑ 0.34	4.19E-01	NS	↑ 0.25
D3DPG0	7.42E-01	NS	↑ 0.02	9.90E-01	NS	↓ -0.10	5.49E-01	NS	↑ 0.14	6.77E-01	NS	↓ -0.22	6.66E-01	NS	↑ 0.13	8.76E-01	NS	↑ 0.06
D9IWP9	4.41E-01	NS	↓ -0.27	9.36E-01	NS	↑ 0.06	8.33E-01	NS	↓ -0.14	4.04E-01	NS	↓ -0.28	1.98E-01	NS	↓ -0.42	3.93E-01	NS	↓ -0.30
M0R2W8	5.26E-01	NS	↑ 0.13	6.29E-01	NS	↑ 0.08	9.89E-01	NS	↓ -0.04	4.83E-01	NS	↓ -0.19	8.66E-01	NS	↑ 0.01	6.51E-01	NS	↓ -0.34
Q59E93	5.40E-01	NS	↑ 0.51	9.81E-01	NS	↑ 0.20	4.13E-01	NS	↑ 0.08	5.72E-01	NS	↓ -0.19	4.46E-01	NS	↑ 0.15	3.45E-01	NS	↑ 0.18
B2R815	9.08E-01	NS	↑ 0.02	7.71E-01	NS	↑ 0.03	6.22E-01	NS	↑ 0.11	6.47E-01	NS	↑ 0.10	7.75E-01	NS	↑ 0.05	8.45E-01	NS	↑ 0.08
B2R4M6	2.61E-02	NS	↑ 2.82	1.79E-02	NS	↑ 2.03	6.72E-01	NS	↑ 0.18	5.37E-01	NS	↓ -0.32	6.85E-01	NS	↓ -0.20	7.00E-01	NS	↑ 0.21
V9GYM3	4.95E-01	NS	↑ 0.44	5.06E-01	NS	↑ 0.58	3.13E-01	NS	↓ -2.26	8.94E-01	NS	↑ 0.13	4.80E-01	NS	↓ -1.05	7.48E-01	NS	↓ -0.66
B4DPQ0	9.30E-01	NS	↓ -0.01	9.13E-01	NS	↓ -0.01	3.81E-01	NS	↑ 0.14	9.46E-01	NS	↓ -0.03	3.56E-01	NS	↑ 0.16	8.64E-01	NS	↓ -0.01
Q5SQ08	6.98E-01	NS	↓ -0.08	7.39E-01	NS	↑ 0.06	1.14E-01	NS	↑ 0.40	2.85E-01	NS	↑ 0.26	3.50E-01	NS	↑ 0.28	7.65E-01	NS	↑ 0.10
B4DRY0	2.56E-01	NS	↑ 1.19	3.52E-01	NS	↑ 1.10	8.05E-01	NS	↓ -0.15	4.21E-01	NS	↓ -0.44	9.07E-01	NS	↓ -0.07	9.03E-02	NS	↑ 0.78
S4R3Y4	7.09E-01	NS	↑ 0.06	8.57E-01	NS	↑ 0.09	3.48E-01	NS	↓ -0.20	2.02E-01	NS	↓ -0.32	7.57E-01	NS	↑ 0.04	8.98E-01	NS	↓ -0.04
A0A0S2Z4I5	8.31E-01	NS	↑ 0.08	8.35E-01	NS	↓ -0.01	3.86E-01	NS	↓ -0.20	7.51E-01	NS	↓ -0.10	2.38E-01	NS	↓ -0.27	2.63E-01	NS	↓ -0.27
A0A0G2JPR0	7.94E-01	NS	↓ -0.05	2.56E-01	NS	↑ 0.26	3.10E-02	NS	↑ 0.56	2.67E-01	NS	↑ 0.27	2.29E-02	NS	↑ 0.46	4.22E-01	NS	↑ 0.14
H0YID6	5.72E-01	NS	↑ 0.71	8.57E-01	NS	↑ 1.08	7.53E-01	NS	↑ 0.04	6.30E-01	NS	↓ -0.44	1.47E-01	NS	↑ 0.89	1.56E-02	NS	↑ 1.63
V9HWD8	3.28E-01	NS	↓ -0.30	3.90E-01	NS	↓ -0.28	2.46E-01	NS	↓ -0.37	2.91E-01	NS	↓ -0.34	3.34E-01	NS	↓ -0.30	3.60E-01	NS	↓ -0.31
H6VRG1	4.10E-01	NS	↑ 0.47	5.57E-01	NS	↑ 0.90	7.23E-01	NS	↓ 0.00	2.88E-01	NS	↓ -0.43	2.11E-01	NS	↓ -0.51	1.35E-01	NS	↑ 0.79
P78492	2.03E-01	NS	↑ 0.16	8.67E-01	NS	↑ 0.05	1.65E-01	NS	↑ 0.23	3.73E-01	NS	↑ 0.12	3.28E-01	NS	↑ 0.20	3.09E-02	NS	↑ 0.32
D3JV41	4.16E-01	NS	↓ -0.13	5.35E-01	NS	↓ -0.06	1.24E-01	NS	↓ -0.31	5.39E-01	NS	↓ -0.09	9.88E-01	NS	↑ 0.09	5.21E-01	NS	↓ -0.14
Q59EP2	4.45E-03	NS	↑ 0.62	2.07E-02	NS	↑ 0.48	1.59E-02	NS	↑ 0.48	3.62E-01	NS	↑ 0.20	7.76E-01	NS	↑ 0.05	7.11E-01	NS	↓ -0.08
C0JYY2	7.64E-01	NS	↓ -0.07	3.95E-01	NS	↓ -0.18	9.57E-01	NS	↓ 0.00	5.27E-01	NS	↑ 0.13	2.62E-01	NS	↑ 0.17	3.12E-01	NS	↑ 0.17
A0A024R1X6	6.62E-01	NS	↑ 0.48	6.25E-01	NS	↑ 0.70	8.25E-01	NS	↓ -0.01	6.82E-01	NS	↓ -0.48	8.74E-01	NS	↓ -0.19	1.62E-01	NS	↑ 0.49
S4R471	6.40E-01	NS	↑ 0.12	5.74E-01	NS	↑ 0.16	7.68E-01	NS	↑ 0.06	9.93E-01	NS	↑ 0.00	2.07E-01	NS	↑ 0.31	8.14E-01	NS	↓ -0.06
A0A024R104	1.47E-01	NS	↓ -0.37	5.98E-02	NS	↓ -0.48	4.20E-01	NS	↓ -0.20	6.15E-01	NS	↓ -0.10	7.86E-01	NS	↓ -0.04	5.41E-01	NS	↓ -0.13
V9HW68	1.91E-01	NS	↑ 0.80	7.07E-01	NS	↑ 0.48	3.55E-01	NS	↓ -0.54	9.61E-01	NS	↑ 0.16	4.50E-01	NS	↓ -0.32	2.50E-01	NS	↓ -0.62
B7Z8Q5	9.46E-01	NS	↑ 0.01	5.48E-01	NS	↓ -0.37	9.26E-01	NS	↓ -0.11	5.87E-01	NS	↓ -0.38	9.51E-01	NS	↓ -0.19	5.93E-01	NS	↑ 0.22
A0A140VKF3	9.62E-01	NS	↓ -0.02	8.77E-01	NS	↓ -0.05	4.86E-01	NS	↑ 0.18	8.85E-01	NS	↓ -0.01	3.75E-01	NS	↑ 0.21	6.02E-01	NS	↑ 0.11
A0A0S2Z3E8	8.55E-01	NS	↓ -0.43	7.79E-01	NS	↓ -0.35	5.11E-01	NS	↑ 0.39	5.72E-01	NS	↑ 0.30	6.43E-01	NS	↓ -0.26	6.51E-01	NS	↑ 0.20
Q59EA3	7.02E-01	NS	↑ 0.01	4.80E-01	NS	↓ -0.52	8.11E-01	NS	↓ -0.36	3.19E-01	NS	↑ 0.19	8.86E-01	NS	↓ -0.31	8.13E-01	NS	↓ -0.08
K7ERE3	1.34E-01	NS	↑ 0.66	2.48E-01	NS	↑ 1.25	5.13E-01	NS	↓ -0.15	3.52E-01	NS	↑ 0.82	5.42E-01	NS	↓ -0.48	3.56E-01	NS	↑ 0.80
V9HWF6	7.70E-01	NS	↑ 0.59	7.47E-01	NS	↑ 0.78	9.24E-01	NS	↑ 0.77	6.52E-01	NS	↓ -1.23	3.26E-01	NS	↓ -0.58	3.12E-01	NS	↓ -0.44
B4DPH4	4.67E-01	NS	↓ -0.10	3.78E-01	NS	↓ -0.13	5.68E-01	NS	↑ 0.14	6.89E-01	NS	↑ 0.08	6.92E-01	NS	↑ 0.06	3.05E-01	NS	↓ -0.15
C9JV77	4.66E-01	NS	↓ -0.19	1.57E-01	NS	↓ -0.48	3.66E-01	NS	↓ -0.23	1.71E-01	NS	↓ -0.46	8.08E-01	NS	↓ -0.06	6.05E-01	NS	↓ -0.17
A0A384MEF1	8.40E-01	NS	↑ 0.11	7.92E-01	NS	↑ 0.08	3.86E-01	NS	↑ 0.42	1.31E-01	NS	↑ 0.34	4.28E-01	NS	↑ 0.30	5.26E-01	NS	↑ 0.30
F8VV57	3.05E-01	NS	↑ 0.58	8.77E-01	NS	↑ 0.46	7.70E-01	NS	↑ 0.04	2.63E-01	NS	↓ -0.43	5.96E-02	NS	↓ -0.68	2.94E-01	NS	↑ 0.40
A0A384N669	9.72E-01	NS	↓ -0.10	7.65E-01	NS	↓ -0.15	3.36E-01	NS	↓ -0.44	2.89E-01	NS	↓ -0.46	5.55E-01	NS	↓ -0.29	5.22E-01	NS	↓ -0.32
Q4LE79	8.88E-01	NS	↓ -0.09	9.48E-01	NS	↓ -0.05	8.42E-01	NS	↑ 0.12	9.32E-01	NS	↓ -0.26	3.11E-01	NS	↓ -0.58	7.09E-01	NS	↓ -0.04
Q6MZL2	6.03E-01	NS	↑ 0.12	6.04E-01	NS	↓ -0.06	6.22E-01	NS	↓ -0.05	3.30E-01	NS	↓ -0.15	3.72E-01	NS	↓ -0.13	5.27E-01	NS	↓ 0.00
Q5J875	4.28E-01	NS	↓ -0.23	3.20E-01	NS	↓ -0.28	1.05E-01	NS	↓ -0.47	3.19E-01	NS	↓ -0.28	9.99E-01	NS	↑ 0.05	6.92E-01	NS	↑ 0.11
Q96K68	3.54E-01	NS	↑ 1.32	8.35E-01	NS	↓ -0.11	4.21E-01	NS	↓ -0.33	6.50E-01	NS	↓ -0.10	5.54E-01	NS	↓ -0.23	3.76E-01	NS	↓ -0.34
A0A0A0MRJ7	3.57E-01	NS	↓ -0.16	7.50E-01	NS	↓ -0.04	1.17E-01	NS	↓ -0.28	5.21E-02	NS	↓ -0.38	2.04E-01	NS	↓ -0.23	3.49E-01	NS	↓ -0.16
Q68CK4	8.13E-01	NS	↓ -0.01	9.04E-01	NS	↑ 0.06	3.18E-02	NS	↓ -0.55	1.60E-02	NS	↓ -0.43	2.90E-01	NS	↓ -0.15	2.16E-01	NS	↓ -0.20
B7ZLE5	9.45E-01	NS	↓ -0.04	9.43E-01	NS	↓ -0.15	6.43E-01	NS	↓ -0.06	4.70E-01	NS	↑ 0.16	5.70E-01	NS	↓ -0.42	4.88E-01	NS	↑ 0.25
D6RAR4	4.09E-01	NS	↓ -0.30	4.18E-01	NS	↓ -0.29	3.25E-01	NS	↓ -0.38	3.18E-01	NS	↓ -0.34	6.17E-01	NS	↓ -0.17	4.72E-01	NS	↓ -0.29
B2R773	8.06E-01	NS	↑ 0.35	4.00E-01	NS	↑ 1.94	4.39E-01	NS	↑ 0.48	8.93E-01	NS	↑ 0.13	8.63E-01	NS	↓ -0.05	9.52E-01	NS	↑ 0.15
A8K8Z4	9.60E-01	NS	↑ 0.01	9.58E-01	NS	↑ 0.03	7.16E-01	NS	↓ -0.05	5.46E-01	NS	↓ -0.08	8.81E-01	NS	↓ -0.02	4.09E-01	NS	↓ -0.11
F5H1C6	2.75E-01	NS	↑ 0.42	8.96E-01	NS	↓ -0.10	7.52E-01	NS	↓ -0.09	7.79E-01	NS	↑ 0.02	4.69E-01	NS	↑ 0.09	8.44E-01	NS	↓ -0.29
A0A024R6N9	2.31E-01	NS	↓ -0.21	4.16E-01	NS	↓ -0.13	1.87E-01	NS	↓ -0.25	1.85E-01	NS	↓ -0.25	5.27E-01	NS	↓ -0.09	5.21E-01	NS	↓ -0.09
Q53F26	1.23E-01	NS	↑ 0.51	7.58E-01	NS	↑ 0.09	3.06E-01	NS	↑ 0.46	3.98E-01	NS	↑ 0.65	2.00E-02	NS	↑ 0.91	5.13E-01	NS	↑ 0.39
B3KNX0	6.29E-01	NS	↑ 0.12	7.78E-01	NS	↑ 0.05	2.33E-01	NS	↑ 0.24	8.19E-01	NS	↑ 0.07	2.06E-01	NS	↑ 0.33	3.16E-01	NS	↑ 0.34
J3KPA1	7.66E-01	NS	↓ 0.00	9.03E-01	NS	↓ -0.03	2.03E-01	NS	↓ -0.37	3.51E-01	NS	↓ -0.26	3.66E-01	NS	↓ -0.22	4.94E-01	NS	↓ -0.17
Q53GN8	4.09E-01	NS	↓ -0.24	4.32E-01	NS	↓ -0.21	8.55E-01	NS	↑ 0.01	5.89E-01	NS	↑ 0.06	3.48E-01	NS	↑ 0.22	9.13E-01	NS	↑ 0.02
A0A1K0GXZ1	2.16E-01	NS	↑ 1.14	5.27E-01	NS	↑ 1.85	6.27E-01	NS	↓ -0.59	1.15E-01	NS	↓ -1.27	4.97E-01	NS	↓ -0.42	2.27E-01	NS	↓ -1.02
V9HWP0	6.27E-01	NS	↑ 0.24	5.67E-01	NS	↑ 0.15	9.31E-01	NS	↑ 0.00	8.48E-01	NS	↓ -0.11	7.21E-01	NS	↑ 0.11	2.73E-01	NS	↓ -0.44
A0A3B3ISJ1	3.12E-01	NS	↓ -0.13	2.92E-01	NS	↓ -0.13	8.78E-01	NS	↑ 0.01	6.25E-01	NS	↓ -0.05	3.13E-01	NS	↓ -0.11	4.43E-01	NS	↓ -0.08
Q8IVC0	2.25E-01	NS	↓ -0.23	4.85E-01	NS	↓ -0.12	4.60E-01	NS	↑ 0.13	6.39E-01	NS	↓ -0.05	6.94E-01	NS	↑ 0.11	6.43E-01	NS	↑ 0.07
E7EQ64	8.67E-01	NS	↑ 0.08	1.13E-01	NS	↓ -0.62	3.24E-01	NS	↓ -0.30	2.30E-01	NS	↓ -0.48	5.47E-01	NS	↓ -0.21	4.44E-01	NS	↓ -0.18
K7EQQ3	9.19E-01	NS	↑ 0.31	9.36E-01	NS	↑ 0.36	9.66E-01	NS	↑ 0.13	2.51E-01	NS	↓ -0.52	4.78E-01	NS	↓ -0.37	1.98E-01	NS	↑ 0.75
Q9BS19	9.33E-01	NS	↑ 0.02	8.85E-01	NS	↑ 0.07	5.70E-01	NS	↓ -0.14	4.00E-01	NS	↑ 0.24	8.31E-01	NS	↓ -0.05	9.48E-01	NS	↑ 0.02
B0YIW2	9.41E-02	NS	↓ -0.65	9.50E-01	NS	↑ 0.07	3.82E-01	NS	↓ -0.25	7.50E-01	NS	↑ 0.07	9.15E-01	NS	↑ 0.21	7.67E-01	NS	↓ -0.06
B2CIS9	7.55E-01	NS	↑ 0.42	5.85E-01	NS	↓ -0.25	5.31E-01	NS	↑ 0.07	4.67E-01	NS	↓ -0.20	9.83E-01	NS	↓ -0.11	9.51E-01	NS	↑ 0.05
Q5JP69	6.67E-01	NS	↓ -0.04	2.71E-01	NS	↑ 0.19	9.32E-01	NS	↑ 0.03	2.98E-01	NS	↑ 0.23	4.48E-01	NS	↑ 0.16	3.73E-01	NS	↑ 0.15
A0A1B0GUU9	3.49E-01	NS	↑ 0.33	4.71E-01	NS	↑ 0.19	7.09E-01	NS	↑ 0.21	9.69E-01	NS	↑ 0.09	3.97E-01	NS	↓ -0.54	9.43E-01	NS	↓ -0.34
Q5JVE7	7.55E-01	NS	↑ 0.08	2.37E-01	NS	↓ -0.31	3.67E-01	NS	↑ 0.13	9.82E-01	NS	↓ -0.02	2.91E-01	NS	↓ -0.28	3.40E-01	NS	↓ -0.25
I3L145	2.71E-01	NS	↓ -0.42	4.67E-01	NS	↓ -0.31	7.37E-01	NS	↓ -0.18	5.98E-01	NS	↓ -0.25	6.24E-01	NS	↓ -0.16	7.67E-01	NS	↑ 0.03
C9JEU5	1.66E-01	NS	↑ 0.84	5.03E-03	NS	↑ 1.06	2.47E-01	NS	↑ 0.67	5.06E-01	NS	↑ 0.43	9.45E-01	NS	↑ 0.14	5.91E-01	NS	↑ 0.27
E9KL23	4.56E-01	NS	↑ 0.48	9.92E-01	NS	↓ -0.07	3.81E-01	NS	↑ 0.82	4.71E-01	NS	↑ 0.10	7.55E-01	NS	↑ 0.12	2.89E-01	NS	↓ -0.49
A0A0G2JPA8	5.34E-01	NS	↓ -0.11	6.60E-01	NS	↓ -0.07	6.02E-01	NS	↑ 0.07	9.85E-01	NS	↓ -0.01	5.80E-01	NS	↑ 0.08	4.85E-01	NS	↑ 0.16
B2R950	7.29E-01	NS	↑ 0.14	8.54E-01	NS	↑ 0.00	7.74E-01	NS	↑ 0.16	8.76E-01	NS	↑ 0.13	7.41E-01	NS	↑ 0.12	7.18E-01	NS	↑ 0.08
A0A024R035	5.15E-01	NS	↑ 0.10	5.69E-01	NS	↑ 0.10	4.76E-01	NS	↑ 0.08	5.69E-01	NS	↑ 0.10	8.07E-01	NS	↑ 0.04	9.66E-01	NS	↓ -0.05
Q6LAN8	8.37E-01	NS	↓ -0.07	7.26E-01	NS	↓ -0.14	6.21E-01	NS	↓ -0.45	5.06E-01	NS	↓ -0.26	4.21E-01	NS	↓ -0.64	3.92E-01	NS	↓ -0.76
Q05CV2	7.00E-01	NS	↓ -0.07	6.60E-01	NS	↓ -0.09	5.22E-01	NS	↑ 0.17	2.65E-01	NS	↑ 0.21	4.04E-01	NS	↓ -0.18	9.36E-01	NS	↓ -0.01
Q13784	3.25E-01	NS	↑ 0.32	7.79E-01	NS	↑ 0.16	3.36E-01	NS	↑ 0.42	9.34E-01	NS	↓ 0.00	9.13E-01	NS	↓ -0.10	5.03E-01	NS	↓ -0.32
A0N071	9.36E-02	NS	↑ 1.08	3.85E-01	NS	↑ 1.01	8.28E-01	NS	↓ -0.32	1.80E-01	NS	↓ -1.32	7.85E-01	NS	↓ -0.53	8.96E-01	NS	↓ -0.50
A0A0J9YY65	4.37E-01	NS	↑ 0.25	9.97E-01	NS	↑ 0.01	8.23E-01	NS	↑ 0.12	3.51E-01	NS	↑ 0.17	9.50E-01	NS	↓ -0.02	2.65E-01	NS	↑ 0.35
B0YJC6	7.81E-01	NS	↓ -0.06	2.94E-01	NS	↓ -0.37	5.74E-01	NS	↓ -0.09	9.13E-01	NS	↓ -0.06	9.48E-01	NS	↓ -0.09	5.81E-01	NS	↑ 0.07
Q8NBH6	4.54E-01	NS	↓ -0.12	3.69E-01	NS	↓ -0.18	8.47E-01	NS	↓ -0.02	8.93E-01	NS	↑ 0.09	3.25E-01	NS	↑ 0.31	9.78E-01	NS	↑ 0.03
B2R7F8	7.36E-01	NS	↑ 0.08	9.92E-01	NS	↓ -0.02	7.85E-01	NS	↓ -0.06	2.96E-01	NS	↑ 0.18	6.81E-01	NS	↓ -0.09	9.85E-01	NS	↑ 0.00
B2RAN2	3.85E-01	NS	↓ -0.15	5.18E-01	NS	↓ -0.13	6.86E-02	NS	↓ -0.38	2.98E-01	NS	↓ -0.21	3.22E-01	NS	↓ -0.20	1.77E-01	NS	↓ -0.29
A0A0F7G8J1	7.81E-01	NS	↑ 0.14	8.24E-01	NS	↑ 0.02	2.40E-01	NS	↑ 0.27	9.70E-02	NS	↑ 0.26	4.22E-01	NS	↑ 0.15	6.28E-01	NS	↓ -0.07
A0A087X1J7	9.44E-01	NS	↑ 0.03	4.22E-01	NS	↓ -0.23	6.60E-01	NS	↑ 0.12	7.53E-01	NS	↑ 0.04	4.98E-01	NS	↑ 0.14	8.71E-01	NS	↑ 0.08
H0YAC1	2.44E-01	NS	↑ 0.19	9.06E-01	NS	↓ 0.00	9.82E-01	NS	↓ -0.02	2.92E-01	NS	↑ 0.26	9.32E-01	NS	↓ -0.02	4.33E-01	NS	↑ 0.13
A0A024R962	8.97E-01	NS	↓ -0.05	6.25E-01	NS	↓ -0.22	7.25E-01	NS	↑ 0.03	7.19E-01	NS	↑ 0.15	7.15E-01	NS	↑ 0.01	7.83E-01	NS	↓ -0.07
B2R6W1	4.04E-01	NS	↓ -0.14	2.68E-01	NS	↓ -0.18	9.85E-01	NS	↑ 0.01	8.22E-01	NS	↓ -0.03	8.32E-01	NS	↓ -0.03	5.35E-01	NS	↓ -0.10
A0A384MDV8	5.55E-01	NS	↑ 0.12	3.29E-01	NS	↑ 0.20	7.59E-01	NS	↑ 0.01	8.00E-01	NS	↑ 0.06	9.61E-01	NS	↑ 0.09	6.25E-01	NS	↓ -0.05
V9HW98	2.64E-01	NS	↑ 0.36	8.87E-01	NS	↑ 0.05	8.58E-01	NS	↓ -0.01	3.67E-01	NS	↓ -0.33	6.07E-01	NS	↑ 0.24	2.82E-01	NS	↓ -0.37
B4E1Z4	9.73E-01	NS	↑ 0.00	3.09E-01	NS	↓ -0.17	3.28E-01	NS	↓ -0.16	6.88E-01	NS	↑ 0.10	9.24E-01	NS	↑ 0.01	7.59E-01	NS	↑ 0.03
D9YZU5	2.45E-01	NS	↑ 0.86	5.64E-01	NS	↑ 0.95	4.26E-01	NS	↓ -1.17	2.80E-02	NS	↓ -2.20	3.24E-01	NS	↓ -0.96	1.40E-01	NS	↓ -1.62
B2R888	3.93E-02	NS	↑ 0.30	8.10E-02	NS	↑ 0.31	6.05E-02	NS	↑ 0.32	9.65E-01	NS	↓ 0.00	6.34E-01	NS	↓ -0.08	4.20E-01	NS	↓ -0.14
F8W7L3	8.52E-01	NS	↑ 0.34	1.12E-01	NS	↑ 0.86	9.27E-01	NS	↓ -0.31	5.52E-01	NS	↑ 0.29	9.21E-01	NS	↓ -0.25	7.99E-01	NS	↓ -0.04
Q59EZ3	6.59E-02	NS	↑ 0.80	9.57E-02	NS	↑ 0.65	6.49E-01	NS	↓ -0.15	5.07E-01	NS	↑ 0.28	1.98E-01	NS	↓ -0.41	1.75E-01	NS	↓ -0.56
Q9UNL6	4.44E-01	NS	↑ 0.33	6.91E-01	NS	↑ 0.61	7.30E-01	NS	↓ -0.41	4.52E-01	NS	↓ -0.80	9.17E-01	NS	↓ -0.35	9.97E-02	NS	↓ -1.28
B4DZ36	8.20E-01	NS	↑ 0.11	7.60E-01	NS	↓ -0.14	6.12E-01	NS	↓ -0.17	5.39E-01	NS	↑ 0.14	9.91E-01	NS	↑ 0.08	6.30E-01	NS	↑ 0.27
A0A024RAG6	2.23E-01	NS	↓ -0.34	5.50E-01	NS	↓ -0.17	4.47E-01	NS	↓ -0.14	3.29E-01	NS	↓ -0.22	6.95E-01	NS	↓ -0.06	3.94E-01	NS	↓ -0.19
Q9UEU7	7.56E-01	NS	↑ 0.22	8.63E-01	NS	↑ 0.29	6.73E-01	NS	↑ 0.20	4.00E-01	NS	↑ 0.43	3.79E-01	NS	↑ 0.31	5.78E-01	NS	↑ 0.18
A0A384NKS6	5.70E-02	NS	↓ -0.40	2.06E-02	NS	↓ -0.33	1.84E-01	NS	↓ -0.23	2.83E-01	NS	↓ -0.19	7.10E-01	NS	↓ -0.05	2.02E-01	NS	↓ -0.21
